# Self-analysis of repeat proteins reveals evolutionarily conserved patterns

**DOI:** 10.1186/s12859-020-3493-y

**Published:** 2020-05-07

**Authors:** Matthew Merski, Krzysztof Młynarczyk, Jan Ludwiczak, Jakub Skrzeczkowski, Stanisław Dunin-Horkawicz, Maria W. Górna

**Affiliations:** 1 Structural Biology Group, Biological and Chemical Research Centre, Department of Chemistry, University of Warsaw, Warsaw, Poland; 2 Laboratory of Structural Bioinformatics, Centre of New Technologies, University of Warsaw, Warsaw, Poland; 3 Laboratory of Bioinformatics, Nencki Institute of Experimental Biology, Warsaw, Poland

**Keywords:** Protein repeat, Repeat identification, Structural bioinformatics, Protein evolution

## Abstract

**Background:**

Protein repeats can confound sequence analyses because the repetitiveness of their amino acid sequences lead to difficulties in identifying whether similar repeats are due to convergent or divergent evolution. We noted that the patterns derived from traditional “dot plot” protein sequence self-similarity analysis tended to be conserved in sets of related repeat proteins and this conservation could be quantitated using a Jaccard metric.

**Results:**

Comparison of these dot plots obviated the issues due to sequence similarity for analysis of repeat proteins. A high Jaccard similarity score was suggestive of a conserved relationship between closely related repeat proteins. The dot plot patterns decayed quickly in the absence of selective pressure with an expected loss of 50% of Jaccard similarity due to a loss of 8.2% sequence identity. To perform method testing, we assembled a standard set of 79 repeat proteins representing all the subgroups in RepeatsDB. Comparison of known repeat and non-repeat proteins from the PDB suggested that the information content in dot plots could be used to identify repeat proteins from pure sequence with no requirement for structural information. Analysis of the UniRef90 database suggested that 16.9% of all known proteins could be classified as repeat proteins. These 13.3 million putative repeat protein chains were clustered and a significant amount (82.9%) of clusters containing between 5 and 200 members were of a single functional type.

**Conclusions:**

Dot plot analysis of repeat proteins attempts to obviate issues that arise due to the sequence degeneracy of repeat proteins. These results show that this kind of analysis can efficiently be applied to analyze repeat proteins on a large scale.

## Background

The relationship between protein sequences and structures has long been a widely accepted tenet of biochemistry [1]. However this is not without noted exceptions as proteins that share high sequence identity typically have nearly identical structures, whereas proteins of similar structures are not required to share any sequence identity [2]. Similarly, while evolutionarily conserved structures are typically associated with evolutionarily conserved protein sequences, conserved sequences themselves are not obliged to maintain a structure [3] as many intrinsically disordered regions in proteins maintain evolutionarily conserved sequences with sequence entropies [4] that are high enough to be statistically indistinguishable from those of structured regions [5]. On the other hand, there are also regions of low-entropy/low complexity sequence regions (LCR) in which stretches of sequence are dominated by clumps of one or a few amino acid types which can be structured or unstructured [6, 7]. Rounding out all these exceptions to the sequence/structure rule are protein repeat (PR) domains which are comprised of 3–25 sets of 20–40 residue long sections of repeated sequence [8] which can be either structured [9] or unstructured [10].

Despite this apparent sequence simplicity, repeat proteins are broadly distributed across the tree of life and participate in a wide range of functional roles including but not limited to virulence [11], organelle regulation [12], nucleotide binding [13], antiviral response [14] and signal transduction [15]. Estimates suggest that up to 25% of all proteins contain some kind of protein repeat [16, 17]. And although repeat proteins are more common in eukaryotes than prokaryotes [18], a survey found that 81% of archaeal and 96% of bacterial taxa contained at least one tetratricopeptide repeat (TPR) protein, and 81% of archaeal and 78% of bacterial taxa contained at least one Armadillo repeat (ARM) domain containing protein within their genomes [19], although functional differences identified between eukaryotic and prokaryotic repeat proteins have suggested that they may have had separate evolutionary origins [17, 18].

Sequence-based analysis of repeat proteins is particularly difficult and the repetitive, highly degenerate sequences found in repeat proteins can and do frustrate standard bioinformatics analyses [20]. For example, the two-helix TPR repeat, widely dispersed in both prokaryotes and eukaryotes [19], was originally defined by a conserved 34 amino acid motif tetratricopeptide repeat (TPR) [21] although later analysis demonstrated that these repeats are comprised of a convergent pattern of large and small hydrophobic amino acids [22] and examples of TPR proteins with up to 42 amino acid repeats have been identified [23]. Furthermore, a recent re-analysis of a set of proteins that had been positively identified as three-helix armadillo (ARM) repeat-containing proteins showed that 25 out of 95 of the examined proteins actually contained two-helix HEAT repeats [24] possibly due to a shared evolutionary origin [25]. Even when they can be correctly identified, repeats do not necessarily occur in integer numbers nor must repeat length always be consistent [26–28]. This also confounds repeat identification techniques as a recent survey found very low (0.2%) consensus identification between four established methods [20]. Even the rate of sequence change in repeat proteins is controversial as it was found that 61% of repeats in humans were conserved to at least the base of mammals [29] while others were found to be highly variable [10, 11, 27, 30]. In general, sequence-based rule sets for repeats have been difficult to apply universally. Fortunately, repeat proteins of known structure have been collected into RepeatsDB [9] which is subdivided into 5 structural groups with 23 subgroups based on the length of the repeating unit [8]. However, the fact that the vast majority of repeat proteins do not have experimentally determined structures, combined with their highly degenerate sequences makes it difficult to differentiate evolutionary convergence from common ancestry [25].

**Fig. 1:**
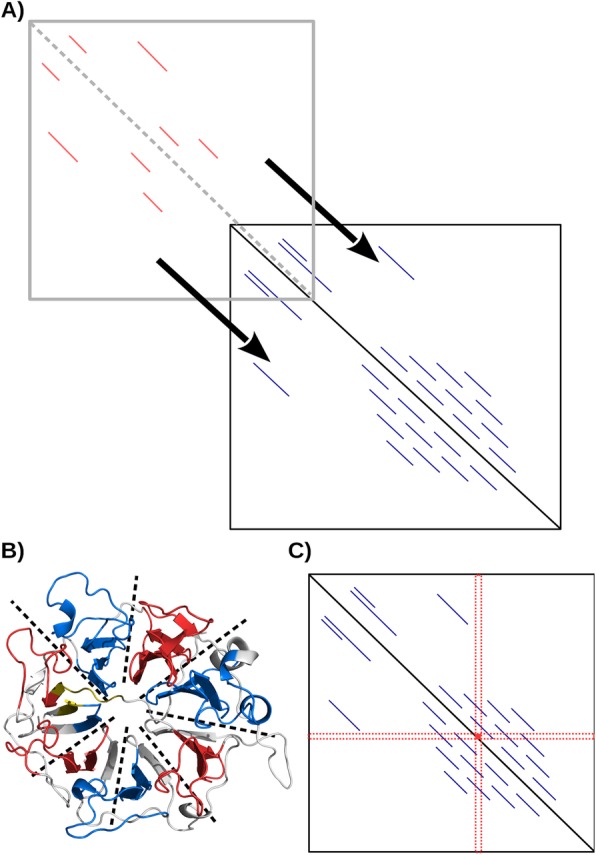
Illustration of the methodological analysis of repeat proteins. **a** A repeat protein fingerprint (red) “sliding” over a second one (blue). At each point, *J*_*X*_ is calculated to find the optimal overlap between the two proteins. The center black line is the self identity line. The length of the repeating sequence and gaps between them are indicated by line length and gap length respectively. The spacing between a colored line and the black identity line indicates the distance between the pairs of repeating sequences. **b** Highlighting of repeats in the seven-bladed human regulator of chromosome condensation protein (PDB ID:1a12) detected by the fingerprint method using a multiple sequence alignment. The protein is colored grey while the putative repeats are indicated in red and blue (alternating). The five residues before the first repeat and after the last repeat are indicated in yellow. Black dashed lines serve as a visual aids to help identify the 7 propeller blades. **c** Deconvolution of the dot plots by reading the indices (red) of each residue also allows reconstruction of the repeats

While it is difficult to compare different repeat proteins, repeats within a single protein necessarily have an analyzable informational relationship between themselves (i.e. it is tautological that a repeat is similar to its sibling repeats). We endeavored to find a method to analyze these informational relationships. After examining several methods we decided to focus on the repeat-detecting, self-comparison program DOTTER [31] (Fig. 1) when we noticed that the dot plots produced were highly similar in related proteins despite millions of years of species divergence and fairly low global sequence similarity. This similarity likely takes into account both the functional and physical constraints on the protein as well as an inertial drag on sequence divergence between related species. DOTTER analysis of repeat proteins provides a fast method of clustering repeat proteins by taking advantage of the extra complexity of sparse 2D matrices over linear sequences. This method is robust to sequence degeneracy and does not require access to experimental structural information. Because these dot plots are readily analyzable by modern computers using a simple Jaccard metric (i.e. the intersection over union), we were able to analyze and cluster the galaxy of known protein sequences within the UniRef90 database [32].

## Results

### Complex patterns of repeats exist in repeat proteins and are fairly common

**Fig. 2:**
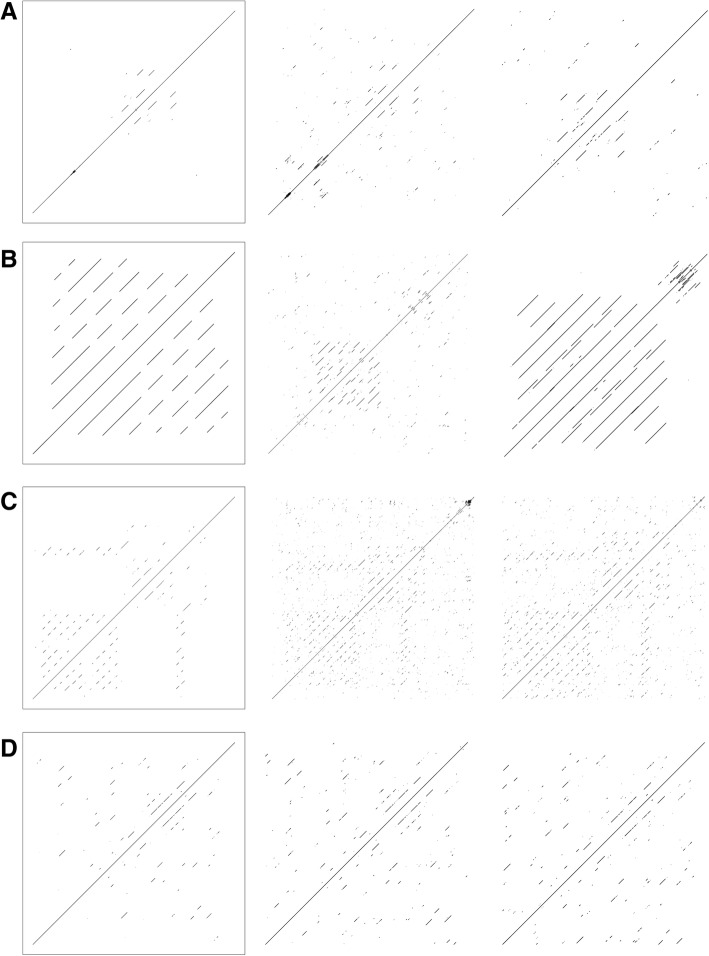
Dot plot patterns are maintained over evolutionary time in repeat proteins. For all sets of images, the leftmost figure is the consensus figure made from a set of related proteins. Black pixels indicate a DOTTER score of ≥31. **A)** An arrow like structure is evident in the consensus (left) and homologs of the plant RAP protein (no structure currently but reported to contain OPR repeats) among the vascular plants from the flowering plant (*S. tuberosum*, center) and is also evident in the earlier diverged species such as the byrophyte mosses (*P. patens*, right, 41.7% group sequence similarity, ***J***_***X***_ = 0.072).** B)** The slow sequence changes in the regulator of chromosome condensation (RCC, RepeatsDB class 4.8, consensus left) protein with its 7-bladed propeller repeat structure maintains a fairly simple, regular pattern along with a more complex one closer to the C-terminus as demonstrated by proteins from the black cottonwood tree (*P. trichocarpa*, center) and the obligate marine actinomycete (*S. arenicola*, right) despite only 23.6% group sequence similarity (***J***_***X***_ = 0.053). **C)** A very complex dot plot pattern is evident among the DSCA proteins (RepeatsDB class 5.5, consensus left) in animals with examples given from the mammalian (*H. glaber,* center) and avian lineages (*C. anna*, right) with overall group 57.5% sequence similarity, ***J***_***X***_ = 0.118). **D)** Similarity among the vertebrate CDC23 (RepeatsDB class 3.3, consensus left) proteins is also high and the protein maintains a complex dot plot demonstrated in both the fish (*N. korthausae,* center) and duck (*A. platyrhynchos,* right) homologs (83.1% group sequence similarity, ***J***_***X***_ = 0.217). Larger versions of these panels are given as SI Fig. 9

The dot plots produced by DOTTER reveal complex patterns that can be used to compare repeat proteins much like traditional sequence alignment methods while also reducing the effect of sequence repetition [20]. Analysis of repeat protein amino acid sequences (Fig. 1) using DOTTER [31] readily revealed visually identifiable patterns for the proteins (Fig. 2 & SI Figs. 1, SI Table 1). Human observation noted that pairs of dot plots with a Jaccard similarity score (***J***_***X***_ ≥ 0.5; ***J***_***X***_ is the ratio of the number of matching black pixels in both dot plots to the total number of black pixels) were typically quite difficult to distinguish and pattern similarities were usually detectable by human observers at ***J***_***X***_ ≥ 0.1. Furthermore, known repeat containing proteins had more information rich dot plots on average than randomly selected proteins. Proteins within the RepeatsDB set had a mean of 272 pixels per protein chain (median 119 pixels/chain, mean length = 345 residues/chain, mean 0.66 ± 1.0 pixels/residue), where pixels were simply the black points within the dot plots corresponding to the comparison of a specific pair of amino acids related by the dot plot indices. This set of known repeat proteins had significantly more signal information in their dot plots than two control sets (“bacillus” and “mouse” generated by searching the PDB for both of these keyword terms) (Table 1, SI Fig. 2). Within the RepeatsDB set, 71.8% of proteins had more than 0.14 pixels/residue, with artificially designed repeat proteins (identified by searching within RepeatsDB for the term “design”) tending to have more pixel information than natural ones on average (SI Fig. 2d). Because the DOTTER-produced dot plots lack the explicit degeneracy that confounds traditional sequence comparisons and pairwise comparison of the plots was rapid and efficient it allowed us to analyze the entirety of the UniRef90 database [32]. To compensate for differences in protein sizes, we introduced a “sliding” method in which the start of the smaller protein was positioned along every possible point that gave any overlap along the self-identity diagonal of the larger protein (Fig. 1). The highest ***J***_***X***_ score was considered the optimal positioning. We identified 13.3 million (16.9%) protein chains (out of 78.9 million) with an information content of at least 0.42 pixels/residue. The 0.42 pixels/residue cutoff was chosen based on a comparison of the RepeatsDB set and the control “mouse” and “bacillus” sets (see Table 1, SI Fig. 2). This is within the range previously reported for previous estimates of the prevalence of repeat-containing proteins [16, 17]. Likewise, we reasonably find that 5.5% of proteins in the set contain one or more LCR regions when a minimum length = 20 filter is applied (and 23.3% for a minimum length = 6 filter) [4].

**Table 1:** Collected statistics from dot plot analyses. Mean values are given while median values are in parentheses. Histograms of these data are given as SI Fig. 2

Dataset	RepeatsDB	“bacillus” set	“mouse” set	“designed” set
Number of chains	6215	1325	985	233
pixel count (pixels/chain)	272 (119)	50 (25)	47 (20)	377 (204)
protein chain length (residues)	345	282	247	233
pixel density (pixels/residue)	0.66 +/- 1.0	0.14 +/- 0.15	0.14 +/- 0.22	1.62 +/- 1.62

### Conservation of dot plot patterns in related proteins

**Fig. 3:**
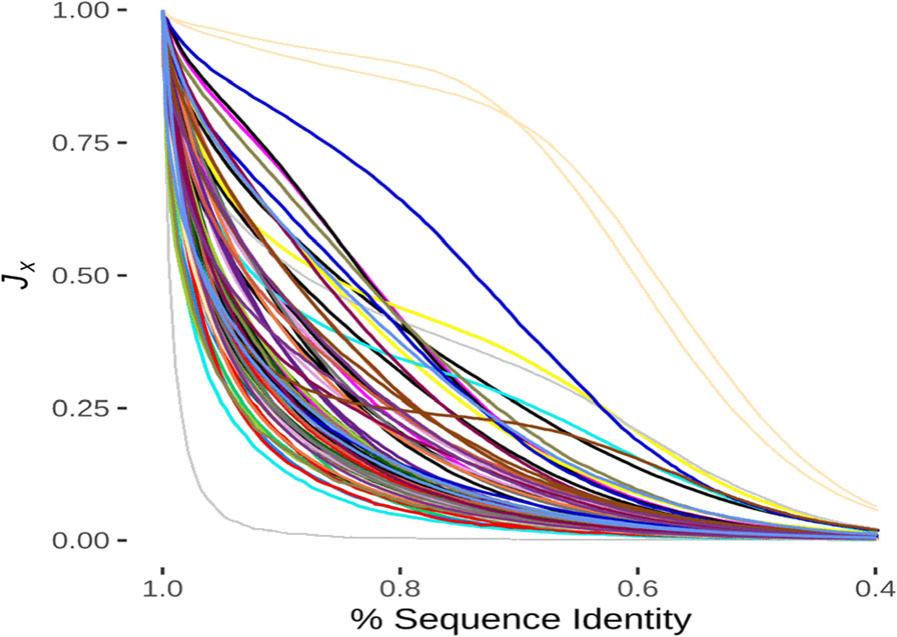
Decay of ***J***_***X***_ under random mutation. The set of standard proteins was subjected to repeated rounds of in silico mutation, then the average ***J***_***X***_ between the mutant and the initial was plotted. 64 of 79 protein chains (84%) demonstrated a simple exponential decay with an R_2_ ≥ 0.98 (see SI Fig. 3 for full figure key)

The patterns present in the dot plots of repeat proteins were maintained longer than should have been expected as compared to randomly changing sequences, suggesting that there is some pressure to maintain these patterns. In order to investigate how the dot plots were affected by changes in sequence, we estimated the rate of information decay by subjecting a set of 79 chains (the standard set, see Methods, SI Table 2) to random *in silico* mutations (Fig. 3, SI Fig. 3) using BLOSUM62 [33]. These 79 proteins (at least two from each of the RepeatsDB subcategories) were used as a standard test set throughout this work. Here, the standard set proteins were mutated *in silico* and the dot plots were calculated for the mutants to compare to the original protein, producing a decay curve for ***J***_***X***_ values. The resulting curves were fit to a simple exponential decay equation (***J***_***X***_ **= e**^**-bz**^) where z indicates the per cent identity difference between the mutant and initial proteins. Random mutation usually resulted in a 50% reduction in ***J***_***X***_ after an 8.2 ± 1.1% loss of sequence identity demonstrating that the patterns decay rapidly in the absence of selective pressures (8.5 ± 0.5 when calculated only from the chains (*N* = 64) with good R^2^ values for the decay experiment, SI Table 1). It should be noted that both these decay constants are within each other’s standard error ranges. In most (19 of 22, 86%) of the subgroups taken directly from RepeatsDB at least 2 out of 3 proteins (SI Table 1) tested exhibited single exponential decay as judged by an R^2^ ≥ 0.98 for the fit, and in 12 of the 22 subgroups (55%) all of the protein chains did so (SI Table 2). Since ***J***_***X***_ values seemed to be conserved better than sequence identity (decay half-life < 10% seq id), we hypothesized that it might be employed as a more robust method to detect evolutionary relationships than approaches that rely solely on sequence alignments.

Because the decay had a “half-life” of less than 10% sequence identity, we examined how well this method could detect commonalities in related proteins and compared it to standard phylogeny using MrBayes [34]. We chose 12 proteins from the standard set to attempt to identify conserved, consensus dot plot patterns that might be conserved among each set of these related proteins. Illustrative examples for 4 sets of closely related proteins are given in Fig. 2 (comparisons of phylogenic and dot plot analysis for all 12 sets are given in SI Fig. 1). Consensus dot plot patterns were identified for 10 of these 12 (83% success rate). We also used the standard set of 79 proteins (see Methods) to examine the effects of insertions on decay of the Jaccard score by randomly inserting amino acids into a protein sequence. Random insertions had a more debilitating effect on the dot plot conservation, with half of ***J***_***X***_ being lost on average after a 0.96% ± 0.37% insertion rate.

### Relationship between sequence and dot plot conservation

**Fig. 4:**
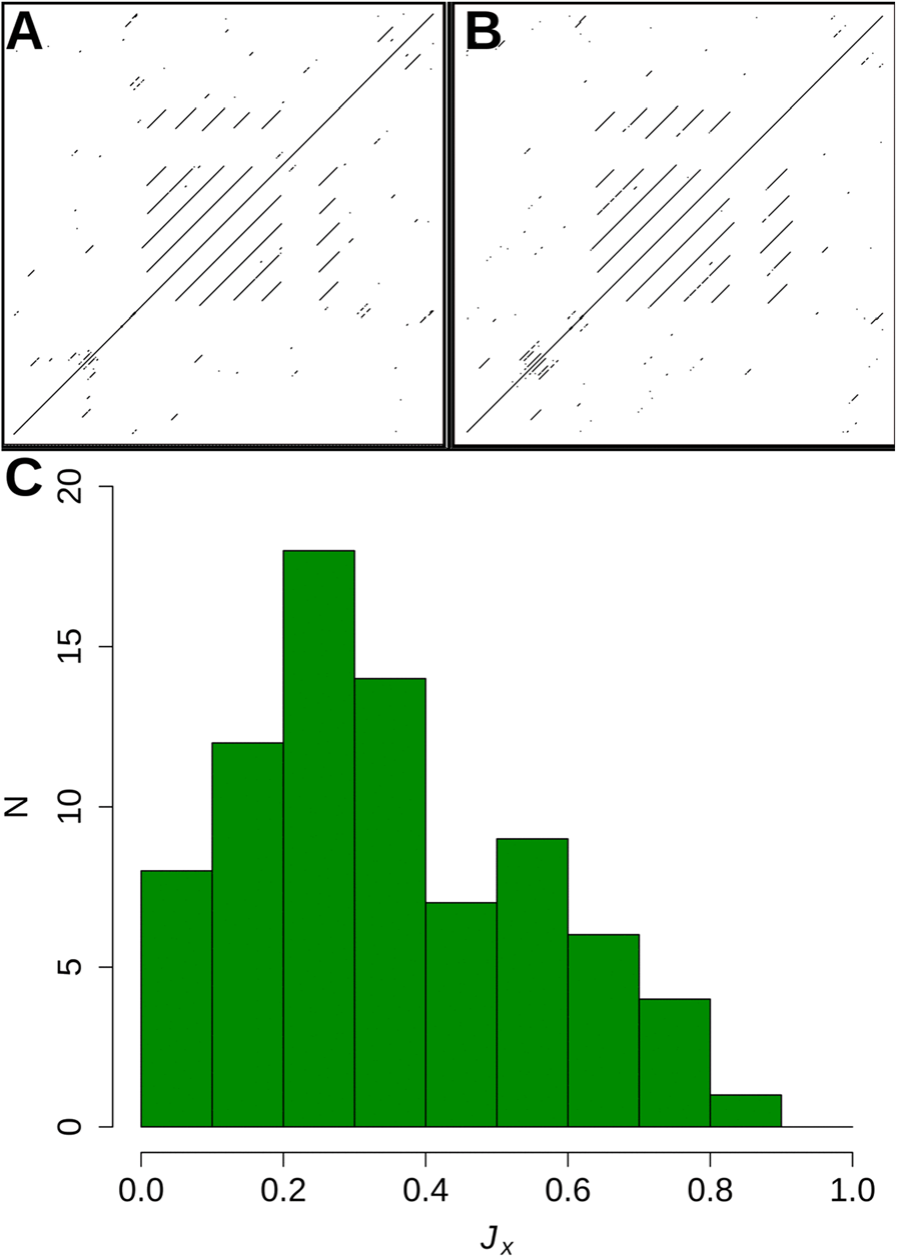
Permuted repeat protein sequences. Changing an entire protein sequence while maintaining the repeat pattern does not destroy the dot pattern. **a** dot plot of *P. marinus* kinesin light chain and **b**) the dot plot of its mutated (no sequence identity) analog. **c** Histogram of the distribution of the Jaccard similarity (*J*_*X*_) between the proteins of the standard set and their permuted analogs

We sought to investigate if the relationships between different dot plots were entirely due to sequence similarity. To do so the pairwise sequence identities for all the members of the full RepeatsDB [9] set were calculated and compared with their Jaccard distances (**J**_**D**_) (SI Fig. 4). This comparison showed two features, a main peak around 10–20% sequence identity comprising most of the pairwise comparisons between the proteins and a smaller one above 90% sequence identity which was highly enriched in streptavidin chains (*N* = 387) that have low information content plots (almost no positive pixels) but do make up a sizable portion (6%) of the total number of chains in the dataset. Additionally, the set of 79 standard proteins when mutated using a replacement matrix (see Methods) showed remarkable maintenance of the dot plot structures and ***J***_***X***_ values (Fig. 4, SI Fig. 5). Despite essentially no sequence identity between the protein and its mutated variant the dot plot patterns were often quite similar (as high as ***J***_***X***_ = 0.88 for GalNAc/Gal-specific lectin, see PDB ID 5f8w chain A in SI Fig. 5). In fact, 71 of the 79 (89.8%) test proteins had a ***J***_***X***_ ≥ 0.1 (our estimate for minimum ***J***_***X***_ that could be recognized by human observers) and 20 out of 79 (25.3%) had ***J***_***X***_ ≥ 0.5, the point at which it is typically difficult for human observers to distinguish two proteins, despite the two proteins having essentially no sequence identity in all cases.

### Analysis of large data sets with DOTTER

We sought to determine how efficiently we could analyze large protein data sets with our method. First, we utilized the RepeatsDB database [9] to produce a general analysis of known repeat proteins (SI Dendrogram). Generation of the DOTTER dot plots for the set of ~ 6000 protein chains obtained from RepeatsDB in batch mode required only a few minutes on a modern LINUX desktop computer. The protein chains from RepeatsDB were analyzed using pairwise distances (**1 –*J***_***X***_ **=** ***J***_***D***_) and then hierarchically clustered and the resulting clusters were scored based on how well they replicated the known sequence identity and structural subgroups defined in RepeatsDB. The clusters from the dendrogram were examined manually with special attention paid to clusters with a high average number of pixels per member (SI Table 3). We chose to examine the clustering generated by the McQuitty method in R because it gave the largest number of total clusters at a reasonable cut-off level and the clusters were the most homogenous with the sequence identity groupings and structural classifications used by RepeatsDB itself (SI Table 3, SI Dendrogram). We were unable to identify any correlation between these clusters and the structural groups as defined by RepeatsDB beyond what would be expected from sequence conservation. But, while most of the resulting groups were immediately obvious upon inspection, manual examination did find an intriguing clustering of the highly immunogenic OspA protein from the spiroform bacterium *B. burgdorferi*, the causative agent of Lyme disease [35] and the LIC proteins of unknown function from the pathogenic spiroform *Leptospira bacteria* [36] which cluster together despite not having significant group median sequence identity (42%). This relationship was also robust, occurring with several methods other than the reported McQuitty method (SI Table 3). We are unaware of this relationship having being noted elsewhere despite the not insignificant sequence identity these families share, although sequence similarity does not correlate well with the distance of evolutionary relationships in repeat proteins.

**Fig. 5:**
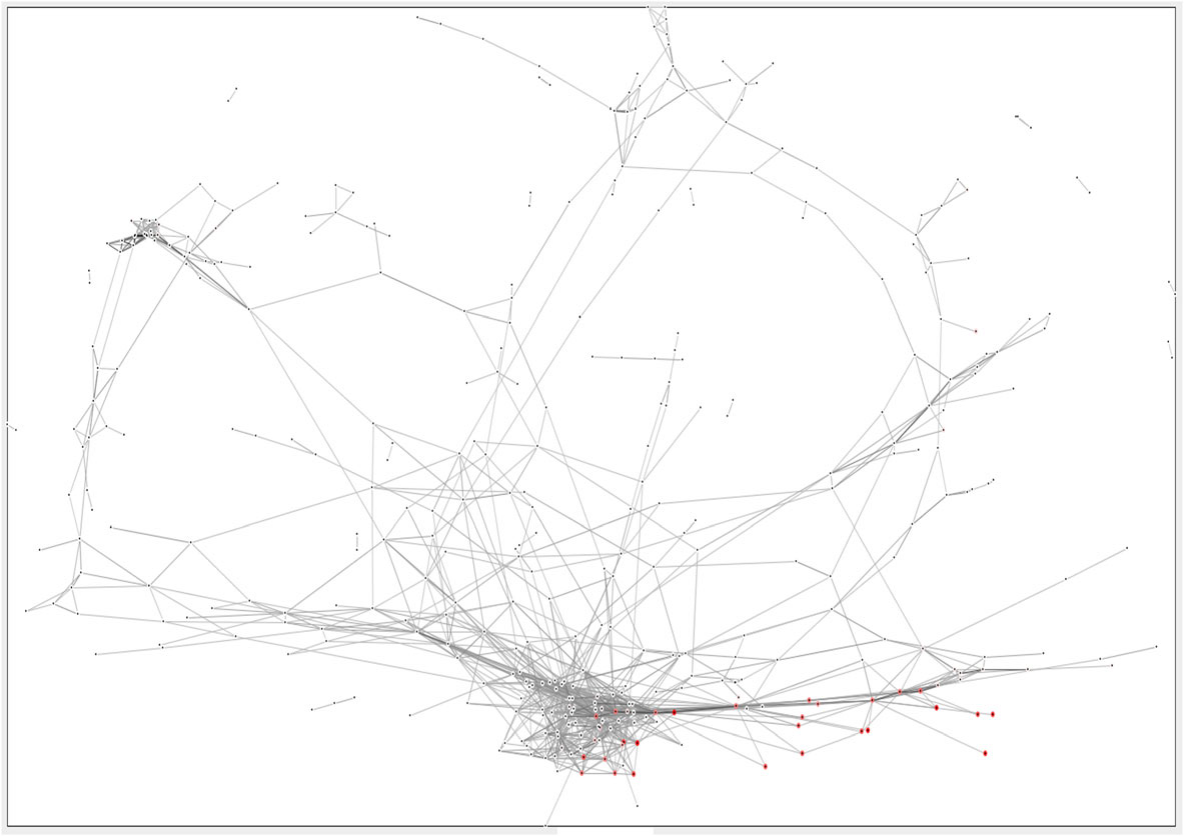
The CLANS plot of the clustering of repeat proteins discovered in UniRef90. Dot plots for every protein chain in UniRef90 (downloaded Sept 17, 2018, *N* = 78915455 chains) were calculated and those proteins with significant signal were collected (n_PROT_ = 13297656) and all possible pairwise Jaccard comparisons were made. These were then clustered using MCL and the medioid point was calculated for every cluster with 5 or more members (n_CLUST_ = 10205) and the inter-medoid distances were used to generate the CLANS figure. Clusters are colored according to the frequency of low complexity regions (LCR) with more intense red indicating the presence of a higher fraction of chains with one or more LCR. Notably, these LCR tend to cluster in the same region of the CLANS plot. This is a 2D representation of a 3D CLANS plot

Second, we applied the method to a large data set, namely the UniRef90 database which contains all known protein sequences at 90% sequence identity. This set was analyzed with DOTTER and HipMCL [37] was used to cluster all sequences that had corresponding dot plots with at least 0.42 pixels/residue of information. This gave 23050 clusters of which 10205 had at least 5 members. We arbitrarily classed clusters with 4 or fewer members as singletons. Manual examination of those clusters which had between 5 and 200 members (*n* = 8569) found that only 538 of the clusters were not comprised of a single functional type as judged by UniProt protein names while 925 clusters were made up of entirely or essentially entirely “uncharacterized” or “hypothetical” proteins. 7104 clusters (82.9%) were easily human identifiable as a single functional type (or 8031 (93.7%) if “uncharacterized” proteins are included as a functional group) (SI Fig. 6). The number of multi-function clusters increases sharply at the lowest 5% of median sequence similarity clusters (SI Fig. 7). Analysis of these 8559 clusters from UniRef90 revealed that they had between 31.8–99.9% median pairwise sequence similarity within a cluster as calculated by a global alignment in BioPython (BLOSUM62, gap opening = − 11, gap extension = − 1) [38] (SI Fig. 7). Calculation of the pairwise sequence similarity for 10 of the clusters failed due to either long sequence length or a high number of non-standard amino acids. The distance relationships for the set of clusters with 5 or more members were visualized by CLANS [39] (Fig. 5). Attempts at finding superclusters of related proteins from this CLANS representation were not particularly successful, however the clusters in which the greatest proportion of their members contained LCR did seem to group in one small region of the plot. A list of the proteins contained in the clusters is included in the supplemental material.

## Discussion

In this study we sought to establish a basic groundwork for the analysis of the informational relationships present in repeat proteins using DOTTER. We are not the first to use self-comparison methods like DOTTER to analyze repeats in individual proteins [40–42]. However we differ from these previous attempts in that we noted the conservation of the patterns within these self-comparisons. This implies that the informational relationships present in the repeats, which can be quite complex (Fig. 2), is more analogous to the “fold” of a protein [43] than it is to a matched set of sequence motifs, likely due to the extra information present in the sparse 2D matrix generated by DOTTER. That is, like protein structure, these patterns can robustly accommodate numerous sequences, although random changes without consideration to the informational relationships can be quickly detrimental to pattern conservation (Fig. 3). This is not without precedent as a study of 28980 protein chains in 506 SCOP folds found that the relationship between sequence and structure was ambiguous and that structural motifs should not be correlated with particular sequences [2]. Upon recognizing this, we utilized a simple model (Jaccard) to estimate the evolutionary distance between pairs of proteins accounting for size differences with a straightforward sliding method to find optimized overlaps and attempt to compensate for insertions and deletions. By optimizing the efficiency of these calculations we were able to identify all the likely repeat proteins in the known protein universe (UniRef90) and cluster them into a relatively small number of clusters requiring about 3.2 × 10^5^ CPU hours (Fig. 5). While other investigators have used self-comparison methods like we did, more recent efforts tend to favor sequence statistical analysis approaches [44–47]. This preference may be partially due to historical attempts to define repeats by the length of the repeating sequence. The identification of 34 residue long TPR [21], the 35 residue long PPR [48], and the 38 residue long OPR [49] two helix repeats as well as the identification of 42 residue long TPR repeats [23] fit this historical tendency. Our method obviates this issue by being agnostic to repeat length (as do others [17]) as it can readily analyze short LCR type repetitions (Fig. 5) as well as the longer domain length repeats such as those in RepeatsDB [9] class 5 (Fig. 2). Furthermore, our model calculations show that simple mutation of less than 10% of a protein sequence or insertion of an additional 1% of the protein sequence will reduce the ***J***_***X***_ score of a protein and such a mutant by 50%. It intrigues us that related proteins which have undergone much greater changes than this often maintain more significantly similar dot plot patterns suggesting that these patterns are reporting on the parts of the protein which are under evolutionary pressure to be maintained.

The efficiency of this method then allowed an attempted analysis of several other aspects of the behavior of repeat proteins. While our estimate of the frequency of repeat proteins in the UniRef90 database is within the previously identified ranges [16, 17], we use a simple information content metric (0.42 pixels/residue) based on structures available in the PDB to make this determination which can easily be adjusted to change the prevalence should a reliable consensus frequency emerge. We were also able to analyze LCR in proteins as well as longer “full” repeats. Significant conservation in amino acid tandem repeats (a type of LCR) was observed in a set 3094 human/mouse protein pairs in agreement with our observation that LCR are largely localized to one region in our clustering of the repeat proteins (Fig. 5) [50]. Perhaps the most intriguing though is the potential to detect possible relationships between repeat proteins that may be obscured by the apparent simplicity of both the sequence and structure of many repeat proteins. For example, all the robust clustering of the OspA protein from *B. burgdorferi* [51] and the LIC proteins of unknown function from *Leptospira* [36]. But this possible relationship is still experimentally unconfirmed.

## Conclusions

The amino acid sequences of repeat proteins maintain an unusual sort of sequence conservation in which there appears to be both maintenance over a long evolutionary period [19, 27] while also being much more forgiving of amino acid substitutions than standard proteins [22]. The interesting question then concerns what kind of information is actually being conserved in repeat proteins and how can this be identified? By recognizing the tautological fact that protein repeats must repeat another part of the protein and mapping the resulting network of relations we can begin to understand what parts of the protein architecture are structurally or functionally important and therefore must be robust to stochastic sequence drift. Identification of these patterns can help to cluster related repeat proteins, discover parts of the protein that are essential for structure and function, and identify relationships between repeat proteins that may be remarkably difficult to analyze by purely sequence-based analysis [52].

Identifying the conservation of these informational relationships is of course the beginning of a line of inquiry and much work and many questions remain unresolved. The most obvious is that we have employed a rather simple sliding method to optimize the apparent matching between two proteins of different sizes (i.e. insertions and deletions). This does not account for large internal insertions which may split the pattern in half, nor for smaller insertions or deletions which would change the spacing between equivalent lines and are likely to efficiently reduce ***J***_***X***_ scores (Fig. 1). Likely an efficient method to divide the patterns into smaller units would improve our analysis. In addition, improvements to cluster analysis are needed. While we were able to efficiently find clusters that contained proteins of a single function (using slow, manual analysis), finding crowded areas comprised of a large number of clusters containing thousands of proteins was frustratingly ineffective as proximal clumps of several clusters in the CLANS plot (Fig. 5) only sometimes seemed to be enriched in single functionalities. Likewise, the clustering of LCR enriched clusters (Fig. 5) may either indicate an actual commonality or be simply a mathematical artifact of the methodology. Additionally, we did not discover any correlation between protein structure and clustering of repeats that would not be obvious from a direct comparison of sequence identity conservation (SI Dendrogram). Furthermore, our basic assumption that “protein repeats must repeat” may not always hold. For example, proteins with a single copy of a repeating unit within a chain would be missed by our method (e.g. a single copy of an ancient β-propeller) [22, 53]. We also do not know exactly what factors give rise to these informational networks or why they are conserved, but we expect that all of the possible explanations (structural or functional constraints, sequence inertia in recently diverged proteins, etc.) may occur singly or in combination in some sub-set of repeat proteins. And we are aware that these patterns can change with a frustrating arbitrariness; the pattern in the RCC proteins is maintained among the eukaryotic lineage despite a low sequence conservation, while there appears to be a different pattern for the sauropsid and synapsid vertebrate lineages for fibrinogen (SI Fig. 1Q, R). And lastly, many of the repeat clusters are comprised of membrane rather than repeat proteins, although the similarity between these two general classes of protein has been noted before [16, 54]. This may indicate a deficiency in our method or it may indicate a shared set of physical constraints in membrane and repeat proteins due to the hydrophobicity of membranes and protein interiors. The question of a commonality between these kinds of proteins is clearly beyond the scope of this manuscript.

Selective pressure on proteins is often quite intense and the recognition of what properties emerge from this pressure often goes a long way to understanding the behavior and function of a protein. Using these simple comparisons we were able to quickly analyze the entirety of known protein sequences in UniRef90 and generate clusters of which 93.7% were clusters of a single or uncharacterized function. These patterns can be quite robust to sequence changes as many of these functional clusters had as low as 31.8% median sequence similarity within the cluster. We were also able to maintain good facsimiles (***J***_***X***_ = 0.88) of the dot plot patterns with artificially generated non-identity mutants although random in silico mutation usually lead to a 50% reduction in ***J***_***X***_ after an 8.2% loss of sequence identity. The recognition of the conservation of the informational relationship between repeats within a protein should help to further study, understand, and design repeat and LCR proteins.

## Methods

Unless otherwise noted, calculations were performed with custom code written in R, FORTRAN, Python, or C++. A software container for the sliding pipeline is available for download by non-commercial users at https://gorna.uw.edu.pl/en/research/software.

### Description of the sliding method

Sequences in FASTA format were subjected to self-analysis by DOTTER [31] in batch mode with a zoom level of 1 with black and white point values of 30 and 31 respectively, to generate .pdf and .ascii files. Scores of 31 or greater therefore defined the pixels in the dot plots. Dot plots were converted into binary format at the black/white level and pairs of plots were compared by calculating a Jaccard index (***J***_***X***_). The method reflects a procedure (Fig. 1) during which dot plots are aligned with respect to their main diagonal and shifted along it to compensate for differences in sizes between the two proteins, including size differences due to insertions and deletions, producing a Jaccard index for every shift. The highest value from the sequence was stored as a result and used in the next step and the maximal ***J***_***X***_ obtained was taken to be the closest relationship between the two sequences. During the analyses, the diagonal was ignored as it represents the trivial self-matches. Scanning the indices of each residue in the dot plots allowed the deconvolution of the plots back into repeats (Fig. 1).

### Random decay of dot plot signals (Fig. 3, SI Fig. 3, SI Table 1 & SI Table 2)

A set of 79 repeat-containing proteins was generated from RepeatsDB 2.0 [9] with at least 3 sequences that differed from each other at the 40% ID level for each repeat subgroup seeded with a few additional sequences of interest, each with > 100 residues length (average length = 423); however only two sets were available from RepeatsDB subgroup 4–7 and none from RepeatsDB subgroup 2–1. For each sequence, a dot plot was generated for the initial sequence. One residue was chosen at random and mutated according to probabilities based on BLOSUM62 and a dot plot was generated for the mutant sequence. ***J***_***X***_ was then calculated to compare the mutant and the initial sequences. This was repeated until the sequence had been subjected to *n* rounds of mutation, where *n* was the length of the sequence. The entire process from the initial sequence was repeated 1000 more times and the average ***J***_***X***_ and average sequence identity was calculated at each step. In order to investigate the influence of insertions on the value of ***J***_***X***_, the following procedure was applied using the standard set of 79 protein sequences. For each sequence, amino-acid insertions were gradually introduced up to a number equal to 20% of its length. At every step the Jaccard index was computed and stored. The entire procedure was repeated 100 times.

### Effect of sequence mutation on dot plot patterns (SI Fig. 5)

To test whether analogous sequences showing little similarity can at the same time yield similar dot plots, we used the amino-acid alphabet “shuffling” procedure. For each of the test set sequences, we generated an artificial analogous counterpart using a replacement matrix defining how the amino-acid alphabet will be changed. Such a matrix can be seen as a dictionary in which each amino-acid type (key) is unambiguously associated with another amino-acid (value) to which it will be replaced. The replacement matrix was independently optimized for each test sequence to generate the analogous counterpart with possibly low similarity to the original sequence. The optimization procedure involved the following steps: (i) Generation of a random 20-element replacement dictionary **V**_**A**_ (ii) “Mutation” of the replacement dictionary **V**_**A**_ into **V**_**M**_ by exchanging two randomly selected keys. (iii) Transformation of the input sequence **S**_**A**_ into a mutated sequence **S**_**M**_ using the substitution dictionary **V**_**M**_. (iv) Calculation of the similarity score between the mutated sequence **S**_**P**_ and the original sequence **S**_**A**_ using the BLOSUM62 matrix [33]. Steps ii to iv were repeated 10000 times using a Monte Carlo procedure. A “mutation” in the substitution dictionary was accepted (**V**_**M**_ stored as new **V**_**A**_) if it decreased the similarity score or if the Monte Carlo (MC) acceptance criterion (kT = 0.04) was fulfilled. In addition, the whole procedure (steps i to iv) was repeated 10 times to ensure better sampling. The application of the MC procedure enabled the finding of dictionaries that generated sequences with low similarity to the original sequences.

### Comparison of dot plots from all repeat proteins of known structure (SI Dendrogram)

The entire UniRef90 set of protein sequences (*n* = 6315 chains) was downloaded on Nov 24th, 2017 and subjected to dot plot analysis as follows. The 78915455 sequences were subjected to DOTTER as above and filtered to only include sequences with a length greater or equal to 121 residues and that had a dot plot with at least 0.42 pixels per residue (considering the top half triangle and not the self-identity line). This gave a set of 13297656 chains for further analysis. Plots were converted to binary as before and pairwise comparisons were calculated for all members. The large comparison matrix was sparsified by selecting 1400 largest values for each dot plot and was clustered using HipMCL [37]. For sequence selection procedure, we modified DOTTER which is a part of the Seqtools suite [55] in order to yield pixel per residue ratios and a binary file containing pixel data. A custom Python script using SCOOP library [56] handled parallel execution of DOTTER on an HPC cluster and produced the metadata required by the pipeline. Both sliding and sparsification procedures were performed using self-written C++ code. For each cluster, the medoid, the point which is the least different as measured by all pairwise ***J***_***X***_ values, was identified and a representation of the clusters was generated in CLANS [39] using cluster medoids as representatives (Fig. 5). The full list of clusters and protein IDs is provided in the SI.

A similar process was also undertaken using the protein chains in the RepeatsDB set, however the distances were clustered in R using HClust [57] and manipulated using the dendextend package [58]. The full set of repeat proteins from RepeatsDB was filtered in R with the protcheck function from the protr package [59] and then the remaining 6280 chains were aligned with the pairwiseAlignment function in the Biostrings package in R [60] to compare pairwise sequence identities between all the protein chains (SI Fig. 4). The results were rounded to the nearest per cent and binned and a heat map of the results was generated using the heatmap.2 function of Gplots in R [61]. Dendrograms were cut evenly at 50 heights and the clusters were then compared to the groupings (using both the structural subgroups and sequence identity) from RepeatsDB as well as examining the ***J***_***D***_ values between group members. The expected amount of information in repeat protein dot plots was determined by counting the number of positive pixels not on the diagonal from the protein chains in the RepeatsDB set. An estimate of the background information that might be expected to occur in non-repeat proteins was measured by creating two control sets comprised of 1325 “bacillus” or 985 “mouse” protein chains from the PDB [62]. Sets were generated by a search of the PDB for proteins with the text keyword “bacillus” or “mouse” with resolution between 0.0 and 2.0 Å and with matches trimmed at ≤30% sequence identity. These were downloaded as FASTA sequences and then chains present in RepeatsDB or those less than 101 residues length were removed.

### Identification of consensus dot plot patterns (SI Fig. 1)

Visual examination is often sufficient to identify a consensus pattern within a group of closely related proteins. Sets of example proteins were generated by selecting proteins from the 79 member test set and then finding related proteins using BLASTp [63], while the FASTKD1 and plant RAP containing protein sets were found using Pfam [64] . A multiple sequence alignment of the related proteins was generated by MUSCLE [65]. Alignment positions in which 20% or more of the sequences had a “gap” position were then trimmed. A set of 25 blank gap spaces (due to the size of the standard DOTTER scoring window [31]) was added to the N and C termini of the aligned sequences to reduce edge effects of the editing and the edited, identical length sequences were re-analyzed by DOTTER as detailed in general methods. The optimal method for hierarchical clustering was performed as with the RepeatsDB set and clustering methods and dendrogram cut levels were scored by looking for clusters in which members shared close relations (***J***_***D***_ ≤ 0.875). The average score at each position in the matrix was calculated and the resulting average DOTTER matrix was converted to a binary format to produce consensus dot plots. Images of consensus dot plots were generated using the heatmap.2 function of gplots in R [61] . Relatedness between the sequences was also confirmed by phylogenetic analysis using MrBayes [34] . During the runs the substitution model was optimized and runs were continued until the standard deviation of split frequencies was < 0.01.

Deconvolution of the consensus dot plots was also used to predict the location of the repeats within the proteins. A sequence position that had a consensus score of at least the cutoff value (multiples of 10 up to 50) anywhere in its associated row or column in the dot plot was considered to be part of the repeat which were readily detectable in the deconvoluted histograms (Fig. 1).

## Supplementary information

**Additional file 1.**

**Additional file 2.**

## Data Availability

Purpose written code for this analysis (a docker container and corresponding source code) is available for non-commercial users to download at https://gorna.uw.edu.pl/en/research/software. Supporting information is available online. Data used in this analysis is available from the online databases UniProt (https://www.uniprot.org/), RepeatsDB (http://repeatsdb.bio.unipd.it/) & the Protein Data Bank (https://www.rcsb.org/). A list of the clusters and the proteins included in each are included as part of the supplemental material.
