# Supplementary material for: Self-analysis of repeat proteins reveals evolutionarily conserved patterns
Source: BMC Bioinformatics. 2020 May 7;21:179. doi: 10.1186/s12859-020-3493-y (PMC7204011; doi:10.1186/s12859-020-3493-y)
Supplement: Supplementary file 1 — Additional file 1. [file 12859_2020_3493_MOESM1_ESM.pdf]

Supporting Information for Self-Analysis of Repeat Proteins Reveals Evolutionarily Conserved Patterns

Matthew Merski[1,*,†], Krzysztof Młynarczyk[1,*], Jan Ludwiczak[2,3], Jakub Skrzeczkowski[1], Stanisław Dunin-Horkawicz[2] and Maria W. Górna [1,†]

[1]Structural Biology Group, Biological and Chemical Research Centre, Department of Chemistry, University of Warsaw, Warsaw, Poland, [2]Laboratory of Structural Bioinformatics, Centre of New Technologies, University of Warsaw, Warsaw, Poland, [3]Laboratory of Bioinformatics, Nencki Institute of Experimental Biology, Warsaw, Poland

* these authors contributed equally to this work

† to whom correspondence should be addressed: merski@gmail.com, mgorna@chem.uw.edu.pl

SI Fig. 1A: Consensus DOTTER fingerprint of CC3 from the consensus of the mammalian proteins using the fingerprint method (SI Fig. 1C) . Black pixels indicate a DOTTER score ≥ 31 while yellow pixels indicate a DOTTER score between 31 and 10 and self to self identity is indicated by the central diagonal line. The proteins included in this analysis were 1) CC3, 2) CDC23, 3) DSCA, 4) vertebrate FASTKD1, 5) RNA-binding domain abundant in Apicomplexans (RAP), 6) fibrinogen 7) intestinal fatty acid binding protein (IFABP), 8) Interferon-induced protein with tetratricopeptide repeats 3 & 5 (IFIT3 & IFIT5), 9) proliferating cell nuclear antigen (PCNA), 10) Regulator of chromosome condensation (RCC), 11) S-phase kinase-associated protein 2 (SKP2), 12) Spindly

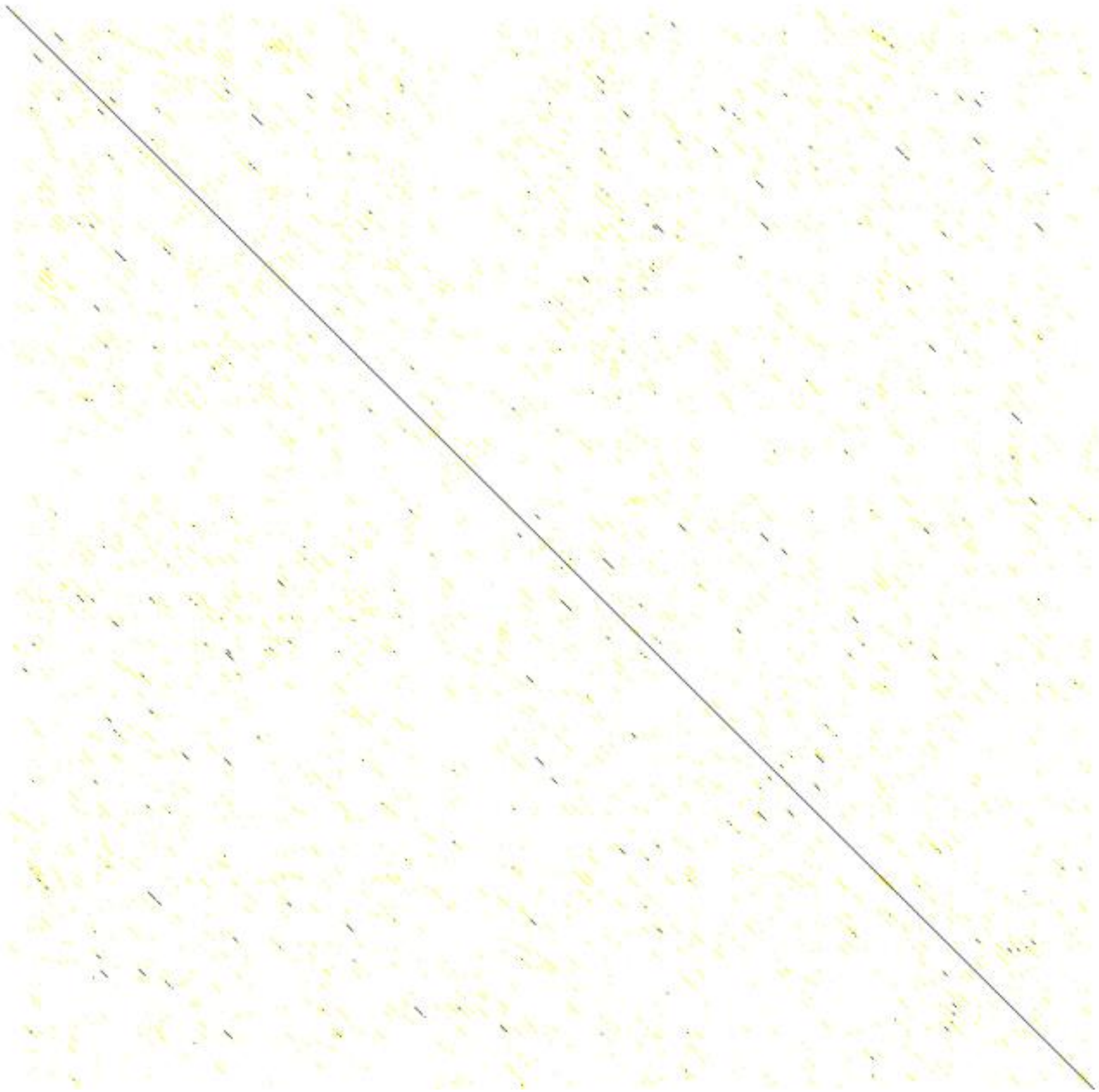

SI Fig. 1B: Phylogenetic tree of CC3 proteins made using MrBayes. The mammalian proteins used to build the consensus DOTTER fingerprint are indicated with red text.

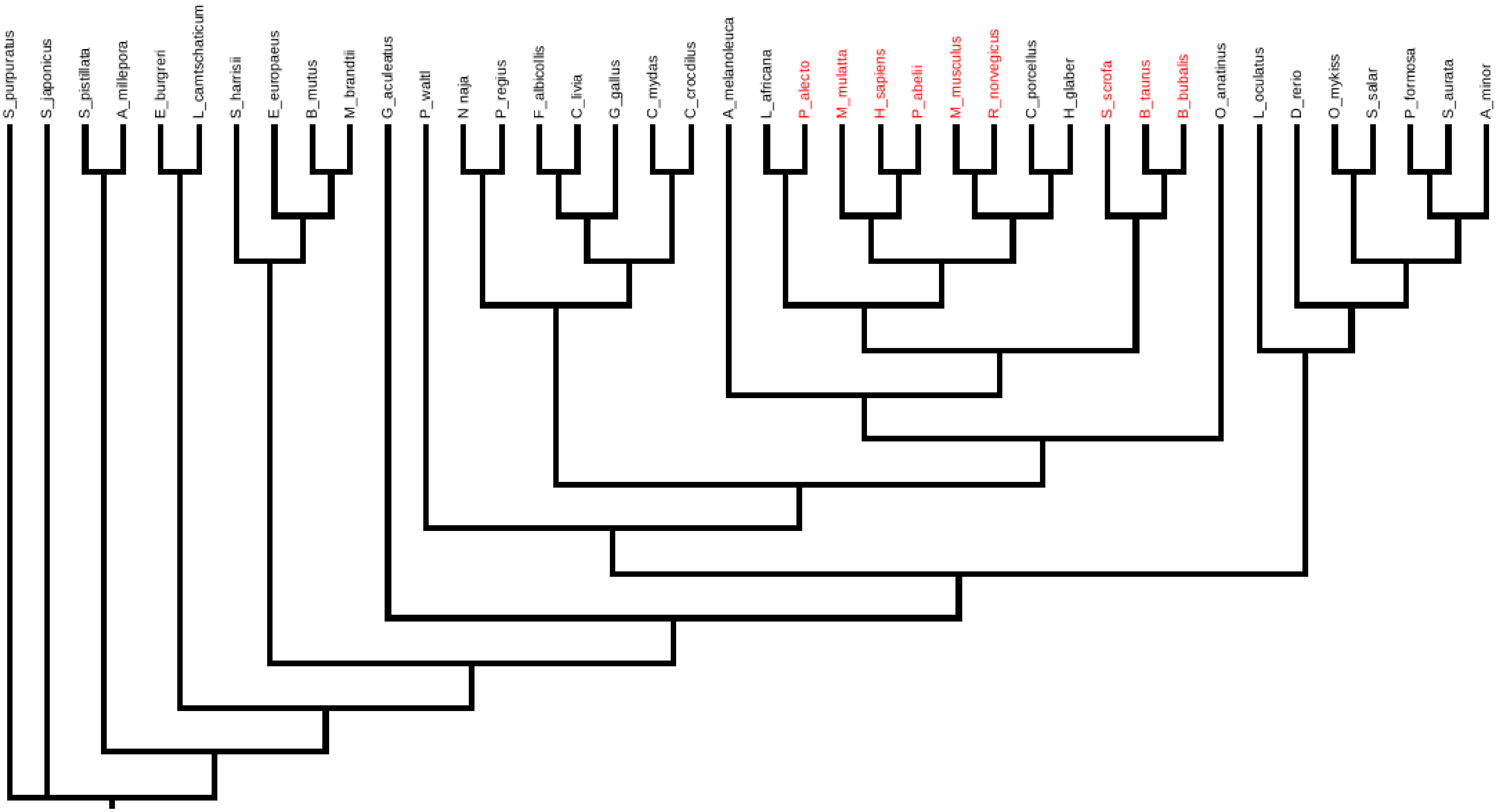

SI Fig. 1C: Complete linkage hierarchical clustering of DOTTER fingerprints for the set of CC3 proteins. The mammalian proteins used to make the consensus fingerprint are indicated by the red box.

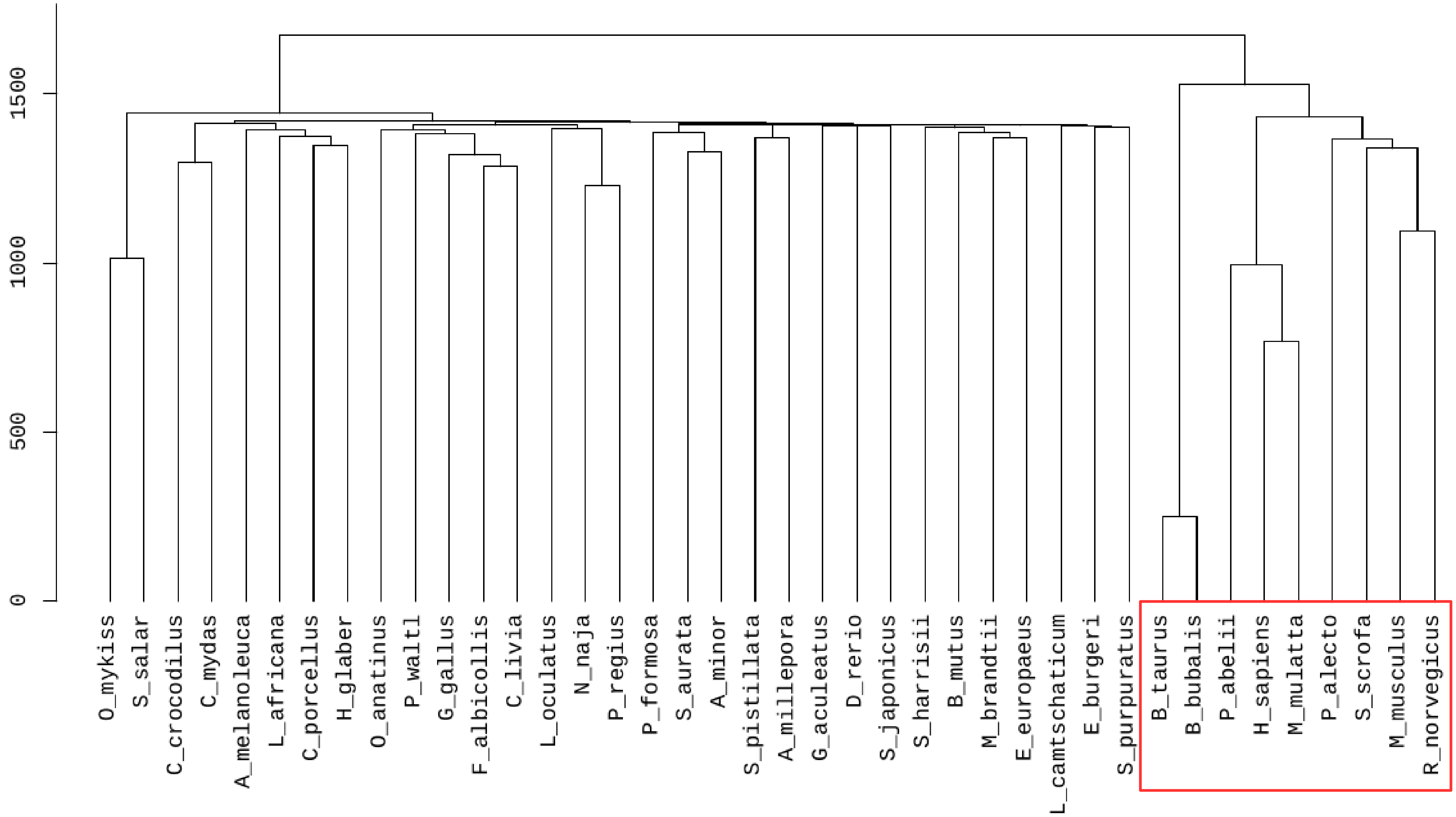

SI Fig. 1D: Consensus DOTTER fingerprint of CDC23 from the consensus of the vertebrate proteins using the fingerprint method (SI Fig. 1F) . Black pixels indicate a DOTTER score ≥ 31 while yellow pixels indicate a DOTTER score between 31 and 10 and self to self identity is indicated by the central diagonal line.

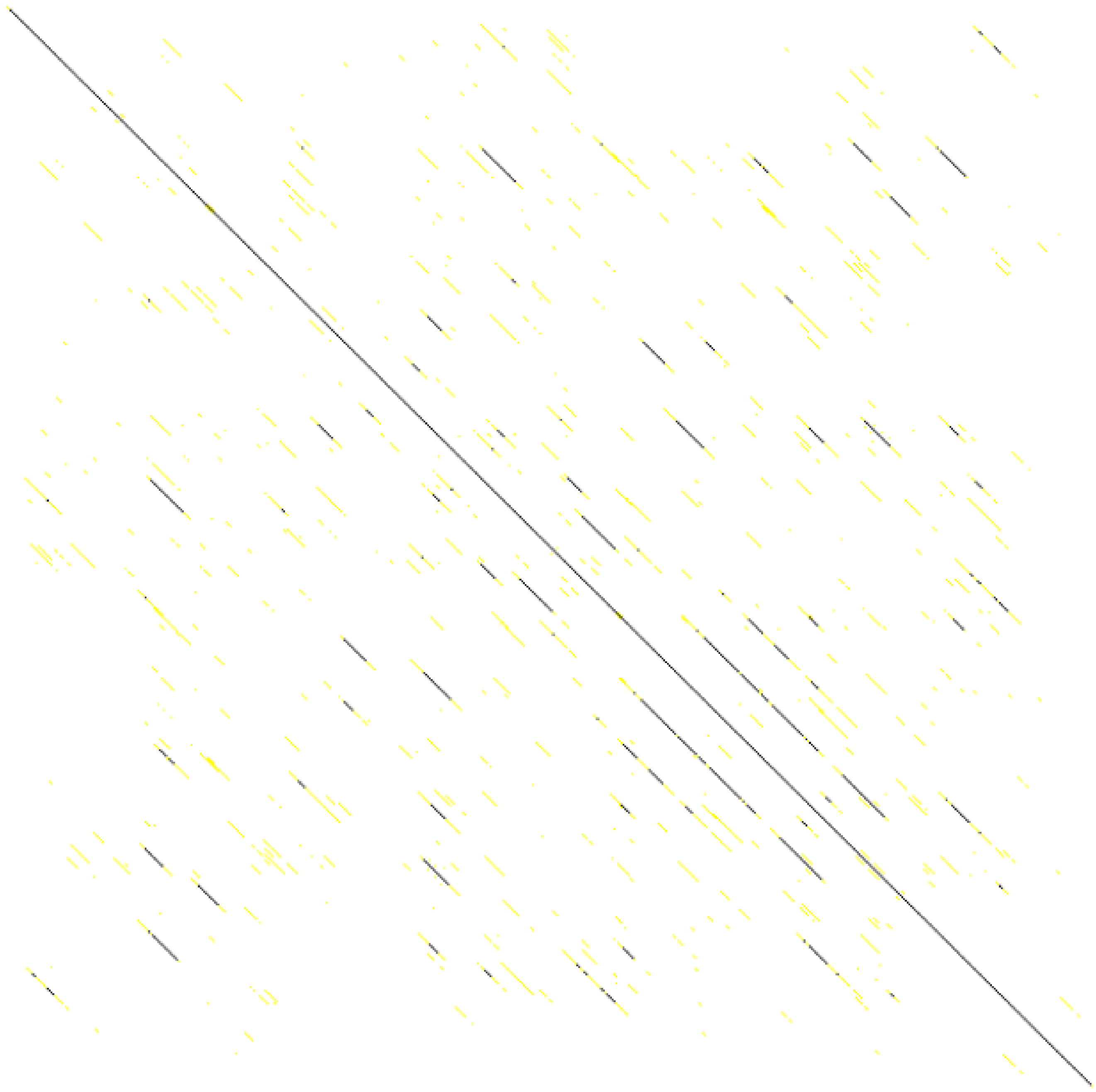

SI Fig. 1E: Phylogenetic tree of CDC23 proteins made using MrBayes. The vertebrate proteins used to build the consensus DOTTER fingerprint are indicated with red text.

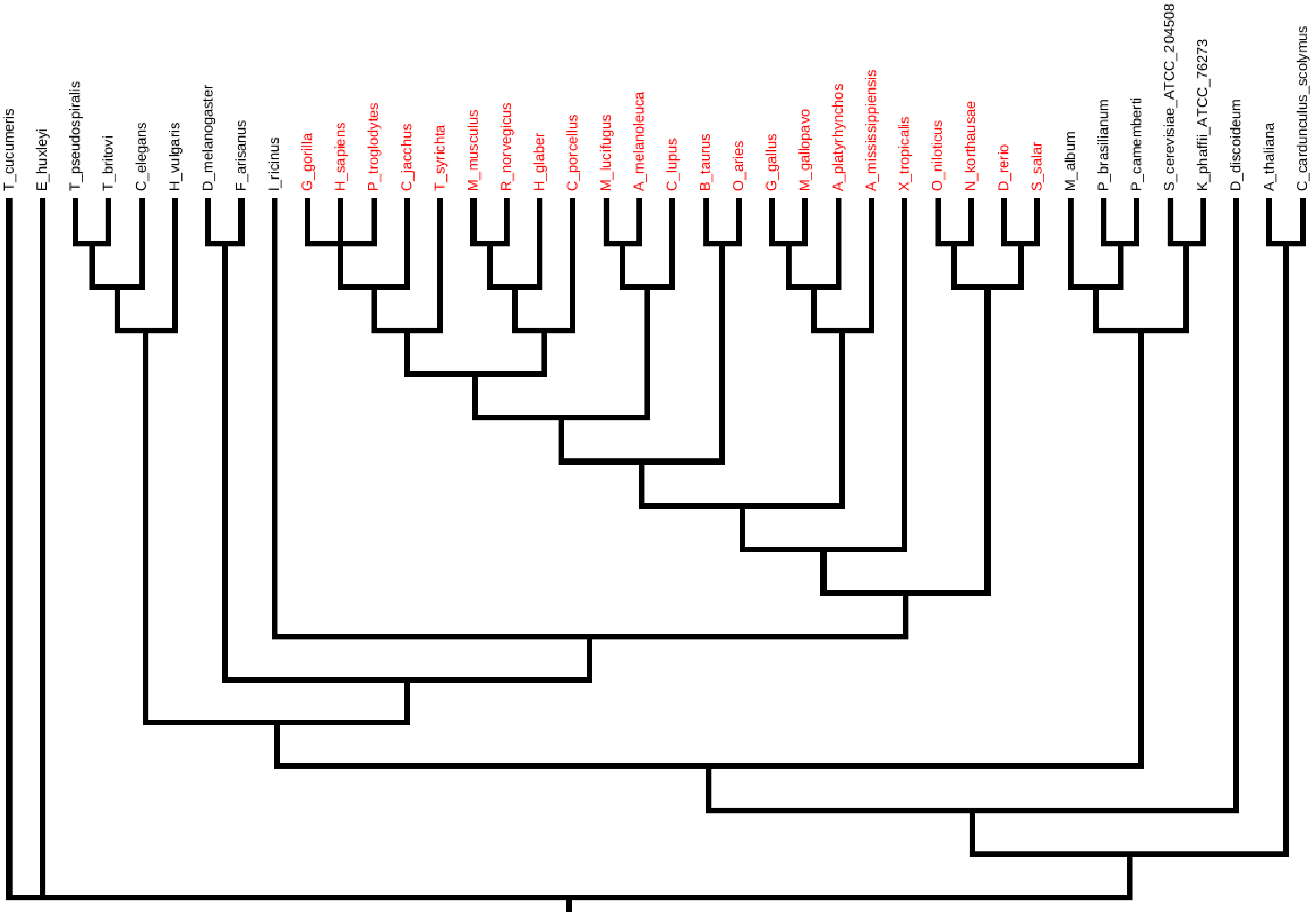

SI Fig. 1F: Single linkage hierarchical clustering of DOTTER fingerprints for the set of CDC23 proteins. The vertebrate proteins used to make the consensus fingerprint are indicated by the red box.

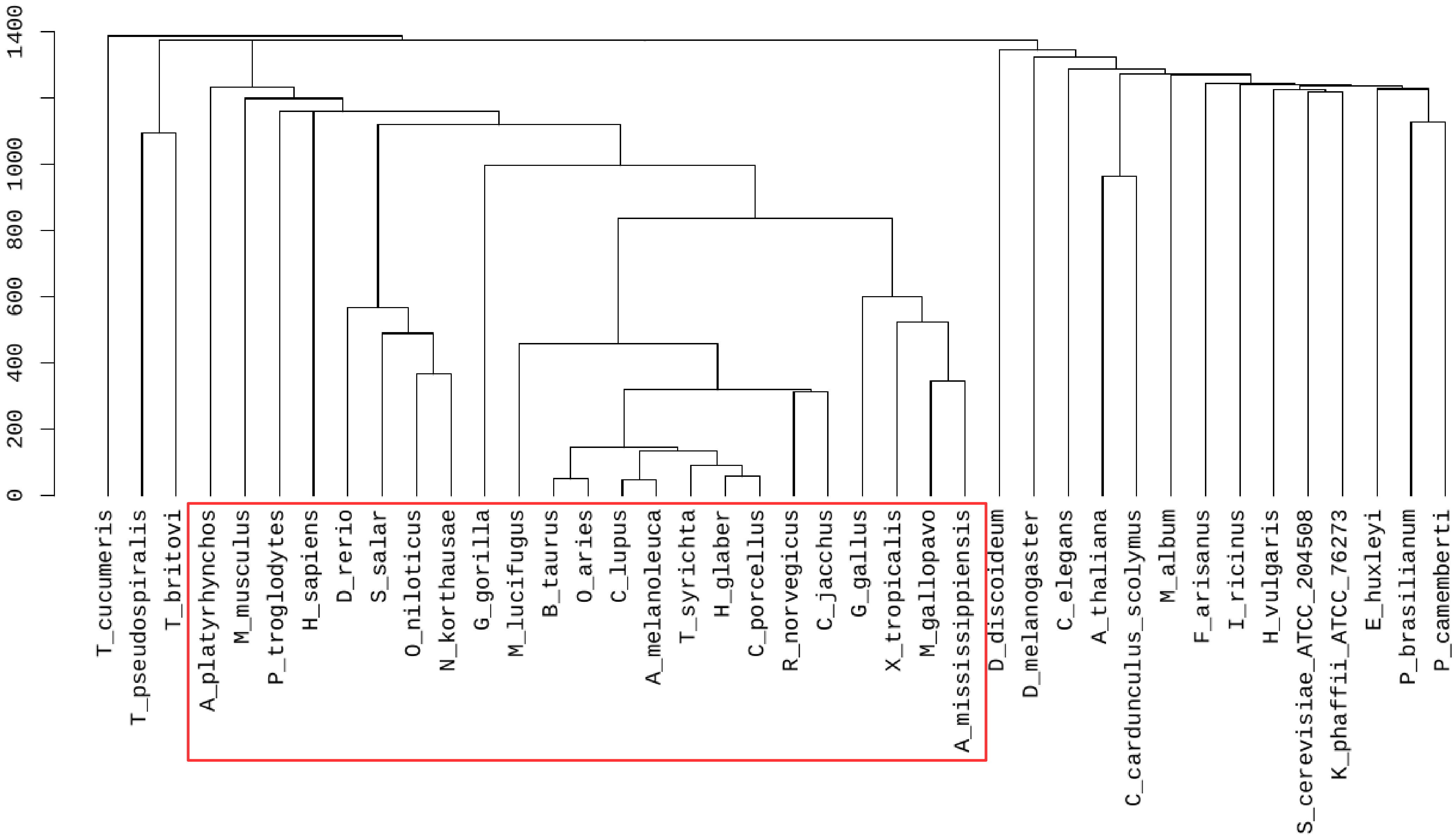

SI Fig. 1G: Consensus DOTTER fingerprint of DSCA from all the proteins in the set using either method (SI Figs. 1H & 1I) . Black pixels indicate a DOTTER score ≥ 31 while yellow pixels indicate a DOTTER score between 31 and 10 and self to self identity is indicated by the central diagonal line.

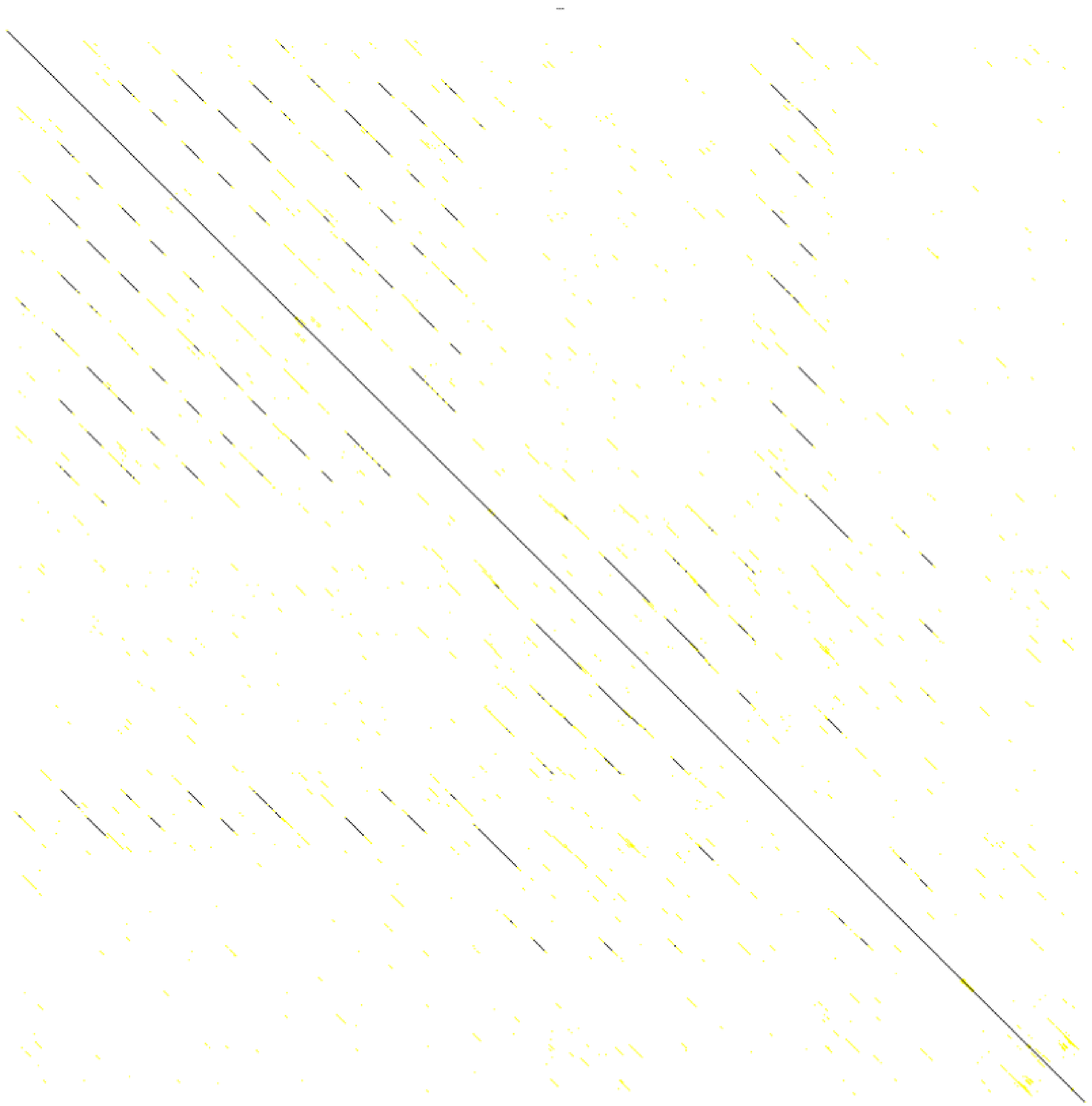

SI Fig. 1H: Phylogenetic tree of DSCA proteins made using MrBayes. The proteins used to build the consensus DOTTER fingerprint are indicated with red text.

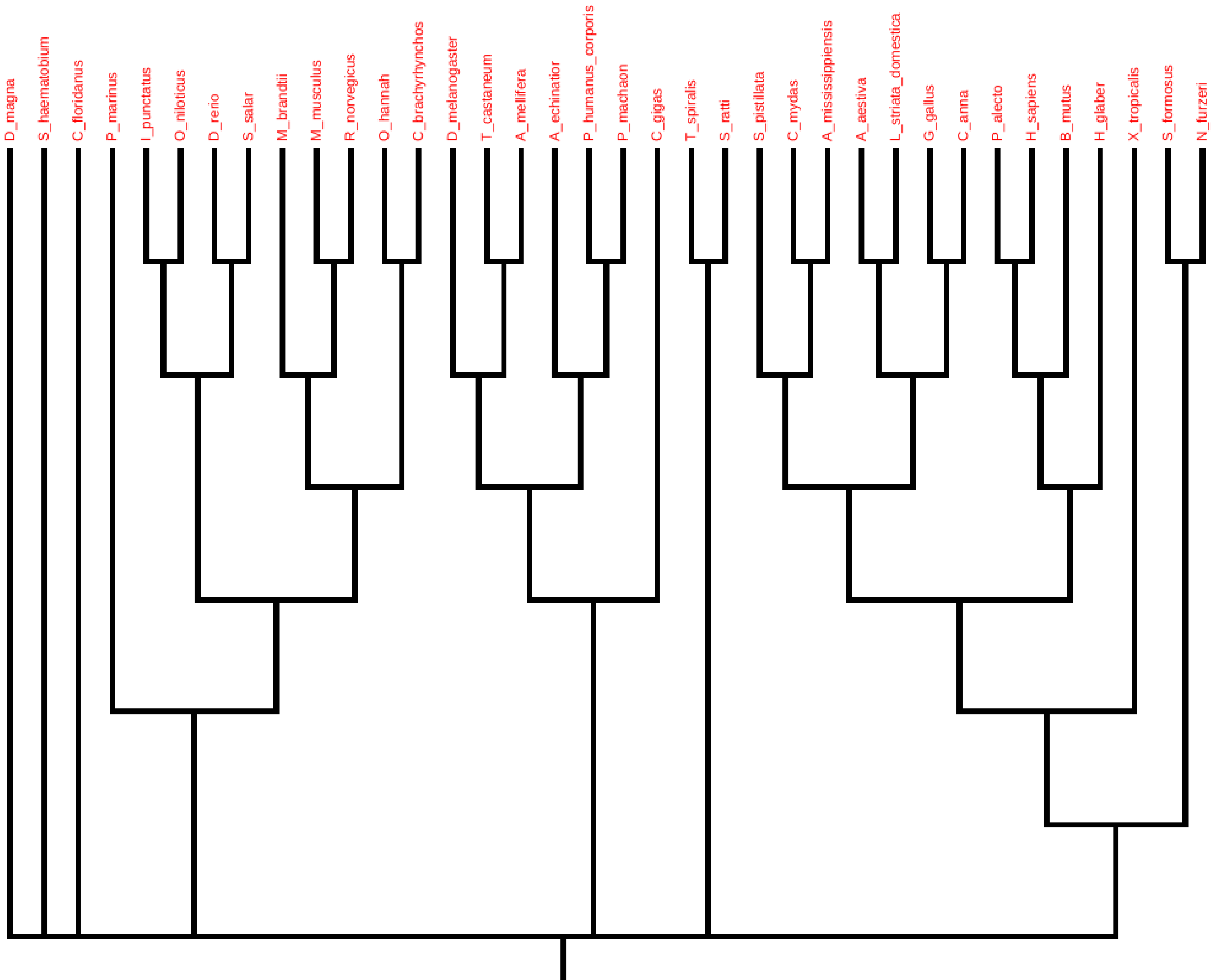

SI Fig. 1I: Centroid linkage hierarchical clustering of DOTTER fingerprints for the set of DSCA proteins. The proteins used to make the consensus fingerprint are indicated by the red box. Optimal clustering appeared to include all the proteins in this set, suggesting a rather strong conservation of the signal.

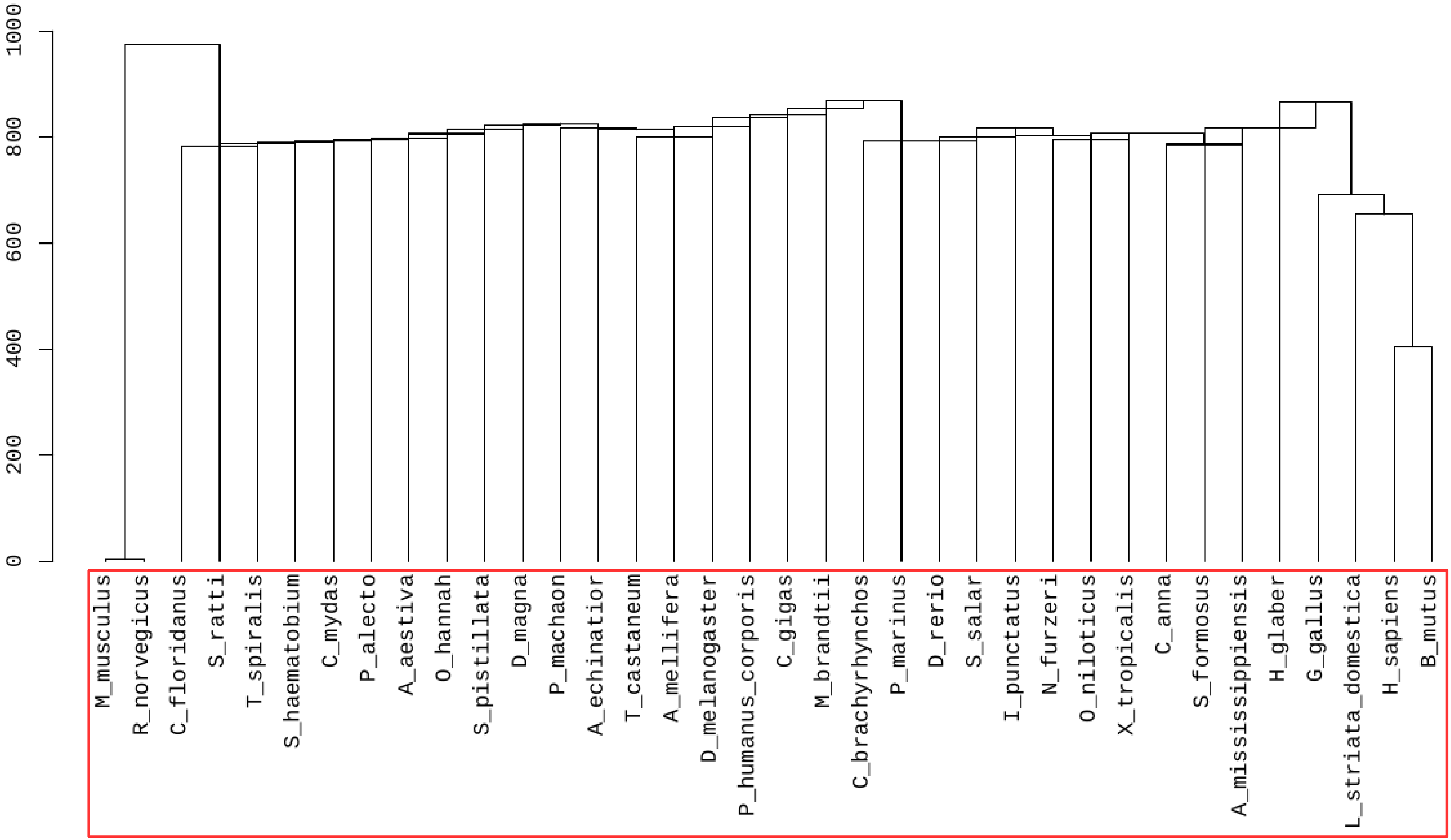

SI Fig. 1J: Consensus DOTTER fingerprint of FASTKD1 from the consensus of the fish & amphibian proteins using phylogeny (SI Fig. 1M) . Black pixels indicate a DOTTER score ≥ 31 while yellow pixels indicate a DOTTER score between 31 and 10 and self to self identity is indicated by the central diagonal line.

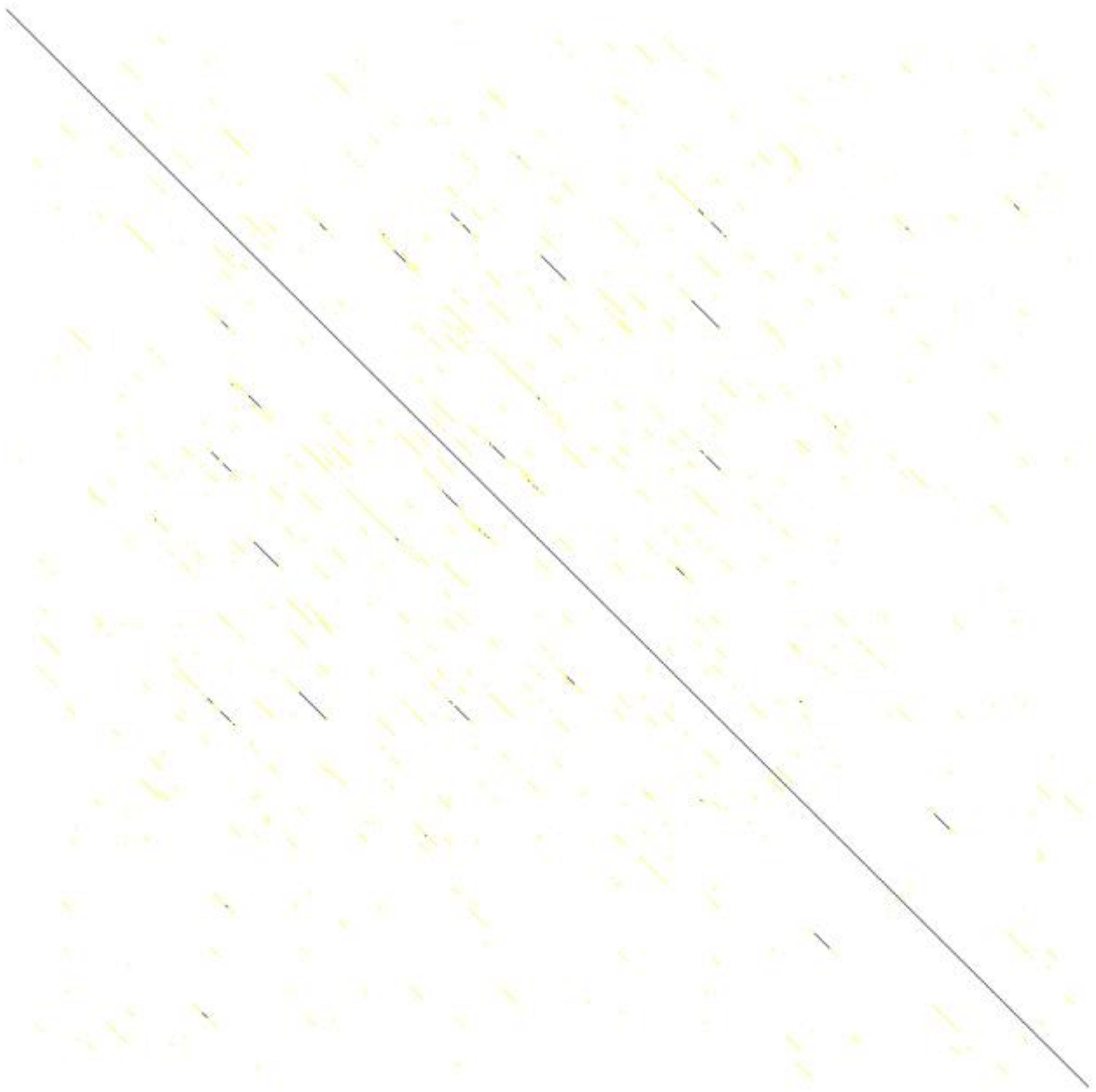

SI Fig. 1K: Consensus DOTTER fingerprint of FASTKD1 from the consensus of the sauropsid proteins using phylogeny (SI Fig. 1M) . Black pixels indicate a DOTTER score ≥ 31 while yellow pixels indicate a DOTTER score between 31 and 10 and self to self identity is indicated by the central diagonal line.

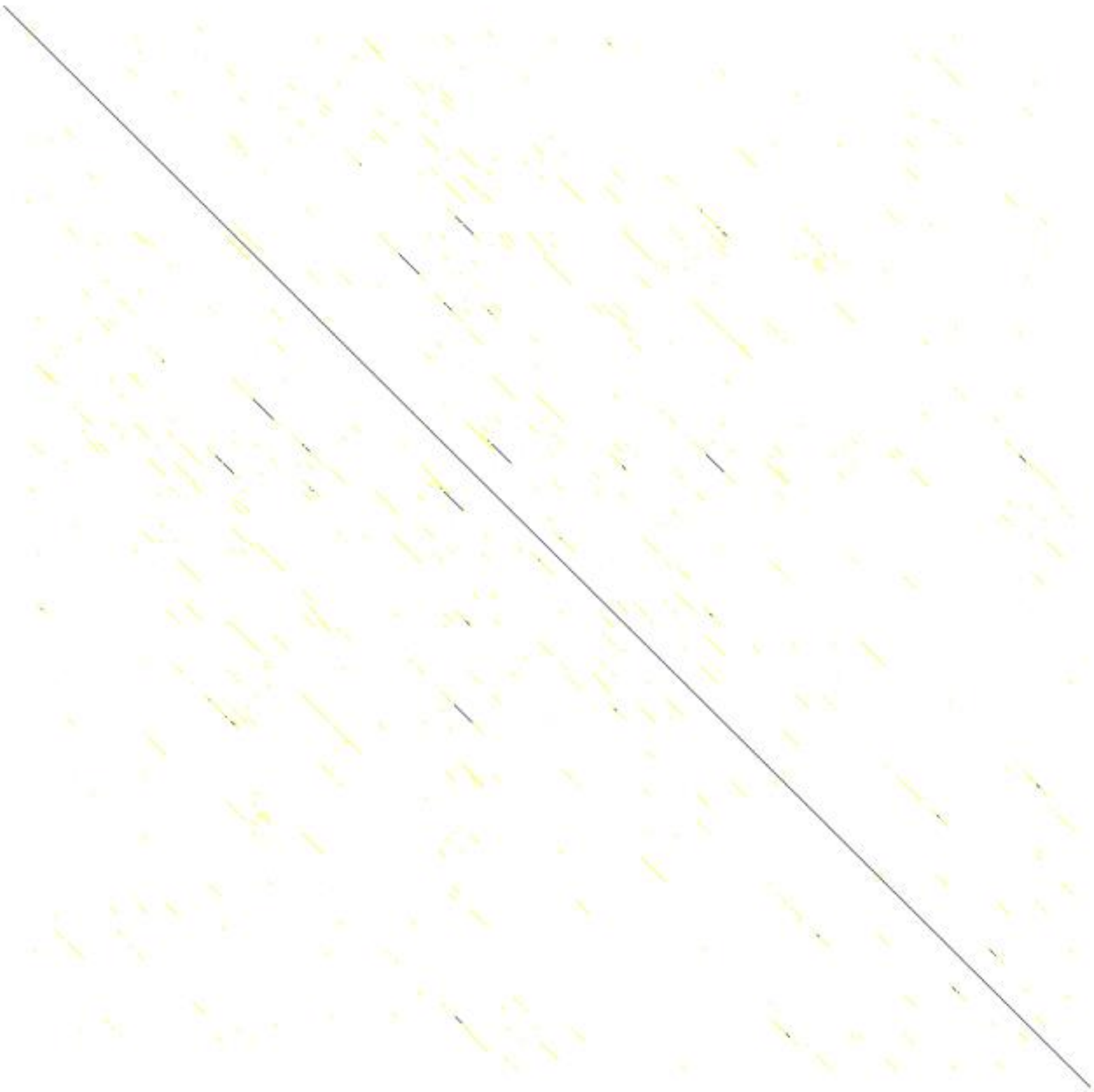

SI Fig. 1L: Consensus DOTTER fingerprint of FASTKD1 from the consensus of the mammalian proteins using phylogeny (SI Fig. 1M) . Black pixels indicate a DOTTER score ≥ 31 while yellow pixels indicate a DOTTER score between 31 and 10 and self to self identity is indicated by the central diagonal line.

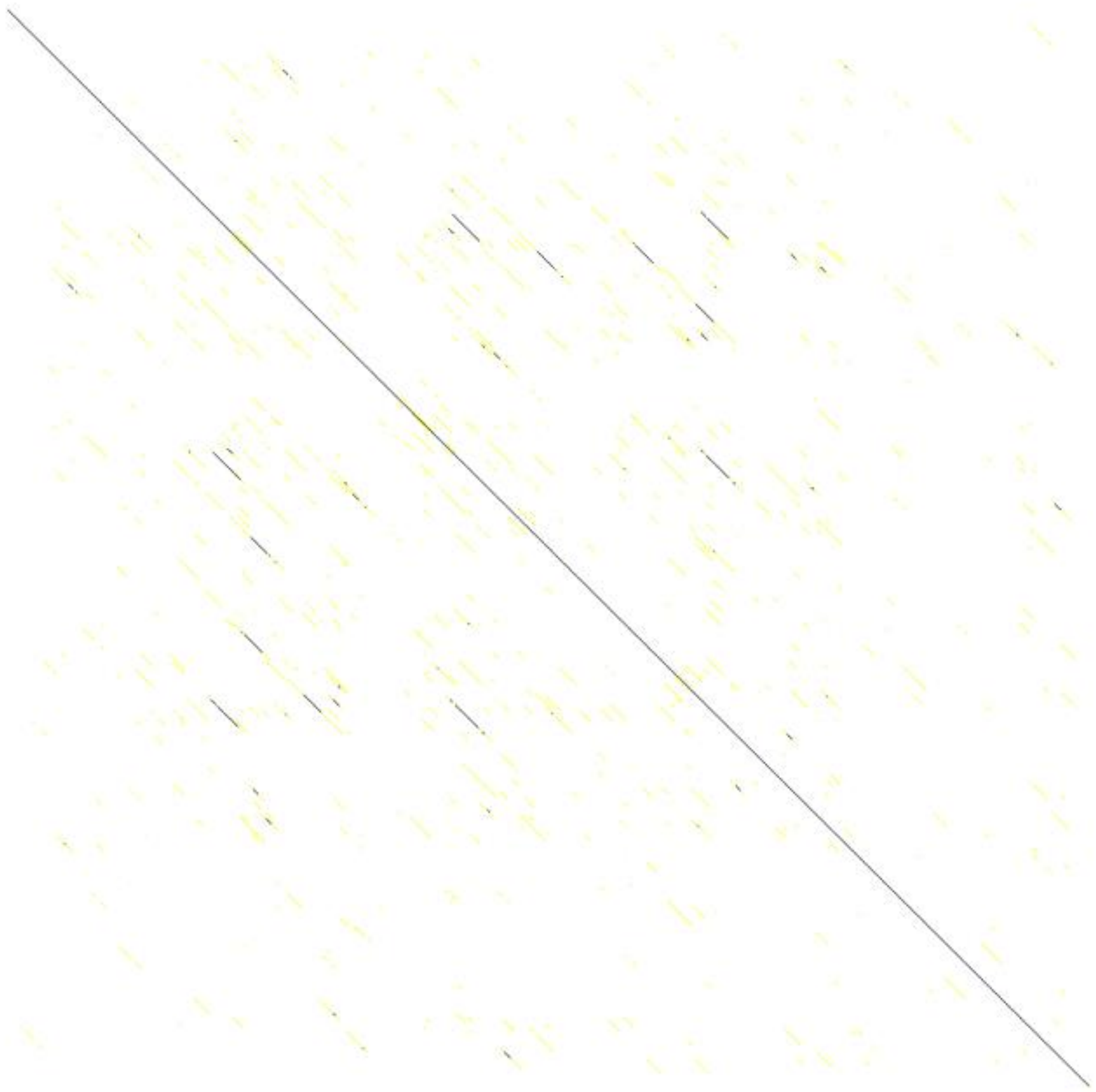

SI Fig. 1M: Phylogenetic tree of FASTKD1 proteins made using MrBayes. Colored text indicates the proteins used to build consensus DOTTER fingerprints for the mammalian (red), sauropsid (green), and fish/amphibian (blue) sets.

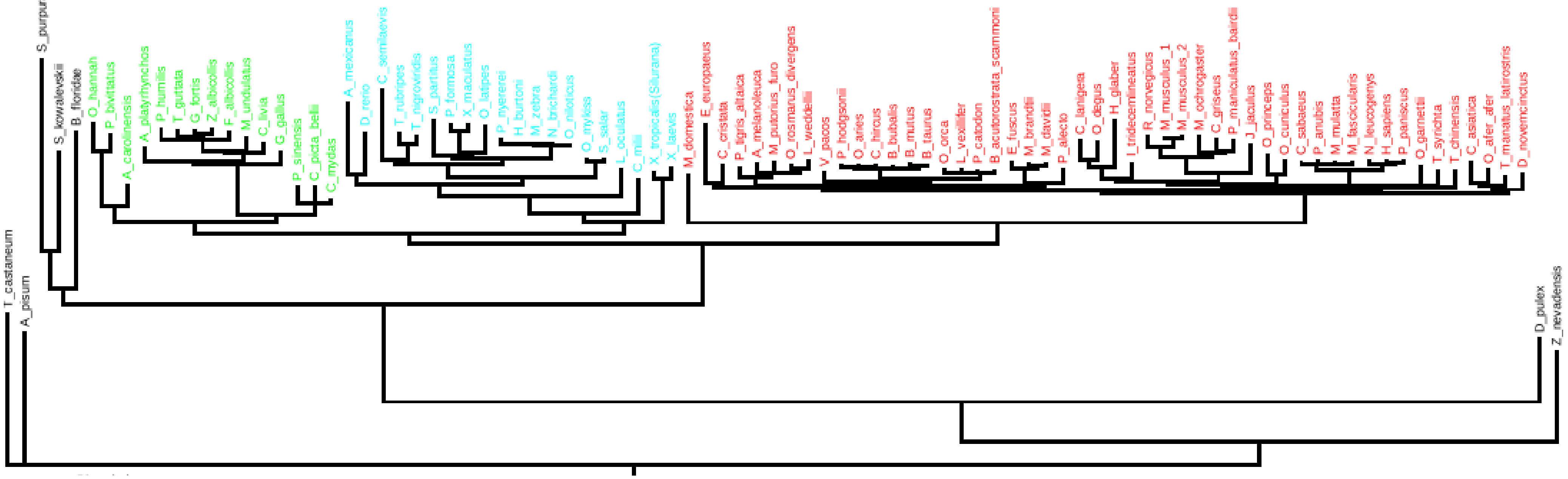

SI Fig. 1N: Consensus DOTTER fingerprint of RAP proteins from plants using all the proteins in the set (SI Fig. 1O) . Black pixels indicate a DOTTER score ≥ 31 while yellow pixels indicate a DOTTER score between 31 and 10 and self to self identity is indicated by the central diagonal line. Optimal clustering appeared to include all the proteins in this set, suggesting a rather strong conservation of the signal.

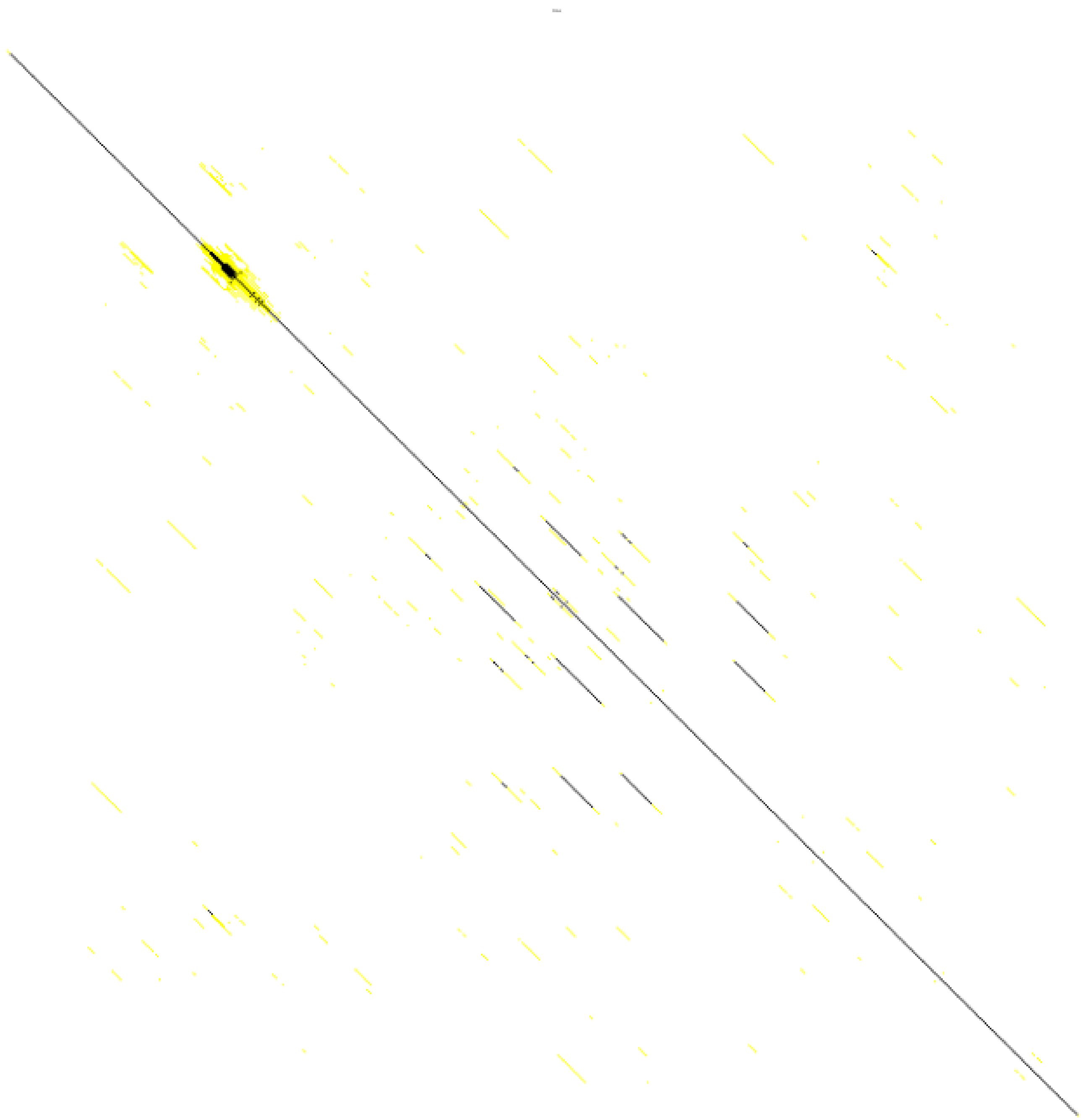

SI Fig. 1O: Phylogenetic tree of plant RAP proteins made using MrBayes. The proteins used to build the consensus DOTTER fingerprint are indicated with red text. No dot plot clustering diagram is included as all the proteins in the set were used to make the consensus dot plot figure as the consensus pattern was visually obvious.

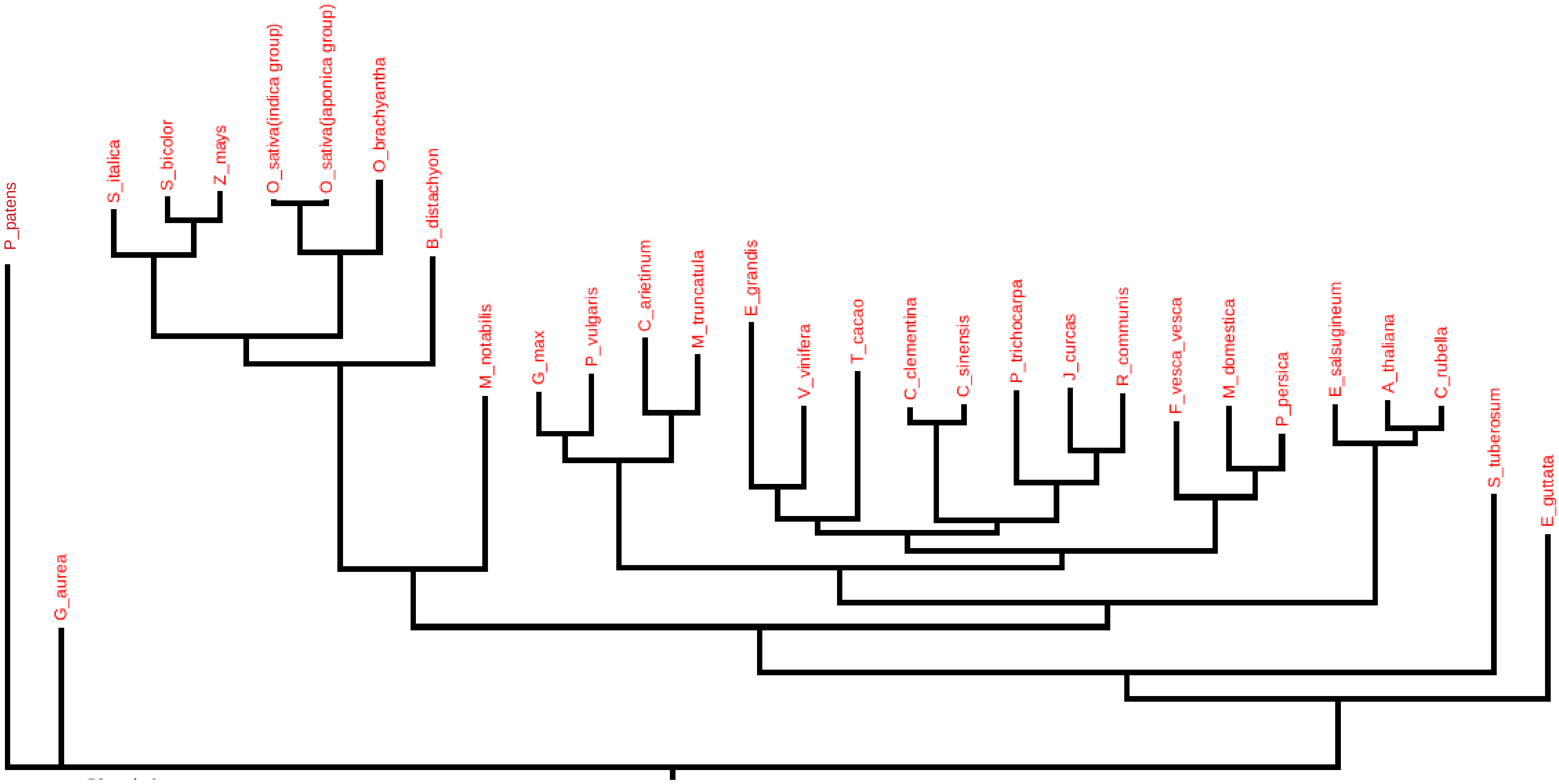

SI Fig. 1P: Consensus DOTTER fingerprint of fibrinogen from the consensus of the mammalian proteins (left) using phylogeny (SI Fig. 1Q) and from a different mammalian group (right) found using the fingerprint method (SI Fig. 1R) Black pixels indicate a DOTTER score ≥ 31 while yellow pixels indicate a DOTTER score between 31 and 10 and self to self identity is indicated by the central diagonal lines.

phylogeny-based consensus

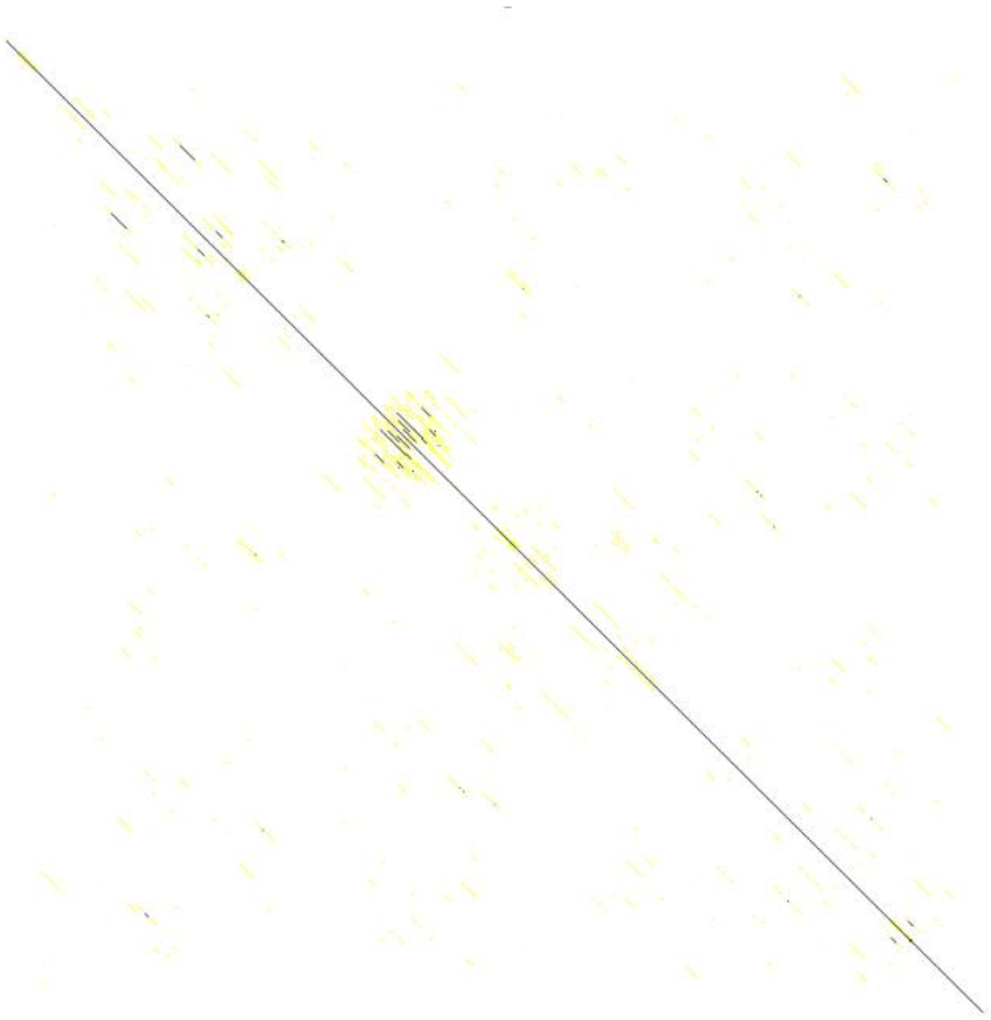

fingerprint-based consensus

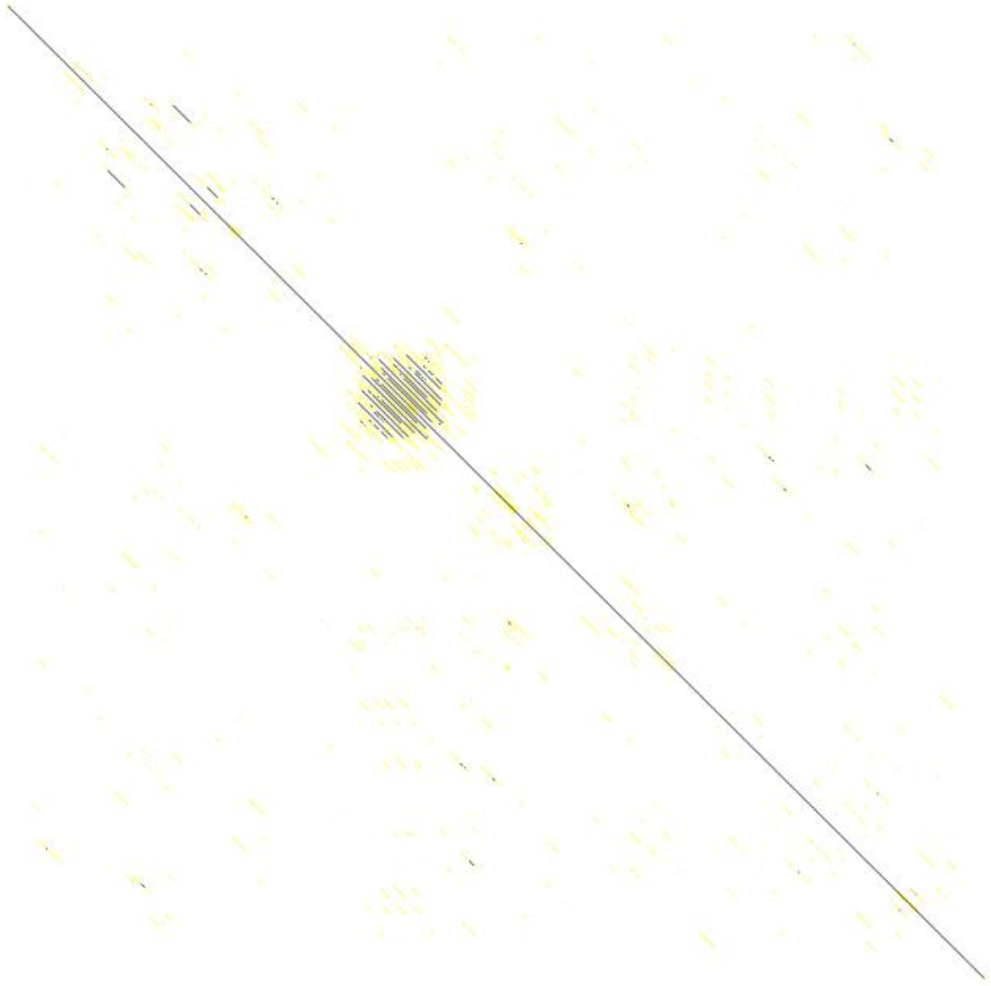

SI Fig. 1Q: Phylogenetic tree of fibrinogen proteins made using MrBayes. The mammalian proteins used to build the consensus DOTTER fingerprint are indicated with red text.

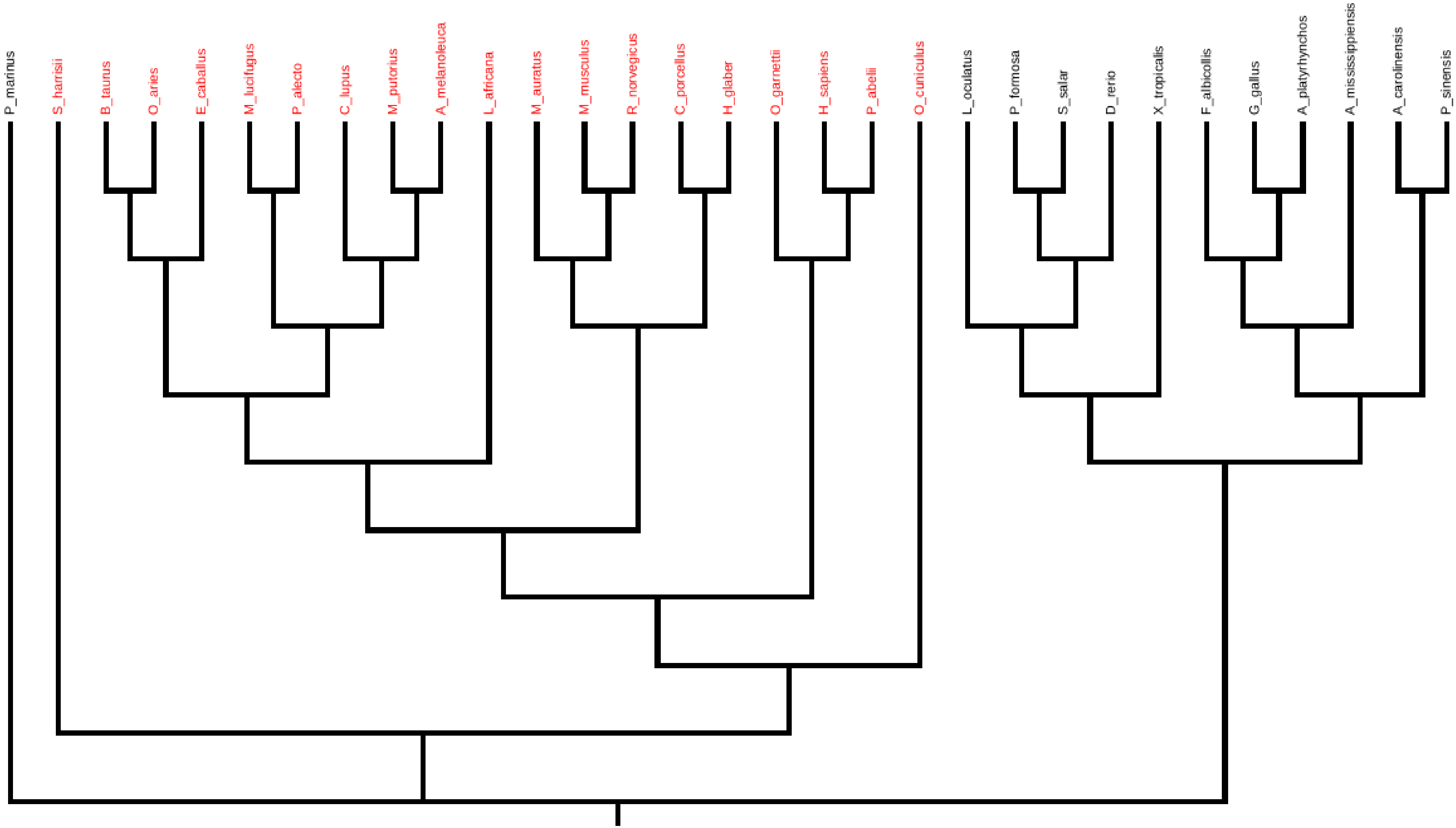

SI Fig. 1R: Average linkage hierarchical clustering of DOTTER fingerprints for the set of fibrinogen proteins. The mammalian proteins used to make the consensus fingerprint are indicated by the red box.

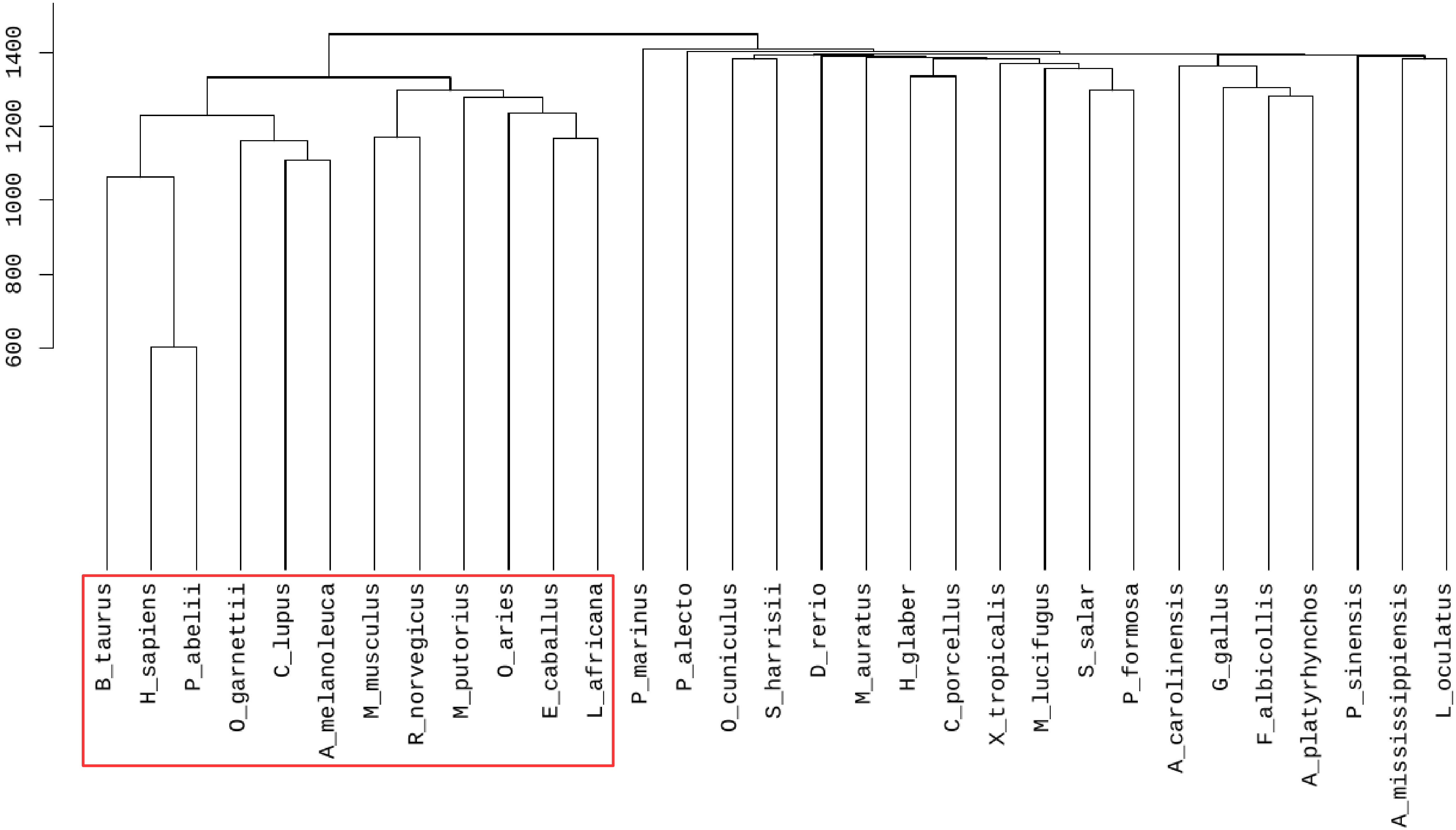

SI Fig. 1S: Consensus DOTTER fingerprint of IFABP from the consensus of the vertebrate proteins using phylogeny (SI Fig. 1T) . Black pixels indicate a DOTTER score ≥ 31 while yellow pixels indicate a DOTTER score between 31 and 10 and self to self identity is indicated by the central diagonal line. The lack of any black pixels in this dot plot classifies it as a failure for this method.

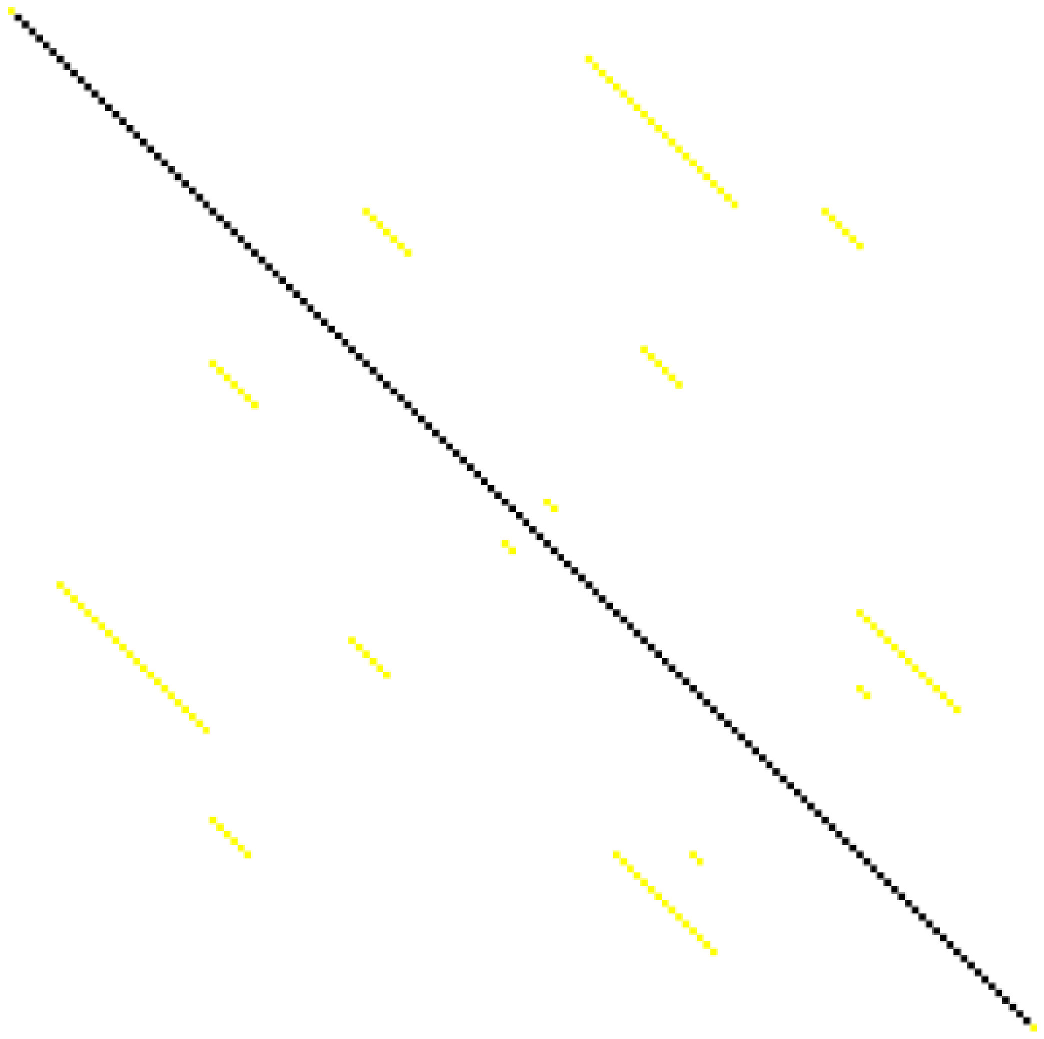

SI Fig. 1T: Phylogenetic tree of IFABP proteins made using MrBayes. The mammalian proteins used to build the consensus DOTTER fingerprint are indicated with red text.

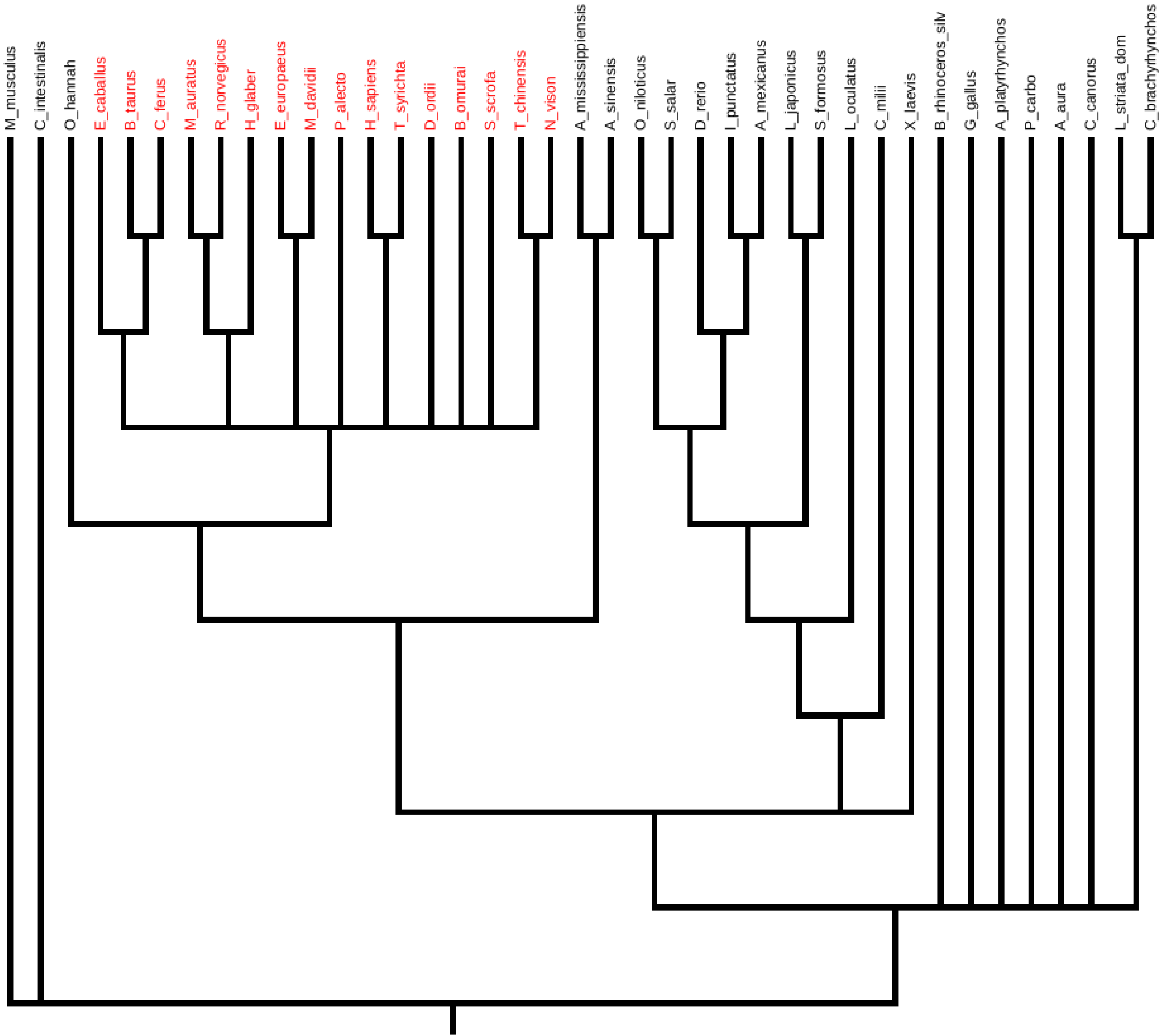

SI Fig. 1U: Complete linkage hierarchical clustering of DOTTER fingerprints for the set of IFABP proteins. The mammalian proteins used to make the consensus fingerprint are indicated by the red boxes.

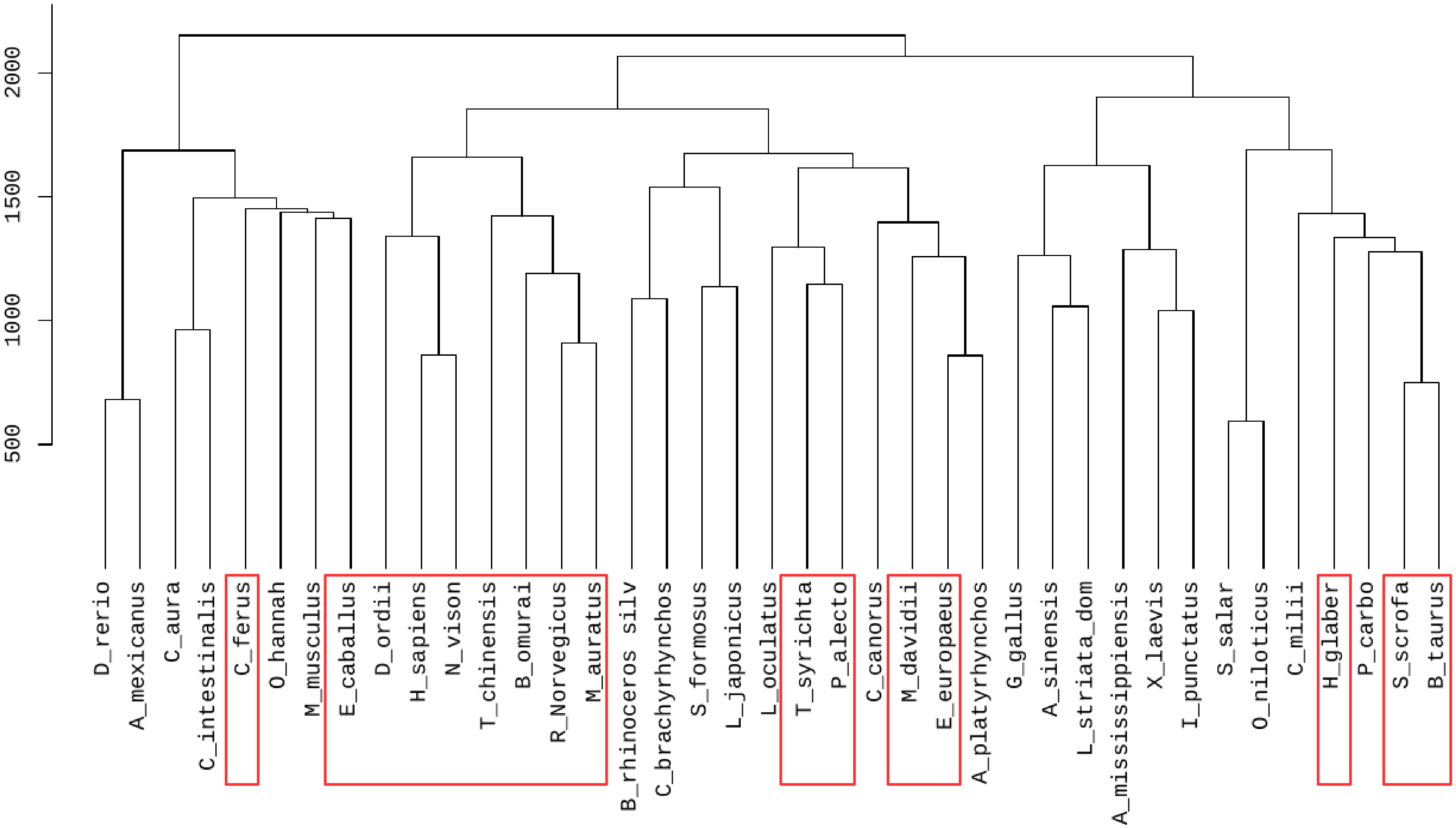

SI Fig. 1V: Consensus DOTTER fingerprint of IFIT3 based on the consensus all the protein sequences in the sample set. Because the total set size was small (N=7), all proteins were used to generate the fingerprint. Black pixels indicate a DOTTER score ≥ 31 while yellow pixels indicate a DOTTER score between 31 and 10 and self to self identity is indicated by the central diagonal line.

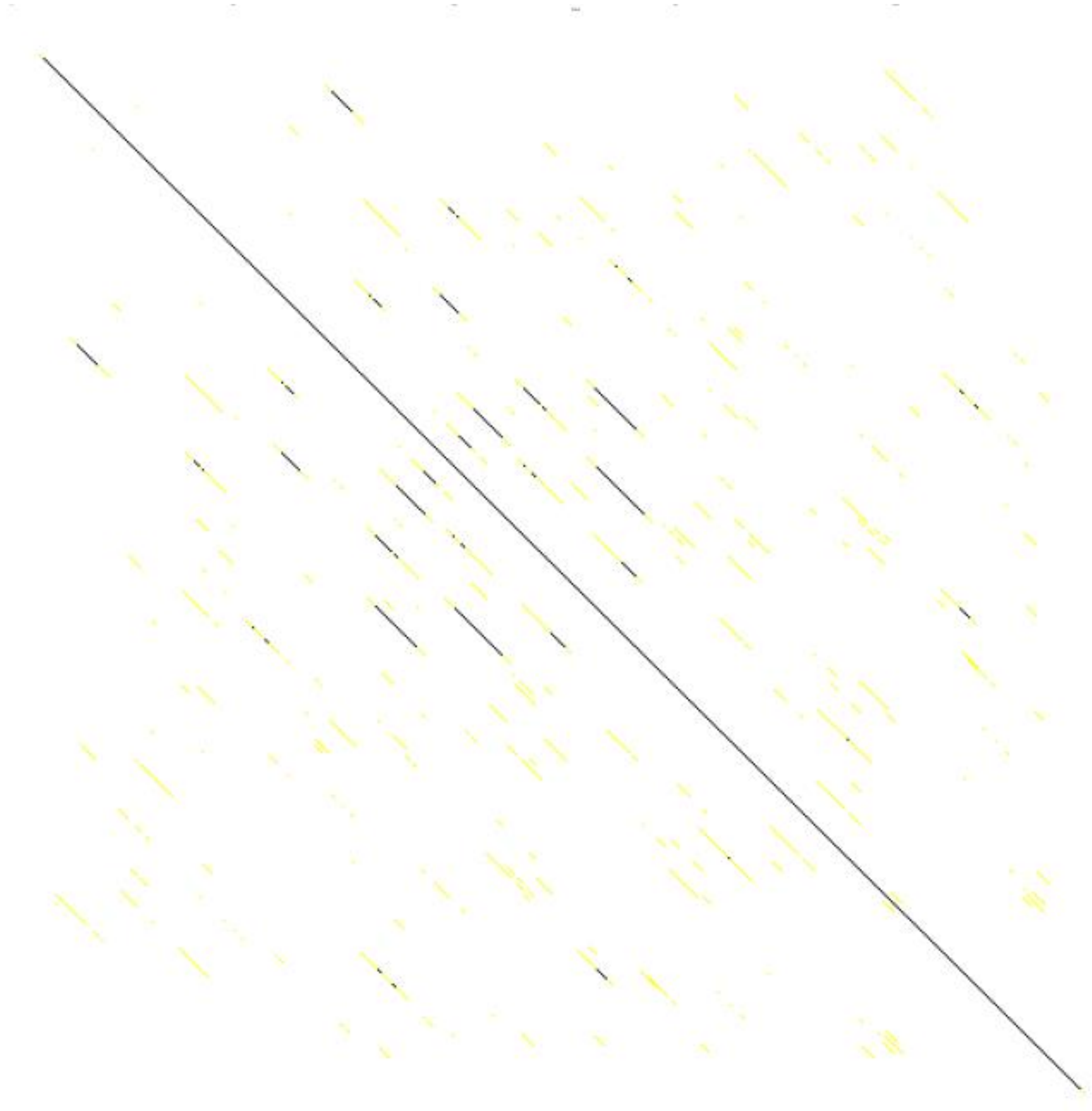

SI Fig. 1W: Consensus DOTTER fingerprint of IFIT5 based on all the protein sequences in the sample set. Because the total set size was small (N=5), all proteins were used to generate the fingerprint. Black pixels indicate a DOTTER score ≥ 31 while yellow pixels indicate a DOTTER score between 31 and 10 and self to self identity is indicated by the central diagonal line.

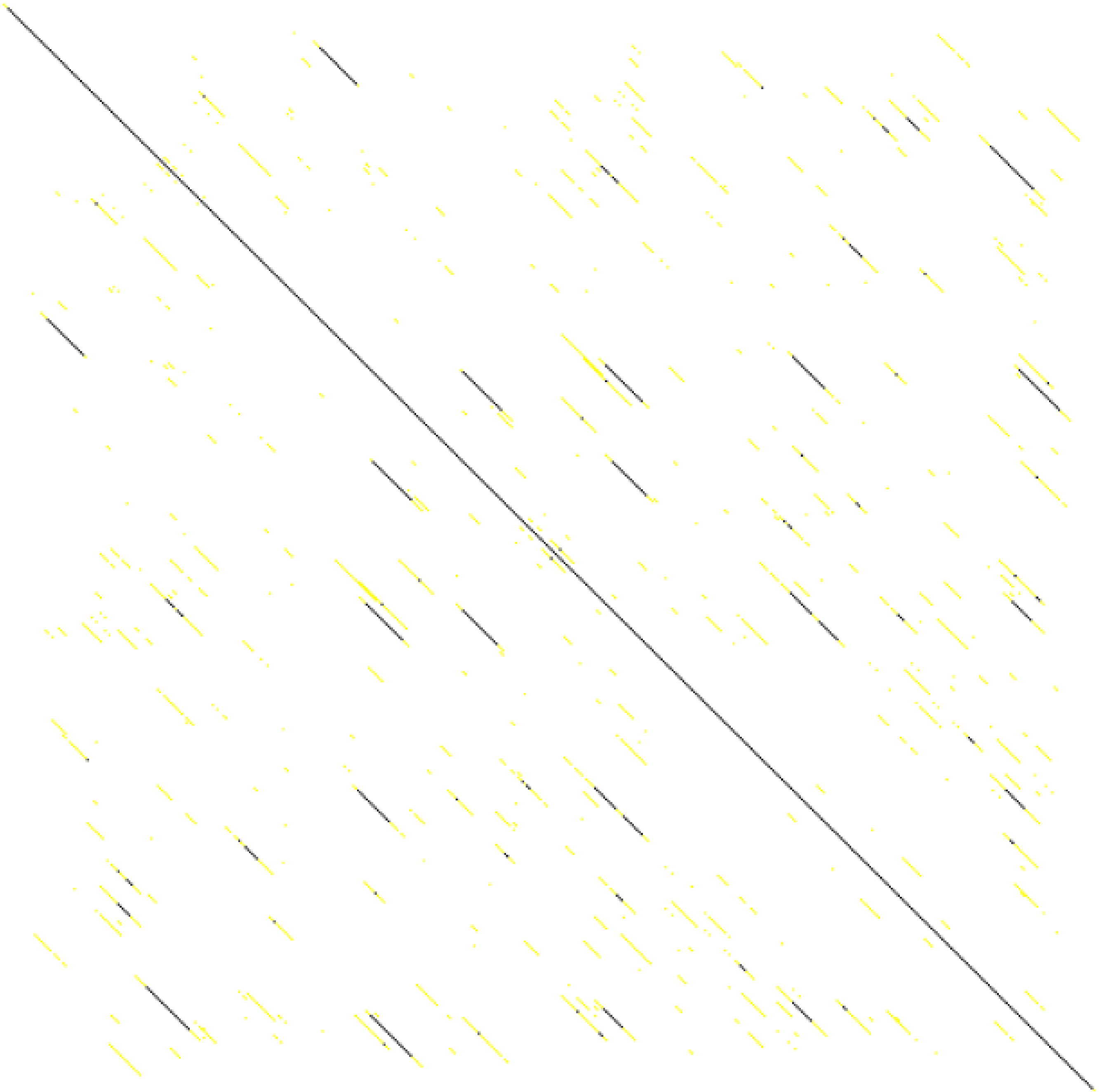

SI Fig. 1X: Consensus DOTTER fingerprint of PCNA from the consensus of the vertebrate proteins using the fingerprint method (SI Fig. 1Z) . Black pixels indicate a DOTTER score ≥ 31 while yellow pixels indicate a DOTTER score between 31 and 10 and self to self identity is indicated by the central diagonal line. The lack of any black pixels in this dot plot classifies it as a failure for this method.

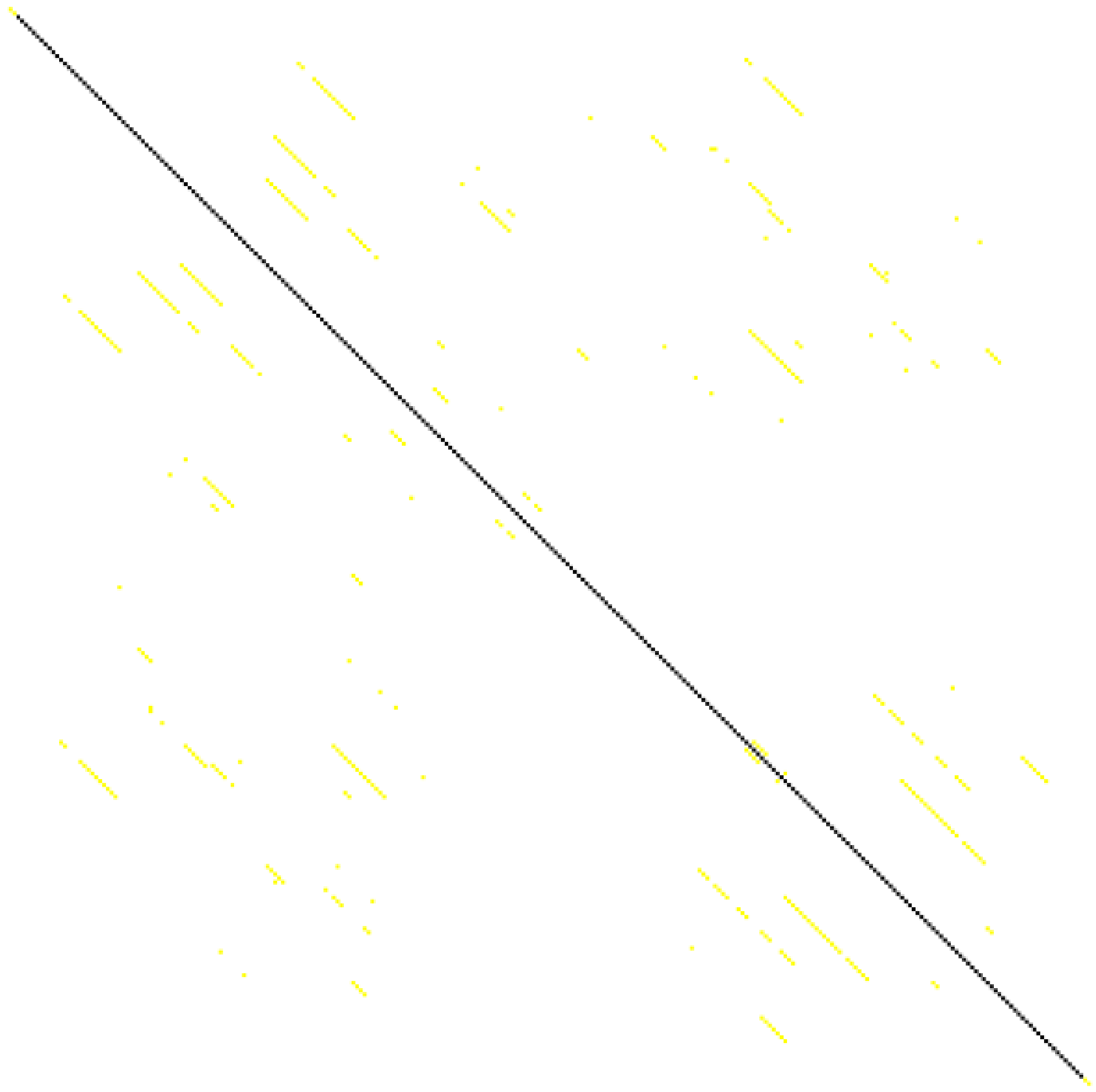

SI Fig. 1Y: Phylogenetic tree of PCNA made using MrBayes. The vertebrate proteins used to build the consensus DOTTER fingerprint are indicated with red text.

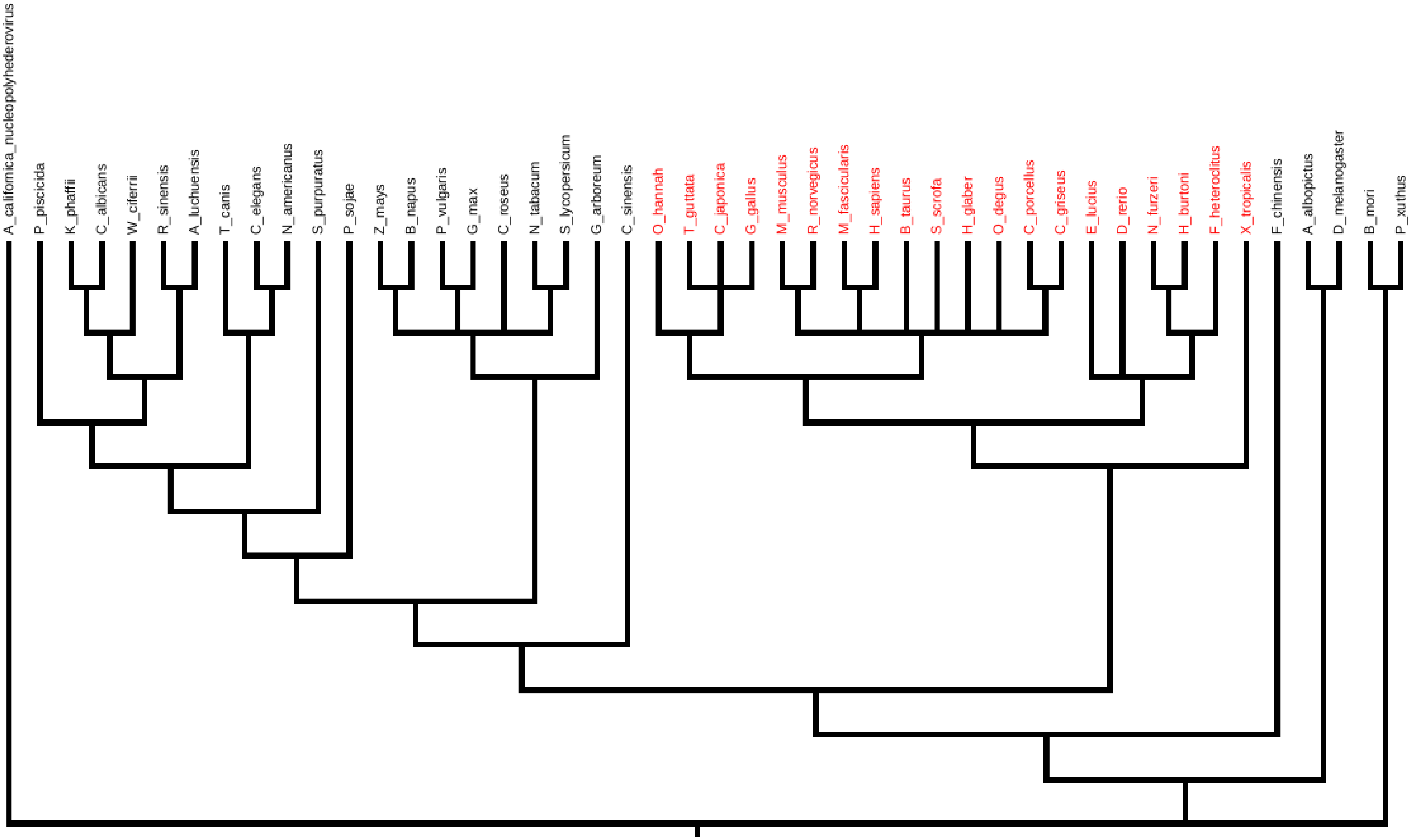

SI Fig. 1Z: Complete linkage hierarchical clustering of DOTTER fingerprints for the set of PCNA proteins. The mammalian proteins used to make the consensus fingerprint are indicated by the red box.

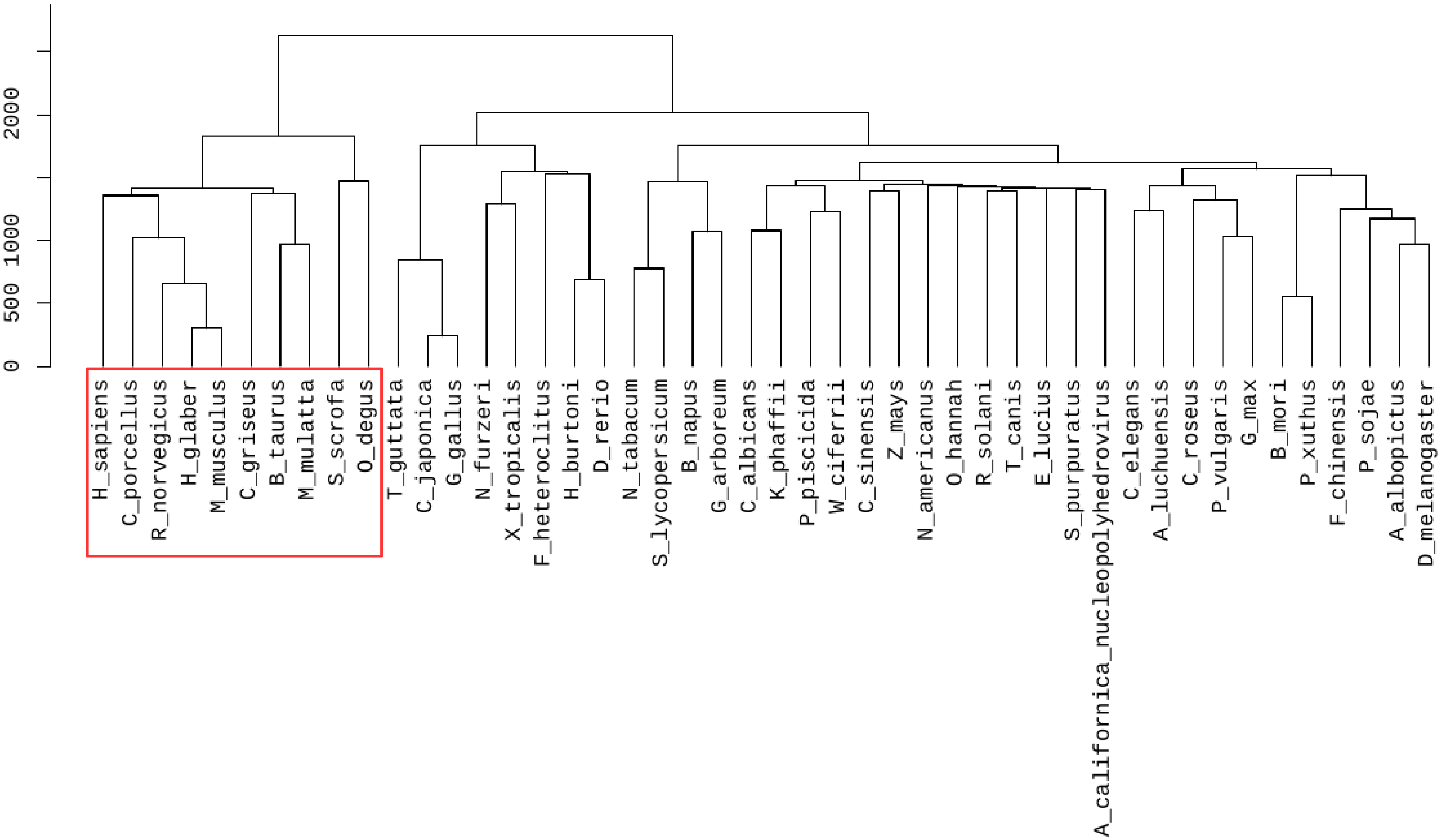

SI Fig. 1AA: Consensus DOTTER fingerprint of RCC from the consensus of all the proteins in the test set using both phylogeny (SI Fig. 1AB) and the fingerprint method (SI Fig. 1AC) . Black pixels indicate a DOTTER score ≥ 31 while yellow pixels indicate a DOTTER score between 31 and 10 and self to self identity is indicated by the central diagonal line.

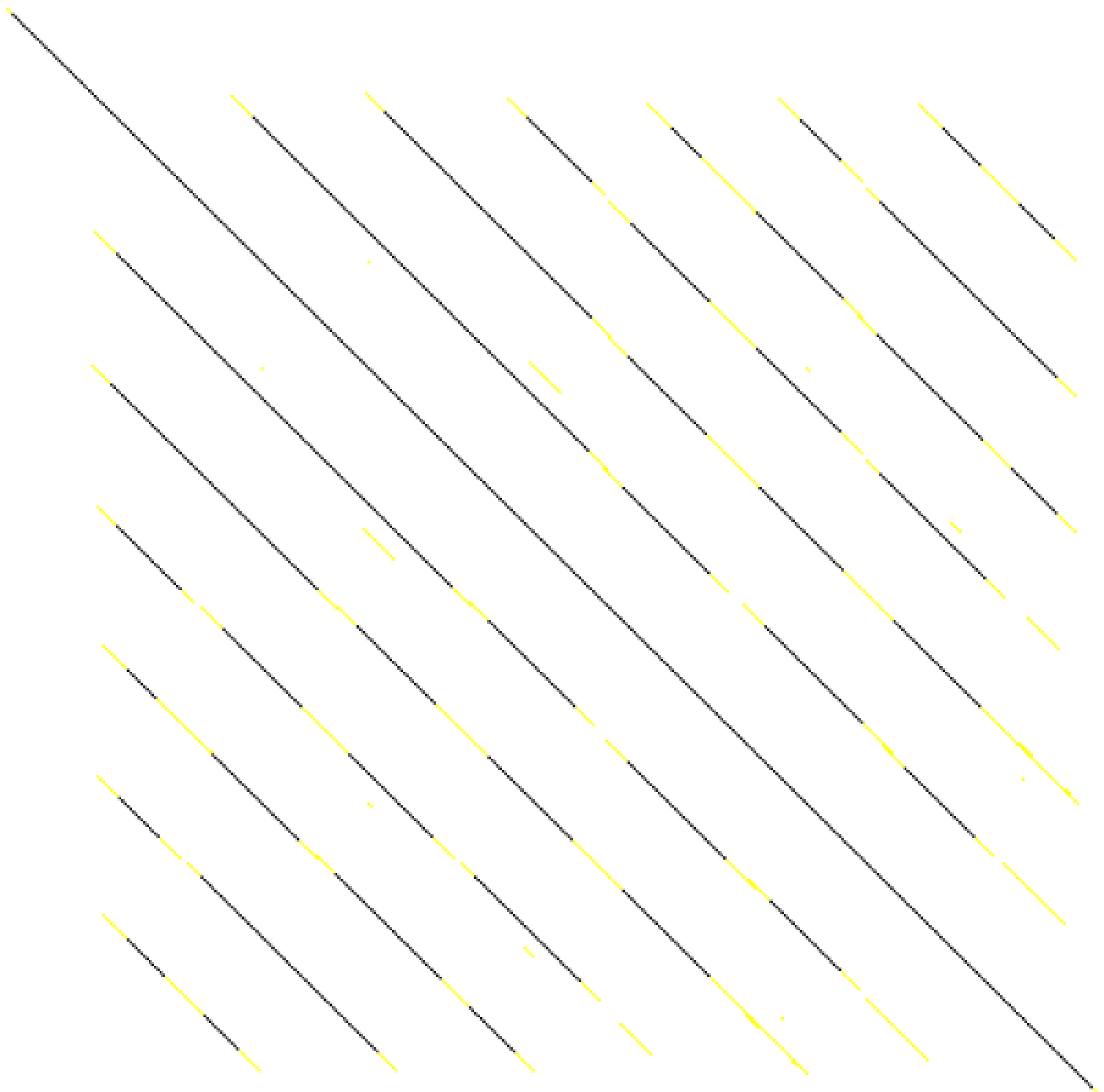

SI Fig. 1AB: Phylogenetic tree of RCC made using MrBayes. The proteins used to build the consensus DOTTER fingerprint are indicated with red text.

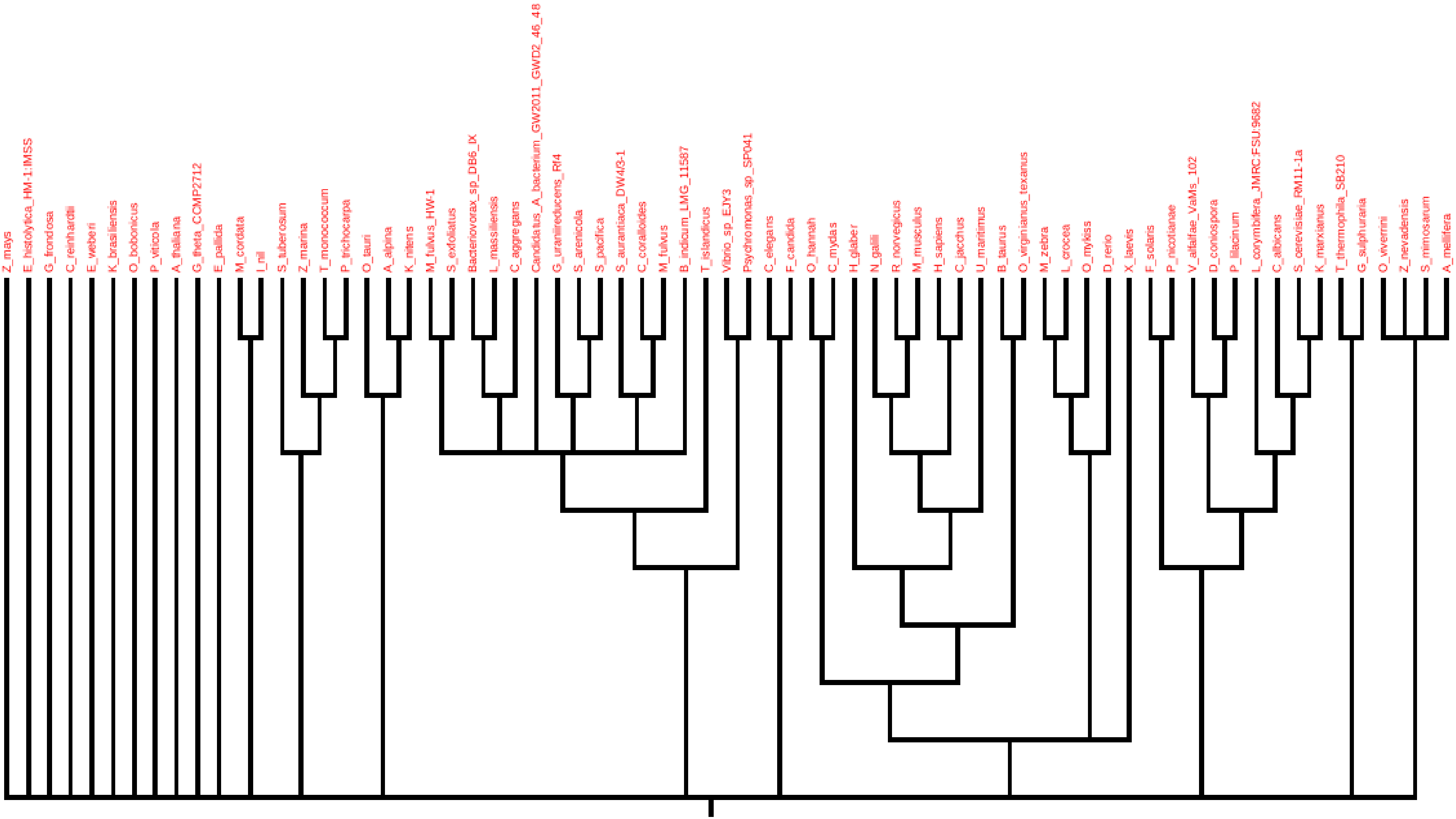

SI Fig. 1AC: Single linkage hierarchical clustering of DOTTER fingerprints for the set of RCC proteins. The proteins used to make the consensus fingerprint are indicated by the red box. . Optimal clustering appeared to include all the proteins in this set, suggesting a rather strong conservation of the signal.

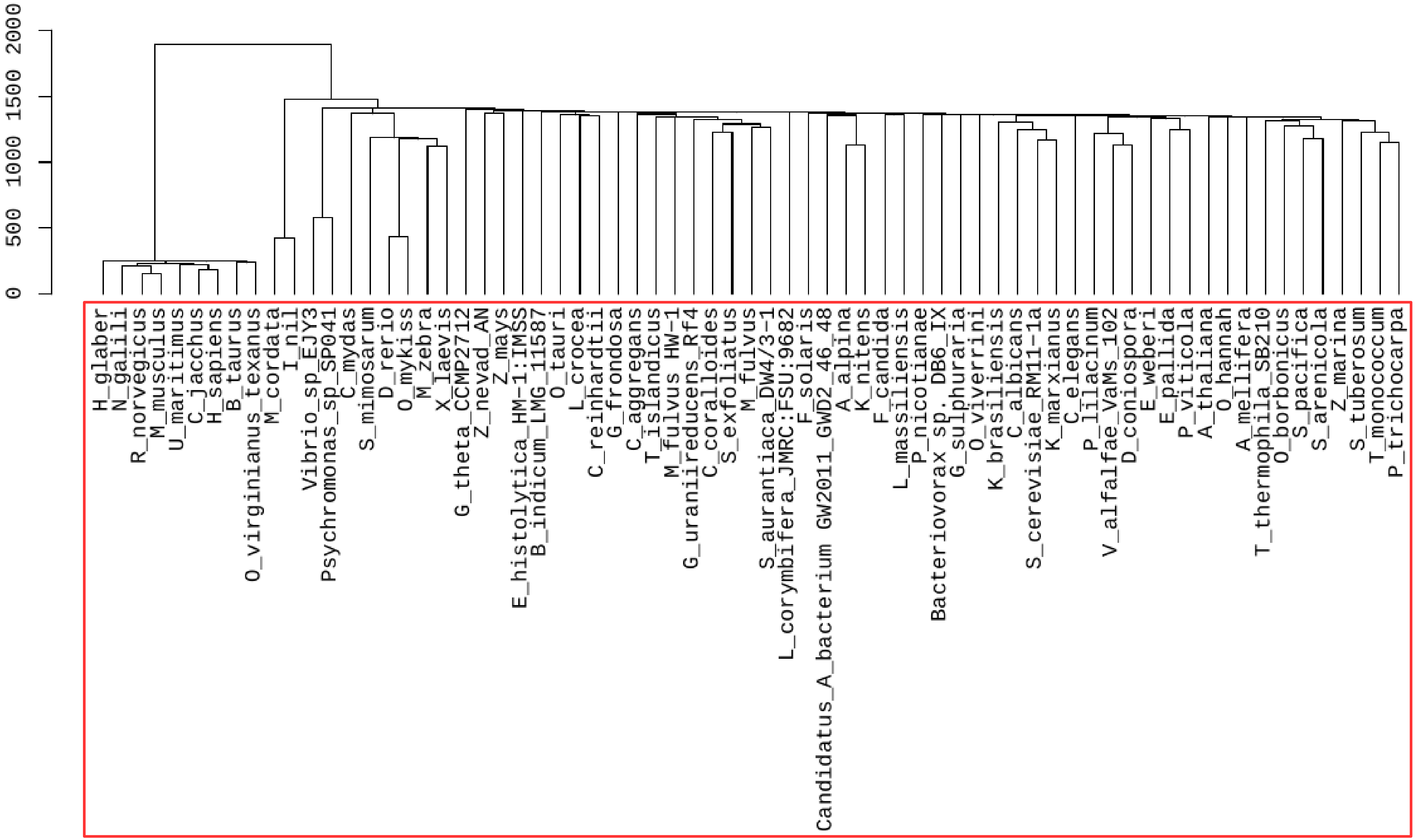

SI Fig. 1AD: Consensus DOTTER fingerprint of SKP2 from the consensus of the invertebrate proteins (left) using phylogeny (SI Fig. 1AE) and the non-mammalian grouping (right) found using the fingerprint method (SI Fig. 1AF) Black pixels indicate a DOTTER score ≥ 31 while yellow pixels indicate a DOTTER score between 31 and 10 and self to self identity is indicated by the central diagonal lines.

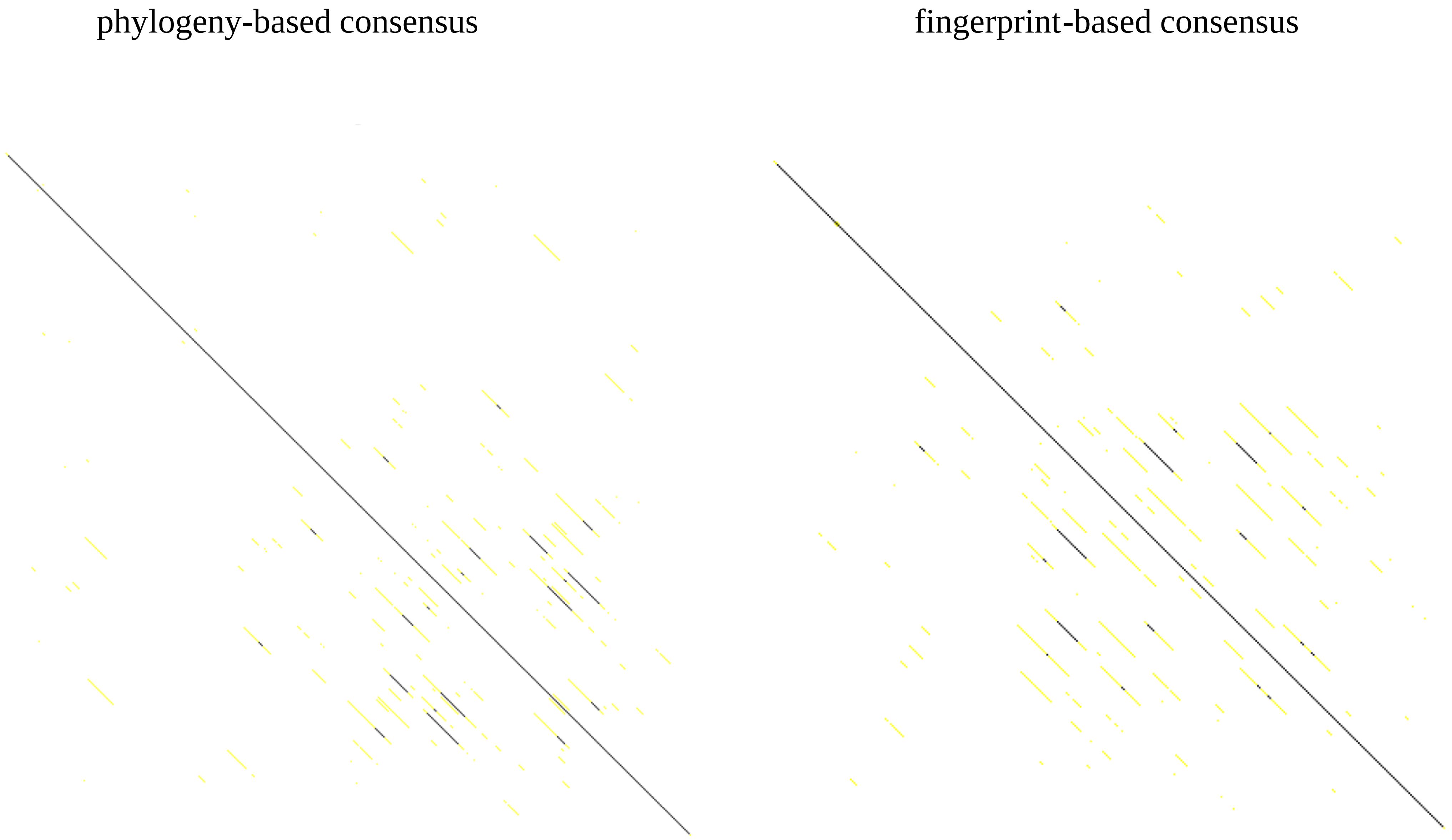

SI Fig. 1AE: Phylogenetic tree of SKP2 made using MrBayes. The group of invertebrate proteins used to build the consensus DOTTER fingerprint are indicated with red text.

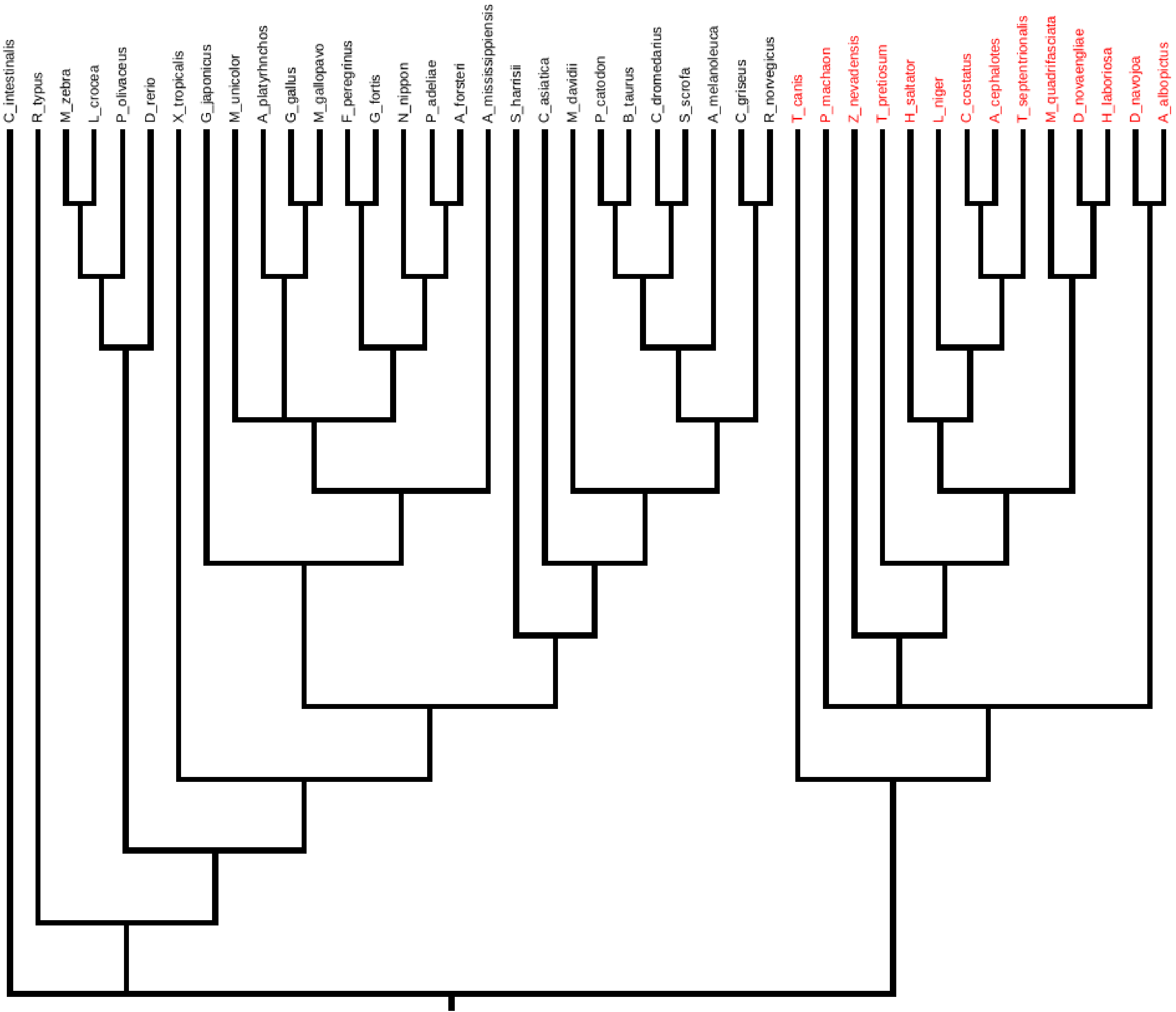

SI Fig. 1AF: Single linkage hierarchical clustering of DOTTER fingerprints for the set of SKP2 proteins. The animal proteins used to make the consensus fingerprint are indicated by the red box.

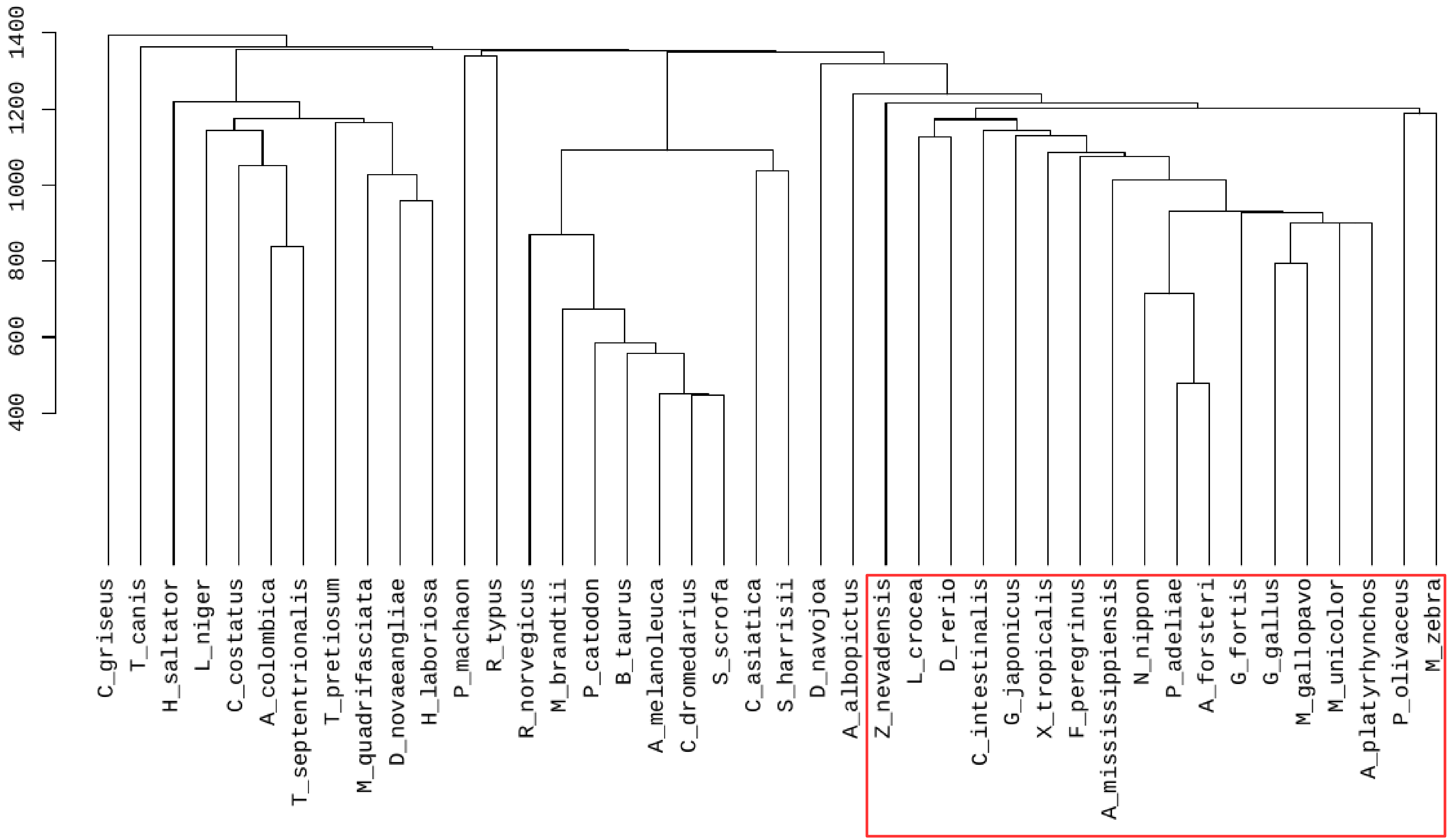

SI Fig. 1AG: Consensus DOTTER fingerprint of the Spindly protein from the consensus of the animal proteins (left) using phylogeny (SI Fig. 1AH) and the vertebrate group (right) found using the fingerprint method (SI Fig. 1AI) Black pixels indicate a DOTTER score ≥ 31 while yellow pixels indicate a DOTTER score between 31 and 10 and self to self identity is indicated by the central diagonal lines.

phylogeny-based consensus | fingerprint-based consensus

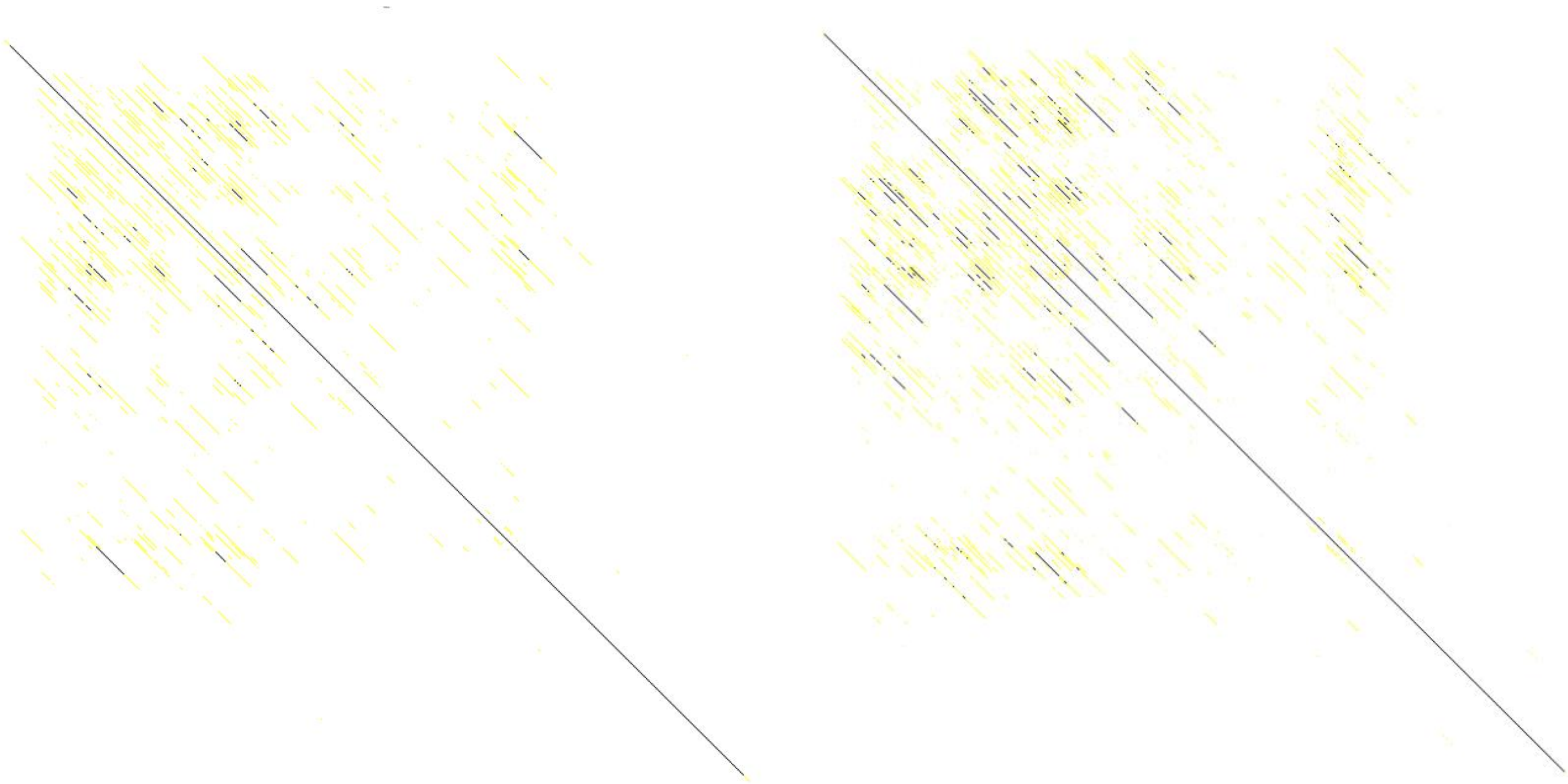

SI Fig. 1AH: Phylogenetic tree of Spindly proteins made using MrBayes. The animal proteins used to build the consensus DOTTER fingerprint are indicated with red text.

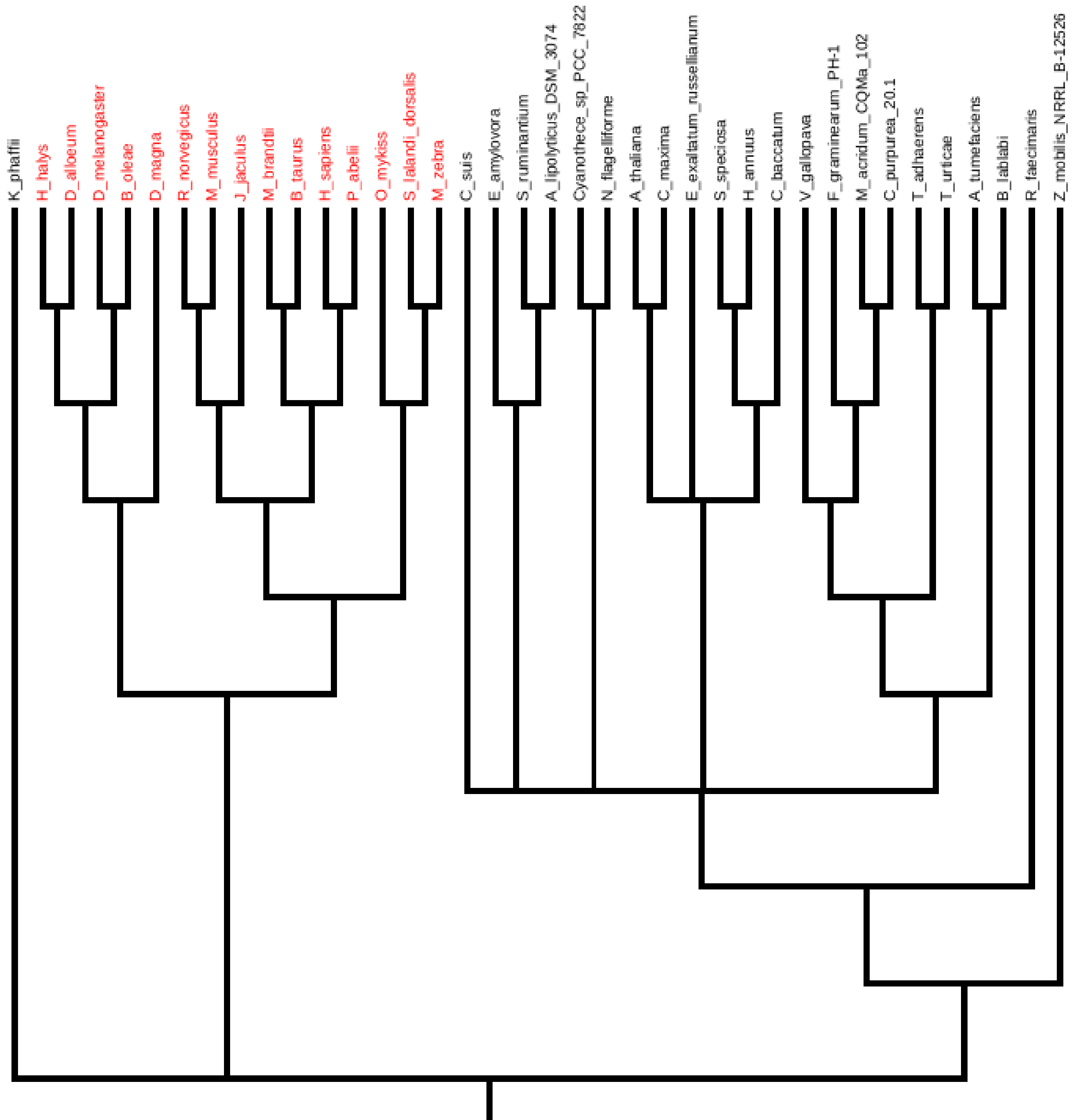

SI Fig. 1AI: Average linkage hierarchical clustering of DOTTER fingerprints for the set of Spindly proteins. The vertebrate proteins used to make the consensus fingerprint are indicated by the red box

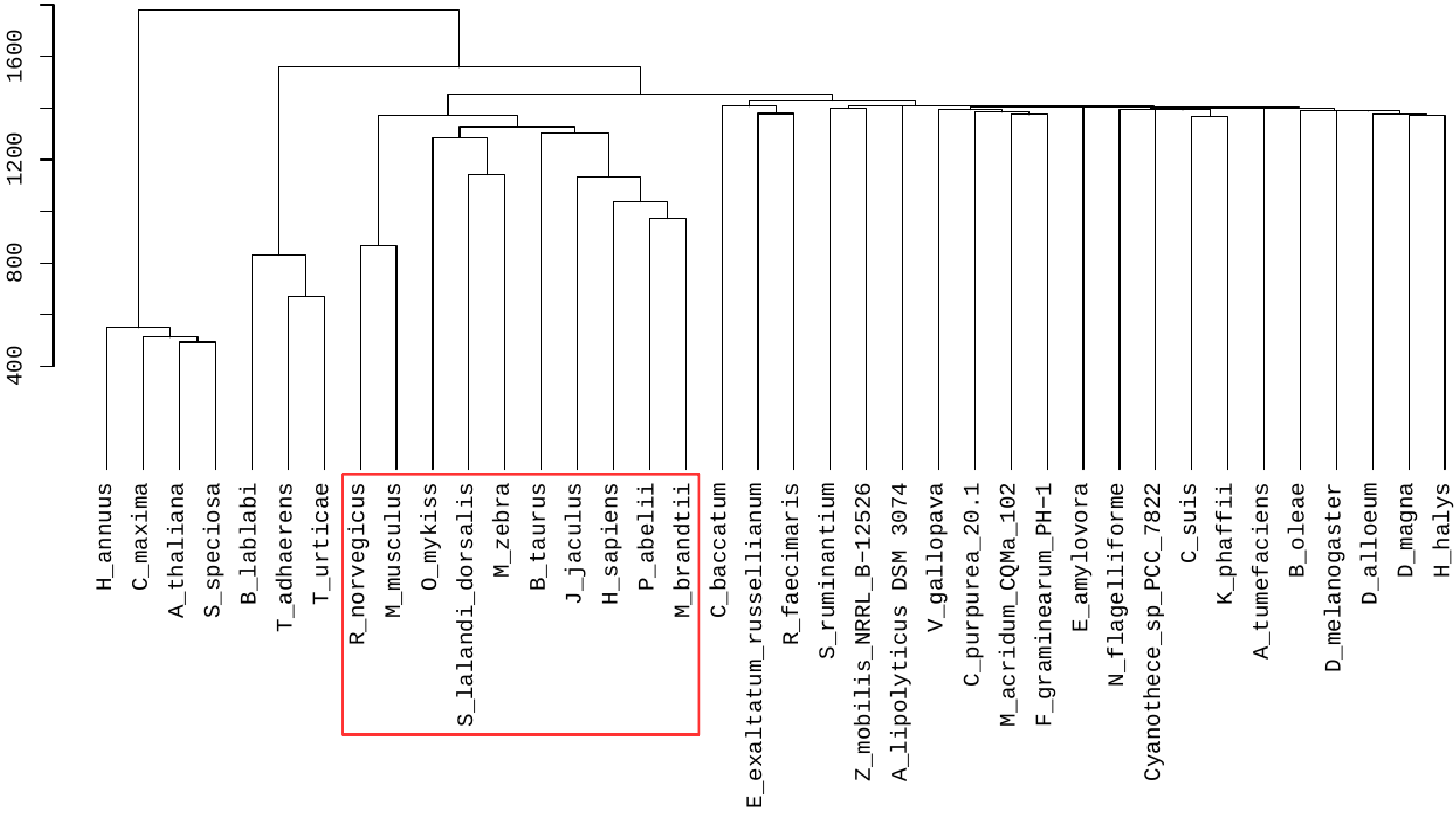

SI Fig. 2A: Histogram of the information content in the DOTTER plots generated from each member of RepeatsDB which contains all the currently confirmed repeat proteins in the PDB.

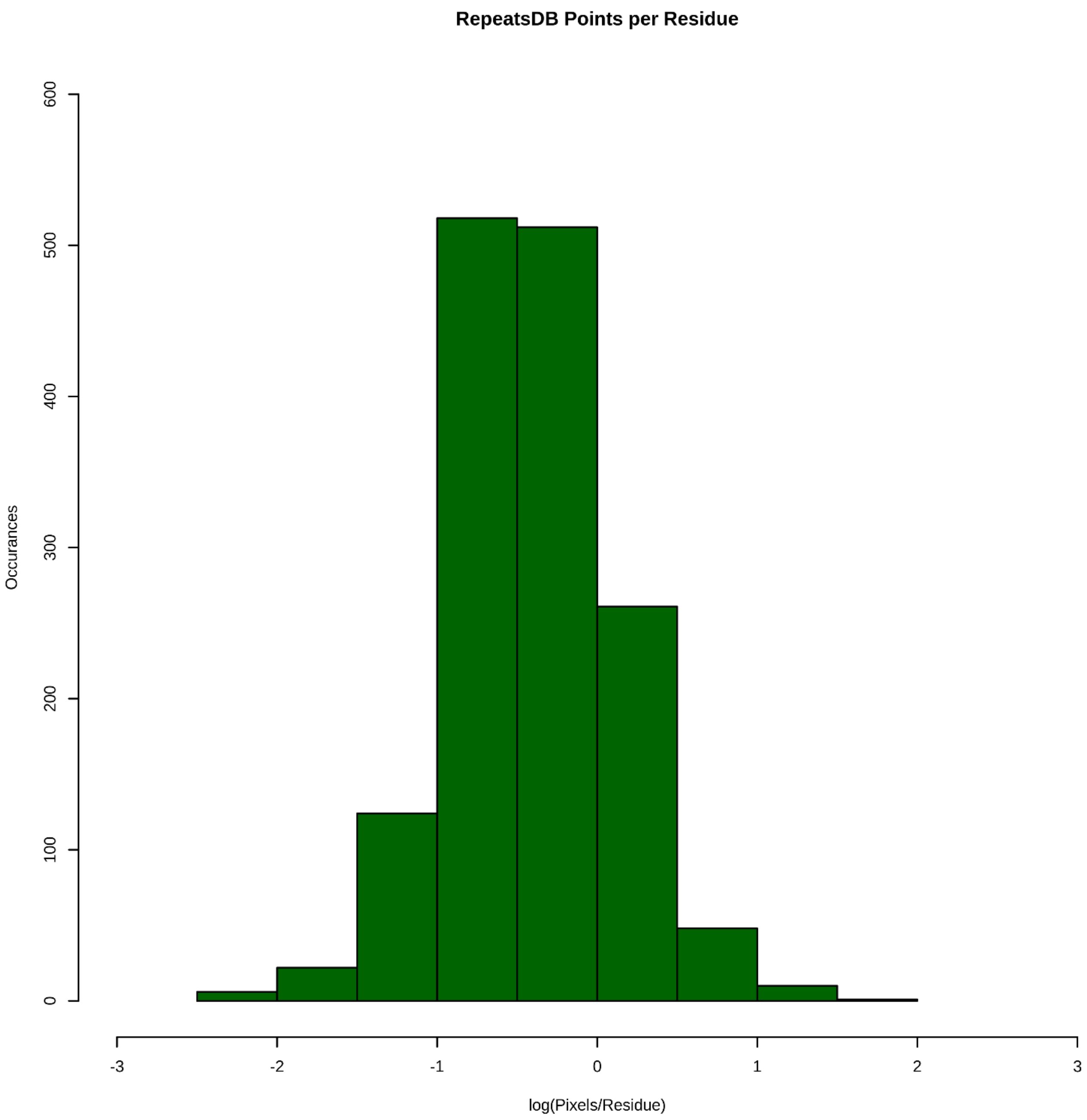

SI Fig. 2B: Histogram of the information content in the DOTTER plots generated from a set of proteins in the PDB found by searching for the keyword “mouse” (n=985). These are generally expected to be non-repeat proteins although it is expected that about 10-20% of all proteins are repeat protiens.

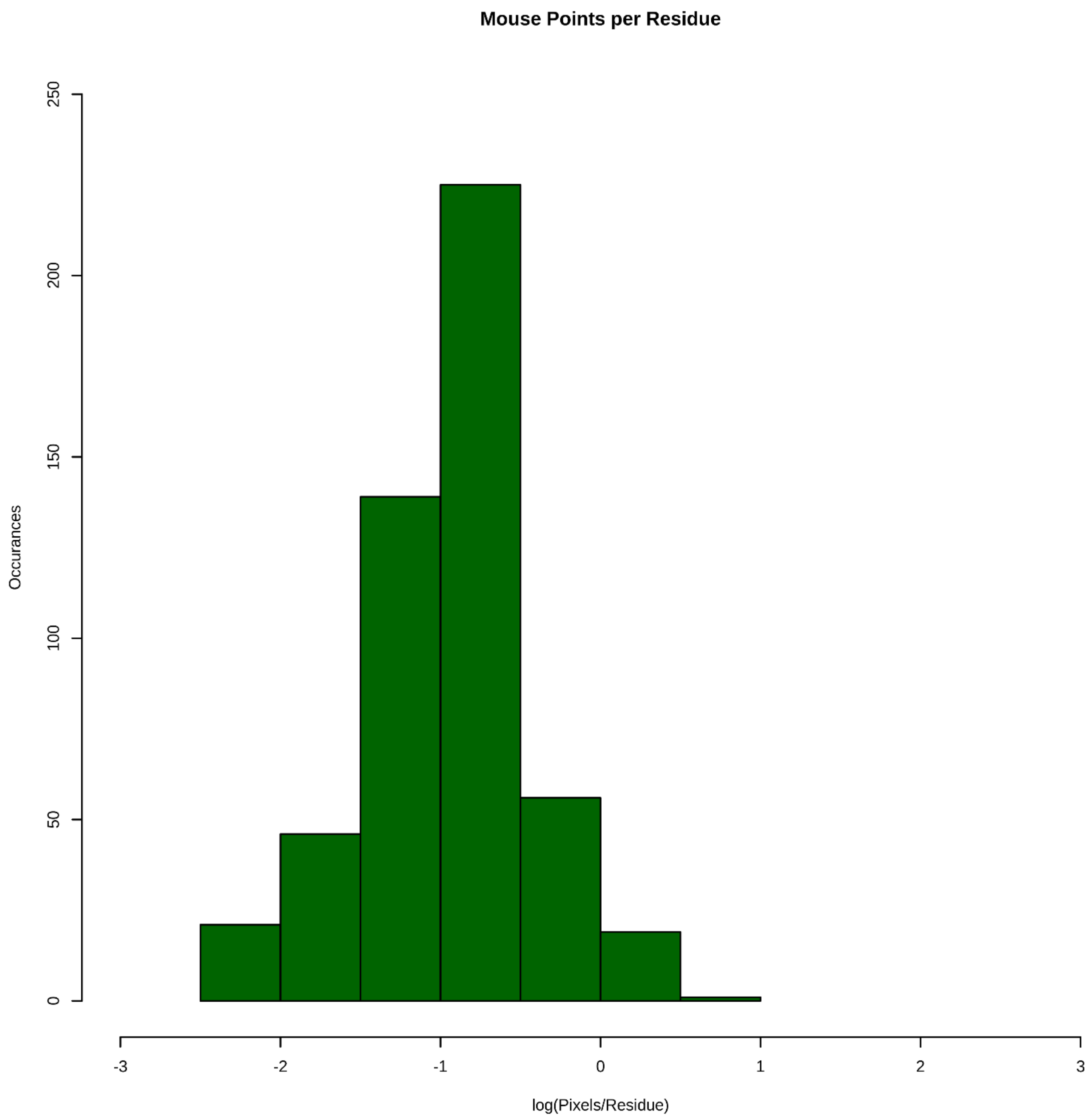

SI Fig. 2C: Histogram of the information content in the DOTTER plots generated from a set of proteins in the PDB found by searching for the keyword "bacillus" (n=1325). These are generally expected to be non-repeat proteins although it is expected that about 10-20% of all proteins are repeat proteins.

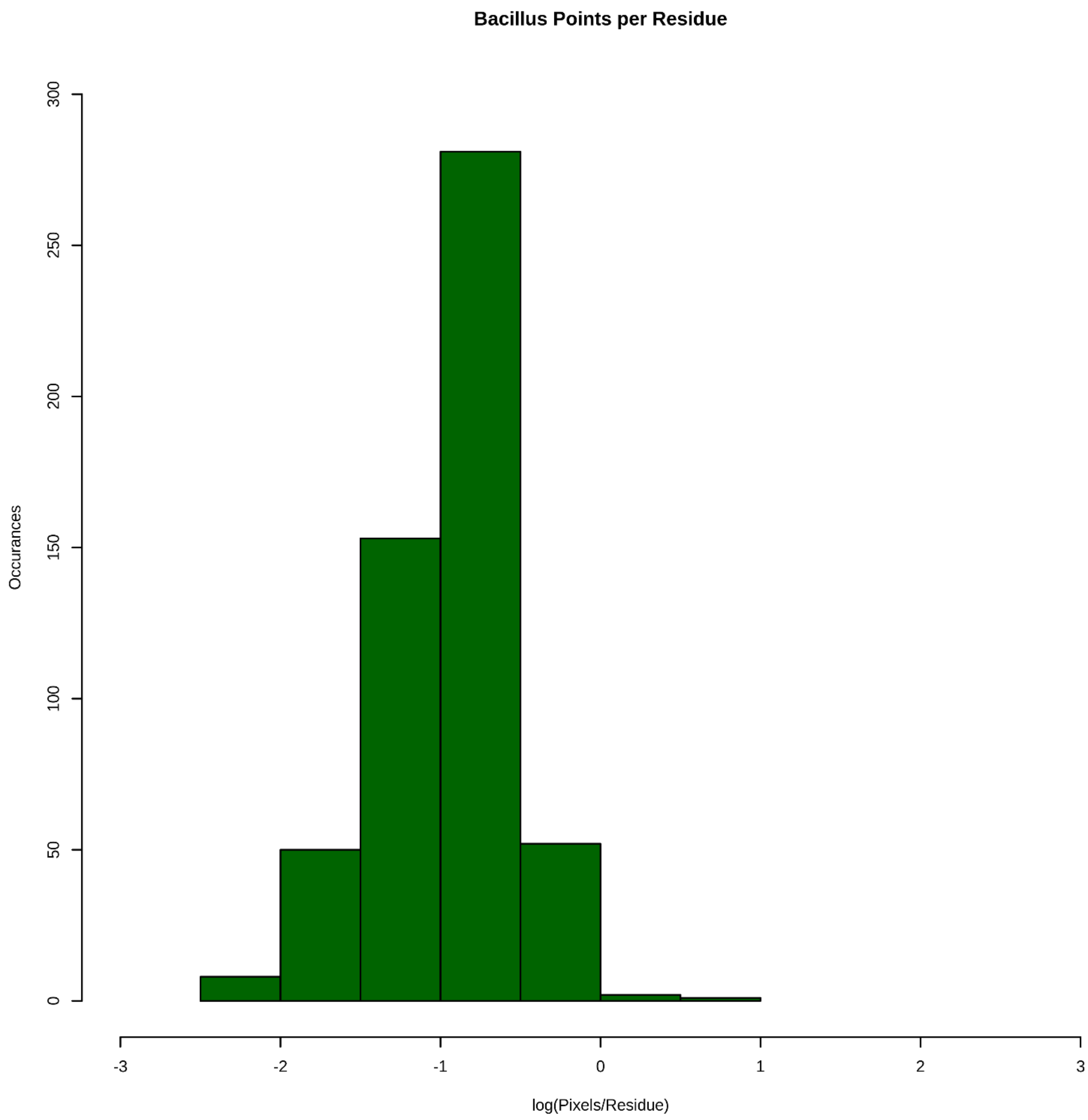

SI Fig. 2D:: Because other reports have noticed differences between natural and artificially designed repeat proteins (Espada, et al., 2015; Jorda, et al., 2010), we examined the information content in sets of natural and designed repeat proteins. RepeatsDB was queried using its own search function for entries with the word "design" or "engineer". (n=233). All of these are engineered repeat proteins.

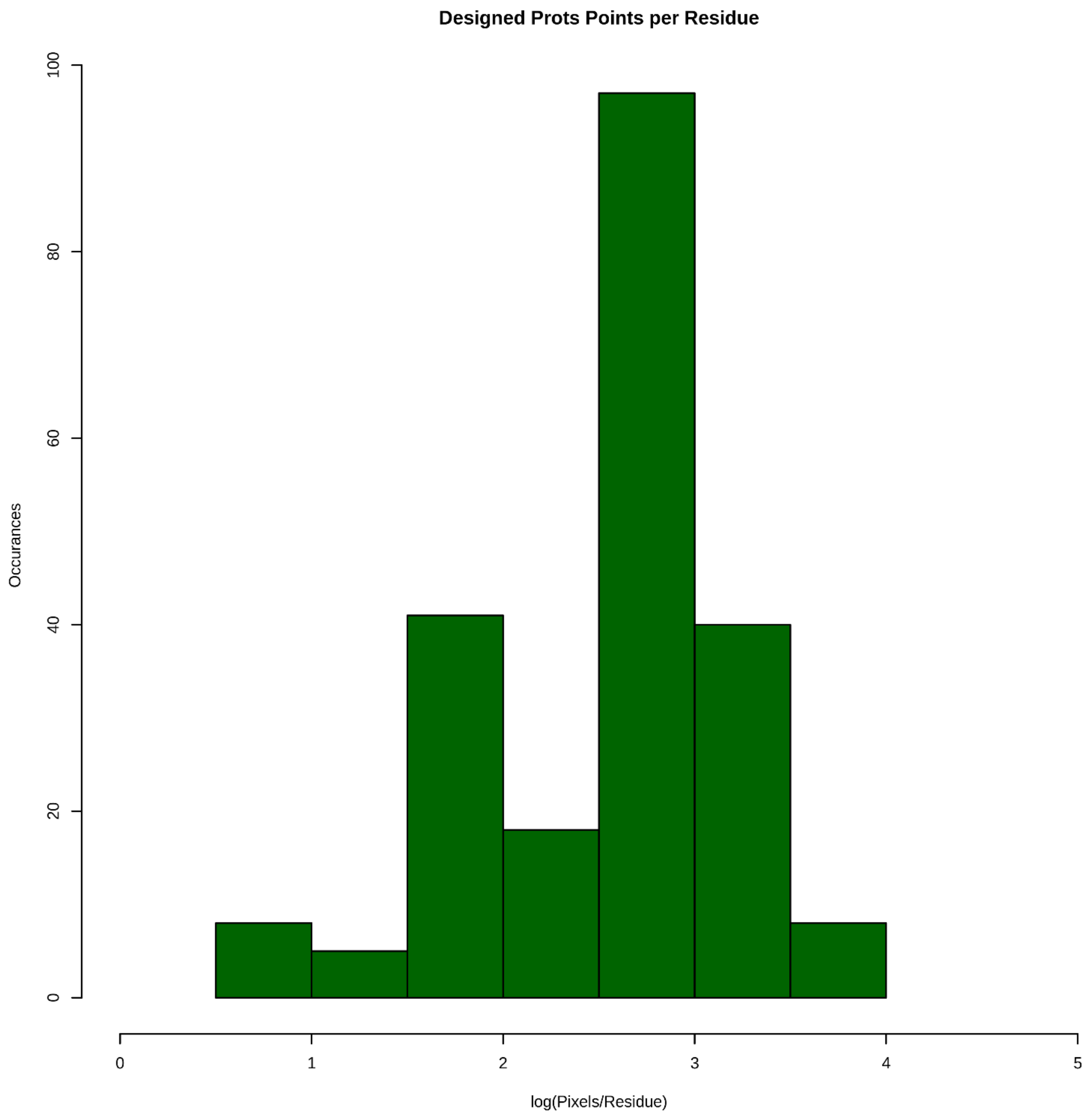

SI Fig. 3: Full version of Fig. 3 with explicit key included identifying all the protein traces (by RepeatsDB class).

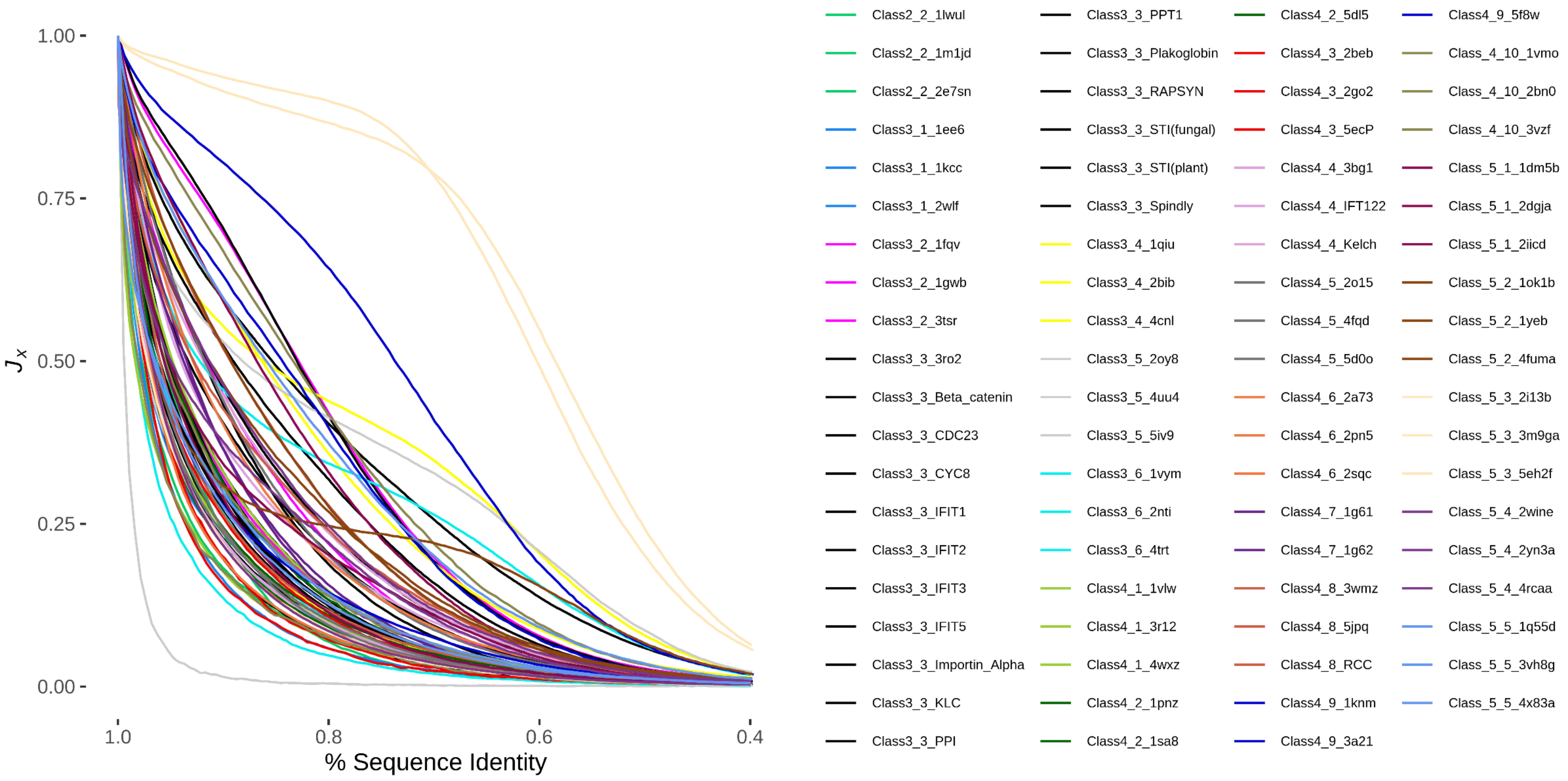

SI Fig. 4: Heatmap detailing the relationship between sequence identity and $\mathbf{J_X}$ in the RepeatsDB set. Note the Z-axis log scale.

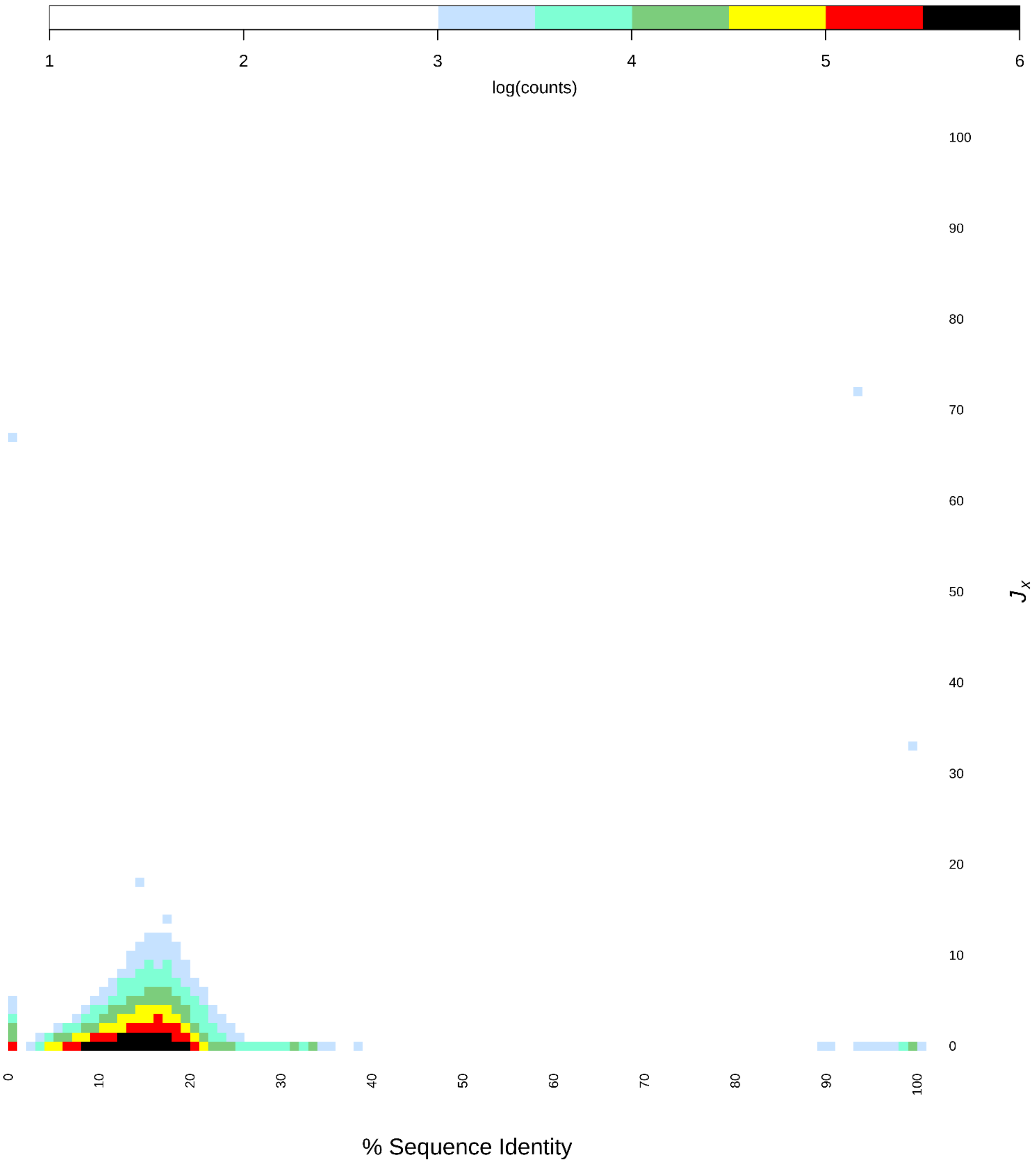

SI Fig. 5: The collected DOTTER plots of the proteins from standard repeat set (left center) and their permuted mutants (right center). On the left of each pair is the RepeatsDB class number and name of the standard protein along with its PDB ID (or species) listed in parentheses. To the right is listed the J_X value

**Protein**		J_X
**2.2** Chicken fibrinogen (1m1j_D)	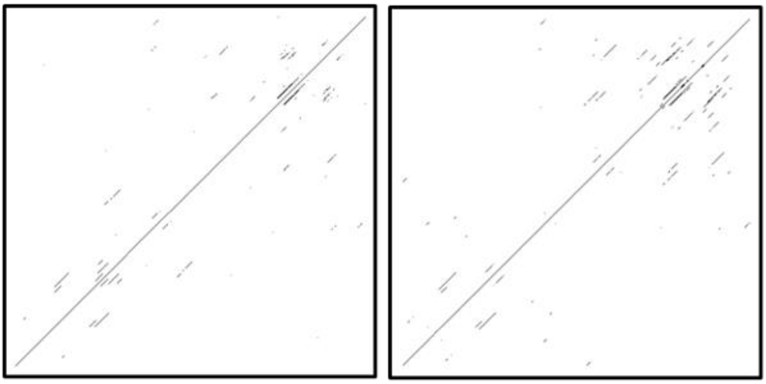	0.331
**2.2** Lamprey fibrinogen (1lwu_L)	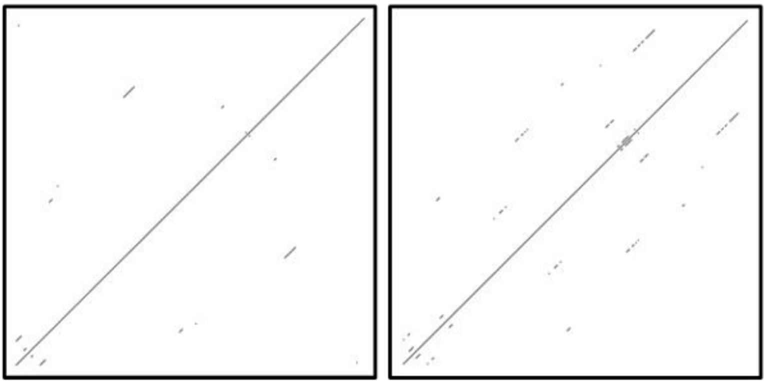	0.092
**2.2** Rab GNEF SEC2 (2e7s_N)	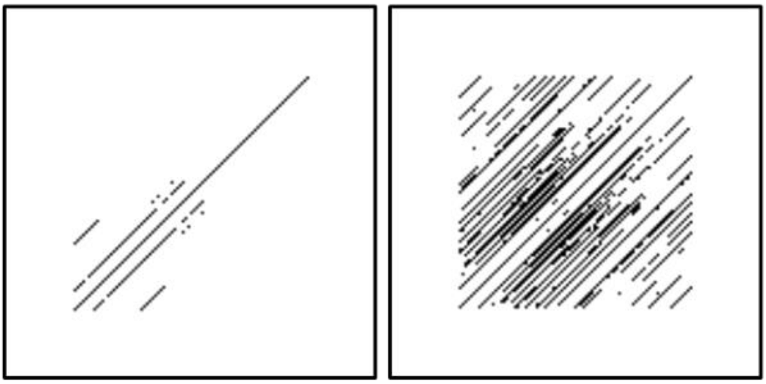	0.062
**3.1** Endopoly-galact-uronase I (1kcc_A)	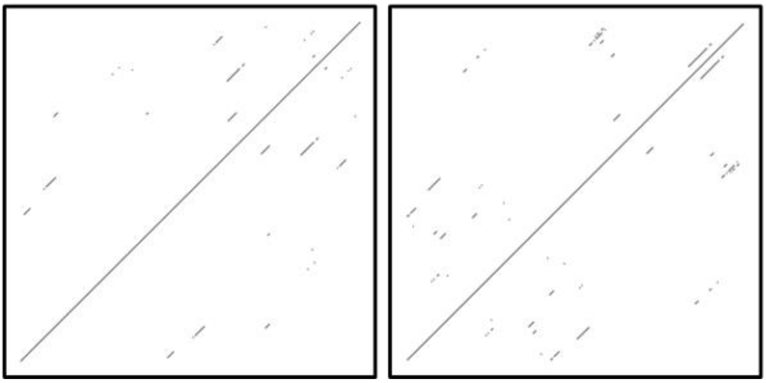	0.205

**Protein**		J_X
**3.1** Pectate lyase (1ee6_A)	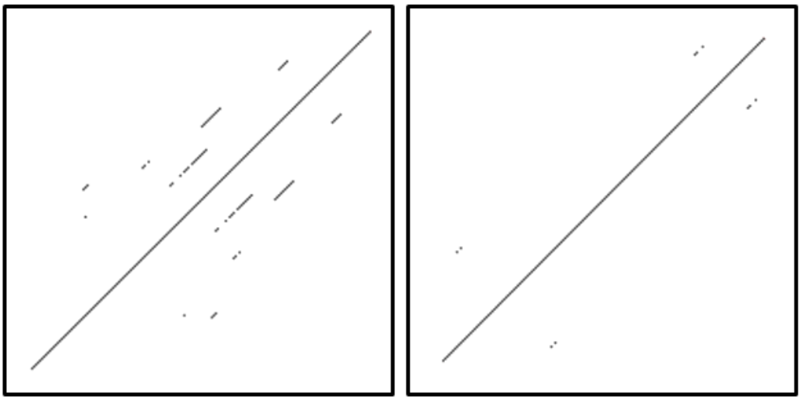	0.079
**3.1** Polysialic acid O-acetyl-transferase (2wlf_A)	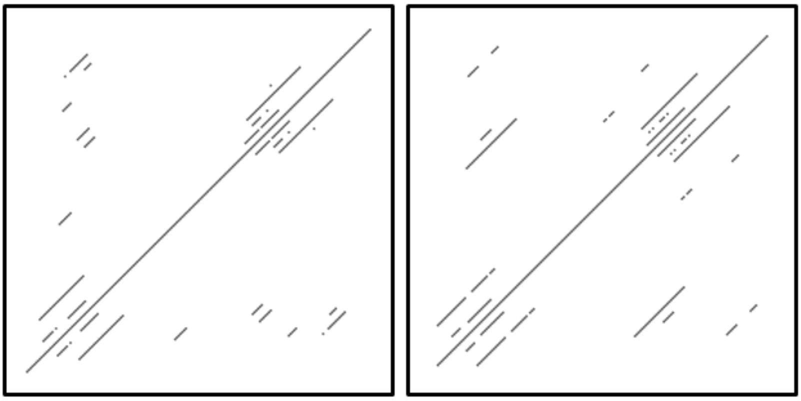	0.508
**3.2** Mouse ribonuclease inhibitor (3tsr_F)	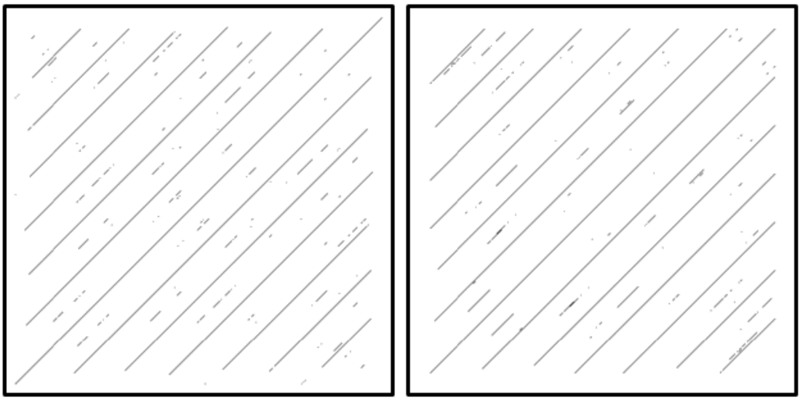	0.763
**3.2** Platelet glycoprotein 1B (1gwb_A)	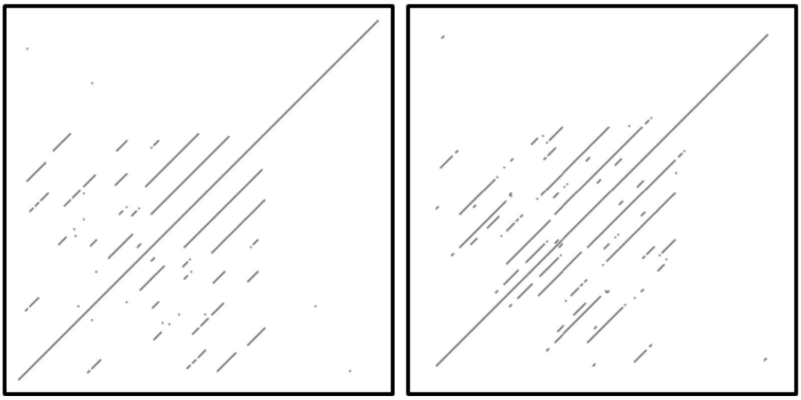	0.360

Protein		J_X
**3.2**<br>SKP2<br>(1fqv_A)	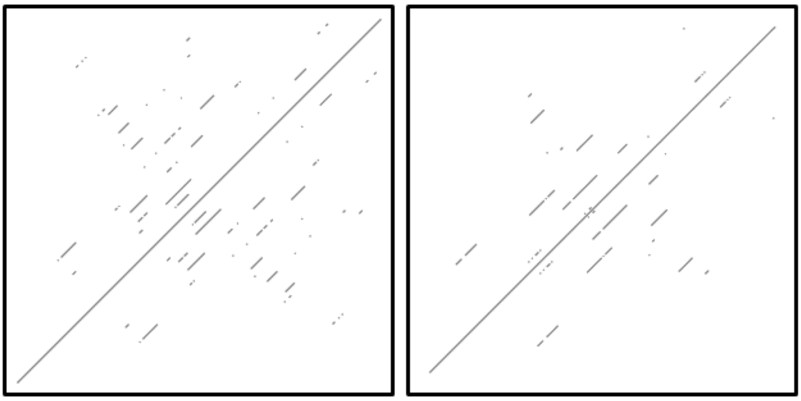	0.209
**3.3**<br>β-catenin<br>(Xenopus<br>laevis)	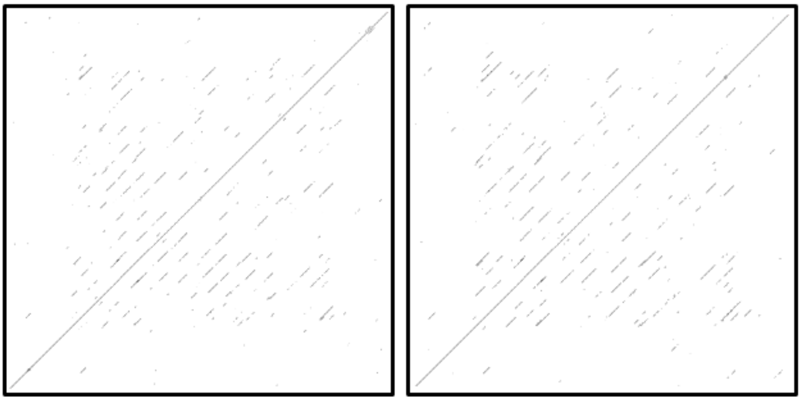	0.364
**3.3**<br>CDC23<br>(Bos taurus)	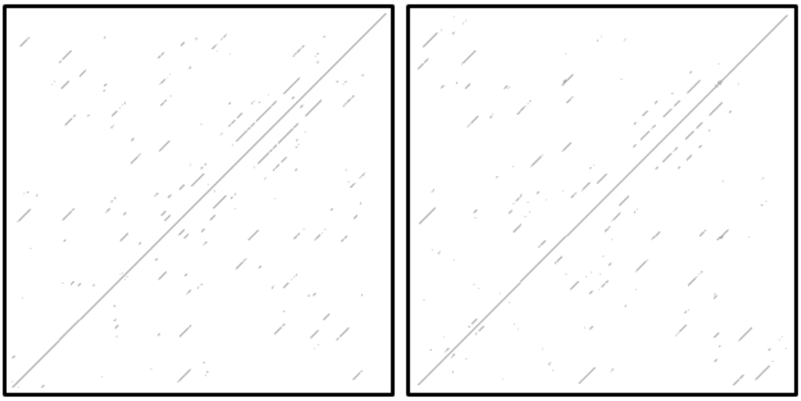	0.339
**3.3**<br>CYC8<br>(Saccharo-<br>myces<br>kudriavzevii)	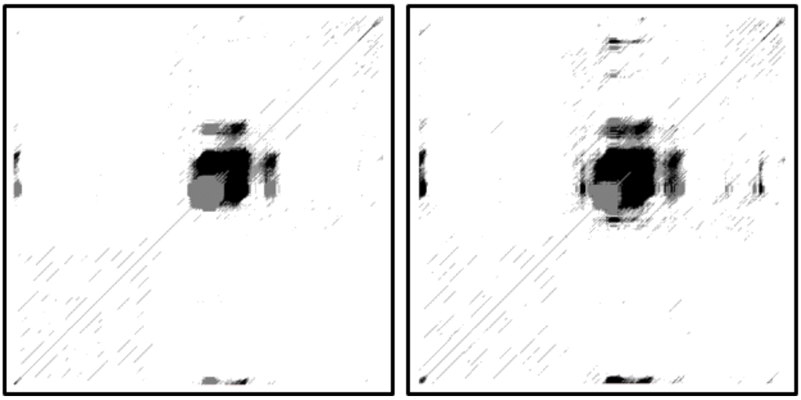	0.590

**Protein**		J_X
**3.3** G-protein signal modulator 2 (3ro2_A)	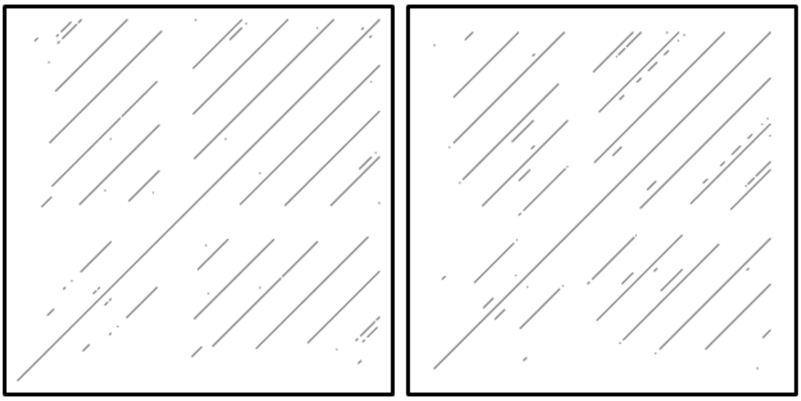	0.721
**3.3** Heat shock protein STI (Zea mays)	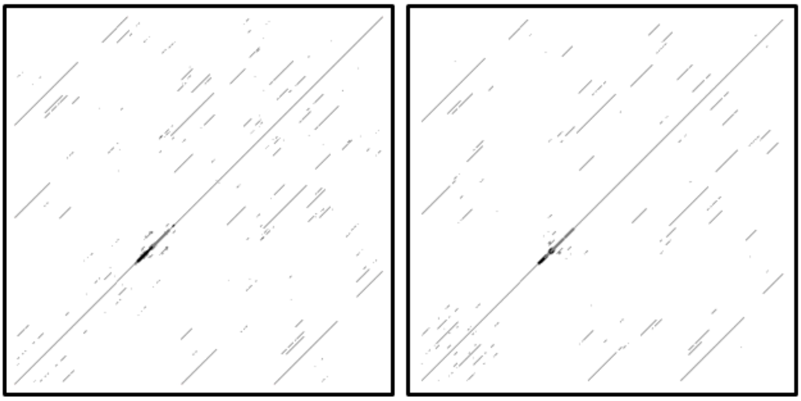	0.427
**3.3** Heat shock protein STI1 (Wallemia ichthyo-phaga)	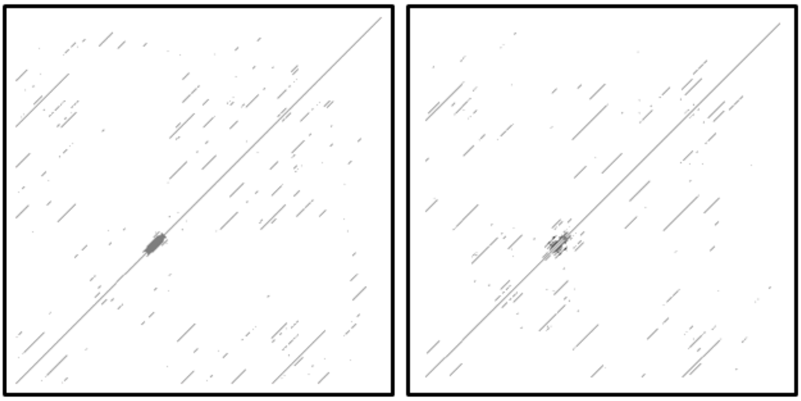	0.300
**3.3** IFIT1 (Homo sapiens)	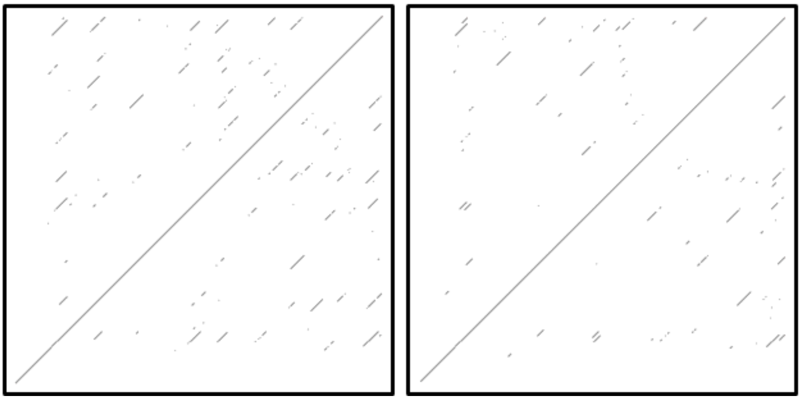	0.295

Protein		J_X
**3.3** IFIT2 (Homo sapiens)	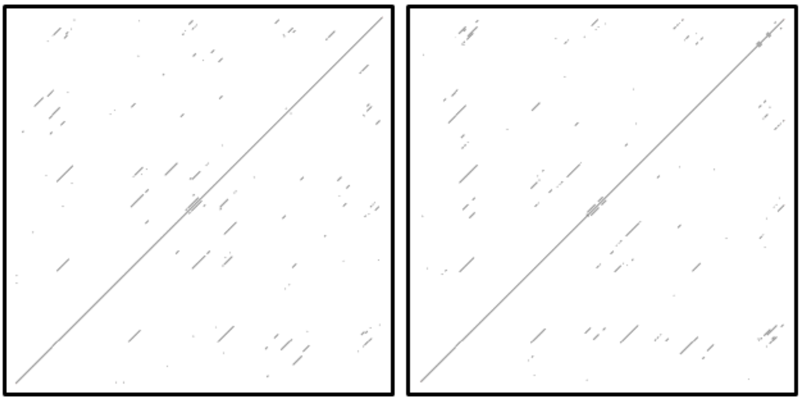	0.321
**3.3** IFIT3 (Homo sapiens)	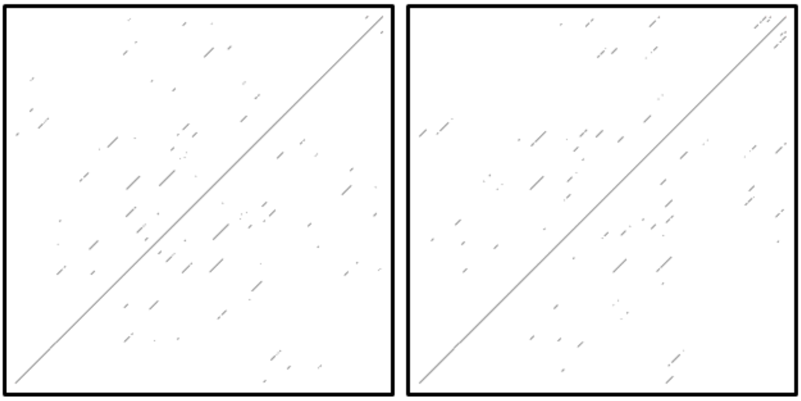	0.271
**3.3** IFIT5 (Homo sapiens)	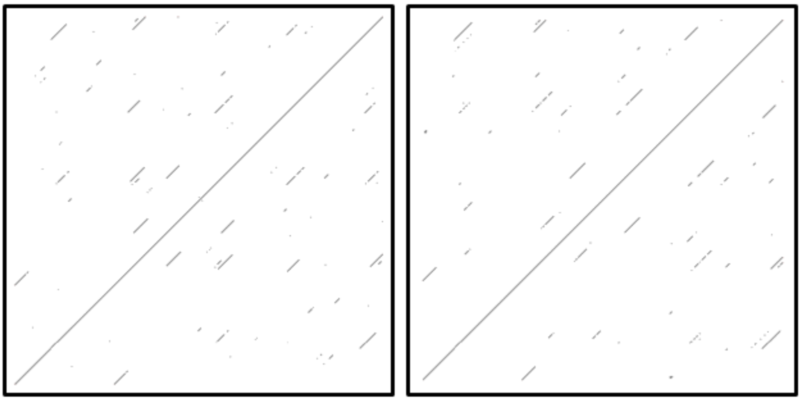	0.306
**3.3** Importin A (Cynara carduncululus)	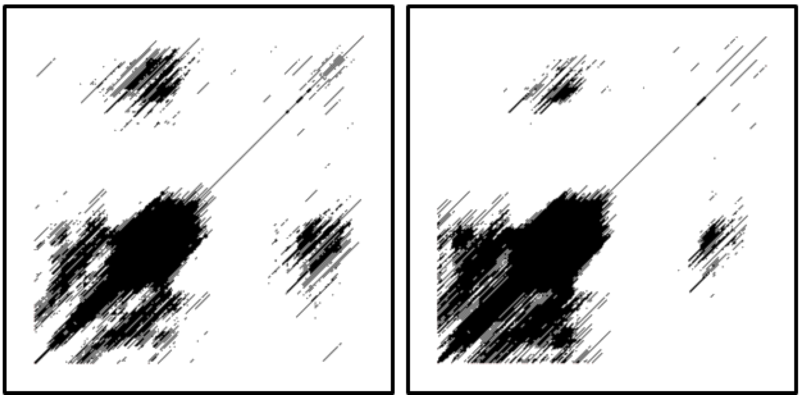	0.660

**Protein**			J_X
**3.3** Kinesin light chain (Petromyzon marinus)	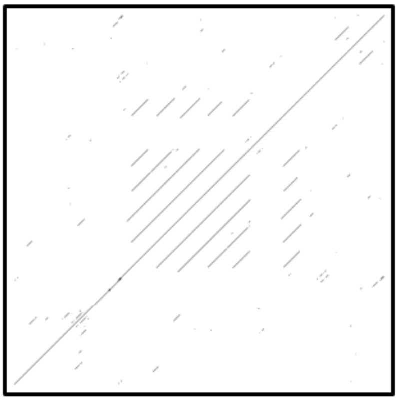	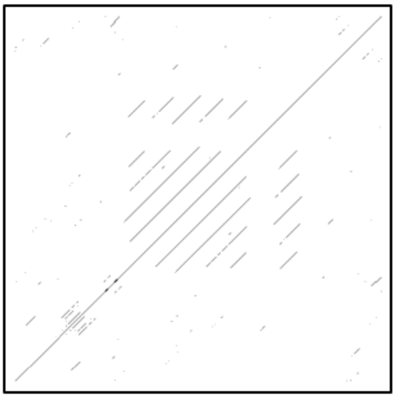	0.635
**3.3** Peptide N-acetyl-glucosaminyl-transferase/ Spindly (Selaginella moellen-dorffii)	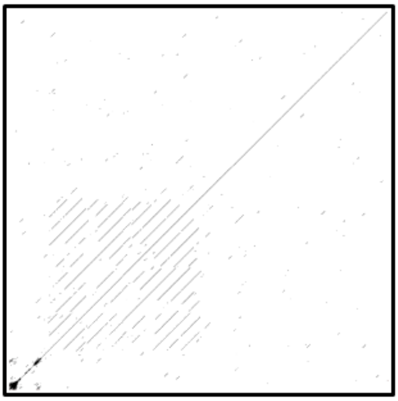	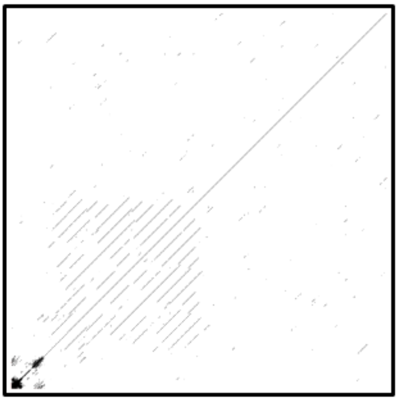	0.554
**3.3** Peptidyl-prolyl isomerase (Oryzias latipes)	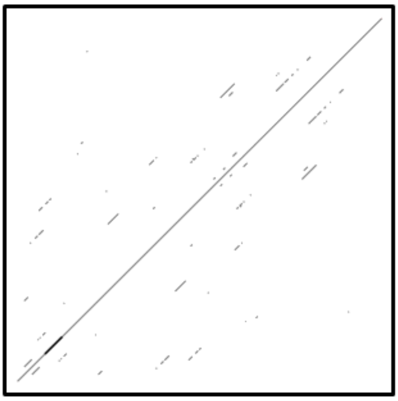	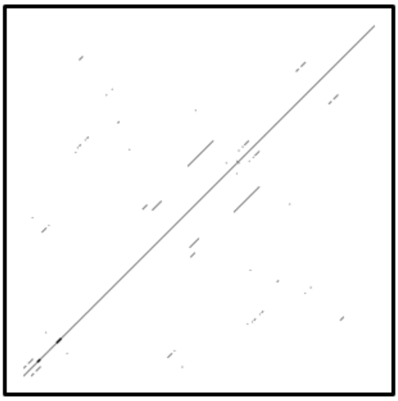	0.141
**3.3** Plakoglobin (Rattus norvegicus)	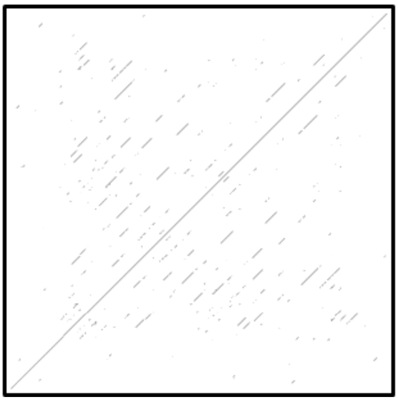	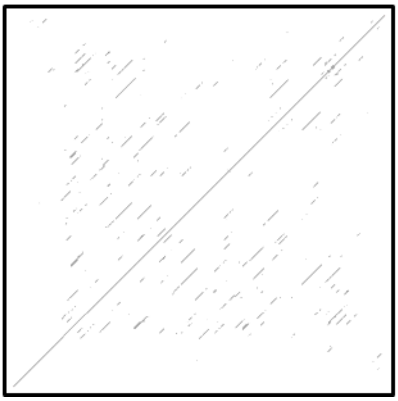	0.290

Protein		J_X
**3.3** RAPSYN (Camelus ferus)	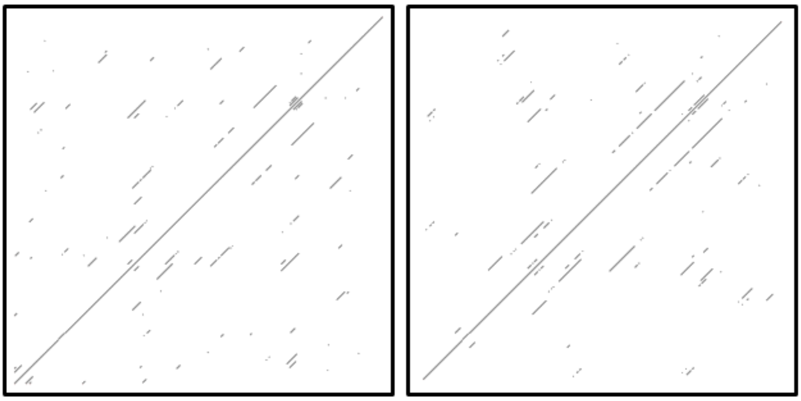	0.286
**3.3** Serine/ Threonine protein phosphatase (Saccharo-myces cerevisiae)	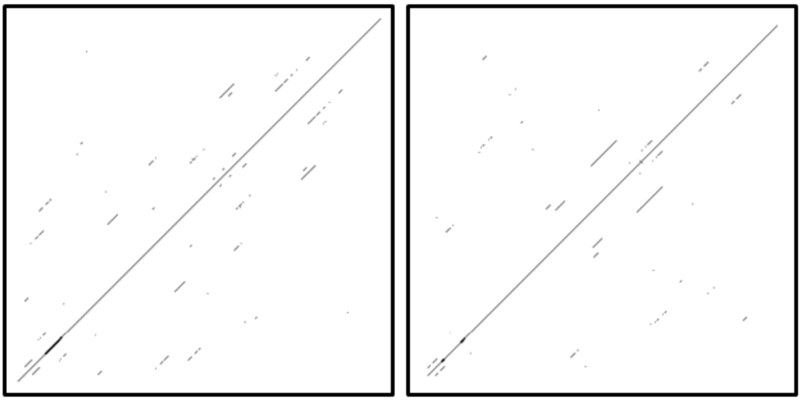	0.205
**3.4** Adenovirus fibre (1qiu_A)	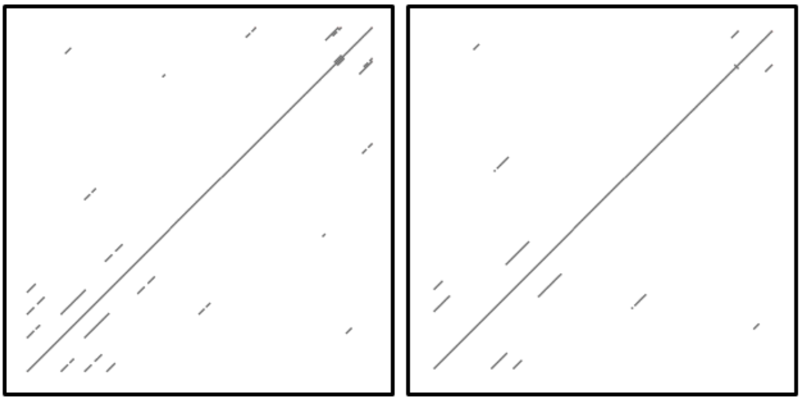	0.306
**3.4** Pneumo-coccal surface prot. (4cnl_A)	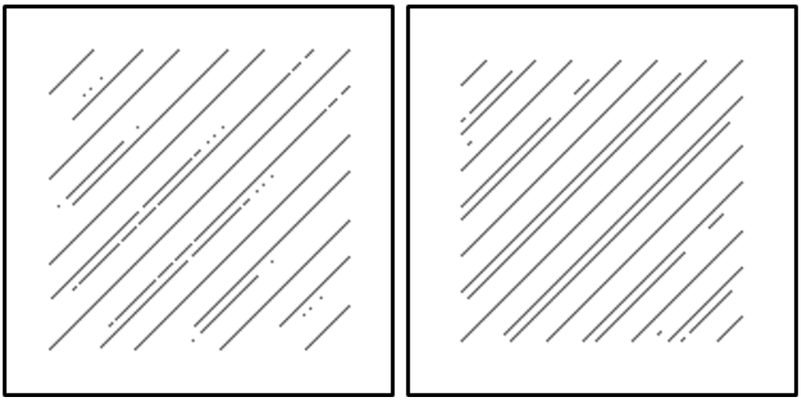	0.775

**Protein**		J_X
**3.4** Teichioic acid phosphoryl-choline esterase Pce (2bib_A)	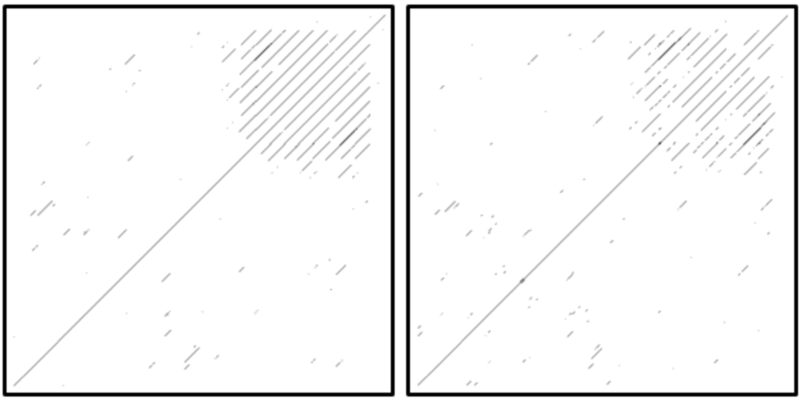	0.561
**3.5** LPS Transporter LptDE (5iv9_A)	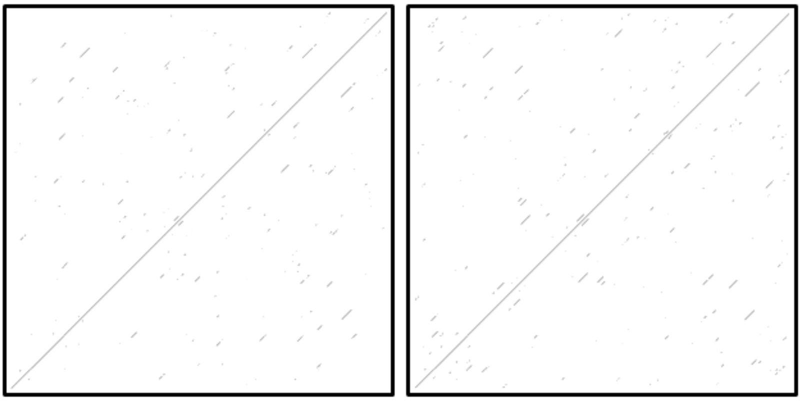	0.216
**3.5** Outer surface protein A (2oy8_A)	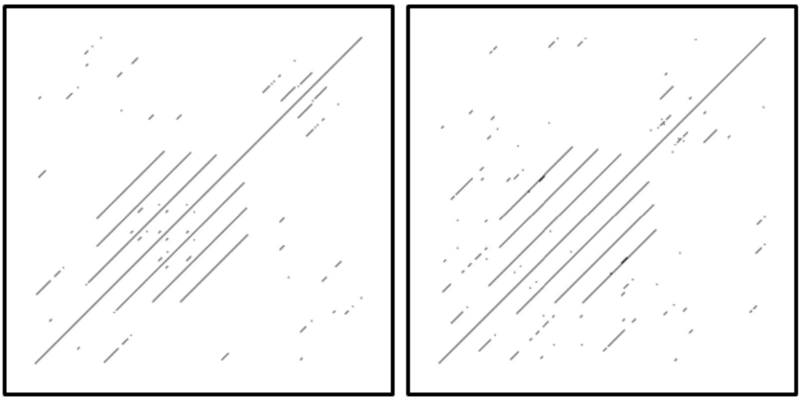	0.552
**3.5** Periplasmic lipopoly-saccharide transport prot. LPTH (4uu4_A)	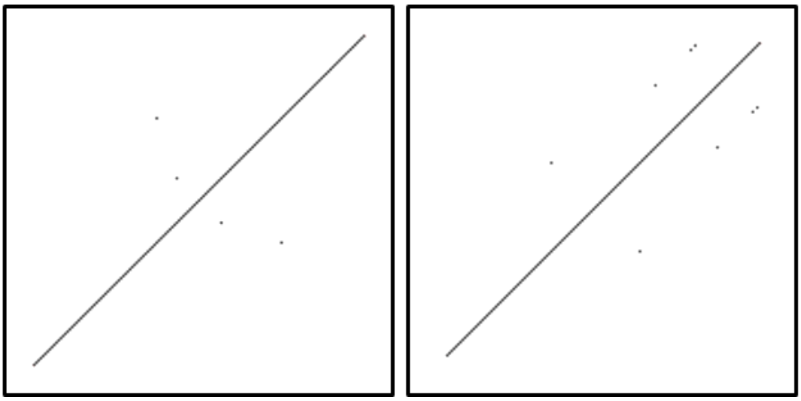	0.000

**Protein**			J_X
**3.6** DNA polymerase III (4trt_B)	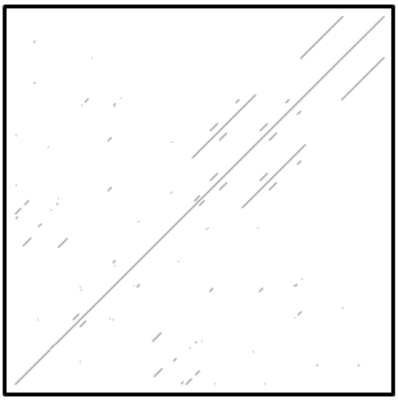	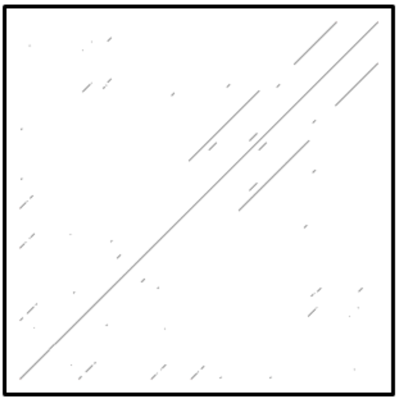	0.383
**3.6** DNA polymerase sliding clamp A (2nti_C)	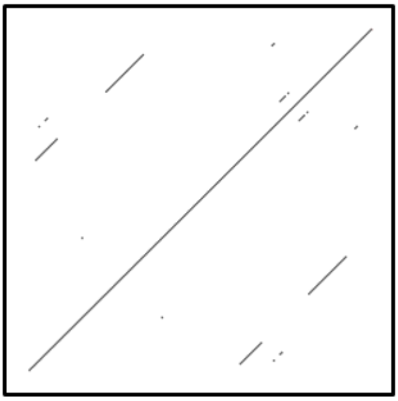	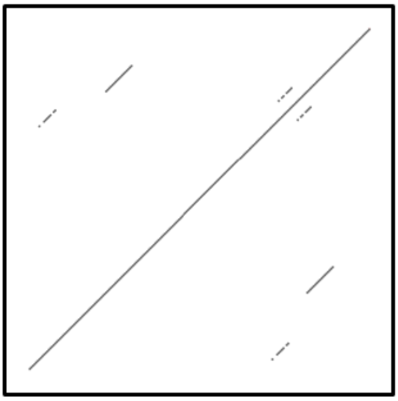	0.421
**3.6** Proliferating cell nuclear antigen (1vym_A)	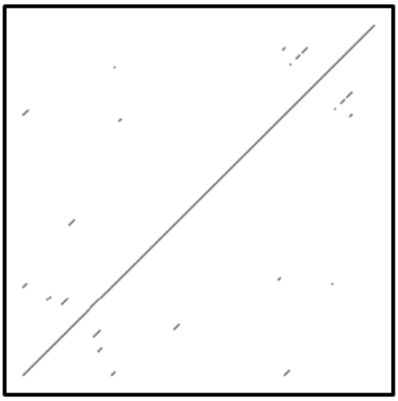	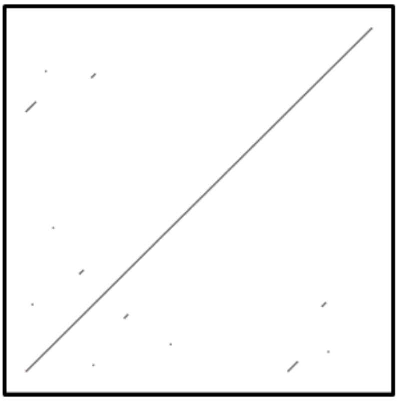	0.091
**4.1** 4-hydroxy-2-oxoglutarate aldolase (1vlwA)	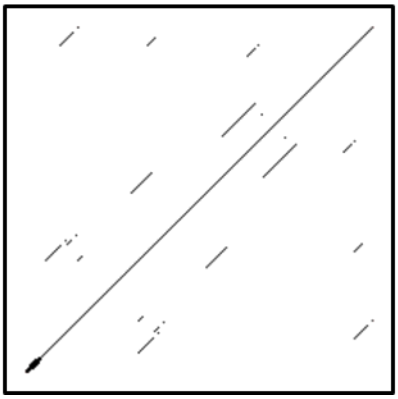	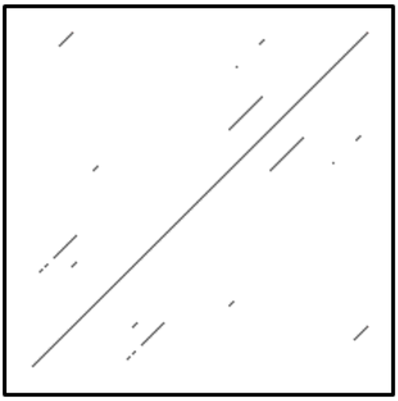	0.262

**Protein**		J_X
**4.1** Deoxyribose-phosphate aldolase (3r12_A)	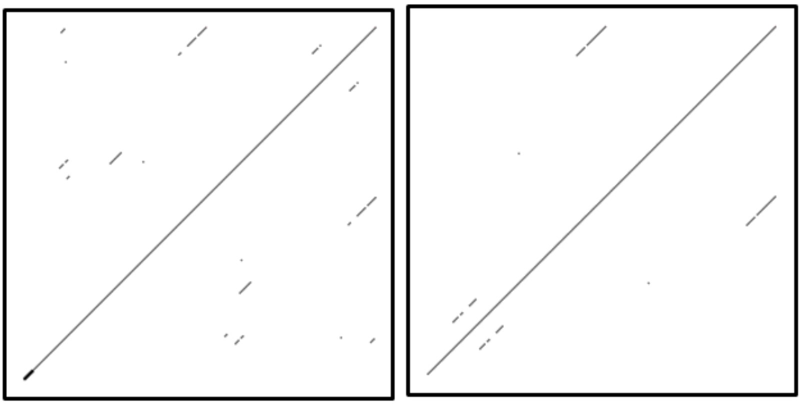	0.246
**4.1** Pyridoxal biosynthesis lyase (4wxz_A)	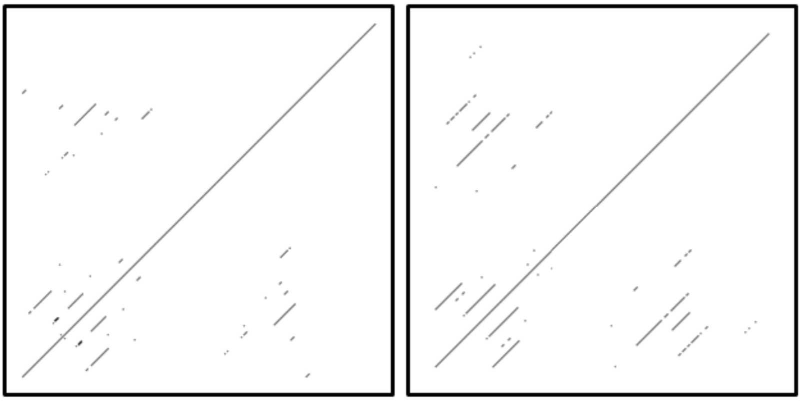	0.287
**4.2** Ferric citrate transporter FecA (1pnz_A)	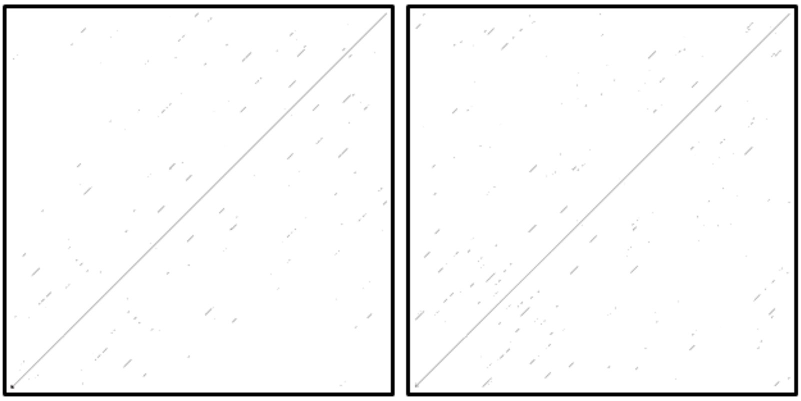	0.160
**4.2** Intestinal fatty acid-binding protein (1sa8_A)	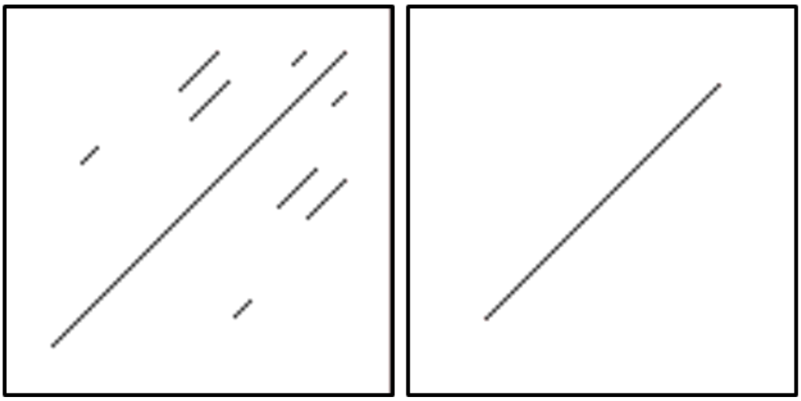	0.000

Protein			J_X
**4.2** Outer membrane permeability OccAB1 (5dl5_A)	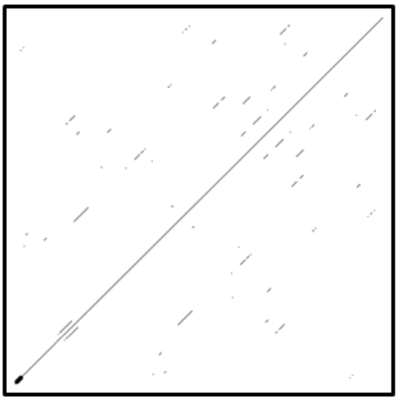	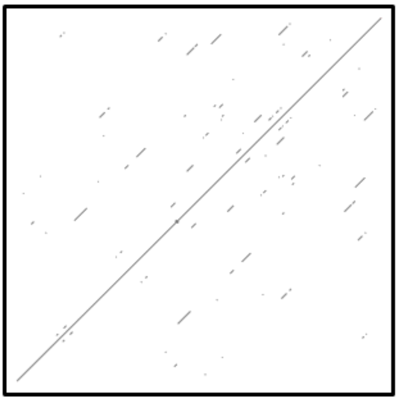	0.213
**4.3** Kunitz-type kallikrein inhibitor BbKI (2go2_A)	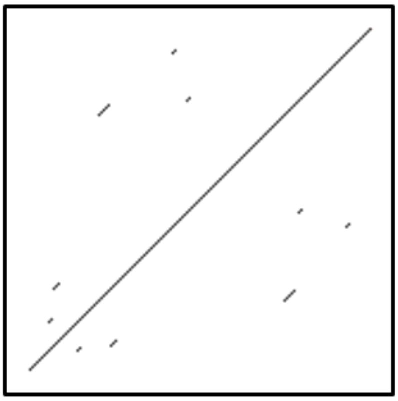	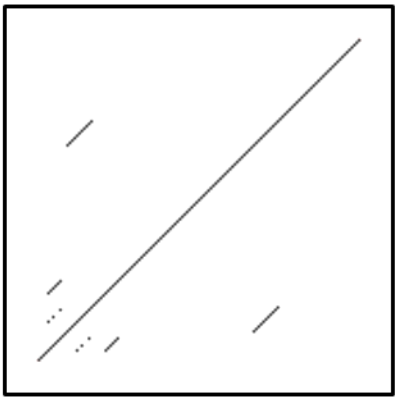	0.172
**4.3** Lysenin pore (5ec5_P)	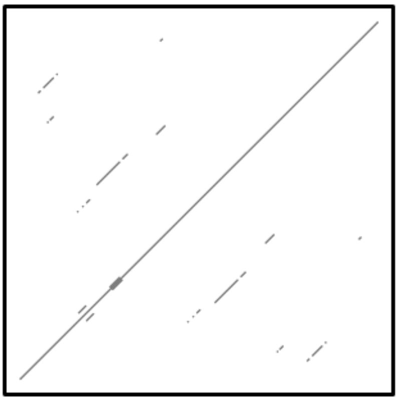	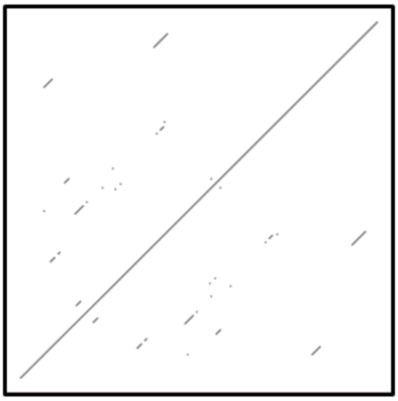	0.172
**4.3** Winged bean chymo-trypsin inhibitor (2beb_A)	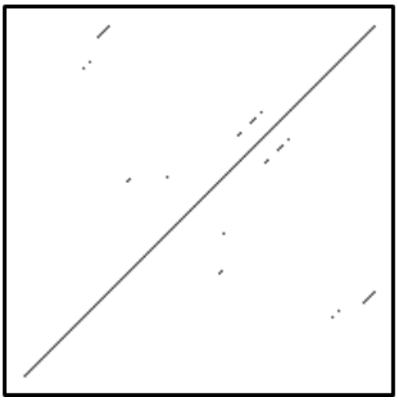	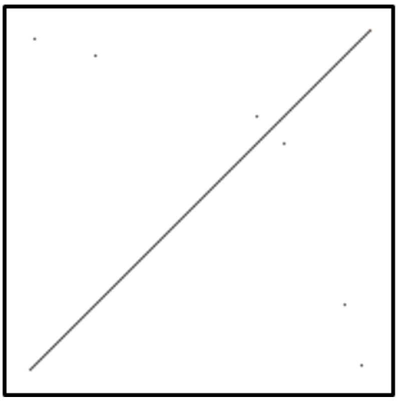	0.111

**Protein**			J_X
**4.4** Coat for the nuclear pore (3bg1_D)	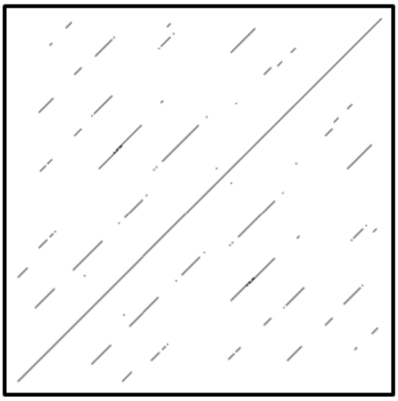	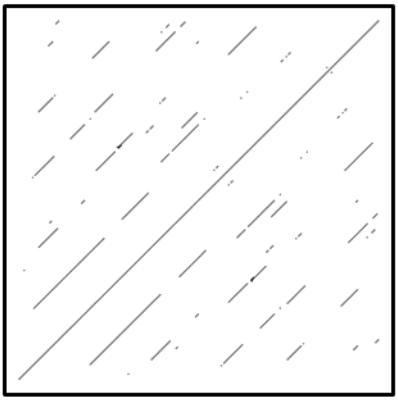	0.559
**4.4** Intraflagellar transport protein 122 (Macaca mulatta)	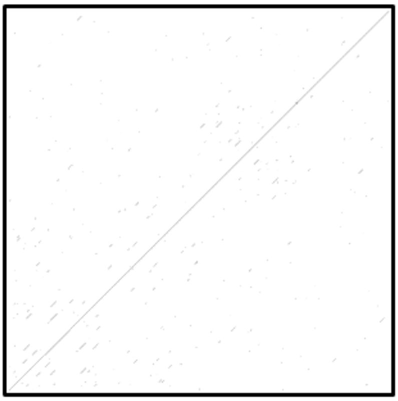	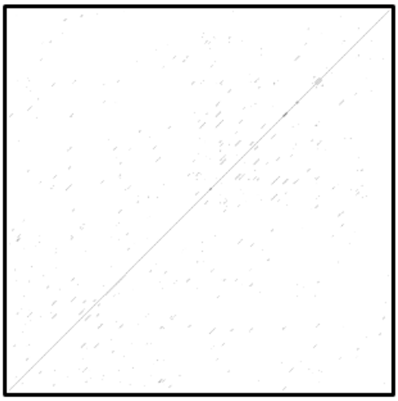	0.226
**4.4** Ring canal kelch protein (Drosophila melano-gaster)	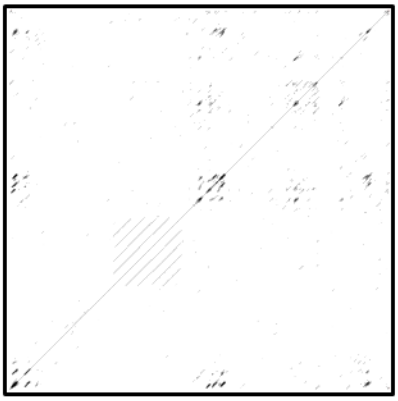	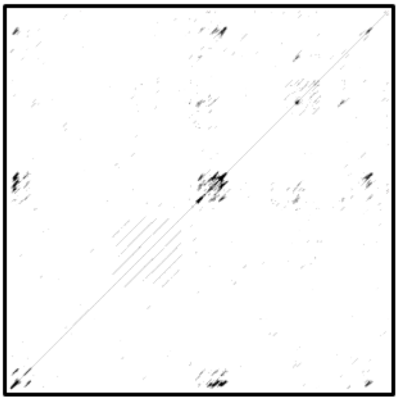	0.358
**4.5** 3-phospho-shikimate 1-carboxyvinyl-transferase (2o15_A)	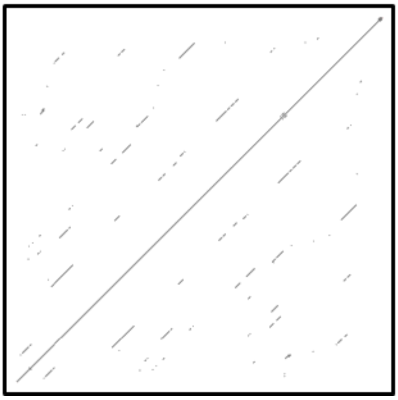	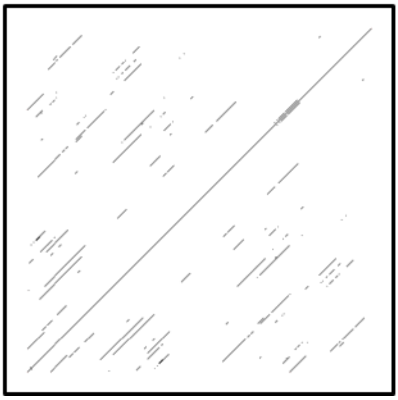	0.184

**Protein**		J_X
**4.5** BamABCDE complex (5d0_O)	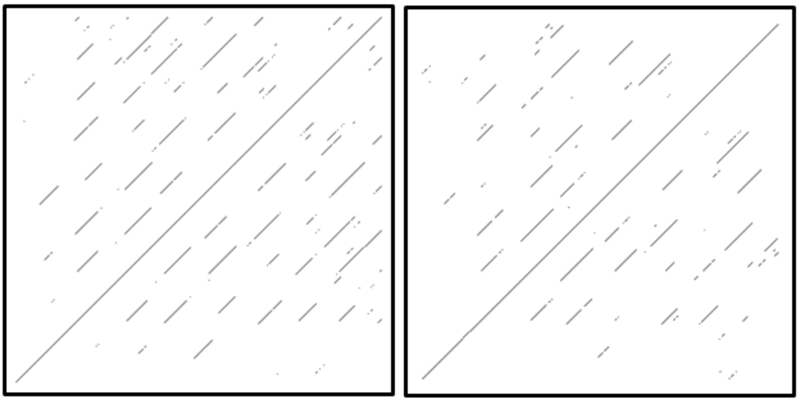	0.485
**4.5** Enolpyruvyl transferase NikO (4fqd_B)	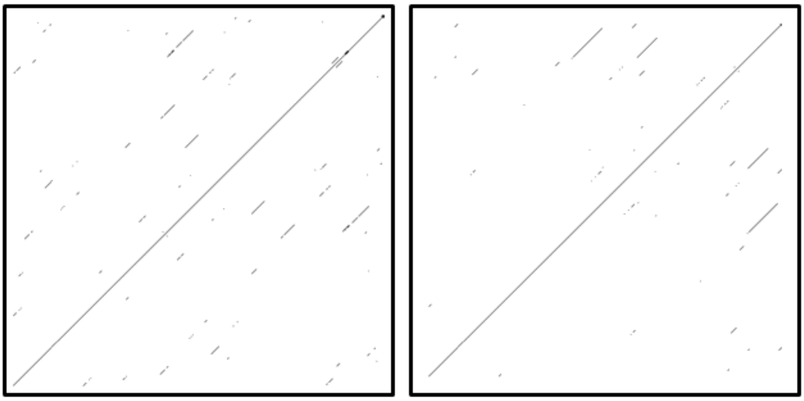	0.125
**4.6** Human complement component C3 (2a73_A)	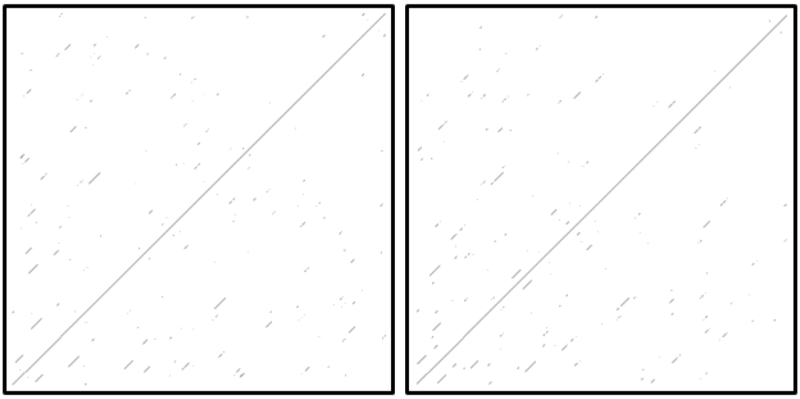	0.175
**4.6** Squalene-hoplene cyclase (2sqc_A)	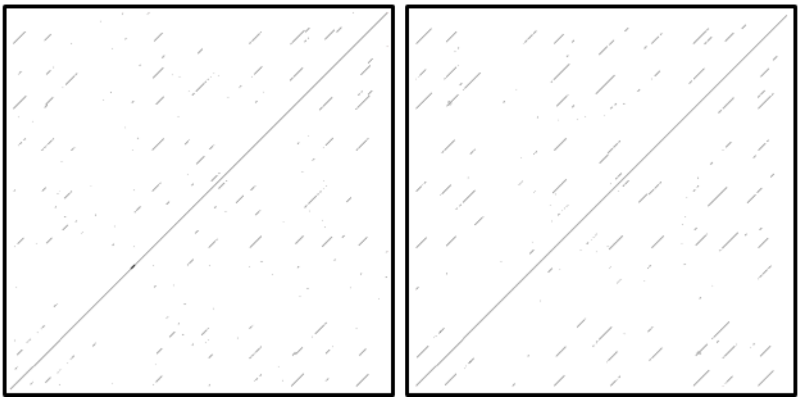	0.408

Protein		J_X
**4.6** Thioester-containing protein I (2pn5_A)	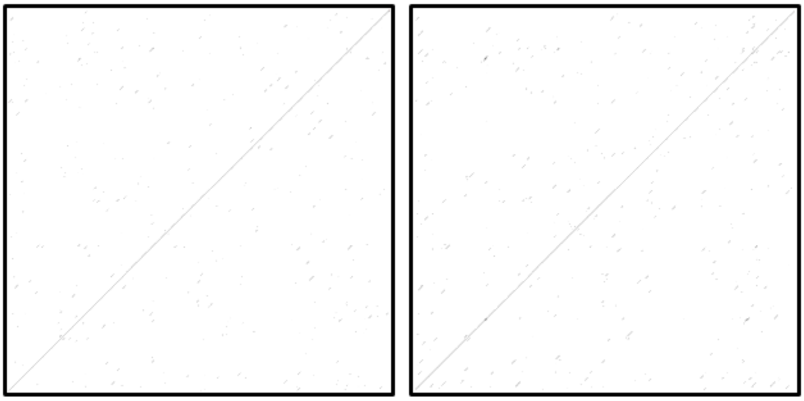	0.171
**4.7** Ribosome anti-association factor IF6, eukaryotic (1g62_A)	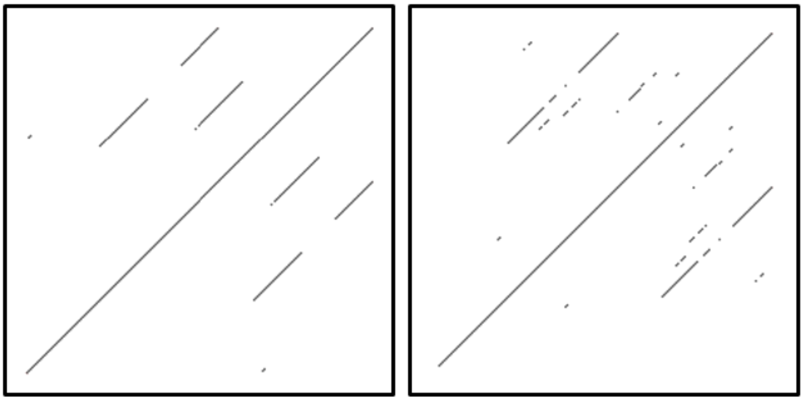	0.464
**4.7** Ribosome anti-association factor IF6, prokaryotic (1g61_B)	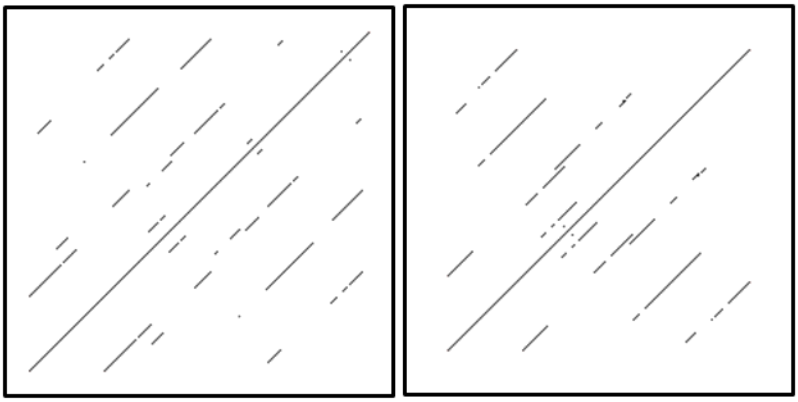	0.305
**4.8** 90S pre-ribosome (5jpq_A)	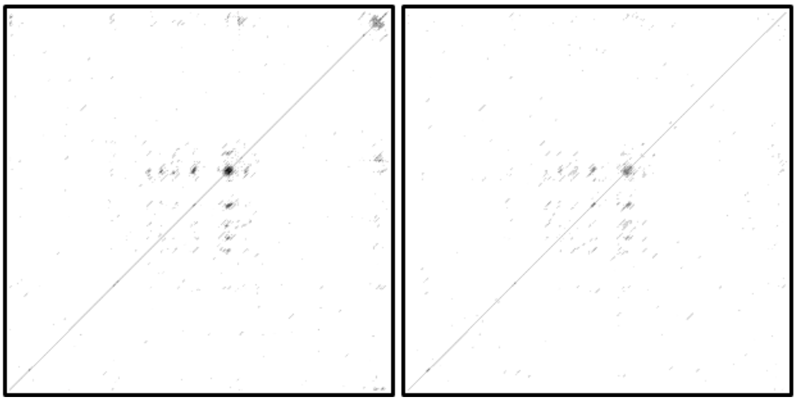	0.258

Protein		J_X
**4.8**<br>a-L-arabino-furanosidase (3wmz_A)	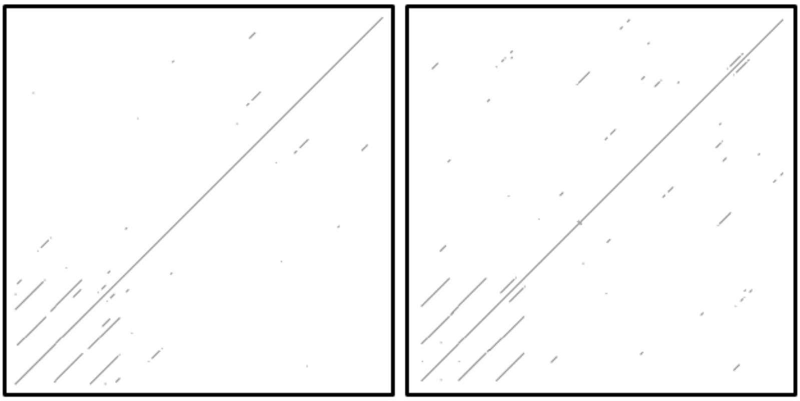	0.382
**4.8**<br>Regulator of chromosome condensa-tion/RCC (Heterocephal us glaber)	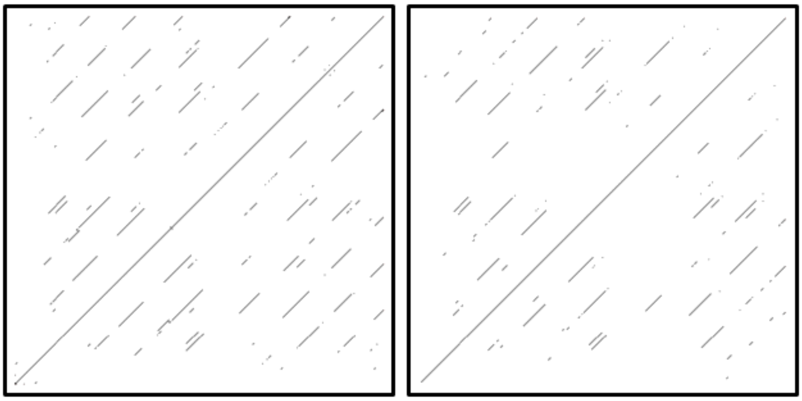	0.522
**4.9**<br>b-L-Arabinopyran -osidase (3a21_B)	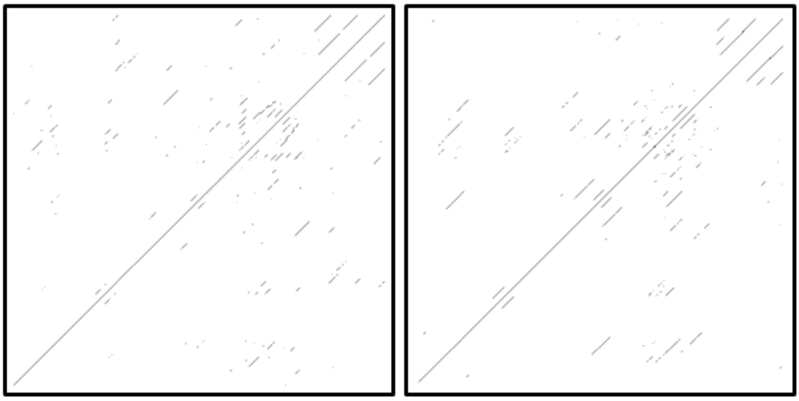	0.198
**4.9**<br>Endo-1,4-beta-xylanase A (1knm_A)	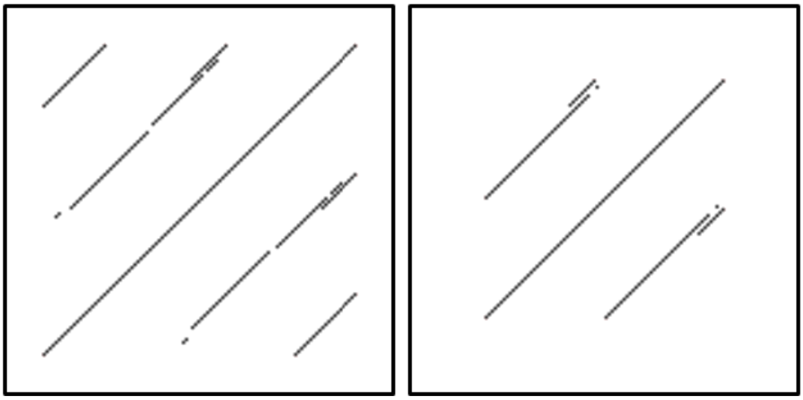	0.257

**Protein**		J_X
**4.9** GalNAc/Gal-specific lectin (5f8w_A)	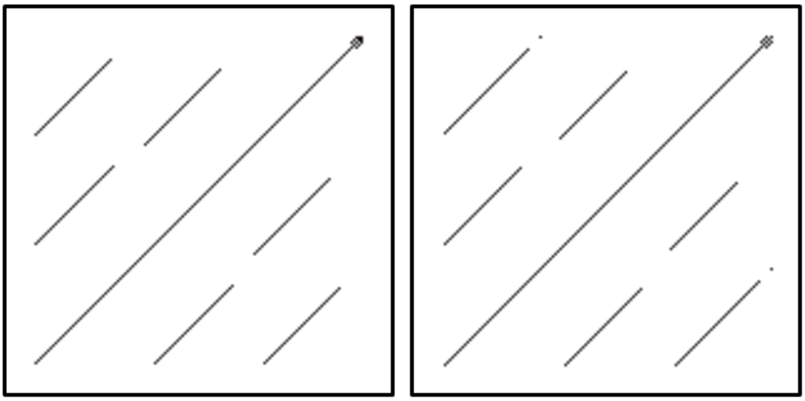	0.883
**4.10** Pancreatic secretory protein ZG16p (3vzf_A)	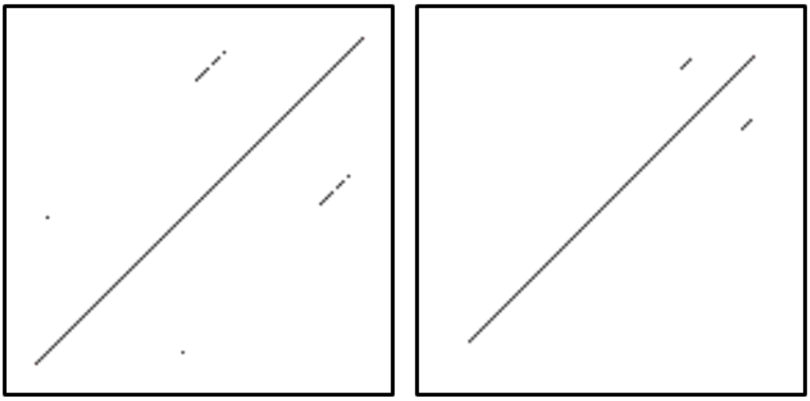	0.000
**4.10** Ripening-associated protein (2bn0_A)	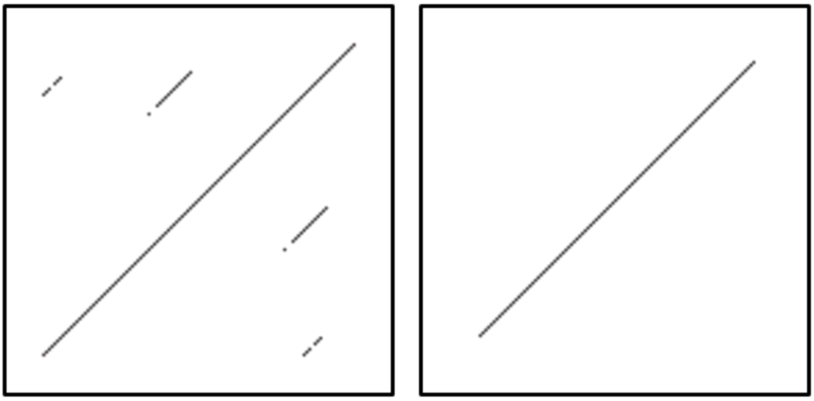	0.000
**4.10** Vitelline membrane outer layer prot. 1 (1vmo_A)	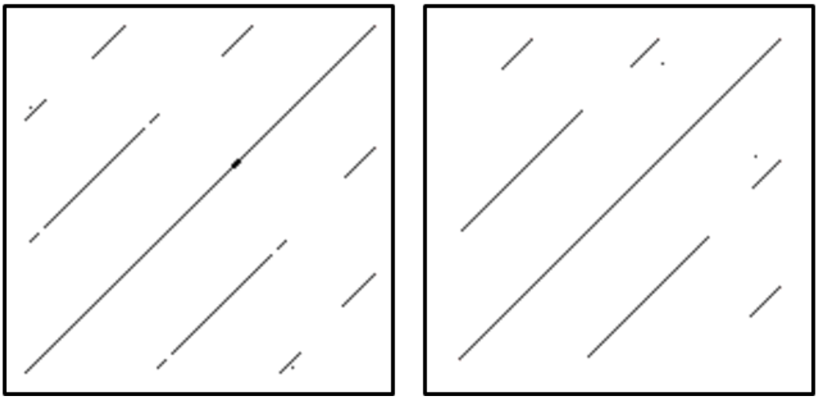	0.687

Protein		J_X
**5.1** Alpha-11 giardin (2iic_D)	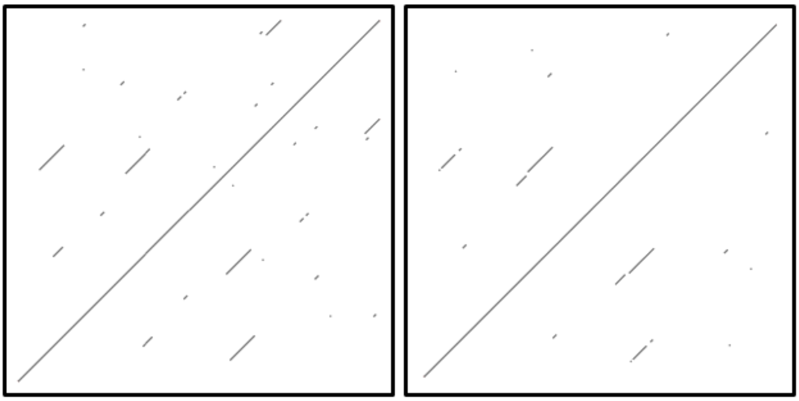	0.354
**5.1** Annexin XII (1dm5_B)	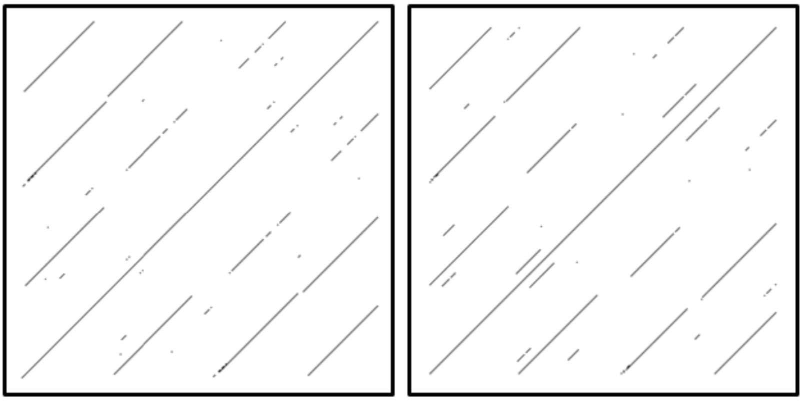	0.639
**5.1** Hypothetical protein ebhA (2dgj_A)	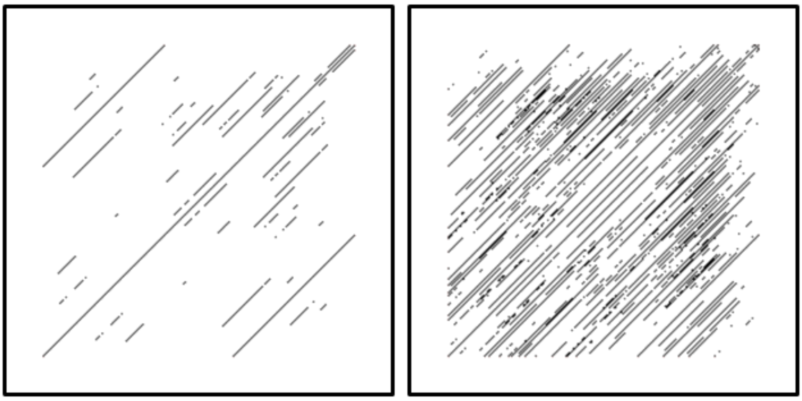	0.127
**5.2** Accumulation associated protein (4fum_A)	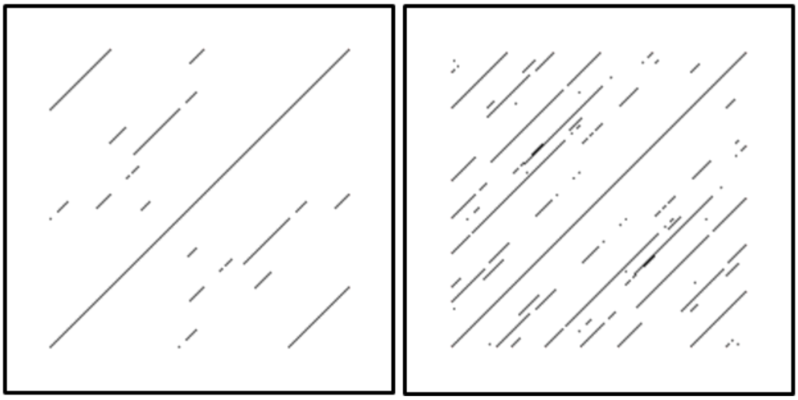	0.265

Protein			J_X
**5.2** CD55 decay accelerating factor (1ok1_B)	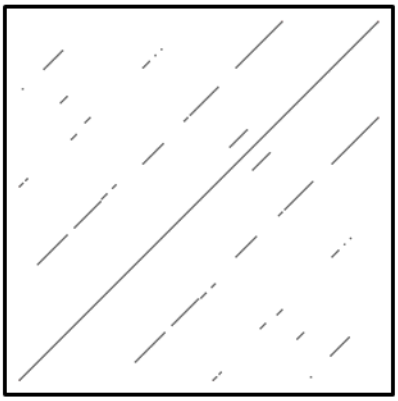	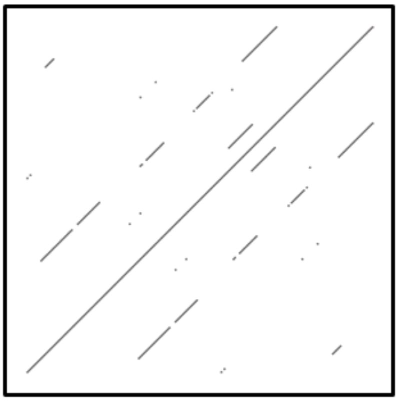	0.559
**5.2** Complement control protein (1y8e_B)	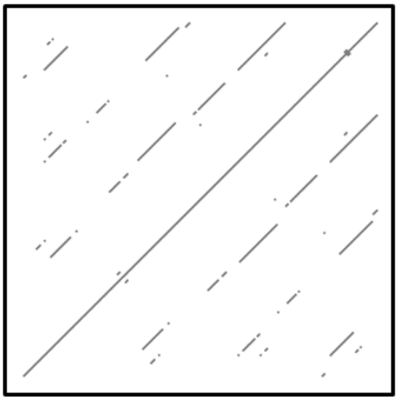	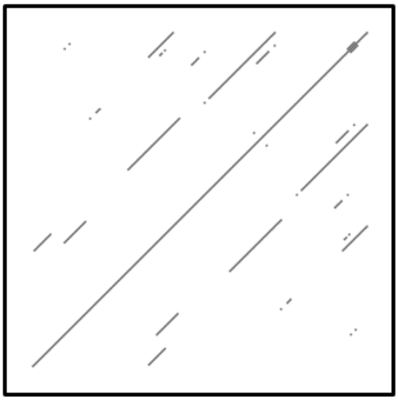	0.406
**5.3** Aart, designed six finger zinc finger (2i13_B)	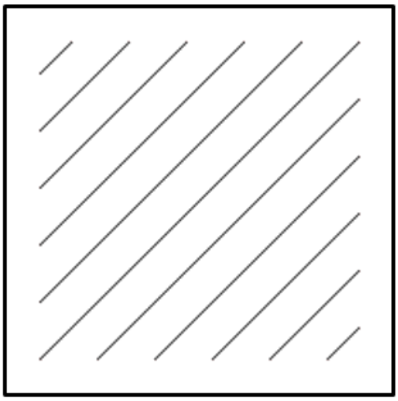	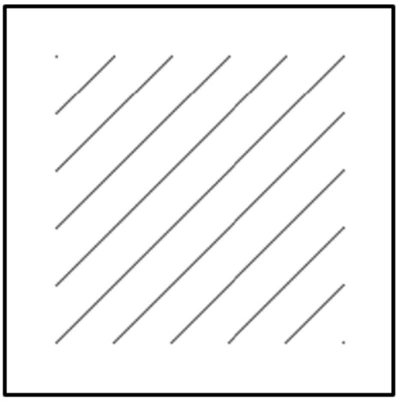	0.792
**5.3** Histone-lysine N-methyltrans-ferase PRDM9 (5eh2_F)	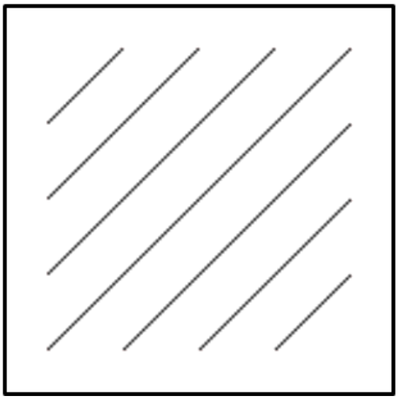	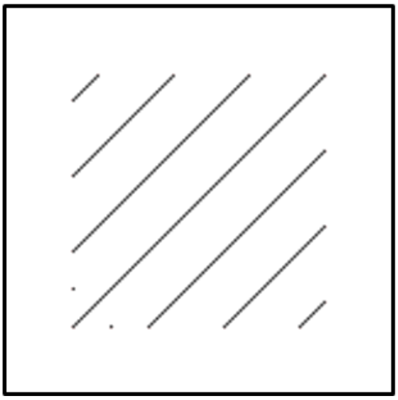	0.675

Protein		J_X
**5.3** Ser/Thr kinase from Staphylo-coccus aureus (3m9g_A)	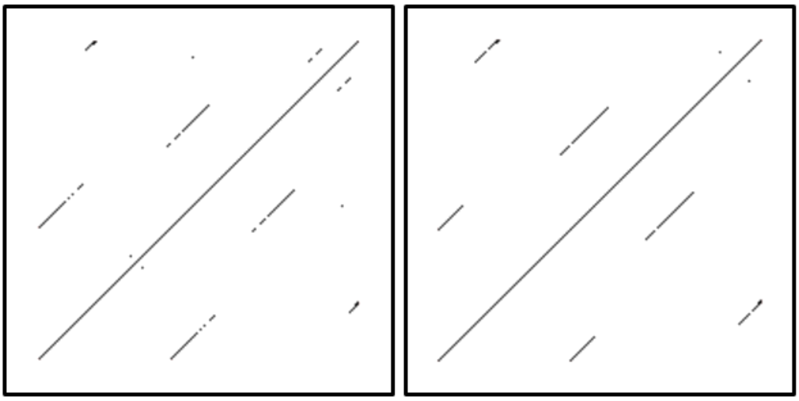	0.561
**5.4** C3 convertase (2win_E)	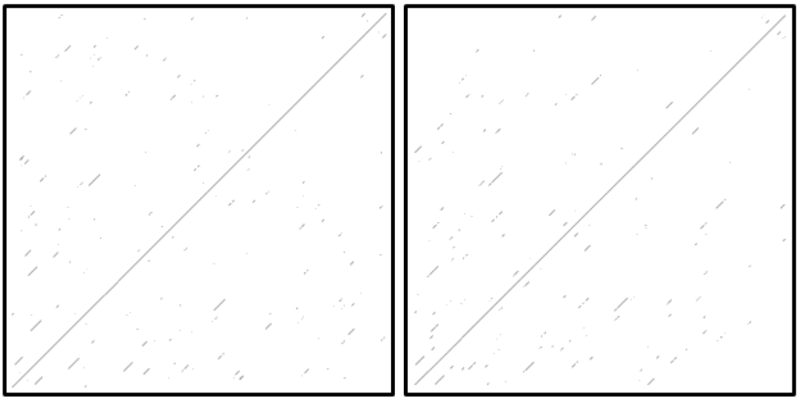	0.170
**5.4** Calcium-binding adhesin SiiE (2yn3_A)	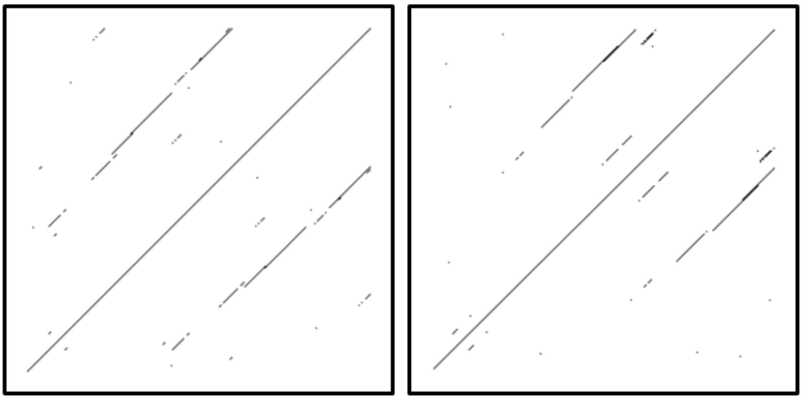	0.315
**5.4** Receptor-type tyrosine-protein phosphatase delta (4rca_A)	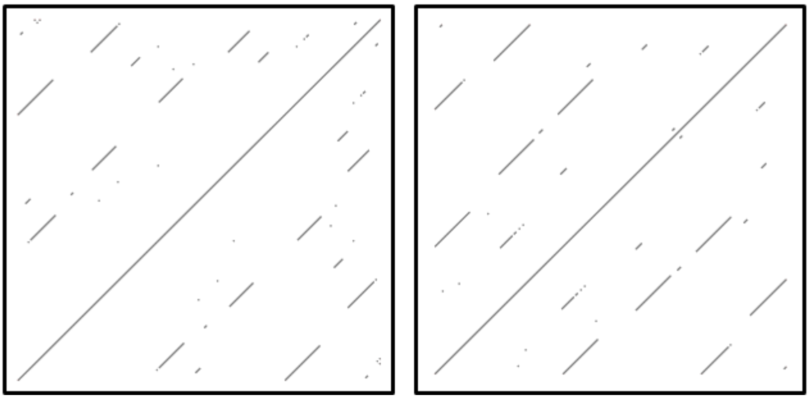	0.462

**Protein**		J_X
**5.5** Down syndrome cell adhesion molecule 1 (4x83_A)	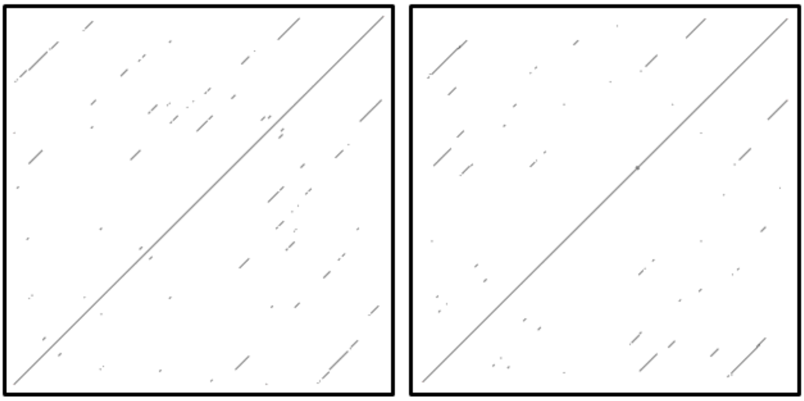	0.289
**5.5** EP-cadherin (1q55_D)	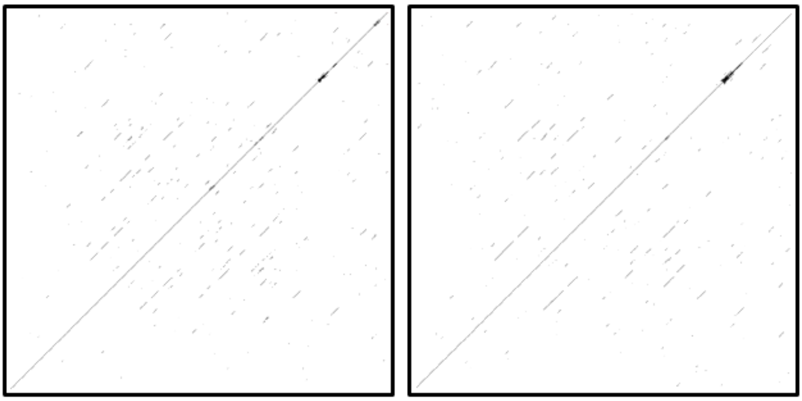	0.271
**5.5** Killer cell immuno-globulin-like receptor 3DL1 (3vh8_G)	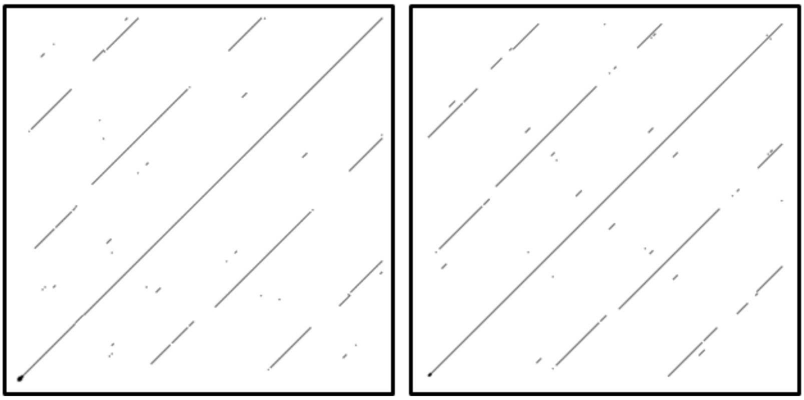	0.626

SI Fig. 6: Chart representing the proportion of manually examined clusters (N=8569) which had a single functional grouping (judged by UniProt protein name). Manual examination of those clusters which had between 5-200 members (n=8569) found that only 538 of the clusters were not comprised of a single functional type as judged by UniProt protein names while 925 clusters were made up of entirely or essentially entirely “uncharacterized” or “hypothetical” proteins. This means that 7104 clusters (82.9 %) were easily human identifiable as a single functional type (or 8031 (93.7%) if “uncharacterized” proteins are included as a functional group).

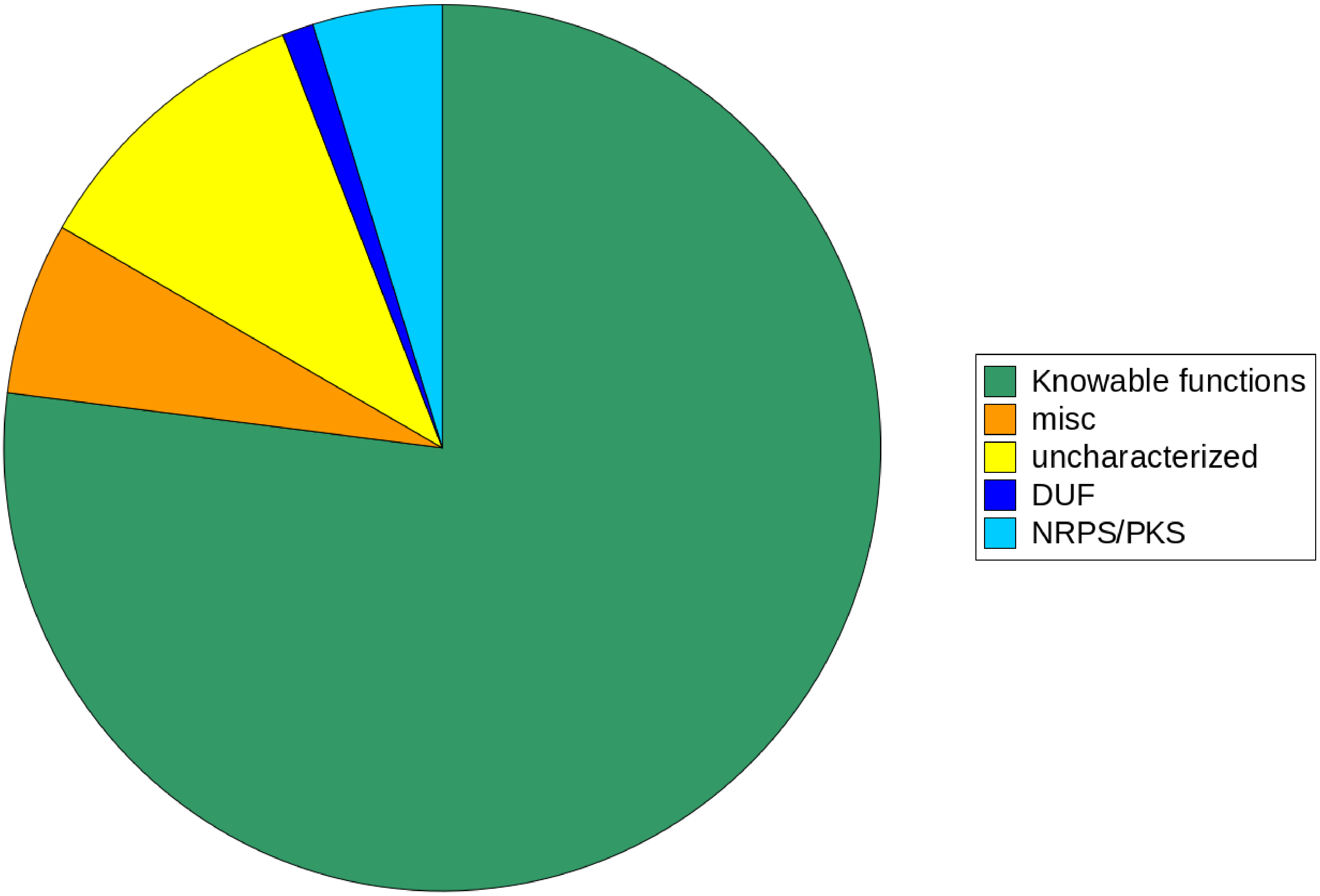

SI Fig. 7: Histogram of the distribution of mean sequence similarity between cluster members calculated using BLOSUM62. While most clusters were fairly sequence similar (≥ 0.8), not all of them were and analysis of the DOTTER plots was still able to make reasonable groupings when the mean similarity was as low as 32%.

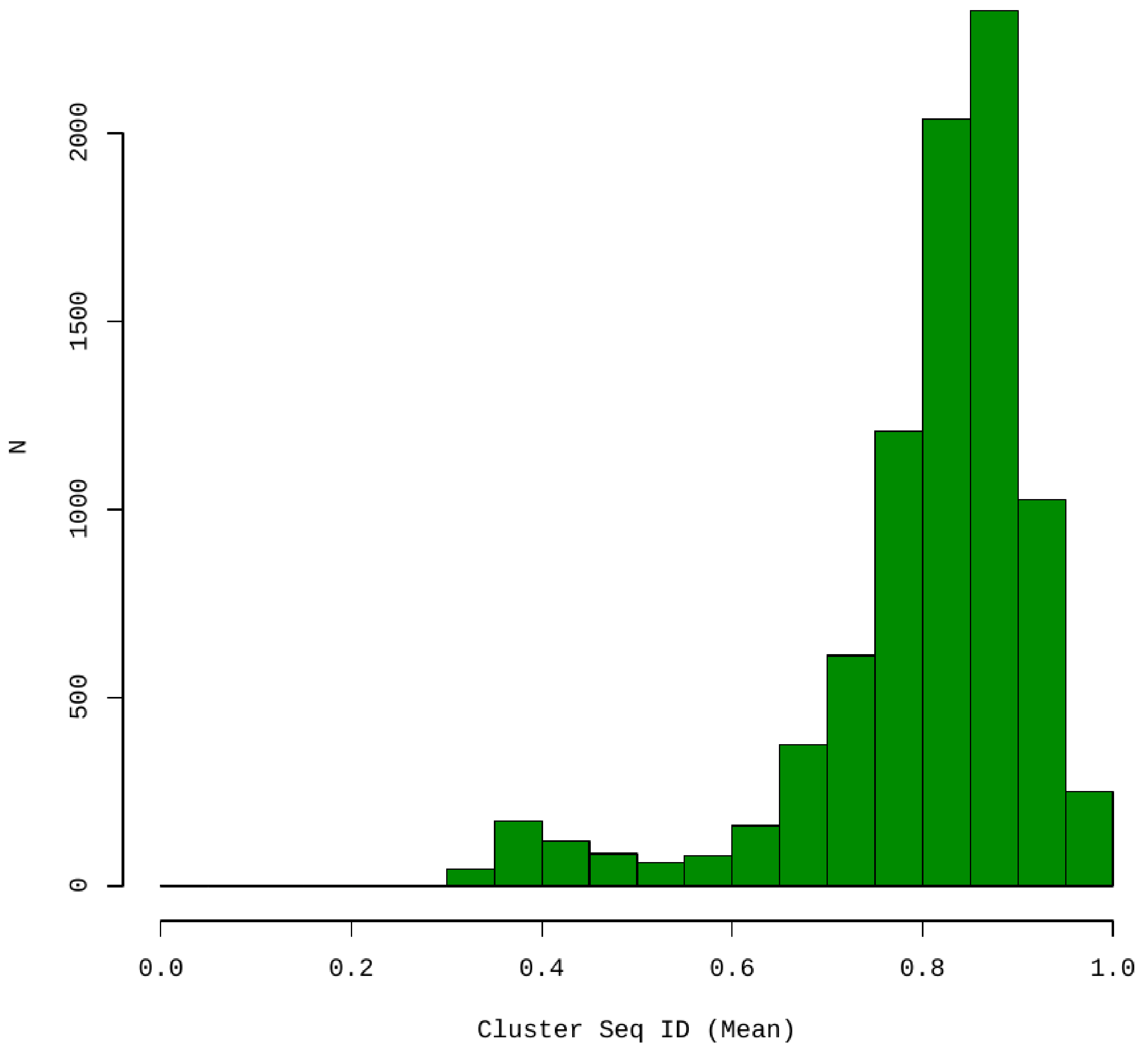

SI Fig. 8A: A larger version of figure 2A left panel (consensus plant RAP protein DOTTER plot)

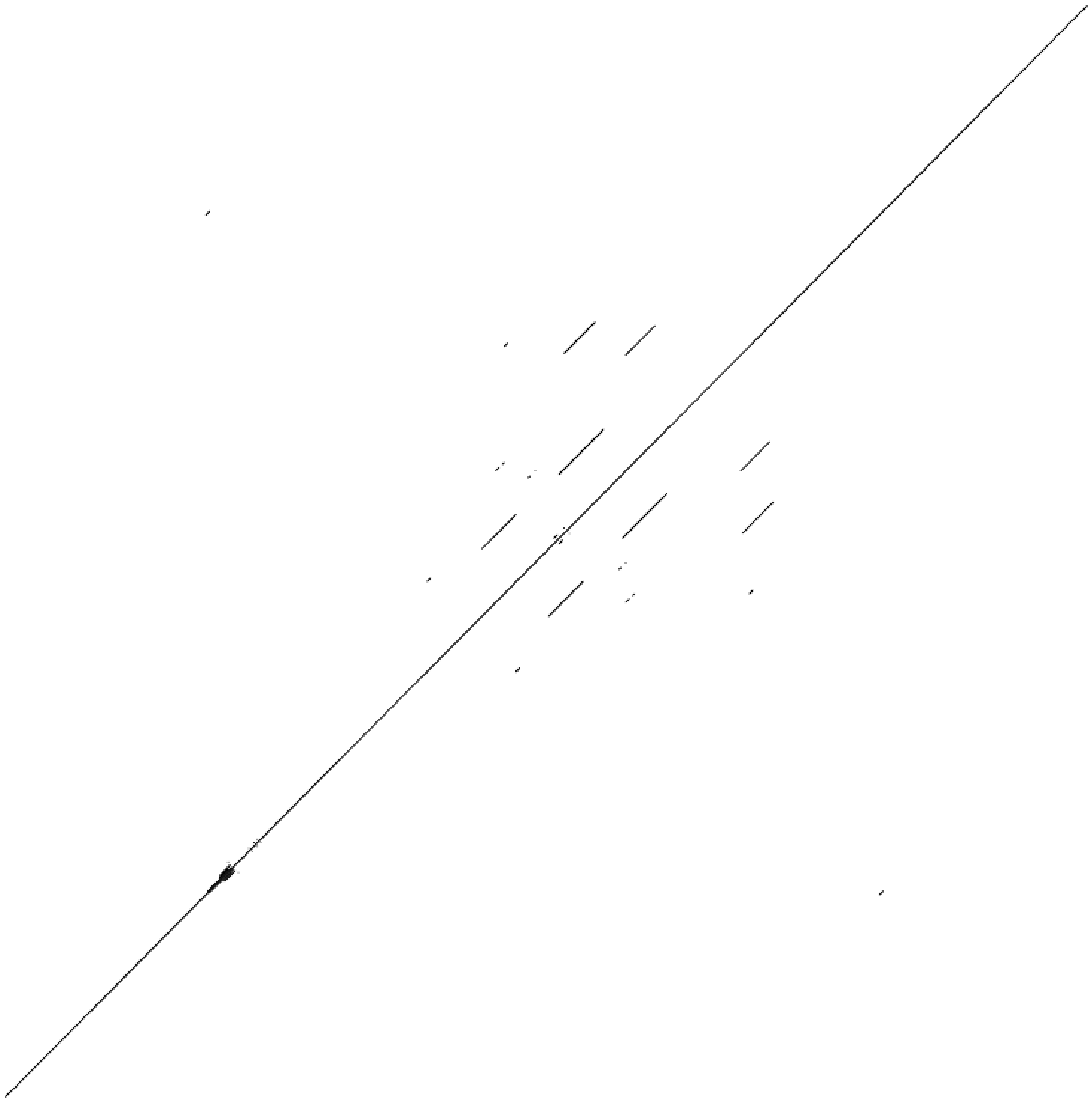

SI Fig. 8B: A larger version of figure 2A center panel (*S. tuberosum* plant RAP protein DOTTER plot)

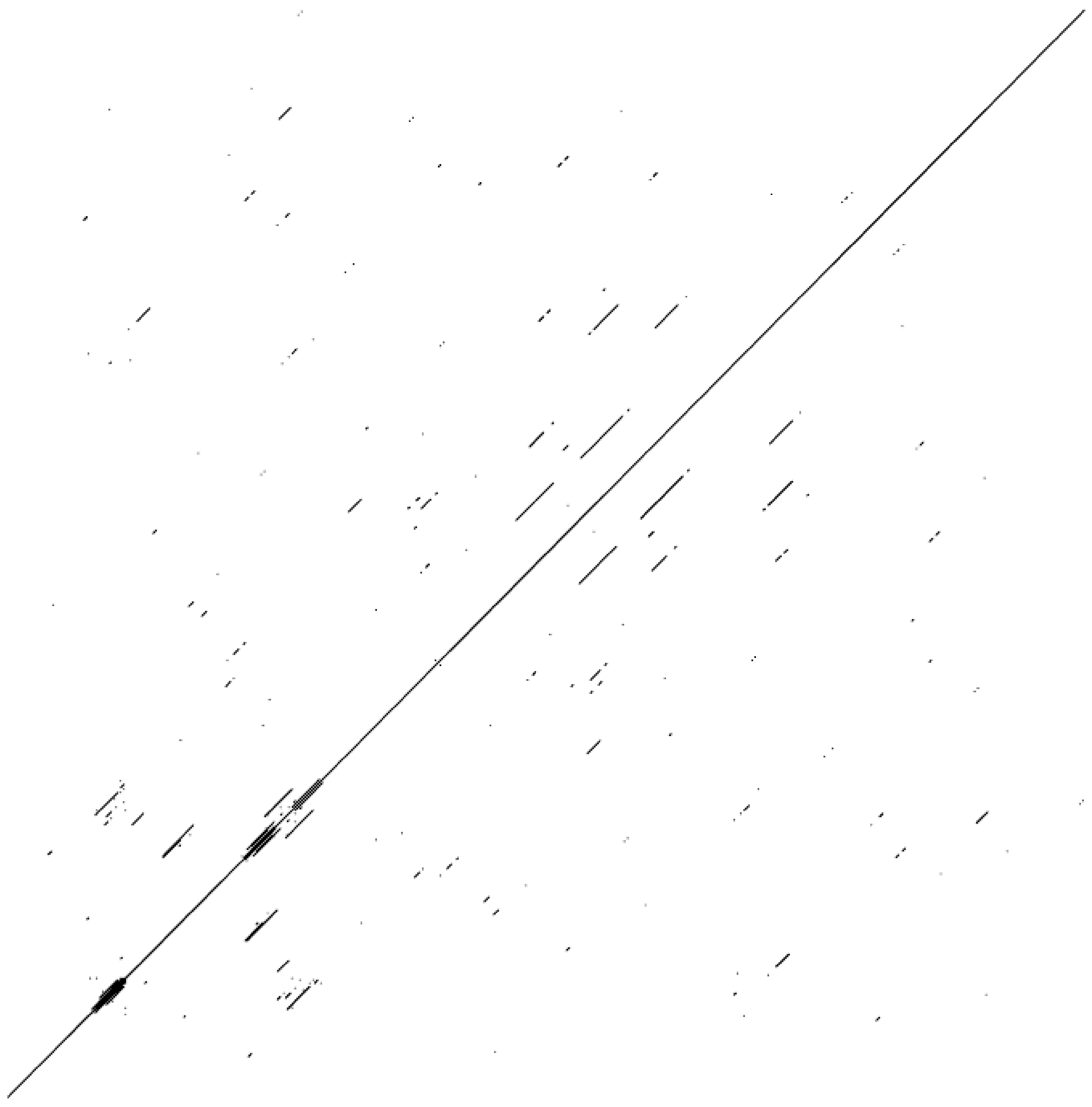

SI Fig. 8C: A larger version of figure 2A right panel (*P. patens* RAP protein DOTTER plot)

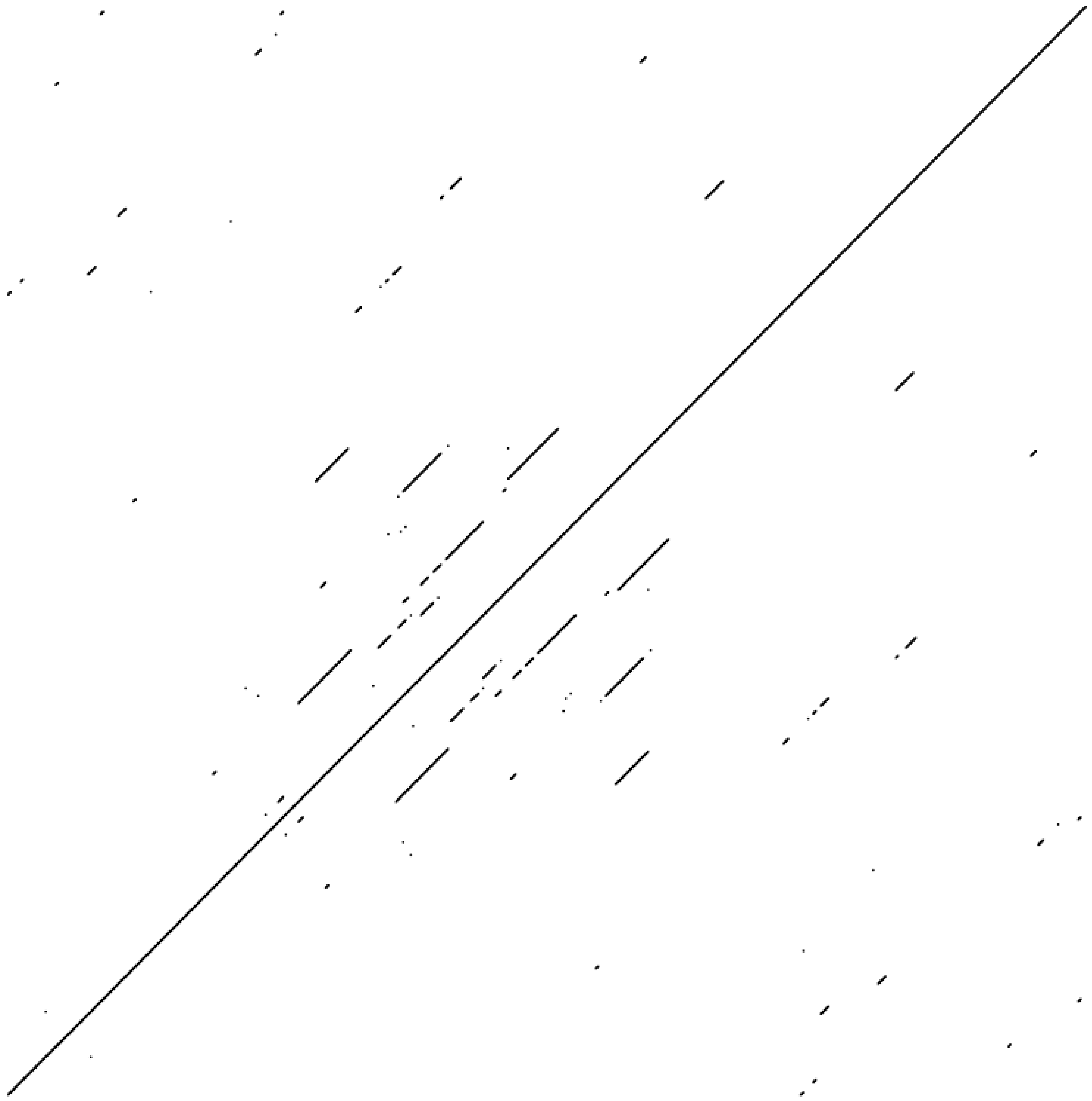

SI Fig. 8D: A larger version of figure 2B left panel (regulator of chromosome condensation (RCC) DOTTER plot)

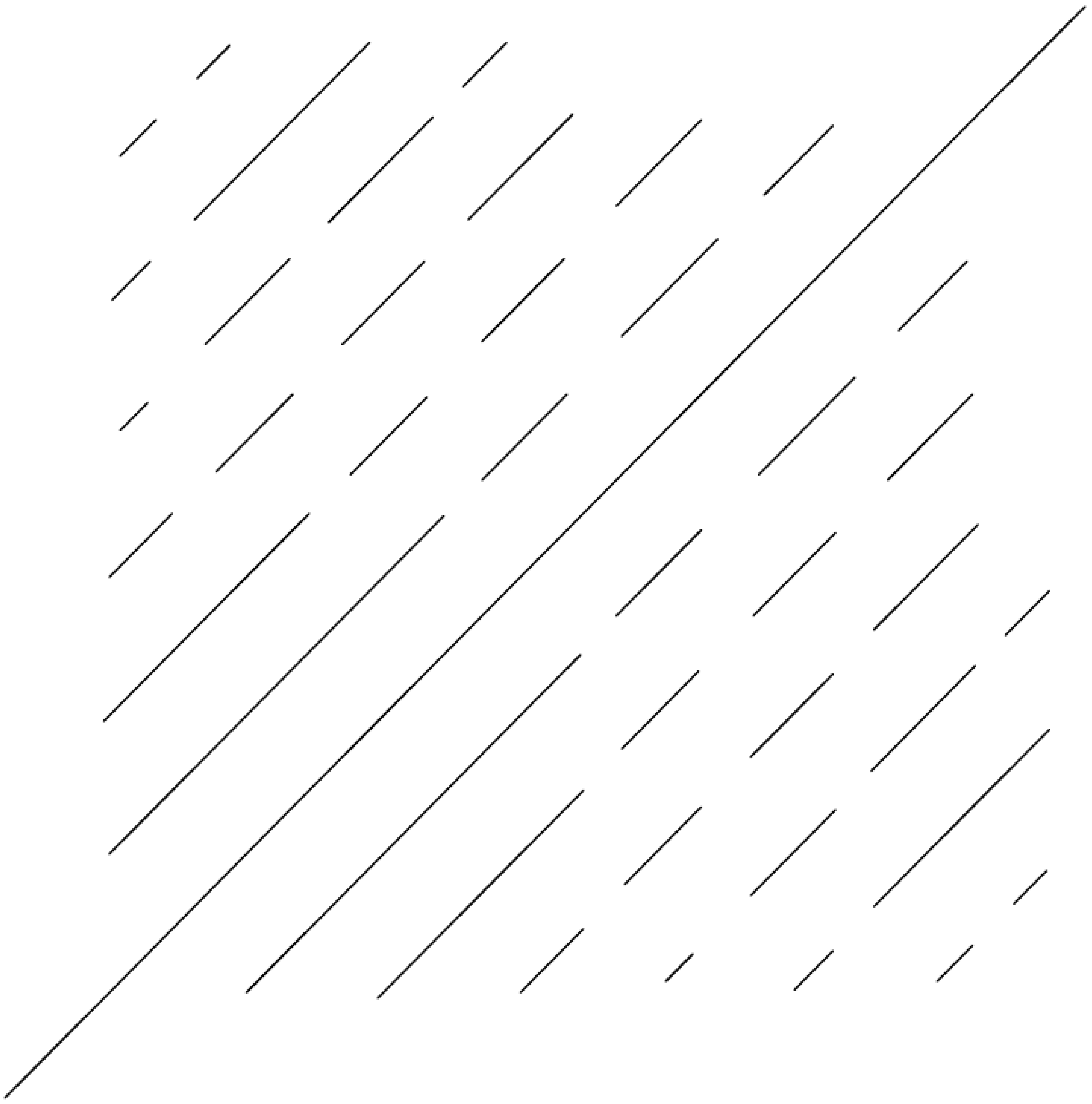

SI Fig. 8E: A larger version of figure 2B center panel (*P. trichocarpa* regulator of chromosome condensation DOTTER plot)

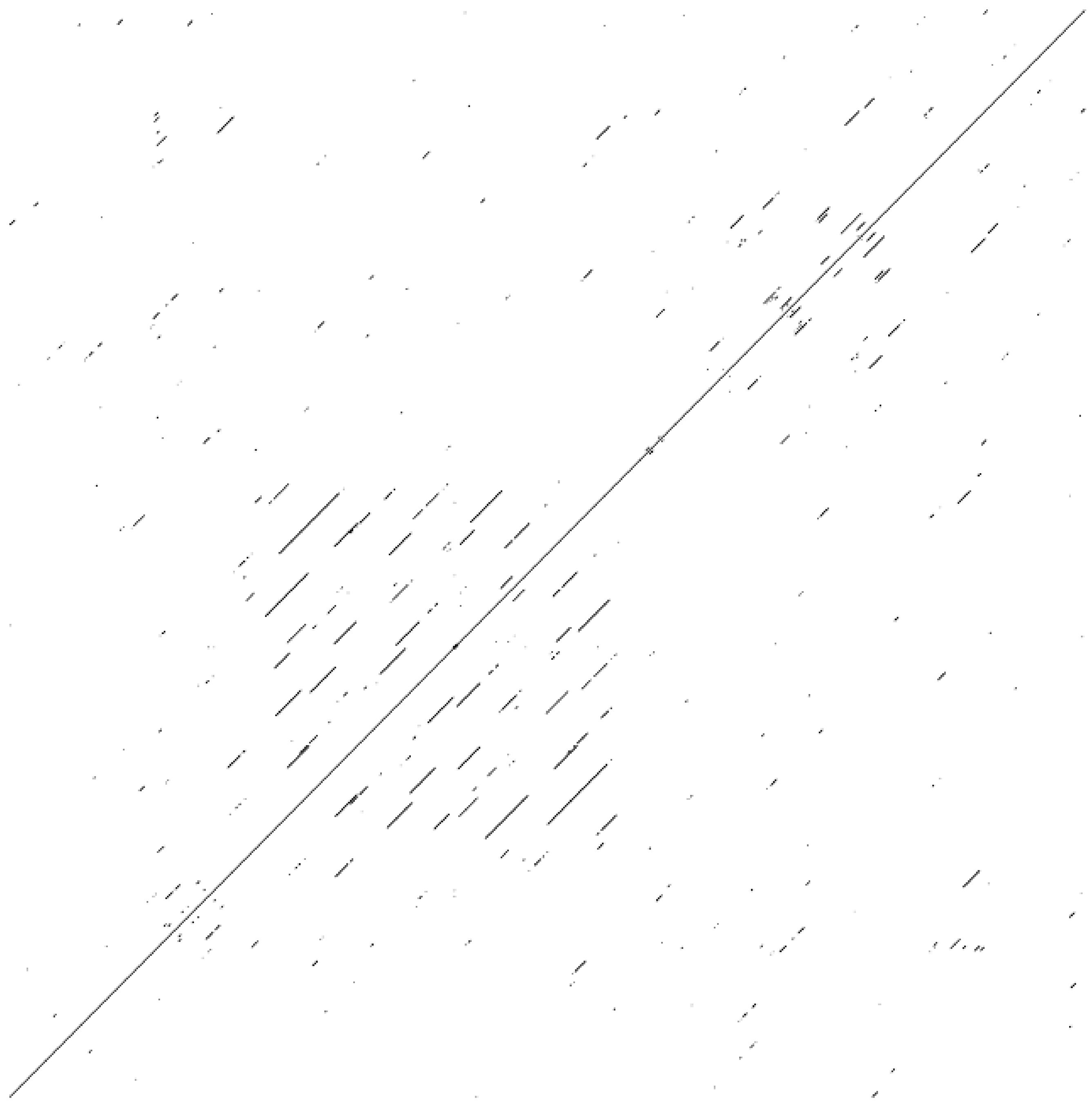

SI Fig. 8F: A larger version of figure 2B right panel (*S. arenicola* regulator of chromosome condensation DOTTER plot)

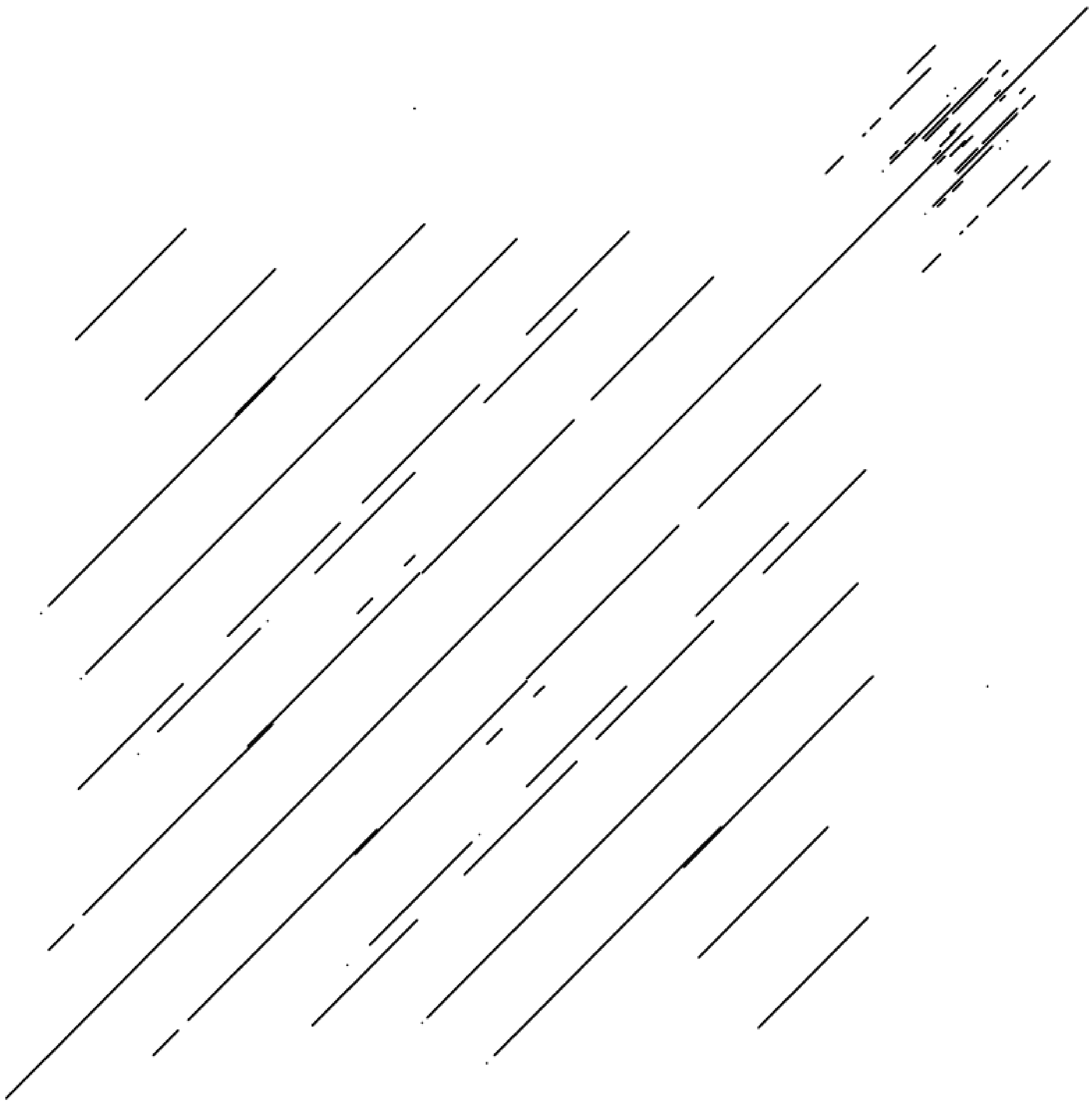

SI Fig. 8G: A larger version of figure 2C left panel (consensus DSCA protein DOTTER plot)

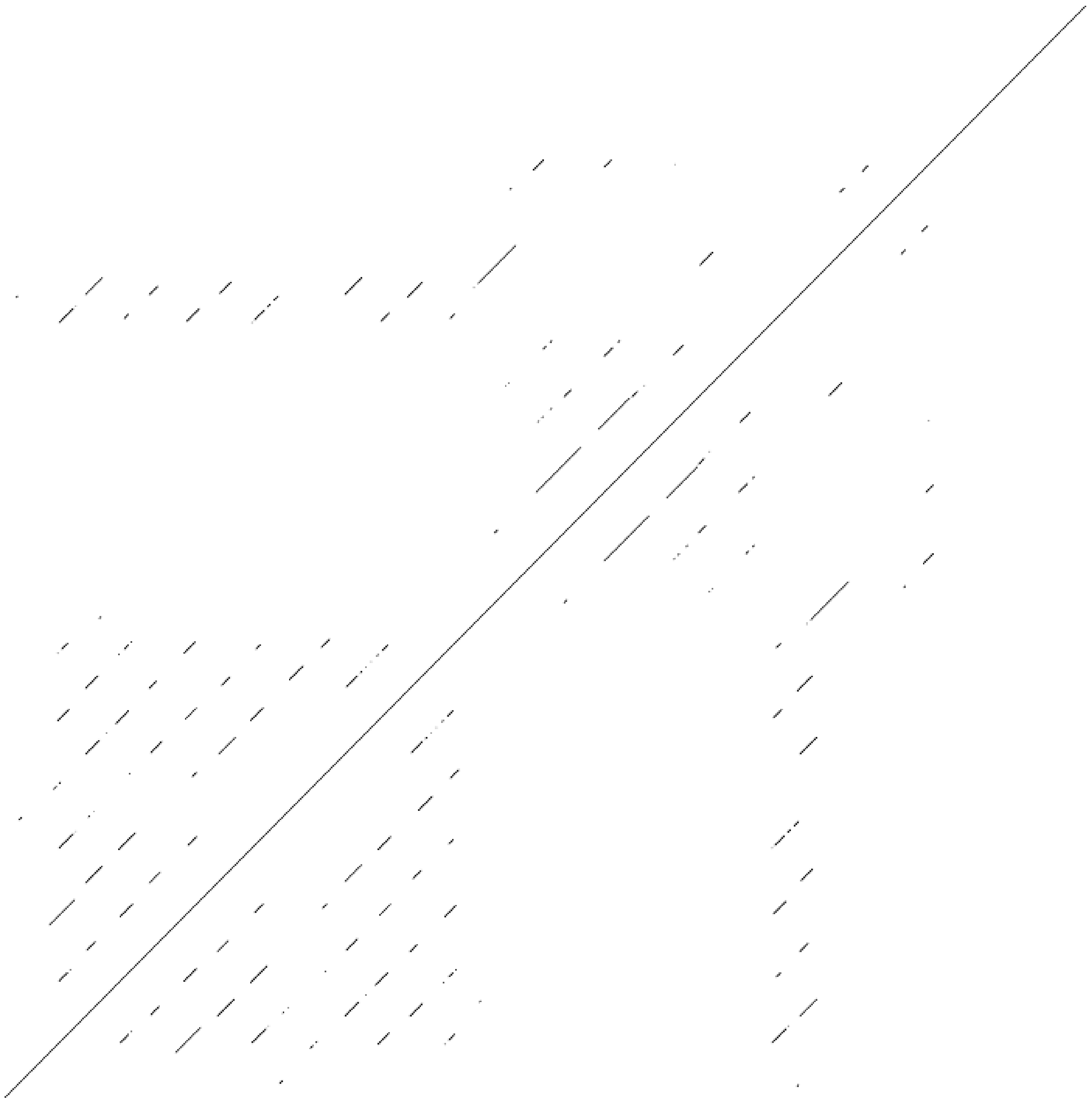

SI Fig. 8H: A larger version of figure 2C center panel (*P. trichocarpa* DSCA protein DOTTER plot)

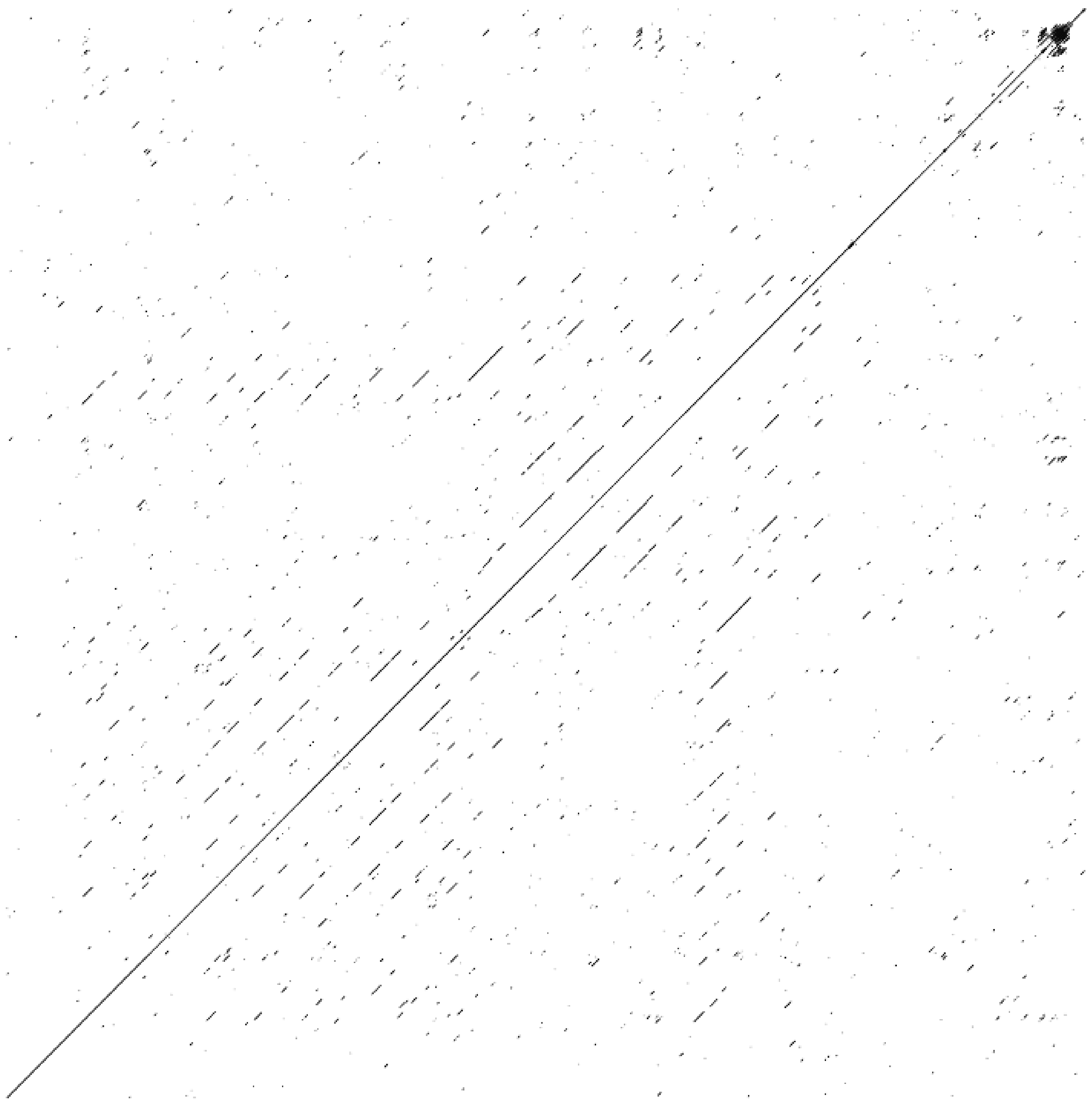

SI Fig. 8I: A larger version of figure 2C right panel (*S. arenicola* DSCA protein DOTTER plot)

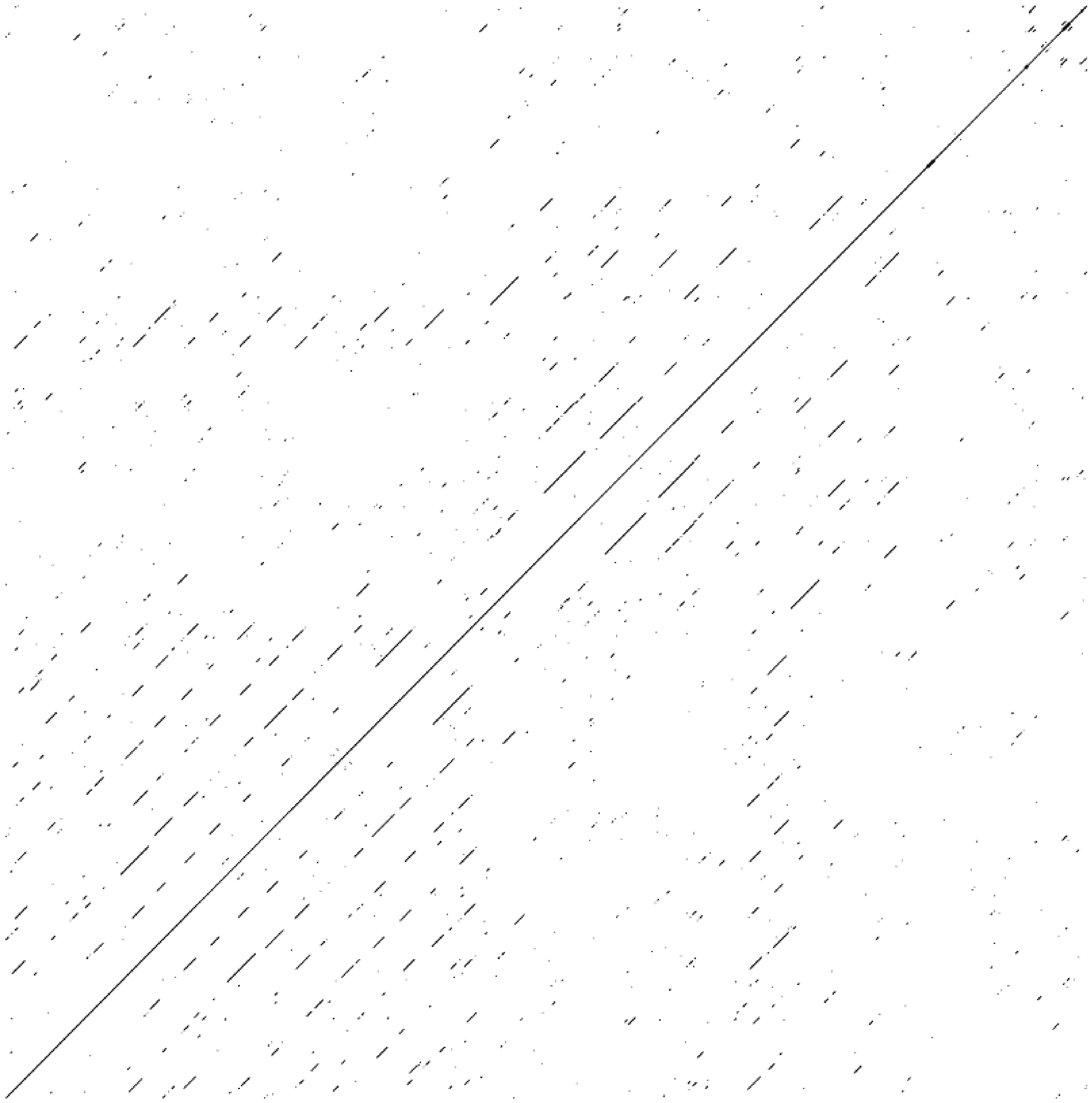

SI Fig. 8J: A larger version of figure 2D left panel (consensus CDC23 protein DOTTER plot)

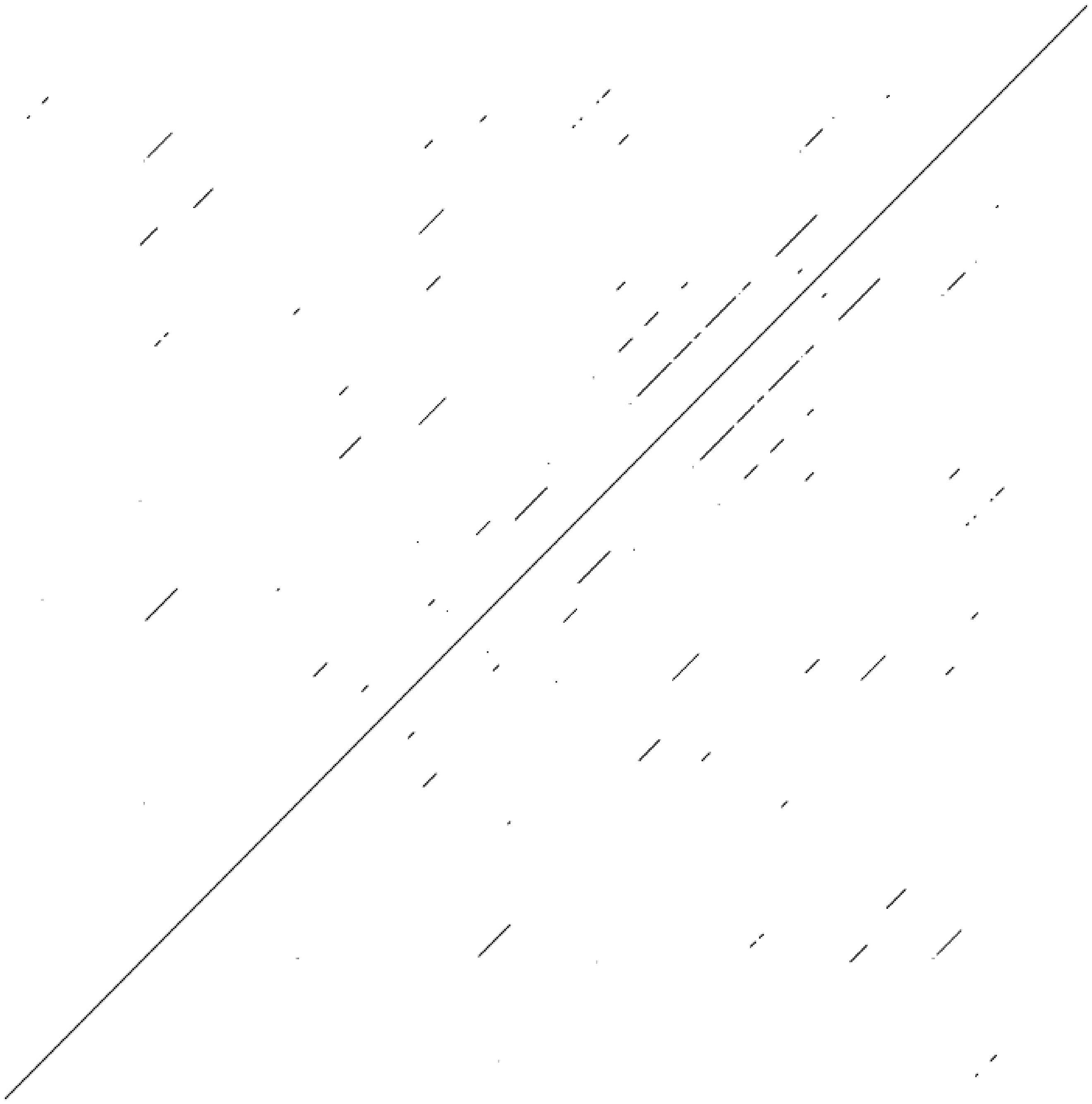

SI Fig. 8K: A larger version of figure 2D center panel (*N. korthausae* CDC23 protein DOTTER plot)

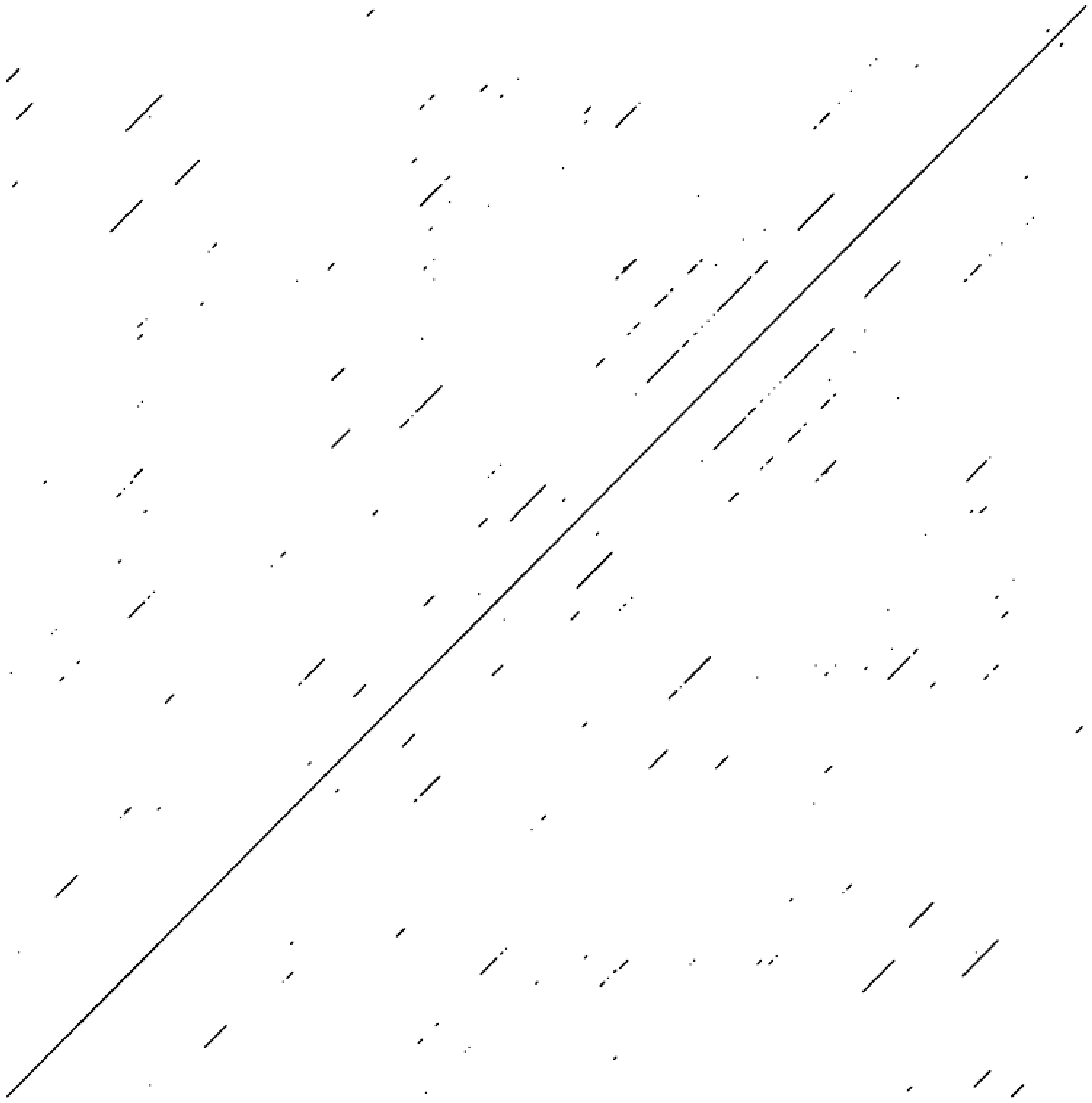

SI Fig. 8L: A larger version of figure 2D right panel (*A. platyrhynchos* CDC23 protein DOTTER plot)

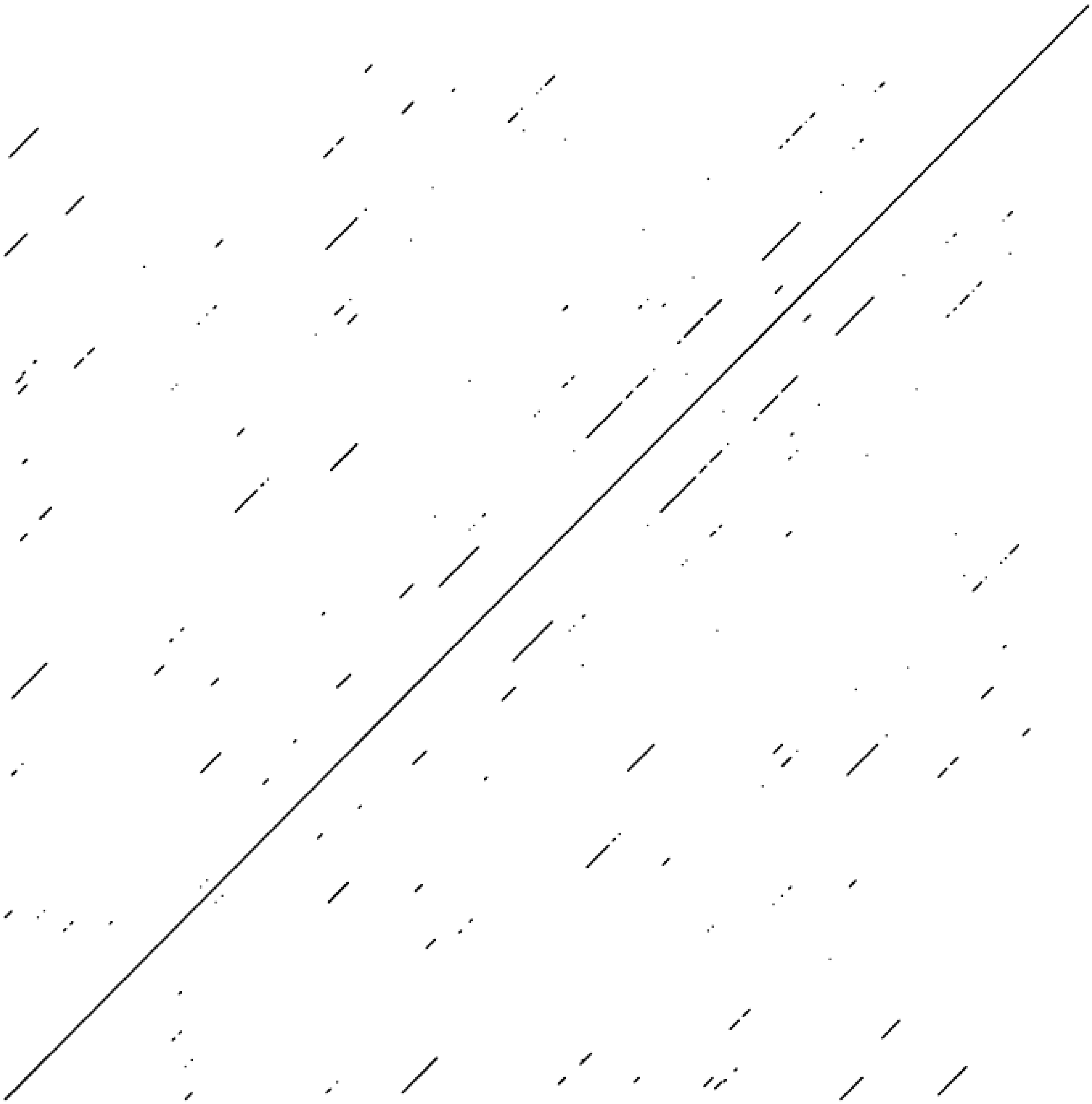

SI Table 1: Tabular results of *in silico* decay of J_X under random mutation showing the structural subgroup from RepeatsDB, name of the protein with PDB ID in parentheses if appropriate, length of the amino acid sequence as well as the exponential term and its associated R^2 value of the fit equation. Proteins with poor ($R^2 < 0.98$) fits to simple exponential decay are indicated by red text.

CLASS	Name	Length (a.a.)	Exp. term	R^2
2_2	chicken fibrinogen (1m1j_D)	491	8.90	0.9977
2_2	lamprey fibrinogen (1lwu_L)	323	8.41	0.9945
2_2	Rab guanine nucleotide exchange factor SEC2 (2e7s_N)	135	9.62	0.9824
3_1	endopolygalacturonase I (1kcc_A)	335	8.79	0.9892
3_1	pectate lyase (1ee6_A)	197	8.98	0.9969
3_1	polysialic acid O-acetyltransferase OatWY (2wlf_A)	215	8.56	0.9981
3_2	ribonuclease inhibitor (3tsr_F)	457	8.24	0.9782
3_2	platelet glycoprot 1B (1gwb_A)	281	8.91	0.9957
3_2	SKP2 (1fqv_A)	336	8.51	0.9995
3_3	*Xenopus laevis* beta catenin	781	8.37	0.9983
3_3	*Bos taurus* CDC23	597	8.62	0.9992
3_3	*Saccharomyces kudriavzevii* CYC8	984	6.21	0.9657
3_3	G-protein signal modulator 2 (3ro2_A)	338	8.34	0.9797
3_3	*Zea mays* heat shock protein STI	581	8.30	0.9967
3_3	*Wallemia ichthyophaga* heat shock protein STI1	573	8.81	0.9996
3_3	*Homo sapiens* IFIT1	478	8.74	0.9987
3_3	*Homo sapiens* IFIT2	472	8.70	0.9977
3_3	*Homo sapiens* IFIT3	490	8.72	0.9982
3_3	*Homo sapiens* IFIT5	482	8.86	0.9995
3_3	*Cynara cardunculus* importin alpha	333	9.47	0.9978
3_3	*Petromyzon marinus* kinesin light chain	640	8.21	0.9784
3_3	*Selaginella moellendorffii* peptide N-acetylglucosaminyltransferase/Spindly	933	8.09	0.9951
3_3	*Oryzias latipes* peptidylprolyl isomerase	404	8.93	0.9945
3_3	*Rattus norvegicus* plakoglobin	745	8.39	0.9989
3_3	*Camelus ferus* receptor-associated protein of the synapse/RAPSYN	412	8.80	0.9991
3_3	*Saccharomyces cerevisiae* serine/threonine-protein phosphatase	513	8.86	0.9968
3_4	adenovirus fibre (1qiu_A)	264	8.70	0.9987
3_4	pneumococcal surface protein (4cnl_A)	182	5.72	0.8993
3_4	teichioic acid phosphorylcholine esterase CbpE (2bib_A)	547	7.25	0.9848
3_5	LPS Transporter LptDE (5iv9_A)	758	7.73	0.9960
3_5	outer surface protein A (2oy8_A)	320	5.45	0.8901
3_5	periplasmic lipopolysaccharide transport protein LPTH (4uu4_A)	175	8.32	0.8431
3_6	DNA polymerase III (4trt_B)	498	5.80	0.9196
3_6	DNA polymerase sliding clamp A (2nti_C)	244	9.32	0.9980
3_6	proliferating cell nuclear antigen (1vym_A)	261	9.22	0.9892
4_1	4-hydroxy-2-oxoglutarate aldolase (1vlw_A)	217	8.33	0.9993
4_1	deoxyribose-phosphate aldolase (3r12_A)	260	7.96	0.9932

**4_1**	pyridoxal biosynthesis lyase (4wxz_A)	304	9.02	0.9988
**4_2**	ferric citrate transporter FecA (1pnz_A)	751	8.11	0.9948
**4_2**	intestinal fatty acid-binding protein (1sa8_A)	106	8.70	0.9982
**4_2**	outer membrane permeability OccAB1 (5dl5_A)	430	8.05	0.9967
**4_3**	Kunitz-type kallikrein inhibitor BbKI (2go2_A)	163	8.71	0.9857
**4_3**	lysenin pore (5ec5_P)	298	8.70	0.9978
**4_3**	Winged bean chymotrypsin inhibitor (2beb_A)	186	8.99	0.9892
**4_4**	coat for the nuclear pore (3bg1_D)	316	7.71	0.9964
**4_4**	*Macaca mulatta* intraflagellar transport protein 122	1182	8.08	0.9949
**4_4**	*Drosophila melanogaster* ring canal kelch protein	739	7.27	0.9990
**4_5**	3-phosphoshikimate 1-carboxyvinyltransferase (2o15_A)	450	8.92	0.9984
**4_5**	BamABCDE complex (5d0o_B)	392	8.65	0.9985
**4_5**	enolpyruvyl transferase NikO (4fqd_B)	479	8.78	0.9973
**4_6**	complement component C3 (2a73_A)	643	8.82	0.9967
**4_6**	squalene-hoplene cyclase (2sqc_A)	631	8.04	0.9988
**4_6**	thioester-containing protein I (2pn5_A)	1325	8.29	0.9904
**4_7**	eukaryotic ribosome anti-association factor IF6 (1g62_A)	224	9.37	0.9989
**4_7**	prokaryotic ribosome anti-association factor IF6 (1g61_B)	228	9.21	0.9994
**4_8**	90S pre-ribosome (5jpq_A)	1183	7.76	0.9959
**4_8**	a-L-arabinofuranosidase (3wmzA)	438	7.80	0.9866
**4_8**	*Heterocephalus glaber* regulator of chromosome condensation/RCC	437	8.47	0.9908
**4_9**	b-L-arabinopyranosidase (3a21_B)	614	7.90	0.9950
**4_9**	endo-1,4-beta-xylanase A (1knm_A)	130	8.46	0.9726
**4_9**	GalNAc/Gal-specific lectin (5f8w_A)	156	6.90	0.9210
**4_10**	pancreatic secretory protein ZG16p (3vzf_A)	141	8.88	0.9923
**4_10**	ripening-associated protein (2bn0_A)	141	9.33	0.9911
**4_10**	vitelline membrane outer layer prot. 1 (1vmoA)	163	7.72	0.9725
**5_1**	alpha-11 giardin (2iic_D)	310	8.80	0.9985
**5_1**	annexin XII (1dm5_B)	315	8.71	0.9841
**5_1**	hypothetical protein EbhA (2dgj_A)	257	8.27	0.9816
**5_2**	accumulation associated protein (4fum_A)	208	4.89	0.8804
**5_2**	CD55 decay accelerating factor (1ok1_B)	254	8.02	0.9917
**5_2**	complement control protein (1y8e_B)	244	7.52	0.9914
**5_3**	Aart, designed six finger zinc finger (2i13_B)	190	4.68	0.8100
**5_3**	histone-lysine N-methyltransferase PRDM9 (5eh2_F)	144	4.22	0.7800
**5_3**	ser/thr kinase (3m9g_A)	201	9.37	0.9976
**5_4**	C3 convertase (2win_E)	645	8.84	0.9965
**5_4**	calcium-binding adhesin SiiE (2yn3_A)	288	8.33	0.9888
**5_4**	receptor-type tyrosine-protein phosphatase delta (4rca_A)	308	7.99	0.9928
**5_5**	Down syndrome cell adhesion molecule 1 (4x83_A)	394	8.15	0.9985
**5_5**	EP-cadherin (1q55_D)	880	7.98	0.9989
**5_5**	killer cell immunoglobulin-like receptor 3DL1 (3vh8_G)	316	7.50	0.9749

SI Table 2: FASTA sequences of all the proteins analyzed in the 79 member standard set and the associated mutated sequences

>chicken fibrinogen (1m1j_D)
QDGKTTFEKEGGGGRGPRILENMHESSCKYEKNWPICVDDDWGTKCPSGCRMQGIIDDTD
QNYSQRIDNIRQQLADSQNKYKTSNRVIVETINILKPGLEGAQQLDENYGHVSTELRRRIVT
LKQRVATQVNRIKALQNSIQEQVVEMKRLEVDIDIKIRACKGSCARSFDYQVDKEGYDNIQ
KHLTQASSIDMHPDFQTTTLSTLKMRPLKDSNVPEHFKLKPSPEMQAMSAFNNIKQMQVV
LERPETDHVAEARGDSSPSHTGKLITSSHRRESPSLVDKTSSASSVHRCTRTVTKKVISGPDG
PREEIVEKMVSSDGSDCSHLQGGREGSTYHFSGTGDFHKLDRLLPDLESFFTHDSVSTSSRH
SIGSSTSSHVTGAGSSHLGTGGKDKFTDLGEEEEDDFGGLQPSGFAAGSASHSKTVLTSSSSS
FNKGGSTFETKSLKTRETSEQLGGVQHDQSAEDTPDFKARSFRPAAMSTRRSYNGKGTQK
>lamprey fibrinogen (1lwu_L)
DSGQKTVQKILEEVRILEQIGVSHDAQIQELSEMWRVNQQFVTRLQQQLVDIRQTCSRPCQ
DTTANKISPITGKDCQQVVDNGGKDSGLYYIKPLKAKQPFLVFCEIENGNGWTVIQHRHDG
SVNFTRDWVSYREGFGYLAPTLTTEFWLGNEKIHLLTGQQAYRLRIDLTDWENTHRYADYG
HFKLTPESDEYRLFYSMYLDGDAGNAFDGFDFGDDPQDKFYTTHLGMLFSTPERDNDKYE
GSCAEQDGSGWWMNRCHAGHLNGKYYFGGNYRKTDVEFPYDDGIIWATWHDRWYSLK
MTTMKLLPMGRDLSGHGGQQQSK
>Rab guanine nucleotide exchange factor SEC2 (2e7s_N)
GPLGSLEEQLNKSLKTIASQKAAIENYNQLKEDYNTLKRELSDRDDEVKRLREDIAKENEL
RTKAEEEADKLNKEVEDLTASLFDEANNLVADARMEKYAIEILNKRLTEQLREKDMLLDTL
TLQLKNLKKVMHS
>endopolygalacturonase I (1kcc_A)
ATCTVKSVDDAKDIAGCSAVTLNGFTVPAGNTLVLNPDKGATVTMAGDITFAKTTLDGPLF
TIDGTGINFVGADHIFDGNGALYWDGKGTNNGTHKPHPFLKIKGSGTYKKFEVLNSPAQAI
SVGPTDAHLTLDGITVDDFAGDTKNLGHNTDGFDVSANNVTIQNCIVKNQDDCIAINDGNN
IRFENNQCSGGHGISIGSIATGKHVSNVVIKGNTVTRSMYGVRIKAQRTATSASVSGVTYDA
NTISGIAKYGVLISQSYPDDVGNPGTGAPFSDVNFTGGATTIKVNNAATRVTVECGNCSGN
WNWSQLTVTGGKAGTIKSDKAKITGGQYL
>pectate lyase (1ee6_A)
APTVVHETIRVPAGQTFDGKGQTYVANPNTLGDGSQAENQKPIFRLEAGASLKNVVIGAPA
ADGVHCYGDCTITNVIWEDVGEDALTLKSSGTVNISGGAAYKAYDKVFQINAAGTINIRNF
RADDIGKLVRQNGGTTYKVVMNVENCNISRVKDAILRTDSSTSTGRIVNTRYSNVPTLFKG
FKSGNTTASGNTQY
>polysialic acid O-acetyltransferase OatWY (2wlf_A)
MGTHMYSEQGINNTINISTTSLTNATQLTVIGNNNSVYIGNNCKIVSSNIRLKGNNITLFIADD
VEIMGLVCSLHSDCSLQIQAKTTMGNGEITIAEKGKISIGKDCMLAHGYEIRNTDMHPIYSL
ENGERINHGKDVIIGNHVWLGRNVTILKGVCIPNNVVVGSHTVLYKSFKEPNCVIAGSPAKI
VKENIVWGRKMYHSTMYDDPTLNEFYK
>ribonuclease inhibitor (3tsr_F)
MMSLDIQCEQLSDARWTELLPLIQQYEVVRLDDCGLTEVRCKDISSAVQANPALTELSLRTN
ELGDGGVGLVLQGLQNPTCKIQKLSLQNCGLTEAGCGILPGMLRSLSTLRELHLNDNPMGD
AGLKLLCEGLQDPQCRLEKLQLEYCNLTATSCEPLASVLRVKADFKELVLSNNDLHEPGVRI
LCQGLKDSACQLESLKLENCGITAANCKDLCDVVASKASLQELDLSSNKLGNAGIAALCPG
LLLPSCKLRTLWLWECDITAEGCKDLCRVLRAKQSLKELSLASNELKDEGARLLCESLLEPG
CQLESLWIKTCSLTAASCPYFCSVLTKSRSLLELQMSSNPLGDEGVQELCKALSQPDTVLRE
LWLGDCDVTNSGCSSLANVLLANRSLRELDLSNNCMGGPGVLQLLESLKQPSCTLQQLVL
YDIYWTNEVEEQLRALEEERPSLRIIS
>platelet glycoprotein 1B (1gwb_A)

PHPICEVSKVASHLEVNCDKRNLTALPPDLPKDTTILHLSENLLYTFSLATLMPYTRLTQLNL
DRCELTKLQVDGTLPVLGTLDLSHNQLQSLPLLGQTLPALTVLDVSFNRLTSLPLGALRGLG
ELQELYLKGNELKTLPPGLLTPTPKLEKLSLANNNLTELPAGLLNGLENLDTLLLQENSLYTI
PKGFFGSHLLPFAFLHGNPWLCNCEILYFRRWLQDNAENVYVWKQGVDVKAMTSNVASV
QCDNSDKFPVYKYPGKGCPTLGDEGDTDLYDYYP
>SKP2 (1fqv_A)
RENFPGVSWDSLPDELLLGIFSCLCLPELLKVSGVCKRWYRLASDESLWQTLDLTGKNLHP
DVTGRLLSQGVIAFRCPRSFMDQPLAEHFSPFRVQHMDLSNSVIEVSTLHGILSQCSKLQNL
SLEGLRLSDPIVNTLAKNSNLVRLNLSGCSGFSEFALQTLLSSCSRLDELNLSWCFDFTEKHV
QVAVAHVSETITQLNLSGYRKNLQKSDLSTLVRRCPNLVHLDLSDSVMLKNDCFQEFFQLN
YLQHLSLSRCYDIIPETLLELGEIPTLKTLQVFGIVPDGTLQLLKEALPHLQINCSHFTTIARPT
IGNKKNQEIWGIKCRLTLQKPSCL
>*Xenopus laevis* beta catenin
MATQADLMELDMAMEPDRKAAVSHWQQQSYLDSGIHSGATTTAPSLSGKGNPEDEDVDT
NQVLYEWEQGFSQSFTQDQVADIDGQYAMTRAQRVRAAMFPETLDEGMQIPSTQFDSAHP
TNVQRLAEPSQMLKHAVVNLINYQDDAELATRAIPELTKLLNDEDQVVVNKAAVMVHQLS
KKEASRHAIMRSPQMVSAIVRTMQNTNDVETARCTAGTLHNLSHHREGLLAIFKSGGIPAL
VKMLGSPVDSVLFYAITTLHNLLLHQEGAKMAVRLAGGLQKMVALLNKTNVKFLAITTDC
LQILAYGNQESKLIILASGGPQALVNIMRTYSYEKLLWTTSRVLKVLSVCSSNKPAIVEAGG
MQALGLHLTDSSQRLVQNCLWTLRNLSDAATKQEGMEGLLGTLVQLLGSDDINVVTCAAG
ILSNLTCNNYKNKMMVCQVGGIEALVRTVLRAGDREDITEPAICALRHLTSRHQEAEMAQN
AVRLHYGLPVVVKLLHPPSHWPLIKATVGLIRNLALCPANHAPLREQGAIPRLVQLLVRAHQ
DTQRRTSIGGTQQQFVEGVRMEEIVEGCTGALHILARDIHNRIVIRGLNTIPLFVQLLYSPIEN
IQRVAAGVLCDVAQDKEAAEAIEAEGATAPLTELLHSRNEGVATYAAAVLFRMSEDKPQDY
KKRLSVELTSSLFRTEPMPWNEAADLGLDIGAQGEPLGYRQDDSSYRSFHAAGYGQDAMG
MDSMMDHDMGGHHPGADYPVDGLPDLSHAQDLMDGLPPGDSNQLAWFDTDL
>*Bos taurus* CDC23
MAASSSIVSVAPTASSVPVLPSSCDFSNLREIKKQLLLIAGLTRERGLLHSSKWSAELAFSLPA
LPLSELQPPPPITEEDAQDMDAYTLAKAYFDVKEYDRAAHFLHGCNSKKAYFLYMYSRYLS
GEKKKDDETVDSLGPLEKGQVKNEALRELRVELSKKHQARELDGFGLYLYGVVLRKLDLV
KEAIDVFVEATHVLPLHWGAWLELCNLITDKEMLKFLSLPDTWMKEFFLAHIYTELQLIEE
ALQKYQNLIDVGFSKSSYIVSQIAVAYHNIRDIDKALSIFNELRKQDPYRIENMDTFSNLLYV
RSMKSELSYLAHNLCEIDKYRVETCCVIGNYYSLRSQHEKAALYFQRALKLNPRYLGAWTL
MGHEYMEMKNTSAAIQAYRHAIEVNKRDYRAWYGLGQTYEILKMPFYCLYYYRRAHQLR
PNDSRMLVALGECYEKLNQLVEAKKCYWRAYAVGDVEKMALVKLAKLHEQLTESEQAAQ
CYIKYIQDIYSCGEIVEHLEESTAFRYLAQYYFKCKLWDEASACAQKCCAFNDTREEGKAL
LRQILQLRNQGETPSTEIPAPFFLPASLSANNTPTRRVSPLNLSSVTP
>*Saccharomyces kudriavzevii* CYC8
MNSGGEQTIMEQPAQQQQQQQQQQAAVPQQPLDPLTQSTAETWLSIASLAETLGDGDRAA
MAYDATLQFNPSSAKALTSLAHLYRSRDMFQRAAELYERALLVNPELSDVWATLGHCYLM
LDDLQRAYNAYQQALYHLSNPNVPKLWHGIGILYDRYGSLDYAEEAFAKVLELDPHFEKAN
EIYFRLGIIYKHQGKWSQALECFRYILPQPPAPLQEWDIWFQLGSVLESMGEWQGAKEAYE
HVLAQNQHHAKVLQQLGCLYGMSNVQFYDPQKALDYLLKSLEADPSDATTWYHLGRVH
MIRTDYTAAYDAFQQAVNRDSRNPIFWCSIGVLYYQISQYRDALDAYTRAIRLNPYISEVWY
DLGTLYETCNNQLSDALDAYKQAARLDVNNVHIRERLEALTKQLENPGNINKSNGAPSNAS
PAPPPVILQPTLQPNDQGNPLNARIPAQSLNAAASLVQQQHPAQQTPINSSATMYTNGASPQ
LQAHAQAQAQAQAQAQAQAQAQAQAQAQAQAQAQAQAQAQAQAQAQAQAQAQAQAQAQA
QAQAQAQAQAQAQAQQHQAQQHQAQQHQAQQHQAQQHQAQQHQAQQHQAQQQQQQ
QQQQQQQQQLQQQQLQALPRQQQHQKGVSVQMLNPQQGQPYVTQPTVIQAHQLQPFSTQ
AMEHPQGSQLSPQQQLQSIQHPQQLQSQHQSQPQPLIQRGLEQNSLPQKRYMEGAIHTLVD

AAVSSSTHPENNAKSPNQLPHSISNQTSATVTPNIGPQPKKQKLNSPNSNINKLVNTATEENS
AHEITNSPLPVAESNATSIQEEKPARANSIPPVIYAQEPSKEATTNEETAKPAPVPTPTNSSNVE
VEPFAAQTTLSSENSTNRANEESTAGTIEPFTDANPAEASPGEEDLKQHAKEEKSTTPEATEK
KEPPTGGENEPQQEDSLTTTTTATATVIKPTLETMETVKEETKMREEEQTPQEKPPQENLLPR
ENVVRQVEEDENYDD
>G-protein signal modulator 2 (3ro2_A)
GSASCLELALEGERLCKSGDCRAGVSFFEAAVQVGTEDLKTLSAIYSQLGNAYFYLHDYAK
ALEYHHHDLTLARTIGDQLGEAKASGNLGNTLKVLGNFDEAIVCCQRHLDISRELNDKVGE
ARALYNLGNVYHAKGKSFGCPGPQDTGEFPEDVRNALQAAVDLYEENLSLVTALGDRAAQ
GRAFGNLGNTHYLLGNFRDAVIAHEQRLLIAKEFGDKAAERRAYSNLGNAYIFLGEFETASE
YYKKTLLLARQLKDRAVEAQSCYSLGNTYTLLQDYEKAIDYHLKHLAIAQELKDRIGEGR
ACWSLGNAYTALGNHDQAMHFAEKHLEISREVGD
>*Zea mays* heat shock protein STI
MADEAKAKGNAAFSAGRFEEAARHFTDAIALAPGNHVLYSNRSAALASLHRYSDALADA
QKTVELKPDWAKGYSRLGAAHLGLGDAASAVAAYEKGLALDPSNEGLKAGLEDAKKAAA
APPRRGPSGPDAIGQMFQGPELWSKIASDPTTRAYLNEPDFMQMLREVQRNPSSISMYLSDP
RMMQVLSLMLNVKIQRPEASEPSQSTPPPPPQQQQQQTSPLQTKAREVEPEPEPEPMDFTDE
EKERKERKAAAQKEKELGNTAYKKKDFEAAIQHYTKALELDDEDISYLTNRAAVYIEMGK
YDECIKDCDKAVERGRELRADFKMISRALTRKGTALVKLAKTSKDFDIAIETYQKALTEHRN
PDTLKKLNEAERAKKELEQQEYYDPKLADEEREKGNEFFKEQKYPEAIKHYTEALKRNPK
DPRVYSNRAACYTKLGAMPEGLKDAEKCLELDPTFTKGYTRKGAIQFFMKEYDKAMETY
QAGLKHDPKNQELLDGVRRCIEQINKANRGEISQDELQERQNKAMQDPEIQNILTDPIMRQ
VLNDFQENPRAAQEHLKNPGVMQKIQKLVSAGIVQMR
>*Wallemia ichthyophaga* heat shock protein STI1
MSADELKAQGNAAFSAKDFTKAVDLFTQAIAQDANNHVLYSNRSASYAGLKQYQKALDD
ASTTISKKSDWPKGYARKGAALHGLQQYMESVEAYEQGLKAAPTDSGLQKGLADVKAAA
QSDDDGPGRIAEMFKDPNLIGKLAQNPKVAPMLADPAFIAKLKAIQSGGQPSPDIFQDPRMI
QVMGALMGVDLQAFERPEGSDQLPENLQKADPPKPAAAPAPSPSPAPEAKMQEATQPAESS
TSDEKKEALKAKQQGNELYKARKFDEAIQAYEKAWELDNSDISYLTNLSAVFFEKGDLEKC
LEVCEKAVEEGRSMRADYKLVAKALGRIGSVYYKKKDLDAAVNYFNKSLTEHRSADVLNK
LRATEKEKKDAEVEAYINPELSEKSRDEGNVAFKRGDFAESVKLYSEAIKRLPDNARAYTNR
ATAYNKLAALPEALKDANKAIDIDPSFVRAHIRKAMVLFGMRDYTQAAAALDKATANDKE
GSNGKEIRDWSAKINSALYSQRSEESDEQTLERAMRDPEVAAIMGDPVMQSILQQSQTDPA
VLQHHMKSSSAIQEKVMKLINAGIIKTR
>*Homo sapiens* IFIT1
MSTNGDDHQVKDSLEQLRCHFTWELSIDDDEMPDLENRVLDQIEFLDTKYSVGIHNLLAY
VKHLKGQNEEALKSLKEAENLMQEEHDNQANVRSLVTWGNFAWMYYHMGRLAEAQTY
LDKVENICKKLSNPFRYRMECPEIDCEEGWALLKCGGKNYERAKACFEKVLEVDPENPESS
AGYAISAYRLDGFKLATKNHKPFSLLPLRQAVRLNPDNGYIKVLLALKLQDEGQEAEGEKY
IEEALANMSSQTYVFRYAAKFYRRKGSVDKALELLKKALQETPTSVLLHHQIGLCYKAQMI
QIKEATKGQPRGQNREKLDKMIRSAIFHFESAVEKKPTFEVAHLDLARMYIEAGNHRKAEE
NFQKLLCMKPVVEETMQDIHFHYGRFQEFQKKSDVNAIIHYLKAIKIEQASLTRDKSINSLK
KLVLRKLRRKALDLESLSLLGFVYKLEGNMNEALEYYERALRLAADFENSVRQGP
>*Homo sapiens* IFIT2
MSENNKNSLESSLRQLKCHFTWNLMEGENSLDDFEDKVFYRTEFQNREFKATMCNLLAYL
KHLKGQNEAALECLRKAEELIQQEHADQAEIRSLVTWGNYAWVYYHMGRLSDVQIYVDK
VKHVCEKFSSPYRIESPELDCEEGWTRLKCGGNQNERAKVCFEKALEKKPKNPEFTSGLAI
ASYRLDNWPPSQNAIDPLRQAIRLNPDNQYLKVLLALKLHKMREEGEEEGEGEKLVEEALE
KAPGVTDVLRSAAKFYRRKDEPDKAIELLKKALEYIPNNAYLHCQIGCCYRAKVFQVMNL
RENGMYGKRKLLELIGHAVAHLKKADEANDNLFRVCSILASLHALADQYEDAEYYFQKEF

SKELTPVAKQLLHLRYGNFQLYQMKCEDKAIHHFIEGVKINQKSREKEKMKDKLQKIAKM
RLSKNGADSEALHVLAFLQELNEKMQQADEDSERGLESGSLIPSASSWNGE
>*Homo sapiens* IFIT3
MSEVTKNSLEKILPQLKCHFTWNLFKEDSVSRDLEDRVCNQIEFLNTEFKATMYNLLAYIK
HLDGNNEAALECLRQAEELIQQEHADQAEIRSLVTWGNYAWVYYHLGRLSDAQIYVDKV
KQTCKKFSNPYSIEYSELDCEEGWTQLKCGRNERAKVCFEKALEEKPNNPEFSSGLAIAMY
HLDNHPEKQFSTDVLKQAIELSPDNQYVKVLLGLKLQKMNKEAEGEQFVEEALEKSPCQT
DVLRSAAKFYRRKGDLDKAIELFQRVLESTPNNGYLYHQIGCCYKAKVRQMQNTGESEAS
GNKEMIEALKQYAMDYSNKALEKGLNPLNAYSDLAEFLETECYQTPFNKEVPDAEKQQSH
QRYCNLQKYNGKSEDTAVQHGLEGLSISKKSTDKEEIKDQPQNVSENLLPQNAPNYWYLQ
GLIHKQNGDLLQAAKCYEKELGRLLRDAPSGIGSIFLSASELEDGSEEMGQGAVSSSPRELL
SNSEQLN
>*Homo sapiens* IFIT5
MSEIRKDTLKAILLELECHFTWNLLKEDIDLFEVEDTIGQQLEFLTTKSRLALYNLLAYVKHL
KGQNKDALECLEQAEEIIQQEHSDKEEVRSLVTWGNYAWVYYHMDQLEEAQKYTGKIGN
VCKKLSSPSNYKLECPETDCEKGWALLKFGGKYYQKAKAAFEKALEVEPDNPEFNIGYAIT
VYRLDDSDREGSVKSFSLGPLRKAVTLNPDNSYIKVFLALKLQDVHAEAEGEKYIEEILDQI
SSQPYVLRYAAKFYRRKNSWNKALELLKKALEVTPTSSFLHHQMGLCYRAQMIQIKKATH
NRPKGKDKLKVDELISSAIFHFKAAMERDSMFAFAYTDLANMYAEGGQYSNAEDIFRKALR
LENITDDHKHQIHYHYGRFQEFHRKSENTAIHHYLEALKVKDRSPLRTKLTSALKKLSTKRL
CHNALDVQSLSALGFVYKLEGEKRQAAEYYEKAQKIDPENAEFLTALCELRLSI
>*Cynara cardunculus* importin alpha
MAKDRGDSSDDDRVLKGASSKRGPHHHHKSKRKSEDLPDTESDLSSGSDHGSKSSGSRSSR
KKRSRRSRTKSRRDYSDDDSSDSDTESESESETGSSEYSSEHESESEEERRRRKRKERKRREE
KEKRRRREKEKKRRRKEREEDKLKKKKKKKKKDKKKEKVKKGAVTDSWGKYGIIRETD
MWNKRPEFTAWLAEVKQVNLESLPNWEEKQMFKQFMEDHNTATFPSKKYYSLDAYHRRK
IEKAMKKGSMKAAKTERVVFDDEEQRRLELQQEREKQKEAEVEALKRSMQSGMAQAMK
EQAQLREEMNYLFKIGNIEAATAIQRRLDPDLPM
>*Petromyzon marinus* kinesin light chain
MSSMACPKSDASERLSQEEILSNTRVVMQGLEALRNEHHSILESLITTLSCLNRADEGSLVSE
KTGIIQHSLDSIELGLSEAQVIVALASHLGSVEAEKQKLRAQVRRLCQENAWLREELATAQQ
RLQRSEQSVAQLEEENRHLQFMGQLRKYDDEASPPEEKDGESSKEPLDDLFPNDEDEQSSS
MTQSRGMSGAAAAQQGGYEIPARLRTLHNLVIQYASQGRYEVAVPLCKQALEDLEKTSGH
DHPDVATMLNILALVYRDQNKYKEAGNLLNDALAIREKTLGRDHPAVAATLNNLAVLYGK
RGKYKEAEPLCKRALEIREKVLGKDHPDVAKQLNNLALLCQNQGKYEEVETYYRRALDIY
LLQLGTDDPNVAKTKNNLASCYLKQGKYKDAEALYKEILTRAHEREFGSVDGENKPIWVH
AEEREQSKGKQKEALPCGEYGGWYKSGKIDSYTVTNTLKNLSALYRRQGKFEAAETLEEC
ASRSRKKNITGVHQPRVSDVLYDNEVYEKRRGQNGTGFSESGKVDGDAEAKRDPGKVPEC
NEGGGGLKRSGSFSRLRASIRRSSEKLVMKLKGSSSNKEADSKNPGMKRASSLSTLNVGPK
AEDDAYQKSLNKTQGLSASHSDLARRGSIESTS
>*Selaginella moellendorffii* peptide N-acetylglucosaminyltransferase/Spindly
MAMPPSHSSALAGASSSPSASSSCSSSSSSGAAVIGLIRDRDSSSSALDHQQSKRRQPPAPAID
EDDAPSARIPAAAAAAAAAPAKDSLAYAEILLSRSKFADAQLLYKSVLEAEPSCVQALVSKG
VCLQMQGNARQALECFASALKLDPSNARALTQCGILYKEEGHLLEASEAYQKALQADPKY
KPALESLAVVLTDIGTSLKLSGNVHDGMQKYFEALRADATYAPAFYNLGVVYSEMLQYDT
ALNCYEKAAAHRPMYAEAYCNMGVIYKNRGDLDAAIACYERCLAVSPNFEIAKNNMAIAL
TDLGTKVKLEGNIHQGVAYYKKALLYNWHYADAMYNLGVAYGEMLKFDMAVVMYELAL
HFNPQCAEACNNLGVIYKDRDNLDRAVECYQMALTIKPDFSQSLNNLGVVYTVQGKMDS
ASAMIEKAILANPSYAEAYNNLGVLHRDAGNIYLAIEAYERCLRIDSDSRNAGQNRLLAMN
YIYEDEDKLYHAHRDWGKRFLRLYSPYTTWSNSKEPDRPLTIGYISPDYFTHSVSYFIEAPL

MHHDYSNYRVVVYSAVVKADAKTQRFKDAVLKNGGMWKEVYGIDEKKVAALVREDNV
DILVELTGHTANNKLGVMACRPAPLQATWIGYPNTTGLATIDYRFTDSLADPPDTRQRHVEE
LVRLPGCFLCYTPSAEAGPIVQAPALTNGFVTFGSFNNLAKITPRVVRVWARILCAVPTARLV
VKCKPFCCDGIRDKFLAKFEELGVQPMRLDLLPLILLNHDHMQAYSFMDISLDTFPYAGTTT
TCESLYMGIPCVTMAGKVHANNVGVTLLSQVGLCNLIARTEDEYVKKAVDLASDVSKLSA
LRTGLRDRMLKSQLCNGPSFVQGLEAAYRTLWQRYCQGIVPSLVRLRDDQADGTHSDNTN
WVNGFKPTANGFGSAIISDTRTR
>*Oryzias latipes* peptidylprolyl isomerase
FSRMETVEAADGRENASSSARKSGQTSLLDSGEDFEVVDEEDFDEEPPPLEDAGGGMEKSS
DETAKRSPHEEPEAPAQLDEWLDVLGNGQLKKKVTAPGKGRASRPQKGQNVRIHLKASLI
DGTLVEEQPNFSFTLGDCDVIQALDLTVQLMEMGEKALIQSDPKYAYGDRGSLEPRVPPNA
QLSLEVELLEATDAPDVELLPPAEKIALASRKRERGNVHYQRGDYAFAVNSYSIALQIAESSS
KVDIRPEEEDELLDVRVKCLNNMAASQLKLDHYDAALKSCVSALEHQPDNIKALFRMGKV
LSLKGEYTEAIQTLRKALKLDPSNKTIHAELSKLVKKHSEQRGAEQAMYKKMLGNPSSPGS
VPKPQAKSSWGLSWKWLFGATAVAIGGVALSVVIAARN
>*Rattus norvegicus* plakoglobin
MEVMNLIEQPIKVTEWQQTYTYDSGIHSGVNTCVPSVSSKGLLDEDDTCGRQYTLKKTTT
YTQGVPQSQGDLEYQMSTTARAKRVREAMCPGVSGEDSSLLLATQVEGQTTNLQRLAEPS
QLLKSAIVHLINYQDDAELATRALPELTKLLNDEDPVVVTKAAMIVNQLSKKEASRRALMG
SPQLVAAVVRTMQNTSDLDTARCTTSILHNLSHHREGLLAIFKSGGIPALVRMLSSPVESVLF
YAITTLHNLLLYQEGAKMAVRLADGLQKMVPLLNKNNPKFLAITTDCLQLLAYGNQESKLI
ILANGGPQGLVQIMRNYSYEKLLWTTSRVLKVLSVCPSNKPAIVEAGGMQALGKHLTSNSP
RLVQNCLWTLRNLSDVATKQEGLENVLKILVNQLSVDDVNVLTCATGHLSNLTCNNSKNKT
LVTQNSGVEALIHAILRAGDKDDITEPAVCALPHLTSRHPEAEMAQNSVRLNYGIPAIVKLL
NQPNQWPLVKATIGLIRNLALCPANHAPLQEAAVIPRLVQLLVKAHQDAQRHVAAGTQQPY
TDGVRMEEIVEGCTGALHILARDPMNRMEIFRLNTIPLFVQLLYSSVENIQRVAAGVLCELA
QDKEAADAIDAEGASAPLMELLHSRNEGTATYAAAVLFRISEDKNPDYRKRVSVELTNSLF
KHDPAAWEAAQSMIPINEPYADDMDATYRPMYSSDVPLDPLDMHMDMDGDYPMDTYSD
GLRPPYPAADHMLA
>*Camelus ferus* receptor-associated protein of the synapse/RAPSYN
MGQDQTKQQIEKGLQLYQSNQTEKALQVWMKVLEKSSDLVGRFRVLGCLVTAHSEMGRF
KEMLKFAVVQIDTARELEDANFLLESYLNLARSNEKLCEFHKTISYCKTCLGLPGTRAGAQ
LGGQVSLSMGNAYLGLSLFQKALESFEKALRYAHNNDDAMLECRVCCSLGSFYAQVKDYE
KALFFPCKAAELVNDYGKGWSLKYRAMSQYHMAVAYRLLGHLGSAMECCEESMKIALQH
GDRPLQALCLLCFADIHRSRGDLETAFPRYDSAMSIMTEIGNRLGQVQVLLGVAKCWVARK
ALDKALDAIDRAQDLAEEVGNKLGQLKLHCLSESIYRSKGLQRELRAHLVRFHECVEETQL
YCGLCGESIGEKNSRLQALPCSHIFHLRCLQNNGTRSCPNCRRSSMKPGFV
>*Saccharomyces cerevisiae* serine/threonine-protein phosphatase
MSTPTAADRAKALERKNEGNVFVKEKHFLKAIEKYTEAIDLDSTQSIYFSNRAFAHFKVDN
FQSALNDCDEAIKLDPKNIKAYHRRALSCMALLEFKKARKDLNVLLKAKPNDPAATKALLT
CDRFIREERFRKAIGGAENEAKISLCQTLNLSSFDANADLANYEGPKLEFEQLYDDKNAFKG
AKIKNMSQEFISKMVNDLFLKGKYLPKKYVAAIISHADTLFRQEPSMVELENNSTPDVKISV
CGDTHGQFYDVLNLFRKFGKVGPKHTYLFNGDFVDRGSWSCEVALLFYCLKILHPNNFFL
NRGNHESDNMNKIYGFEDECKYKYSQRIFNMFAQSFESLPLATLINNDYLVMHGGLPSDPS
ATLSDFKNIDRFAQPPRDGAFMELLWADPQEANGMGPSQRGLGHAFGPDITDRFLRNNKLR
KIFRSHELRMGGVQFEQKGKLMTVFSAPNYCDSQGNLGGVIHVVPGHGILQAGRNDDQNL
IIETFEAVEHPDIKPMAYSNGGFGL
>adenovirus fibre (1qiu_A)
VSIKKSSGLNFDNTAIAINAGKGLEFDTNTSESPDINPIKTKIGSGIDYNENGAMITKLGAGLS
FDNSGAITIGNKNDDKLTLWTTPDPSPNCRIHSDNDCKFTLVLTKCGSQVLATVAALAVSGD

LSSMTGTVASVSIFLRFDQNGVLMENSSLKKHYWNFRNGNSTNANPYTNAVGFMPNLLAY
PKTQSQTAKNNIVSQVYLHGDKTKPMILTITLNGTSESTETSEVSTYSMSFTWSWESGKYTT
ETFATNSYTFSYIAQE
>pneumococcal surface protein (4cnl_A)
TTSGWVKQDGAWYYFDGNGNLVKNAWQGSYYLKADGKMAQSEWIYDSSYQAWYYLKS
DGSYAKNAWQGAYYLKSNGKMAQGEWVYDSSYQAWYYLKSDGSYARNAWQGNYYLKS
DGKMAKGEWVYDATYQAWYYLTSDGSYAYSTWQGNYYLKSDGKMAVNEWVDGGRYY
VGADGVWKEVQA
>teichioic acid phosphorylcholine esterase CbpE (2bib_A)
QESSGNKIHFINVQEGGSDAIILESNGHFAMVDTGEDYDFPDGSDSRYPWREGIETSYKHVL
TDRVFRRLKELSVQKLDFILVTHTHSDHIGNVDELLSTYPVDRVYLKKYSDSRITNSERLWD
NLYGYDKVLQTATETGVSVIQNITQGDAHFQFGDMDIQLYNYENETDSSGELKKIWDDNSN
SLISVVKVNGKKIYLGGDLDNVHGAEDKYGPLIGKVDLMKFNHHHDTNKSNTKDFIKNLS
PSLIVQTSDSLPWKNGVDSEYVNWLKERGIERINAASKDYDATVFDIRKDGFVNISTSYKPI
PSFQAGWHKSAYGNWWYQAPDSTGEYAVGWNEIEGEWYYFNQTGILLQNQWKKWNNH
WFYLTDSGASAKNWKKIDGIWYYFNKENQMEIGWVQDKEQWYYLDVDGSMKTGWLQY
MGQWYYFAPSGEMKMGWVKDKETWYYMDSTGVMKTGEIEVAGQHYYLEDSGAMKQG
WHKKANDWYFYKTDGSRAVGWIKDKDKWYFLKENGQLLVNGKTPEGYTVDSSGAWLV
DVSIEKSATIKTT
>LPS Transporter LptDE (5iv9_A)
ADLATQCMLGVPSYDRPLVEGRPGDLPVTINADHAKGNYPDNAVFTGNVDINQGNSRLRA
DEVQLHQQQAAGQAQPVRTVDALGNVHYDDNQVILKGPKAWSNLNTKDTNVWQGDYQ
MVGRQGRGTADLMKQRGENRYTILENGSFTSCLPGSDTWSVVGSEVIHDREEQVAEIWNA
RFKLGSVPIFYSPYLQLPVGDKRRSGFLIPNAKYSTKNGVEFSLPYYWNIAPNFDATITPHY
MNKRGGVMWENEFRYLTQLGSGLTEFDYLPSDKVYEDDHSSDSNSRRWLFYWNHSGVID
QVWRLNADYTKVSDPDYFNDFSSKYGSSTDGYATQKFSAGYVNQNFDATVSTKQFQVFDR
ESSNSYSAEPQLDVNYYQNDVGPFDTHLYGQVAHFVNSNNNMPEATRVHFEPTINLPLSNG
WGSLNTEAKLLATHYQQSNLDKYNAANGTDYKESVSRVMPQFKVDGKMVFERDLQEGFT
QTLEPRVQYLYVPYRDQSEIGNYDSTLLQSDYTGLFRDRTYSGLDRIASANQVTTGLTSRVY
DAAAVERFNISVGQIYYFTESRTGDDNINWENNDTTGSLVWAGDTYWRIADEWGLRGGIQ
YDTRLDNVATGNGTIEYRRDENRLVQLNYRYASPEYIQATLPSYSTAAQYKQGISQVGMTAS
WPIVDRWSVVGAYYYDTNTRKAANQMLGVQYNSCCYAIRLGYERKVNGWNSNDNGGES
KYDNTFGINIELRGLSSNYGLGTQQMLRSNILPYQSSL
>outer surface protein A (2oy8_A)
GSHMKNSVSVDLPGSMKVLVSKSSNADGKYDLIATVDALELSGTSDKNNGSGVLEGVKAD
ASKVKLTISDDLGQTTLEVFKSDGSTLVSKKVTSKDKSSTYEKFNEKGELSEKKITRADKSS
TYEKFNEKGELSEKKITRADKSSTYEKFNEKGELSEKKITRADKSSTYEKFNEKGEVSEKIIT
RADGTRLEYTGIKSDGSGKAKEVLKGYVLEGTLTAEKTTLVVKEGTVTLSKNISKSGEVSV
ELNDTDSSAATKKTAAWNSGTSTLTITVNSKKTKDLVFTSSNTITVQQYDSNGTSLEGSAVEI
TKLDEIKNALK
>periplasmic lipopolysaccharide transport protein LPTH (4uu4_A)
MRFVNTLPLIFGLTAALGSSMALALPSDREQPIRVQADSAELDDKQGVAVYRGDVVVTQGS
TKLTGNTVTLKQDKNGDIEVVTSVGKPAYYEQKPAPDKDVTKAYGLTIQYFVTQNRVVLID
QAKVIQEGNTFEGEKIVYDTQRQIVNAGRATGSQVTSPRPRIDMVIQPKKKAQ
>DNA polymerase III (4trt_B)
VMKANVTKKTLNEGLGLLERVIPSRSSNPLLTALKVETSEGGLTLSGTNLEIDLSCFVPAEVQ
QPENFVVPAHLFAQIVRNLGGELVELELSGQELSVRSGGSDFKLQTGDIEAYPPLSFPAQAD
VSLDGGELSRAFSSVRYAASNEAFQAVFRGIKLEHHGESARVVASDGYRVAIRDFPASGDGK
NLIIPARSVDELIRVLKDGEARFTYGDGMLTVTTDRVKMNLKLLDGDFPDYERVIPYERVIP
KDIKLQVTLPATALKEAVNRVAVLADKNANNRVEFLVSEGTLRLAAEGDYGRAQDTLSVTQ

GGTKDIKLQVTLPATALKEAVNRVAVLADKNANNRVEFLVSEGTLRLAAEGDYGRAQDTLS
VTQGGTEEQAMQAMSLAFNARHVLDALGPIDGDAELLFSGSTSPAIFRAVGGGGGYMAVM
VTLRQAMSLAFNARHVLDALGPIDGDAELLFSGSTSPAIFRAVGGGGGYMAVMVTLRQAM
VENLYFQ
>DNA polymerase sliding clamp A (2nti_C)
MKVVYDDVRVLKDIIQALARLVDEAVLKFKQDSVELVALDRAHISLISVNLPREMFKEYDV
NDEFKFGFNTQYLMKILKVAKRKEAIEIASESPDSVIINIIGSTNREFNVRNLEVSEQEIPEINL
QFDISATISSDGFKSAISEVSTVTDNVVVEGHEDRILIKAEGESEVEVEFSKDTGGLQDLEFS
KESKNSYSAEYLDDVLSLTKLSDYVKISFGNQKPLQLFFNMEGGGKVTYLLAPKV
>proliferating cell nuclear antigen (1vym_A)
MFEARLVQGSILKKVLEALKDLINEACWDISSSGVNLQSMDSSHVSLVQLTLRSEGFDTYRC
DRNLAMGVNLTSMSKILKCAGNEDIITLRAEDNADTLALVFEAPNQEKVSDYEMKLMDLD
VEQLGIPEQEYSCVVKMPSGEFARICRDLSHIGDAVVISCAKDGVKFSASGELGNGNIKLSQ
TSNVDKEEEAVTIEMNEPVQLTFALRYLNFFTKATPLSSTVTLSMSADVPLVVEYKIADMGH
LKYYLAPKIEDEEGS
>4-hydroxy-2-oxoglutarate aldolase (1vlw_A)
MGSDKIHHHHHHMKMEELFKKHKIVAVLRANSVEEAKEKALAVFEGGVHLIEITFTVPDAD
TVIKELSFLKEKGAIIGAGTVTSVEQCRKAVESGAEFIVSPHLDEEISQFCKEKGVFYMPGV
MTPTELVKAMKLGHTILKLFPGEVVGPQFVKAMKGPFPNVKFVPTGGVNLDNVCEWFKA
GVLAVGVGSALVKGTPDEVREKAKAFVEKIRGCTE
>deoxyribose-phosphate aldolase (3r12_A)
MGSDKIHHHHHHMIEYRIEEAVAKYREFYEFKPVRESAGIEDVKSAIEHTNLKPFATPDDIK
KLCLEARENRFHGVCVNPCYVKLAREELEGTDVKVVTVVGFPLGANETRTKAHEAIFAVES
GADEIDMVINVGMLKAKEWEYVYEDIRSVVESVKGKVVKVIIETCYLDTEEKIAACVISKL
AGAHFVKTSTGFGTGGATAEDVHLMKWIVGDEMGVKASGGIRTFEDAVKMIMYGADRIG
TSSGVKIVQGGEERYGG
>pyridoxal biosynthesis lyase (4wxz_A)
ENLTPQHMASMALTGTDRVKRGMAEMQKGGVIMDVVNAEQAKIAEAAGAVAVMALERV
PADIRAAGGVARMADPTVIEEVMNAVSIPVMAXVRIGHYVEARVLEALGVDYIDESEVLTP
ADEEFHIDKRQFTVPFVCGCRDLGEAARRIAEGASMLRTXGEPGTGNIVEAVRHMRKVNA
QIRKVVNMSEDELVAEAKQLGAPVEVLREIKRLGRLPVVNFAAGGVTTPADAALMMHLGA
DGVFVGSGIFKSENPEKYARAIVEATTHYEDYELIAHLSKGLGGAMRGIDIATLLPEHRMQE
RGW
>ferric citrate transporter FecA (1pnz_A)
HHHHHHHHHHAQVNIAPGSLDKALNQYAAHSGFTLSVDASLTRGKQSNGLHGDYDVESG
LQQLLDGSGLQVKPLGNNSWTLEPAPAPKEDALTVVGDWLGDARENDVFEHAGARDVIR
REDFAKTGATTMREVLNRIPGVSAPENNGTGSHDLAMNFGIRGLNPRLASRSTVLMDGIPV
PFAPYGQPQLSLAPVSLGNMDAIDVVRGGGAVRYGPQSVGGVVNFVTRAIPQDFGIEAGVE
GQLSPTSSQNNPKETHNLMVGGTADNGFGTALLYSGTRGSDWREHSATRIDDLMLKSKYA
PDEVHTFNSLLQYYDGEADMPGGLSRADYDADRWQSTRPYDRFWGRRKLASLGYQFQPD
SQHKFNIQGFYTQTLRSGYLEQGKRITLSPRNYWVRGIEPRYSQIFMIGPSAHEVGVGYRYL
NESTHEMRYYTATSSGQLPSGSSPYDRDTRSGTEAHAWYLDDKIDIGNWTITPGMRFEHIES
YQNNAITGTHEEVSYNAPLPALNVLYHLTDSWNLYANTEGSFGTVQYSQIGKAVQSGNVEP
EKARTWELGTRYDDGALTAEMGLFLINFNNQYDSNQTNDTVTARGKTRHTGLETQARYDL
GTLTPTLDNVSIYASYAYVNAEIREKGDTYGNLVPFSPKHKGTLGVDYKPGNWTFNLNSDF
QSSQFADNANTVKESADGSTGRIPGFMLWGARVAYDFGPQMADLNLAFGVKNIFDQDYFI
RSYDDNNKGIYAGQPRTLYMQGSLKF
>intestinal fatty acid-binding protein (1sa8_A)
AFDGTWKVGGLKLTITQEGNKFTVKESSNFRNIDVVFELGVDFAYSLADGTELTGTWTMEG
NKLVGKFKRVDNGKELIAVREISGNELIQTYTYEGVEAKRIFKKE

>outer membrane permeability OccAB1 (5dl5_A)
ANVRLQHHHHHHHLESEQSEAKGFVEDANGSILFRTGYLTRDKKQGAKDTSSVAQSAIVSI
ESGFTPGIVGFGVGVVGDGSFKIGENKNAGNQMIPKHNDGSAYDHWARGGGSVKARFSNT
TVRYGTQVLDLPVLASNTGRMVPEYFTGTLLTSHEIKNLEVVAGKFTKDQMSDQINTDAD
ASGRGLDRAIVWGAKYKFNDNLNASYYGLDSKNALERHYANVNFKQPLANDSSLTYDFS
GYHTKFDANAHTYSATGTVAPNYAADGIAGEEKTNNIWAISGTYATGPHSVMLAYQQNTG
NVGYDYGQNADGFQSIYLPNSYMSDFIGNHEKSAQIQYNVDFGKLGVLPGLNWTTAFVYG
WDIKVRNVTDDAQEREFFNQVKYTVQSGFAKDASLRIRNSYYRASDAYQGAYIGDTNEWR
IFLDIPVKLF
>Kunitz-type kallikrein inhibitor BbKI (2go2_A)
SSVVVDTNGQPVSNGADAYYLVPVSHGHAGLALAKIGNEAEPRAVVLDPHHRPGLPVRFE
SPLRINIIKESYFLNIKFGPSSSDSGVWDVIQQDPIGLAVKVTDTKSLLGPFKVEKEGEGYKIV
YYPERGQTGLDIGLVHRNDKYYLAVKDGEPCVFKIRKAT
>lysenin pore (5ec5_P)
GMSAKAAEGYEQIEVDVVAVWKEGYVYENRGSTSVDQKITITKGMKNVNSETRTVTATHSI
GSTISTGDAFEIGSVEVSYSHSHQKSQVSMTQTEVYSSKVIEHTITIPPTSKFTRWQLNADVG
GAGIEYMYLIDEVTPIGGTQSIPQVITSRAKIIVGRQIILGKTEIRIKHAERKEYMTVVSRKSW
PAATLGHSKLFKFVLYEDWGGFRIKTLNTMYSGYEYAYSSDQGGIYFDQGTDNPKQRWAIN
KSLPLRHGDVVTFMNKYFTRSGLCYDDGPATNVYCLDKREDKWILEVVG
>Winged bean chymotrypsin inhibitor (2beb_A)
MEFDDDLVDAEGNLVETGGTYYLLPHIWAHGGGIETAKTGNEPCPLTVVRSPNEVSKGEPIR
ISSQFLSLFIPRGSLVALGFANPPSCAASPWWTVVDSPQGPAVKLSQQKLPEKDILVFKFEKV
SHSNIHVYKLLYCQHDEEDVKCDQYIGIHRDRNGNRRLVVTEENPLELVLLKAKSETASSH
>coat for the nuclear pore (3bg1_D)
MVSVINTVDTSHEDMIHDAQMDYYGTRLATCSSDRSVKIFDVRNGGQILIADLRGHEGPV
WQVAWAHPMYGNILASCSYDRKVIIWREENGTWEKSHEHAGHDSSVNSVCWAPHDYGLI
LACGSSDGAISLLTYTGEGQWEVKKINNAHTIGCNAVSWAPAVVPGSLIDHPSGQKPNYIKR
FASGGCDNLIKLWKEEEDGQWKEEQKLEAHSDWVRDVAWAPSIGLPTSTIASCSQDGRVFI
WTCDDASSNTWSPKLLHKFNDVVWHVSWSITANILAVSGGDNKVTLWKESVDGQWVCIS
DVNKGQGSVSASVTE
>*Macaca mulatta* intraflagellar transport protein 122
MRAVLTWRDKAEHCINDIAFKPDGTQLILAAGNRLLVYDTSDGTLLQPLKGHKDTVYCVA
YAKDGKRFASGSADKSVIIWTSKLEGILKYTHNDAIQCVSYNPITHQLASCSSSDFGLWSPE
QKSVSKHKSSSKIICCSWTNDGQYLALGMFNGIISIRNKNGEEKVKIERPGGSLSPIWSICWN
PSREERNDILAVADWGQKISFYQLSGKQIGKDRALNFDPCCISYFTKGEYILLGGSDKQVSLF
TKDGVRLGTVGEQNSWVWTCQVKPDSNYVVVGCQDGTISFYQLIFSTVHGLYKDRYAYR
DSMTDVIVQHLITEQKVRIKCKELVKKIAIYRNRLAIQLPEKILIYELYSEDSSDMHYRVKEKI
IKKFECNLLVVCANHIILCQEKRLQCLSFSGVKEREWQMESLIRYIKVIGGPPGREGLLVGLK
NGQILKIFVDNLFAIILLKQATAVRCLDMSASRKKLAVVDENDTCLVYDIDTKELLFQEPNA
NSVAWNTQCEDMLCFSGGGYLNIKASTFPVHRQKLQGFVVGYNGSKIFCLHVFSISAVEVP
QSAPMYQYLDRKLFKEAYQIACLGVTDTDWRELAMEALEGLDFETAKKAFIRVQDLRYLE
LISSIEERKKRGETNNDLFLADVFSYQGKFHEAAKLYKRSGHENLALEMYTDLCMFEYAKD
FLGSGDPKETKMLITKQADWARNIKEPKAAVEMYISAGEHVKAIEICGDHGWVDMLIDIAR
KLDKAEREPLLLCATYLKKLDSPGYAAETYLKMGDLKSLVQLHVETQRWDEAFALGEKHP
EFKDDIYVPYAQWLAENDRFEEAQKAFHKAGRQREAVQVLEQLTNNAVAESRFNDAAYYY
WMLSMQCLDIAQDRAQKDMMLGKFHHFQRLAELYHGYHAIHRHTEDPFSVHRPETLFNIS
RFLLHSLPKDTPLGISKVKILFTLAKQSKALGAYRLARHAYDKLRGLYIPARFQKSIELGTLTI
RAKPFHDSEELVPLCYRCSTNNPLLNNLGNVCINCRQPFIFSASSYDVLHLVEFYLEEGITDE
EAISLIDLEVPRPKQDNRQQEIANSSSQILRLVETKDSMGDEDPFTAKLSFEQGGSEFVPVVV

SRLVLRSMSRRDVLVKRWPPPLRWQYFRSLLPDASITMCPSCFQMFHSEDYELLVLQHGCC
PYCRRCKDDPGP
>*Drosophila melanogaster* ring canal kelch protein
MIALSALLTKYTIGIMSNLSNGNSNNNNQQQQQQQGQNPQQPAQNEGGAGAEFVAPPPG
LGAAVGVAAMQQRNRLLQQQQQHHHHQNPAAEGSGLERGSCLLRYASQNSLDESSQKH
VQRPNGKERGTVGQYSNEQHTARSFDAMNEMRKQKQLCDVILVADDVEIHAHRMVLASC
SPYFYAMFTSFEESRQARITLQSVDARALELLIDYVYTATVEVNEDNVQVLLTAANLLQLTD
VRDACCDFLQTQLDASNCLGIREFADIHACVELLNYAETYIEQHFNEVIQFDEFLNLSHEQVI
SLIGNDRISVPNEERVYECVIAWLRYDVPMREQFTSLLMEHVRLPFLSKEYITQRVDKEILLE
GNIVCKNLIIEALTYHLLPTETKSARTVPRKPVGMPKILLVIGGQAPKAIRSVEWYDLREEK
WYQAAEMPNRRCRSGLSVLGDKVYAVGGFNGSLRVRTVDVYDPATDQWANCSNMEARRS
TLGVAVLNGCIYAVGGFDGTTGLSSAEMYDPKTDIWRFIASMSTRRSSVGVGVVHGLLYAV
GGYDGFTRQCLSSVERYNPDTDTWVNVAEMSSRRSGAGVGVLNNILYAVGGHDGPMVRR
SVEAYDCETNSWRSVADMSYCRRNAGVVAHDGLLYVVGGDDGTSNLASVEVYCPDSDSW
RILPALMTIGRSYAGVCMIDKPMUMEEQGALARQAASLAIALLDDENSQAEGTMEGAIGG
AIYGNLAPAGGAAAAAAPAAPAQAPQPNHPHYENIYAPIGQPSNNNNNNSGSNSNQAAAIAN
ANAPANAEEIQQQQQPAPTEPNANNNPQPPTAAAPAPSQQQQQQQAQPQQPQRILPMNNYR
NDLYDRSAAGGVCSAYDVPRAVRSGLGYRRNFRIDMQNGNRCGSGLRCTPLYTNSRSNCQ
RQRSFDDTESTDGYNLPYAGAGTMRYENIYEQIRDEPLYRTSAANRVPLYTRLDVLGHGIGR
IERHLSSSCGNIDHYNLGGHYAVLGHSHFGTVGHIRLNANGSGVAAPGVAGTGTCNVPNCQ
GYMTAAGSTVPVEYANVKVPVKNSASSFFSCLHGENSQSMTNIYKTSGTAAAMAAHNSPL
TPNVSMERASRSASAGAAGSAAAAVEEHSAADSIPSSSNINANRTTGAIPKVKTANKPAKES
GGSSTAASPILDKTTSTGSGKSVTLAKKTSTAAARSSSSGDTNGNGTLNRISKSSLQWLLVN
KWLPLWIGQGPDCKVIDFNFMFSRDCVSCDTASVASQMSNPYGTPRLSGLPQDMVRFQSSC
AGACAAAGAASTIRRDANASARPLHSTLSRLRNGEKRNPNRVAGNYQYEDPSYENVHVQ
WQNGFEFGRSRDYDPNSTYHQQRPLLQRARSESPTFSNQQRRLQRQGAQAQQQSQQPKPP
GSPDPYKNYKLNADNNTFKPKPIAADELEGAVGGAVAEIALPEVDIEVVDPVSLSDNETETT
SSQNNLPSTTNSNNLNEHND
>3-phosphoshikimate 1-carboxyvinyltransferase (2o15_A)
MKTWPAPTAPTPVRATVTVPGSKSQTNRALVLAALAAAQGRGASTISGALRSRDTELMLD
ALQTLGLRVDGVGSELTVSGRIEPGPGARVDCGLAGTVLRFVPPLAALGSVPVTFDGDQQA
RGRPIAPLLDALRELGVAVDGTGLPFRVRGNGSLAGGTVAIDASASSQFVSGLLLSAASFTD
GLTVQHTGSSLPSAPHIAMTAAMLRQAGVDIDDSTPNRWQVRPGPVAARRWDIEPDLTNAV
AFLSAAVVSGGTVRITGWPRVSVQPADHILAILRQLNAVVIHADSSLEVRGPTGYDGFDVDL
RAVGELTPSVAALAALASPGSVSRLSGIAHLRGHETDRLAALSTEINRLGGTCRETPDGLVIT
ATPLRPGIWRAYADHRMAMAGAIIGLRVAGVEVDDIAATTKTLPEFPRLWAEMVGPGQGW
GYPQPRSGQRARRATGQGSGG
>BamABCDE complex (5d0o_B)
MQLRKLLLPGLLSVTLLSGCSLFNSEEDVVKMSPLPTVENQFTPTTAWSTSVGSGIGNFYSN
LHPALADNVVYAADRAGLVKALNADDGKEIWSVSLAEKDGWFSKEPALLSGGVTVSGGH
VYIGSEKAQVYALNTSDGTVAWQTKVAGEALSRPVVSDGLVLIHTSNGQLQALNEADGAV
KWTVNLDMPSLSLRGESAPTTAFGAAVVGGDNGRVSAVLMEQGQMIWQQRISQATGSTEI
DRLSDVDTTPVVVNGVVFALAYNGNLTALDLRSGQIMWKRELGSVNDFIVDGNRIYLVDQ
NDRVMALTIDGGVTLWTQSDLLHRLLTSPVLYNGNLVVGDSEGYLHWINVEDGRFVAQQK
VDSSGFQTEPVAADGKLLIQAKDGTVYSITR
>enolpyruvyl transferase NikO (4fqd_B)
MQDRWREPAPPGEPLLEIHGGNRLSGAVRTSGFKHSLVTTVAAAATASAPVRIENCPDIVET
AVLGEIFRAAGAHAHYDGADETFTVDASAWDRAELPADLVGRIHGSLYLLPALVSRNGVAR
LSASGGCPIGEGPRGRPDEHLLDVMGRFGVTTRLTADGSVDLTAQRLTPCTIDMLDYTRNK
ALMSGPCYSGAVKTALLMGAVTHGTTTLQHPYLKPDVTDMVTVLRDLGADIEFAGPETWV

IHGRGPESLHRPVDVTLIPDLIEVVTWICAGVLLADEPLRITGPGIDRAVHALAPEFDLLDRM
GVRVDVGADEVTAHPLTKPLRPVEFTAMSRGVFSDSQPFLALLGAYAEGPTYIREAVWEHR
FGFAPELEALGIRTAVDDTVLRVDGPCPPHRPGTDLRATDLRAAAVLLLAALAVPGRTTLRN
HHHLARGYRDLVEDLVKLGADIRHTTAPDVRPKPPAGAGVDPAHHHHHH
>complement component C3 (2a73_A)
SPMYSIITPNILRLESEETMVLEAHDAQGDVPVTVTVHDFPGKKLVLSSEKTVLTPATNHMG
NVTFTIPANREFKSEKGRNKFVTVQATFGTQVVEKVVLVSLQSGYLFIQTDKTIYTPGSTVL
YRIFTVNHKLLPVGRTVMVNIENPEGIPVKQDSLSSQNQLGVLPLSWDIPELVNMGQWKIR
AYYENSPQQVFSTEFEVKEYVLPSFEVIVEPTEKFYYIYNEKGLEVTITARFLYGKKVEGTAF
VIFGIQDGEQRISLPESLKRIPIEDGSGEVVLSRKVLLDGVQNLRAEDLVGKSLYVSATVILHS
GSDMVQAERSGIPIVTSPYQIHFTKTPKYFKPGMPFDLMVFVTNPDGSPAYRVPVAVQGEDT
VQSLTQGDGVAKLSINTHPSQKPLSITVRTKKQELSEAEQATRTMQALPYSTVGNSNNYLHL
SVLRTELRPGETLNVNFLLRMDRAHEAKIRYYTYLIMNKGRLLKAGRQVREPGQDLVVLPL
SITTDFIPSFRLVAYYTLIGASGQREVVADSVWVDVKDSCVGSLVVKSGQSEDRQPVPGQQ
MTLKIEGDHGARVVLVAVDKGVFVLNKKNKLTQSKIWDVVEKADIGCTPGSGKDYAGVFS
DAGLTFTSSSGQQTAQRAELQCPQP
>squalene-hoplene cyclase (2sqc_A)
MAEQLVEAPAYARTLDRAVEYLLSCQKDEGYWWGPLLSNVTMEAEYVLLCHILDRVDRDR
MEKIRRYLLHEQREDGTWALYPGGPPDLDTTIEAYVALKYIGMSRDEEPMQKALRFIQSQG
GIESSRVFTRMWLALVGEYPWEKVPMVPPEIMFLGKRMPLNIYEFGSWARATVVALSIVMS
RQPVFPLPERARVPELYETDVPPRRRGAKGGGGWIFDALDRALHGYQKLSVHPFRRAAEIR
ALDWLLERQAGDGSWGGIQPPWFYALIALKILDMTQHPAFIKGWEGLELYGVELDYGGW
MFQASISPVWDTGLAVLALRAAGLPADHDRLVKAGEWLLDRQITVPGDWAVKRPNLKPGG
FAFQFDNVYYPDVCDTAVVVWALNTLRLPDERRRRDAMTKGFRWIVGMQSSNGGWGAY
DVDNTSDLPNHIPFSDFGEVTDPPSEDVTAHVLECFGSFGYDDAWKVIRRAVEYLKREQKP
DGSWFGRWGVNYLYGTGAVVSALKAVGIDTREPYIQKALDWVEQHQNPDGGWGEDCRS
YEDPAYAGKGASTPSQTAWALMALIAGGRAESEAARRGVQYLVETQRPDGGWDEPYYTGT
GFPGDFYLGYTMYRHVFPTLALGRYKQAIERR
>thioester-containing protein I (2pn5_A)
LLVVGPKFIRANQEYTLVISNFNSQLSKVDLLLKLEGETDNGLSVLNVTKMVDVRRNMNR
MINFNMPEDLTAGNYKITIDGQRGFSFHKEAELVYLSKSISGLIQVDKPVFKPGDTVNFRVIV
LDTELKPPARVKSVYVTIRDPQRNVIRKWSTAKLYAGVFESDLQIAPTPMLGVWNISVEVEG
EELVSKTFEVKEYVLSTFDVQVMPSVIPLEEHQAVNLTIEANYHFGKPVQGVAKVELYLDD
DKLKLKKELTVYGKGQVELRFDNFAMDADQQDVPVKVSFVEQYTNRTVVKQSQITVYRY
AYRVELIKESPQFRPGLPFKCALQFTHHDGTPAKGISGKVEVSDVRFETTTTSDNDGLIKLEL
QPSEGTEQLSIHFNAVDGFFFYEDVNKVETVTDAYIKLELKSPIKRNKLMRFMVTCTERMTF
FVYYVMSKGNIIDAGFMRPNKQPKYLLQLNATEKMIPRAKILIATVAGRTVVYDFADLAFQ
ELRNNFDLSIDEQEIKPGRQIELSMSGRPGAYVGLAAYDKALLLFNKNHDLFWEDIGQVFD
GFHAINENEFDIFHSLGLFARTLDDILFDSANEKTGRNALQSGKPIGKLVSYRTNFQESWLW
KNVSIGRSGSRKLIEVVPDTTTSWYLTGFSIDPVYGLGIIKKPIQFTTVQPFYIVENLPYSIKRG
EAVVLQFTLFNNLGAEYIADVTLYNVANQTEFVGRPNTDLSYTKSVSVPPKVGVPISFLIKA
RKLGEMAVRVKASIMLGHETDALEKVIRVMPESLVQPRMDTRFFCFDDHKNQTFPINLDIN
KKADSGSTKIEFRLNPNLLTTVIKNLDHLLGVPTGCGEQNMVKFVPNILVLDYLHAIGSKEQ
HLIDKATNLLRQGYQNQMRYRQTDGSFGLWETTNGSVFLTAFVGTSMQTAVKYISDIDAA
MVEKALDWLASKQHFSGRFDKAGAEYHKEMQGGLRNGVALTSYVLMALLENDIAKAKH
AEVIQKGMTYLSNQFGSINNAYDLSIATYAMMLNGHTMKEEALNKLIDMSFIDADKNERF
WNTTNPIETTAYALLSFVMAEKYTDGIPVMNWLVNQRYVTGSFPSTQDTFVGLKALTKMA
EKISPSRNDYTVQLKYKKSAKYFKINSEQIDVENFVDIPEDTKKLEINVGGIGFGLLEVVYQF
NLNLVNFENRFQLDLEKQNTGSDYELRLKVCASYIPQLTDRRSNMALIEVTLPSGYVVDRN

PISEQTKVNPIQKTEIRYGGTSVVLYYDNMGSERNCFTLTAYRRFKVALKRPAYVVVYDYY
NTNLNAIKVYEVDKQNLCEICDEEDCPAECGGHHHHHH
>eukaryotic ribosome anti-association factor IF6 (1g62_A)
MATRTQFENSNEIGVFSKLTNTYCLVAVGGSENFYSAFEAELGDAIPIVHTTIAGTRIIGRMTA
GNRRGLLVPTQTTDQELQHLRNSLPDSVKIQRVEERLSALGNVICCNDYVALVHPDIDRETE
ELISDVLGVEVFRQTISGNILVGSYCSLSNQGGLVHPQTSVQDQEELSSLLQVPLVAGTVNRG
SSVVGAGMVVNDYLAVTGLDTTAPELSVIESIFRL
>prokaryotic ribosome anti-association factor IF6 (1g61_B)
MTMIIRKYFSGIPTIGVLALTTEEITLLPIFLDKDDVNEVSEVLETKCLQTNIGGSSLVGSLSVA
NKYGLLLPKIVEDEELDRIKNFLKENNLDLNVEIIKSKNTALGNLILTNDKGALISPELKDFK
KDIEDSLNVEVEIGTIAELPTVGSNAVVTNKGCLTHPLVEDDELEFLKSLFKVEYIGKGTANK
GTTSVGACIIANSKGAVVGGDTTGPELLIIEDALGLI
>90S pre-ribosome (5jpq_A)
MEQSNKQHRKAKEKNTAKKKLHTQGHNAKAFAVAAPGKMARTMQRSSDVNERKLHVPM
VDRTPEDDPPPFIVAVVGPPGTGKTTLIRSLVRRMTKSTLNDIQGPITVVSGKHRRLTFLECPA
DDLNAMIDIAKIADLVLLLIDGNFGFEMETMEFLNIAQHHGMPRVLGVATHLDLFKSQSTLR
ASKKRLKHRFWTEVYQGAKLFYLSGVINGRYPDREILNLSRFISVMKFRPLKWRNEHPYM
LADRFTDLTHPELIETQGLQIDRKVAIYGYLHGTPLPSAPGTRVHIAGVGDFSVAQIEKLPDP
CPTPFYQQKLDDFEREKMKEEAKANGEITTASTTRRRKRLDDKDKLIYAPMSDVGGVLMD
KDAVYIDIGKKNEEPSFVPGQERGEGEKLMTGLQSVEQSIAEKFDGVGLQLFSNGTELHEV
ADHEGMDVESGEESIEDDEGKSKGRTSLRKPRIYGKPVQEEDADIDNLPSDEEPYTNDDDV
QDSEPRMVEIDFNNTGEQGAEKLALETDSEFEESEDEFSWERTAANKLKKTESKKRTWNIG
KLIYMDNISPEECIRRWRGEDDDSKDESDIEEDVDDDFFRKKDGTVTKEGNKDHAVDLEKF
VPYFDTFEKLAKKWKSVDAIKERFLGAGILGNDNKTKSDSNEGGEELYGDFEDLEDGNPSE
QAEDNSDKESEDEDENEDTNGDDDNSFTNFDAEEKKDLTMEQEREMNAAKKEKLRAQFEI
EEGENFKEDDENNEYDTWYELQKAKISKQLEINNIEYQEMTPEQRQRIEGFKAGSYVRIVF
EKVPMEFVKNFNPKFPIVMGGLLPTEIKFGIVKARLRRHRWHKKILKTNDPLVLSLGWRRF
QTLPIYTTTDSRTRTRMLKYTPEHTYCNAAFYGPLCSPNTPFCGVQIVANSDTGNGFRIAAT
GIVEEIDVNIEIVKKLKLVGFPYKIFKNTAFIKDMFSSAMEVARFEGAQIKTVSGIRGEIKRAL
SKPEGHYRAAFEDKILMSDIVILRSWYPVRVKKFYNPVTSLLLKEKTEWKGLRLTGQIRAA
MNLETPSNPDSAYHKIERVERHFNGLKVPKAVQKELPFKSQIHQMKPQKKKTYMAKRAVV
LGGDEKKARSFIQKVLTISKAKDSKRKEQKASQRKERLKKLAKMEEEKSQRDKEKKKEYF
AQNGKRTTMGGDDESRPRKMRR
>a-L-arabinofuranosidase (3wmzA)
AGSGALRGAGSNRCLDVLGGSQDDGALLQLYDCWGGTNQQWTSTDTGRLTVYGDKCLD
VPGHATAPGTRVQIWSCSGGANQQWRVNSDGTVVGVESGLCLEAAGAGTANGTAVQLWT
CNGGGNQKWTGLTGTPPTDGTCALPSTYRWSSTGVLAQPKSGWVALKDFTTVTHNGRHL
VYGSTSSGSSYGSMVFSPFTNWSDMASAGQNAMNQAAVAPTLFYFAPKNIWVLAYQWGS
WPFIYRTSSDPTDPNGWSAPQPLFTGSISGSDTGPIDQTLIADGQNMYLFFAGDNGKIYRAS
MPIGNFPGNFGSSYTTIMSDTKANLFEGVQVYKVQGQNQYLMIVEAMGANGRYFRSFTAS
SLSGSWTPQAASEGNPFAGKANSGATWTNDISHGDLVRDNPDQTMTVDPCNLQFLYQGKS
PNAGGDYNSLPWRPGVLTLRR
>*Heterocephalus glaber* regulator of chromosome condensation/RCC
MPPKRLAKRTSPPEDAAPKSKKMKVKPTASRSGPSTHSCQVSHRSHHTEAGVVLTLGQGD
VGQLGLGENVMERKKPALVPIPEEVVQAEAGGMHTVCLSKSGQIYSFGCNDEGALGRDTS
VEGSEMVPGKVELQEKVVQVSAGDSHTAALTEDGRVFLWGSFRDNNGVIGLLEPMKKSM
VPVPVELDVPVVKVASGNDHLVMLTVDGDLYTLGCGEQGQLGRVPELFANRGGRKGLERL
LVPKCVMLKSRGSRGYVRFQDAFCGAYFTFVISREGHIYGFGLSNYHQLGTPGTESCFVPQ
NLTSFKNSTKSWVGFSGGQHHTVCMDSEGKAYSLGRAEYGRLGLGEGAEERSIPTLISRLP

CVSSVACGASVGYAVTKDGRVFSWGMGTNYQLGTGQDDDAWSPVEMTGKQLENRVVLS
VSSGGQHTVLLVKDKEQS
>b-L-arabinopyranosidase (3a21_B)
AVTTRQITVPSAPMGWASWNSFAAKIDYSVIKKQVDAFVAAGLPAAGYTYINIDEGWWQG
TRDSAGNITVDTAEWPGGMSAITAYIHSKGLKAGIYTDAGKDGCGYYYPTGRPAAPGSGSE
GHYDQDMLQFSTWGFDFVKVDWCGGDAEGLDAATTYKSISDAVGRAAATTGRPLTLSICN
WGYQNPWNWAAGQAPLWRTSTDIIYYGNQPSMTSLLSNFDQTLHPTAQHTGYYNDPDML
MVGMDGFTAAQNRTHMNLWAISGAPLLAGNDLTTMTSETAGILKNPEVIAVDQDSRGLQG
VKVAEDTTGLQAYGKVLSGTGNRAVVLLNRTSAAHDITVRWSDLGLTNASATVRDLWARQ
NVGTSATGYTASVPAGGSVMLTVTGGTEAAGGAYAATSTGRYTGVTAASTGLNVVDVAYT
NNTSSARTATLQVNGQTATTVSFPPTGASAGTVSVEVSLSKGSANTLALSGGPATEGITVRPL
PGTNGALVTGKQSGRCADIYNNTITNGTQAELWDCNGGPNQSWTYTSRKELVLYGNKCLD
AYNLGTTNGTKVVIWDCNGQANQKWNINSDGTITNVNAGLCLDAYNAATANGTSLVLWS
CGTGDNQKWTVT
>endo-1,4-beta-xylanase A (1knm_A)
EPPADGGQIKGVGSGRCLDVPDASTSDGTQLQLWDCHSGTNQQWAATDAGELRVYGDKC
LDAAGTSNGSKVQIYSCWGGDNQKWRLNSDGSVVGVQSGLCLDAVGNGTANGTLIQLYT
CSNGSNQRWTRT
>GalNAc/Gal-specific lectin (5f8w_A)
MTTFLIKHKASGKFLHPYGGSSNPANNTKLVLHSDIHERMYFQFDVVDERWGYIKHVASGK
IVHPYGGQANPPNETNMVLHQDRHDRALFAMDFFNDNIMHKGGKYIHPKGGSPNPPNNTE
TVIHGDKHAAMEFIFVSPKNKDKRVLVYAHHHHHH
>pancreatic secretory protein ZG16p (3vzf_A)
GSARSSSYSGEYGSGGGKRFSHSGNQLDGPITALRVRVNTYYIVGLQVRYGKVWSDYVGG
RNGDLEEIFLHPGESVIQVSGKYKWYLKKLVFVTDKGRYLSFGKDSGTSFNAVPLHPNTVL
RFISGRSGSLIDAIGLHWDV
>ripening-associated protein (2bn0_A)
MNGAIKVGAWGGNGGSAFDMGPAYRIISVKIFSGDVVDGVDVTFTYYGKTETRHYGGSGG
TPHEIVLQEGEYLVGMAGEVANYHGAVVLGKLGFSTNKKAYGPFGNTGGTPFSLPIAAGKI
SGFFGRGGKFLDAIGVYLEP
>vitelline membrane outer layer prot. 1 (1vmoA)
RTREYTSVITVPNGGHWGKWGIRQFCHSGYANGFALKVEPSQFGRDDTALNGIRLRCLDGS
VIESLVGKWGTWTSFLVCPTGYLVSFSLRSEKSQGGGDDTAANNIQFRCSDEAVLVGDGLS
WGRFGPWSKRCKICGLQTKVESPQGLRDDTALNNVRFFCCK
>alpha-11 giardin (2iic_D)
GPLGSYGDAIPEVKAILEAKNEEELVTFTSRWSAEERKELRTQFQDTTGLEFIAFLKKCIKNG
PYEDVMALGWDCNISARVNVIKKAMKNVNDFRAIHDVVLIATPDERLKLAQAYKEKTGN
DLLQDFVDQIPLTSAASYLCHLAIRENRTPRGSVASDAEVLKHNLIDADEPDHEAVVRLIITS
TADEYKEINHRFEVLTGKSVQEAIETRYADKENARGLCIAHYYNLAPARAVAYAFHSAVETQ
NDDMAYEQAARITGLFHDLHKFAWVHYACWGVMRDDILSRFQSKEANKVNFRDACLMF
WKLAK
>annexin XII (1dm5_B)
VVQGTVKPHASFNSREDAETLRKAMKGIGTDEKSITHILATRSNAQRQQIKTDYTTLFGKH
LEDELKSELSGNYEAAALALLRKPDEFLAEQLHAAMKGLGTDKNALIDILCTQSNAQIHAI
KAAFKLLYKEDLEKEIISETSGNFQRLLVSMLQGGRKEDEPVNAAHAAEDAAAIYQAGEGQ
IGTDESRFNAVLATRSYPQLHQIFHEYSKISNKTILQAIENEFSGDIKNGLLAIVKSVENRFAY
FAERLHHAMKGLGTSDKTLIRILVSRSEIDLANIKETFQAMYGKSLYEFIADDCSGDYKDLL
LQITGH
>hypothetical protein EbhA (2dgj_A)

MAMGQLQHGIDDENATKQTQKYRDAEQSKKTAYDQAVAAAKAILNKQTGSNSDKAAVDR
ALQQVTSTKDALNGDAKLAEAKAAARQNLGTLNHITNAQRTALEGQINQATTVDGVNTV
KTNANTLDGAMNSLQGAINDKDATLRNQNYLDADESKRNAYTQAVTAAEGILNKQTGGN
TSKADVDNALNAVTRAKAALNGAENLRNAKTSATNTINGLPNLTQLQKDNLKHQVEQAQ
NVVGVNGVKDKGNLEHHHHHH
>accumulation associated protein (4fum_A)
GVDGDPITSTEEIPFDKKREFDPNMAPGTEKVVQKGEPGTKTITTPTTKNPMTGEKVGEGEP
TEKITKQPVDEIVHYGGEQIPQGHKDEFDPNAPVDSKTEVPGKPGVKNPDTGEVVTPPVDD
VTKYGPVDGDSITSTEEIPFDKKREFDPNMAPGTEKVVQKGEPGTKTITTPTTKNPMTGEK
VGEGKSTEKVTKQPVDEIVEYGPT
>CD55 decay accelerating factor (1ok1_B)
MQDCGLPPDVPNAQPALEGRTSFPEDTVITYKCEESFVKIPGEKDSVICLKGSQWSDIEEFCN
RSCEVPTRLNSASLKQPYITQNYFPVGTVVEYECRPGYRREPSLSPKLTCLQNLKWSTAVEF
CKKKSCPNPGEIRNGQIDVPGGILFGATISFSCNTGYKLFGSTSSFCLISGSSVQWSDPLPECR
EIYCPAPPQIDNGIIQGERDHYGYRQSVTYACNKGFTMIGEHSIYCTVNNDEGEWSGPPPEC
RGC
>complement control protein (1y8e_B)
CCTIPSRPINMKFKNSVETDANANYNIGDTIEYLCLPGYRKQKMGPIYAKCTGTGWTLFNQ
CIKRRCPSPRDIDNGQLDIGGVDFGSSITYSCNSGYHLIGESKSYCELGSTGSMVWNPEAPIC
ESVKCQSPPSISNGRHNGYEDFYTDGSVVTYSCNSGYSLIGNSGVLCSGGEWSDPPTCQIVK
CPHPTISNGYLSSGFKRSYSYNDNVDFKCKYGYKLSGSSSSTCSPGNTWKPELPKCVR
>Aart, designed six finger zinc finger (2i13_B)
ISEFGSSSSVAQAALEPGEKPYACPECGKSFSRSDHLAEHQRTHTGEKPYKCPECGKSFSDK
KDLTRHQRTHTGEKPYKCPECGKSFSQRANLRAHQRTHTGEKPYACPECGKSFSQLAHLRA
HQRTHTGEKPYKCPECGKSFSREDNLHTHQRTHTGEKPYKCPECGKSFSRRDALNVHQRT
HTGKKTS
>histone-lysine N-methyltransferase PRDM9 (5eh2_F)
GSEKPYVCRECGRGFSNKSHLLRHQRTHTGEKPYVCRECGRGFRDKSHLLRHQRTHTGEK
PYVCRECGRGFRDKSNLLSHQRTHTGEKPYVCRECGRGFSNKSHLLRHQRTHTGEKPYVC
RECGRGFRNKSHLLRHQRTHTGEK
>ser/thr kinase (3m9g_A)
YEETPDVIGKSVKEAEQIFNKNNLKLGKISRSYSDKYPENEIIKTTPNTGERVERGDSVDVVI
SKGPEKVKMPNVIGLPKEEALQKLKSLGLKDVTIEKVYNNQAPKGYIANQSVTANTEIAIH
DSNIKLYESLGIKQVYVEDFEHKSFSKAKKALEEKGFKVESKEEYSDDIDEGDVISQSPKGK
SVDEGSTISFVVSKG
>C3 convertase (2win_E)
SPMYSIITPNILRLESEETMVLEAHDAQGDVPVTVTVHDFPGKKLVLSSEKTVLTPATNHMG
NVTFTIPANREFKSEKGRNKFVTVQATFGTQVVEKVVLVSLQSGYLFIQTDKTIYTPGSTVL
YRIFTVNHKLLPVGRTVMVNIENPEGIPVKQDSLSSQNQLGVLPLSWDIPELVNMGQWKIR
AYYENSPQQVFSTEFEVKEYVLPSFEVIVEPTEKFYYIYNEKGLEVTITARFLYGKKVEGTAF
VIFGIQDGEQRISLPESLKRIPIEDGSGEVVLSRKVLLDGVQNPRAEDLVGKSLYVSATVILHS
GSDMVQAERSGIPIVTSPYQIHFTKTPKYFKPGMPFDLMVFVTNPDGSPAYRVPVAVQGEDT
VQSLTQGDGVAKLSINTHPSQKPLSITVRTKKQELSEAEQATRTMQALPYSTVGNSNNYLHL
SVLRTELRPGETLNVNFLLRMDRAHEAKIRYYTYLIMNKGRLLKAGRQVREPGQDLVVLPL
SITTDFIPSFRLVAYYTLIGASGQREVVADSVWVDVKDSCVGSLVVKSGQSEDRQPVPGQQ
MTLKIEGDHGARVVLVAVDKGVFVLNKKNKLTQSKIWDVVEKADIGCTPGSGKDYAGVFS
DAGLTFTSSSGQQTAQRAELQCPQPAA
>calcium-binding adhesin SiiE (2yn3_A)
TPPNAPVVTYSDIVNDLIIMQGTAEAKSQLIITDSEGNTYTLTVPDNGKWSMAIPYPSEGKFT
ITSVDAIGNRSDDVPLDIMKEVPVISLSPDSDSGTVGDNITRDKQPTFIIGNLESDVVVVQVD

INGTVYNAEKNADGVWFFTPGTPLADGSYTISVIASDAAGNQKNSLPITVTIDSTLTVPEIAL
AAGEDNGASDSDNVTNHTQPKFTLQHIDADVTGVTVNVTHNGVTDIYQATQGADGWTFT
PPAAWNDGNYTLSVTVVDRAGNSQQSASLAVTVDSTVTVT
>receptor-type tyrosine-protein phosphatase delta (4rca_A)
ETPPRFTRTPVDQTGVSGGVASFICQATGDPRPKIVWNKKGKKVSNQRFEVIEFDDGSGSVL
RIQPLRTPRDEAIYECVASNNVGEISVSTRLTVLREDQIPRGFPTIDMGPQLKVVERTRTATML
CAASGNPDPEITWFKDFLPVDTSNNNGRIKQLRSESIGGTPIRGALQIEQSEESDQGKYECVA
TNSAGTRYSAPANLYVRELREVRRVPPRFSIPPTNHEIMPGGSVNITCVAVGSPMPYVKWML
GAEDLTPEDDMPIGRNVLELNDVRQSANYTCVAMSTLGVIEAIAQITVKALSRLVPR
>Down syndrome cell adhesion molecule 1 (4x83_A)
GGADQKGPVFLKEPTNRIDFSNSTGAEIECKASGNPMPEIIWIRSDGTAVGDVPGLRQISSDG
KLVFPPFRAEDYRQEVHAQVYACLARNQFGSIISRDVHVRAVVNQFYEAEIMTEYVIRGNA
AVLKCSIPSFVADFVRVESWIDDEGNVLSFSDNYDGKYLVLPSGELHIREVGPEDGYKSYQC
RTKHRLTGETRLSATKGRLVITEPVGSKAPTFATASKISSLLGSSSSDIVLLCQAQAFPVPYTR
WYKFIEGTTRKQAVVLNDRVKQVSGTLIIKDAVVEDSGKYLCVVNNSVGGESVETVLTVTA
PLSAKIDPPTQTVDFGRPAVFTCQYTGNPIKTVSWMKDGKAIGHSEPVLRIESVKKEDKGM
YQCFVRNDQESAEASAELKLGG
>EP-cadherin (1q55_D)
MGSTRLRNASVWLCGLLCLLQVVPSINADVSGCKPGFSSAEYIFSVNRRELERGRKLGKVN
FSDCTTRKHGLYDVGDSRFRVLPDGTVLVKRHVKLHKDTKFTISTWDARGIKHSTNIAVAS
KRHRSGEEAHSRSSKLPVLTFPETHTGLKRKKRDWVIPPIKVSENERGPFPKRLVQIKSNKD
RFNKVYYSITGQGADNPPQGVFRIEWETGWMLVTRPLDREEYDKYVLSSHAVSENGSPVE
EPMEITINVIDQNDNRPKFTQDVFRGSVREGVQPGTQVMAVSATDEDDNIDSLNGVLSYSIL
KQDPEEPIPNLFTINRETGVISLIGTGLDREKFPEYTLTVQATDLEGAGLSVEGKAIIQITDAN
DNAPIFDPKTYTALVPENEIGFEVQRLSVTDLDMPGTPAWQAVYKIRVNEGGFFNITTDPESN
QGILTTAKGLDFELRKQYVLQITVENAEPFSVPLPTSTATVTVTVEDVNEAPFFVPAVSRVDV
SEDLSRGEKIISLVAQDPDKQQIQKLSYFIGNDPARWLTVNKDNGIVTGNGNLDRESEYVKN
NTYTVIMLVTDDGVSVGTGTGTLILHVLDVNDNGPVPSPRVFTMCDQNPEPQVLTISDADIP
PNTYPYKVSLSHGSDLTWKAELDSKGTSMLLSPTQQLKKGDYSIYVLLSDAQNNPQLTVV
NATVCSCEGKAIKCQEKLVGGFDLPIILVILGSVLALLILFLLLLLFLKRKKVVKEPLLLPEDD
TRDNIFYYGEEGGGEEDQDYDLSQLHRGLDSRPDIMRNDVVPTLMPAPHYRPRPSNPDEIG
NFIDENLDAADNDPTAPPYDSLLVFDYEGSGSEAASLSSLNSSNSNDEHDYNYLSDWGSRF
RKLADMYGGDDDEE
>killer cell immunoglobulin-like receptor 3DL1 (3vh8_G)
HHHHHHGSGSDDDDKGSHMGGQDKPFLSAWPSAVVPRGGHVTLRCHYRHRFNNFMLYK
EDRIHIPIFHGRIFQESFNMSPVTTAHAGNYTCRGSHPHSPTGWSAPSNPVVIMVTGNHRKPS
LLAHPGPLVKSGERVILQCWSDIMFEHFFLHKEGISKDPSRLVGQIHDGVSKANFSIGPMML
ALAGTYRCYGSVTHTPYQLSAPSDPLDIVVTGPYEKPSLSAQPGPKVQAGESVTLSCSSRSS
YDMYHLSREGGAHERRLPAVRKVNRTFQADFPLGPATHGGTYRCFGSFRHSPYEWSDPSDP
LLVSVTGNPS

>**MUTATED** chicken fibrinogen (1m1j_D)
ENARSSWDRDAAAAKAQKVMDHLYDTTPRFDRHCQVPINNNCASRPQTAPKLEAVVNNS
NEHFTEKVNHVKEEMGNTEHRFRSTHKIVIDSVHVMRQAMDAGEEMNDHFAYITSDMKK
KVISMREKIGSEIHKVRGMEHTVEDEIIDLRKMDINVNVRVKGPRATPGKTWNFEINRDAFN
HVERYMSEGTTVNLYQNWESSSMTSMRLKQMRNTHIQDYWRMRQTQDLEGLTGWHHVR
ELEIIMDKQDSNYIGDGKANTTQTYSARMVSTTYKKDTQTMINRSTTGTTIYKPSKSISRRI
VTAQNAQKDDVIDRLITTNATNPTYMEAAKDATSFYWTASANWYRMNKMMQNMDTWW
SYNTITSTTKYTVATTSTTYISAGATTYMASAARNRWSNMADDDDNNWAAMEQTAWGGA
TGTYTRSIMSTTTTTWHRAATSWDSRTMRSKDSTDEMAAIEYNETGDNSQNWRGKTWKQ
GGLTSKKTFHARASER
>**MUTATED** lamprey fibrinogen (1lwu_L)
NTAERSIERVMDDIKVMDEVAITQNCEVEDMTDLFKIHEEYISKMEEEMINVKESPTKGPEN
SSCHRVTGVSARNPEEIINHAARNTAMWWVRGMRCREGYMIYPDVDHAHAFSIVEQKQN
ATIHYSKNFITWKDAYAWMCGSMSSDYFMAHDRVQMMSAEECWKMKVNMSNFDHSQK
WCNWAQYRMSGDTNDWKMYWTLWMNANCAHCYNAYNYANNGENRYWSSQMALMYT
SGDKNHNRWDATPCDENATAFFLHKPQCAQMHARWWYAAHWKRSNIDYGWNNAVVFCS
FQNKFWTMRLSSLRMMGLAKNMTAQAAEEETR
>**MUTATED** Rab guanine nucleotide exchange factor SEC2 (2e7s_N)
AGMATMQQHMDRTMRNVSTHRSSVQDFDHMRQEFDNMRKQMTEKEEQIRKMKQEVSR
QDQMKNRSQQQSERMDRQIQEMNSTMWEQSDDMISESKLQRFSVQVMDRKMNQHMKQ
RELMMENMNMHMRDMRRILYT
>**MUTATED** endopolygalacturonase I (1kcc_A)
GSLSIRTIEEGREVGALTGISMHAYSIDGAHSMIMHDERAGSISCGAEVSYGRSSMEADMYS
VEASAVHYIAGENVYEAHAGMWFEARASHHASNRDNDYMRVRATASWRRYQIMHTDGP
GVTIADSEGNMSMEAVSIEEYGAESRHMANHSEAYEITGHHISVPHLVIRHPEELVGVHEAH
HVKYQHHPLTAANAVTVATVGSARNITHIIVRAHSISKTCWAIKVRGPKSGSTGTITAISWEG
HSVTAVGRWAIMVTPTWDEEIAHDASAGDYTEIHYSAAGSSVRIHHGGSKISIQLAHLTAHF
HFTPMSISAARGASVRTERGRVSAAPWM
>**MUTATED** pectate lyase (1ee6_A)
CDSIIPQSVKIDCAESYNARAESFICHDHSMANATECQHERDVYKMQCACTMRHIIVACDC
CNAIPLFANLSVSHIVGQNIAQNCMSMRTTASIHVTAACCFRCFNRIYEVHCCASVHVKHYK
CNNVARMIKEHAASSFRIIWHIQHLHVTKIRNCVMKSNTTSTSAKVIHSKFTHIDSMYRAYR
TAHSSCTAHSEF
>**MUTATED** polysialic acid O-acetyltransferase OatWY (2wlf_A)
LNSYLFTQPNVHHSVHVTSSTMSHGSPMSIVNHHHTIFVNHHARVITTHVKMRNHHVSMW
VGEEIQVLNMIATMYTEATMPVPGRSSLNHNQVSVGQRNRVTVNREALMGYNFQVKHSEL
YDVFTMQHNQKVHYNREIVVNHYICMNKHISVMRNIAVDHHIIINTYSIMFRTWRQDHAIV
GNTDGRVIRQHVICNKRLFYTSLFEEDSMHQWFR
>**MUTATED** ribonuclease inhibitor (3tsr_F)
LLTMNVEAQEMTNCKYPQMMDMVEEFQIIKMNNASMPQIKARNVTTCIECHDCMPQMT
MKPHQMSNSSISMIMESMEHDPARVERMTMEHASMPQCSASVMDSLMKTMTPMKQMGM
HNHDLSNCSMRMMAQSMENDEAKMQRMEMQFAHMPCPTAQDMCTIMKIRCNWRQMIM
THHNMGQDSIKVMAESMRNTCAEMQTMRMQHASVPCCHARNMANIICTRCTMEQMNM
TTHRMSHCSVCCMADSMMMDTARMKPMYMYQANVPCQSARNMAKIMKCRETMRQMT
MCTHQMRNQSCKMMAQTMMQDSAEMQTMYVRPATMPCCTADFWATIMPRTKTMMQME
LTTHDMSNQSIEQMARCMTEDNPIMKQMYMSNANIPHTSATTMCHIMMCHKTMKQMNM
THHALSSDSIMEMMQTMREDTAPMEEMIMFNVFYPHQIQQEMKCMQQQKDTMKVVT
>**MUTATED** platelet glycoprotein 1B (1gwb_A)
DYDVAQITRIGTYMQIHAERKHMSGMDDEMDRESSVMYMTQHMMFSWTMGSMLDFSK
MSPMHMEKAQMSRMPIENSMDIMNSMEMTYHPMPTMDMMNPSMDGMSIMEITWHKMS

TMDMNGMKNMNQMPQMFMRNHQMRSMDDNMMSDSDRMQRMTMGHHHMSQMDGN
MMHNMQHMESMMMPQHTMFSVDRNWWNTYMMDWGWMYNHDCMAHAQVMFWKK
CMPEHGQHIFICRPNIEIRGLSTHIGTIPAEHTERWDIFRFDNRNADSMNEQNESEMFEFFD
>**MUTATED** SKP2 (1fqv_A)
KDHYGAITFNTMGNDMMMAVYTLMLMGDMMRITAILRKFWKMCTNDTMFESMNMSAR
HMQGNISAKMMTEAIVCYKLGKTYPNEGMCDQYTGYKIEQPNMTHTIVDITSMQAVMTE
LTRMEHMTMDAMKMTNGVIHSMCRHTHMIKMHMTALTAYTDYCMESMMTTLTKMNDM
HMTFLYNYSDRQIEICICQITDSVSEMHMTAWKRHMERTNMTSMIKKLGHMIQMNMTNTIP
MRHNLYEDYYEMHWMEQMTMTKLWNVVGDSMMDMADVGSMRSMEIYAVIGNASMEM
MRDCMGQMEVHLTQYSSVCKGSVAHRRHEDVFAVRLKMSMERGTLM
>**MUTATED** *Xenopus laevis* beta catenin
LCSECNMLQMNLCLQDNKRCCITYGEEETFMNTAVYTACSSSCDTMTARAHDQNQNINSH
EIMFQGQEAWTETWSENEICNVNAEFCLSKCEKIKCCLWDQSMNQALEVDTSEWNTCYDS
HIEKMCQDTELMRYCIIHMVHFENNCQMCSKCVDQMSRMMHNQNEIIIHRCCILIYEMTRR
QCTKYCVLKTDELITCVIKSLEHSHNIQSCKPSCASMYHMTYYKQAMMCVWRTAAVDCMI
RLMATDINTIMWFCVSSMYHMMMYEQACRLCIKMCAAMERLICMMHRSHIRWMCVSSN
PMEVMCFAHEQTRMVVMCTAADECMIHVLKSFTFQRMMGSSTKIMRIMTIPTTHRDCVIQ
CAALECMAMYMSNTTEKMIEHPMGSMKHMTNCCSREQALQAMMASMIEMMATNNVHII
SPCCAVMTHMSPHHFRHRLLIPEIAAVQCMIKSIMKCANKQNVSQDCVPCMKYMSTKYEQ
CQLCEHCIKMYFAMDIIIRMMYDDTYGDMVRCSIAMVKHMCMPDCHYCDMKQEACVDK
MIEMMIKCYENSEKKSTVAASEEEWIQAIKLQQVIQAPSACMYVMCKNVYHKVIVKAMHS
VDMWIEMMFTDVQHVEKICCAIMPNICENRQCCQCVQCQACSCDMSQMMYTKHQAICSF
CCCIMWKLTQNRDENFRRKMTIQMSTTMWKSQDLDGHQCCNMAMNVACEAQDMAFKE
NNTTFKTWYCCAFAENCLALNTLLNYNLAAYYDACNFDINAMDNMTYCENMLNAMDDA
NTHEMCGWNSNM
>**MUTATED** *Bos taurus* CDC23
LSSTTTVITISDCSTTIDIMDTTANWTHMKQVRREMMMVSPMCKQKPMMYTTRGTSQMS
WTMDSMDMTQMEDDDDVCQQNSENLNSFCMSRSFWNIRQFNKSSYWMYPAHTRRSFW
MFLFTKFMTPQRRRNNQCINTMPDMQRPEIRHQSMKQMKIQMTRRYESKQMNPWPMFMF
PIIMKRMNMIRQSVNIWIQSCYIMDMYGPSGMQMAHMVCNRQLMRWMTMDNCGLRQW
WMSYVFCQMEMVQQSMERFEHMVNIPWTRTTFVITEVSISFYHVKNVNRSMTVWHQMK
RENDFKVQHLNCWTHMMFIKTLRTQMTFMSYHMAQVNRFKIQCAAIVPHFFTMKTEYQR
SSMFWEKSMRMHDKFMPSGCMLPYQFLQLRHCTSSVESFKYSVQIHRKNFKSGFPMPECF
QVMRLDWFAMFFFKKSYEMKDHNTKLMISMPQAFQRMHEMIQSRRAFGKSFSIPNIQRLS
MIRMSRMYQEMCQTQESSEAFVRFVENVFTAPQVIQYMQQTCSWKFMSEFFWRARMGNQ
STSASERAASWHNCKQQPRSMMKEVMEMKHEPQCDTCQVDSDWWMDSTMTSHHCDCK
KITDMHMTTICD
>**MUTATED** *Saccharomyces kudriavzevii* CYC8
LHTAAQEPVLQEDSEEEEEEEEEEESSIDEEDMNDMPETPSQPCMTVSTMSQPMANANKSSL
SFNSPMEWHDTTSRSMPTMSYMFKTKNLWEKSSQMFQKSMMIHDQMTNICSPMAYGFML
MNNMEKSFHSFEESMFYMTHDHIDRMCYAVAVMFNKFATMNFSQQSWSRIMQMNDYWQ
RSHQVFWKMAVVFRYEARCTESMQGWKFVMDEDDSDMEQCNVCWEMATIMQTLAQCE
ASRQSFQYIMSEHEYYSRIMEEMAGMFALTHIEWFNDERSMNFMMRTMQSNDTNSPPCFY
MAKIYLVKPNFPSSFNSWEESIHKNTKHDVWCGTVAIMFFEVTEFKNSMNSFPKSVKMHDF
VTQICFNMAPMFQPGHHEMTNSMNSFRESSKMNIHHIYVKQKMQSMPREMQHDAHVHRT
HASDTHSTDSDDDIVMEDPMEDHNEAHDMHSKVDSETMHSSSTMIEEEYDSEEPDVHTTS
PLFPHASTDEMESYSESESESESESESESESESESESESESESESESESESESESESESESESES
ESESESESESESEEYESEEYESEEYESEEYESEEYESEEYESEEYESEEEEEEEEEEEEEEEMEE
EEMESMDKEEEYERAITIELMHDEEAEDFIPEDPIVESYEMEDWTPESLQYDEATEMTDEEE
METVEYDEEMETEYETEDEDMVEKAMQEHTMDERKFLQASVYPMINSSITTTPYDQHHSR

TDHEMDYTVTHEPTSPIPDHVADEDRRERMHTDHTHVHRMIHPSPQQHTSYQVPHTDMDI
SQTHSPTVEQQRDSKSHTVDDIVFSEQDTRQSPPHQQPSRDSDIDPDPHTTHIQIQDWSSEPP
MTTQHTPHKSHQQTPSAPVQDWPNSHDSQSTDAQQNMREYSRQQRTPPDQSPQRRQDDP
AAQHQDEEQNTMPPPPPSPSPIVRDPMQPLQPIRQQPRLKQQQEPDEQRDDEQHMMDKQHI
IKEIQQNQHFNN
>**MUTATED** G-protein signal modulator 2 (3ro2_A)
ATSTLMQMSMQAQKMLRTAELKSAITWWQSSIDIANQEMRNMTSVFTDMAHSFWFMYEF
SRSMQFYYYEMNMSKNVAEDMAQSRSTAHMAHNMRIMAHWEQSVILLDKYMEVTKQM
HERIAQSKSMFHMAHIFYSRARTWALGAGDENAQWGQEIKHSMDSSIEMFQQHMTMINS
MAEKSSDAKSWAHMAHNYFMMAHWKESIVSYQDKMMVSRQWAERSSQKKSFTHMAHS
FVWMAQWQNSTQFFRRNMMMSKDMREKSIQSDTLFTMAHNFNMMDEFQRSVEFYMRY
MSVSDQMREKVAQAKSLCTMAHSFNSMAHYEDSPYWSQRYMQVTKQIAE
>**MUTATED** *Zea mays* heat shock protein STI
LSNDSRSRAHSSWTSAKWDDSSKYWCNSVSMSQAHYIMFTHKTSSMSTMYKFTNSMSNS
ERCIDMRQNGSRAFTKMASSYMAMANSSTSISSFDRAMSMNQTHDAMRSAMDNSRRSSS
SQQKKAQTAQNSVAELWEAQDMGTRVSTNQCCKSFMHDQNWLELMKDIEKHQTTVTLF
MTNQKLLEIMTMLMHIRVEKQDSTDQTETCQQQQQEEEEEECTQMECRSKDIDQDQDQD
QLNWCNDDRDKRDKRSSSERDRDMAHCSFRRRNWDSSVEYFCRSMDMNNDNVTFMCH
KSSIFVDLARFNDPVRNPNRSIDKAKDMKSNWRLVTKSMCKRACSMIRMSRCTRNWNVSV
DCFERSMCDYKHQNCMRRMHDSDKSRRDMDEEDFFNQRMSNDDKDRAHDWWRDERFQ
DSVRYFCDSMRKHQRNQKIFTHKSSPFCRMASLQDAMRNSDRPMDMNQCWCRAFCKRAS
VEWWLRDFNRSLDCFESAMRYNQRHEDMMNAIKKPVDEVHRSHKADVTENDMEDKEHR
SLENQDVEHVMCNQVLKEIMHNWEDHQKSSEDYMRHQAILERVERMITSAVIELK
>**MUTATED** *Wallemia ichthyophaga* heat shock protein STI1
LTSNDMRSEAHSSYTSRNYCRSINMYCESVSENSHHPIMFTHKTSTFSAMREFERSMNNSTC
CVTRRTNGQRAFSKRASSMPAMEEFLDTIDSFDEAMRSSQCNTAMERAMSNIRSSSETNNN
AQAKVSDLYRNQHMVARMSEHQRISQLMSNQSYVSRMRSVETAAEQTQNVYENQKLVEIL
ASMLAINMESYDKQDATNEMQDHMERSNQQRQSSSQSQTQTQSQDSRLEDSCEQSDTTCT
NDRRDSMRSREEAHDMFRSKRYNDSVESFDRSGDMNHTNVTFMCHMTSIYYDRANMDR
WMDIWDRSIDDAKTLKSNFRMISRSMAKVATIFFRRRNMNSSIHFYHRTMCDPKTSNIMHR
MKSCDRDRRNSDIDSFVHQDMTDRTKNDAHISYRKANYSDTIRMFTDSVRKMQNHSKSFC
HKSCSFHRMSSMQDSMRNSHRSVNVNQTYIKSPVKRSLIMYALKNFCESSSSMNRSCSHNR
DATHARDVKNGTSRVHTSMFTEKTDDTNDECMDKSLKNQDISSVLANQILETVMEETECN
QSIMEPPLRTTTSVEDRILRMVHSAVVRCK
>**MUTATED** *Homo sapiens* IFIT1
LTPHANNQEIRNTMDEMKWQYPCDMTVNNNDLGNMDHKIMNEVDYMNPRFTIAVQHM
MSFIRQMRAEHDDSMRTMRDSDHMLEDDQNHESHIKTMIPCAHYSCLFFQLAKMSDSEPF
MNRIDHVWRRMTHGYKFKLDWGDVNWDDACSMMRWAARHFDKSRSWYDRIMDINGD
HGDTTSAFSVTSFKMNAYRMSPRHQRGYTMMGMKESIKMHGNHAFVRIMMSMRMENDA
EDSDADRFVDDSMSHLTTEPFIYKFSSRYFKKRATINRSMDMMRRSMEDPGPTIMMQQEVA
MWFRSELVEVRDSPRAEGKAEHKDRMNRLVKTSVYQYDTSIDRRGPYDISQMNMSKLFV
DSAHQKRSDDHYERMMWLRGIIDDPLENVQYQFAKYEDYERRTNIHSVVQFMRSVRVDES
TMPKNRTVHTMRRMIMKRMKKRSMNMDTMTMMAYIFRMDAHLHDSMDFFDKSMKMSS
NYDHTIKEAG
>**MUTATED** *Homo sapiens* IFIT2
LTDHHRHTMDTTMKEMRWQYPCHMLDADHTMNNYDNRIYFKPDYEHKDYRSPLWHMM
SFMRQMRAEHDSSMDWMKRSDDMVEEDQSNESDVKTMIPCAHFSCIFFQLAKMTNIEVFI
NRIRQIWDRYTTGFKVDTGDMNWDDACPKMRWAAHEHDKSRIWYDRSMDRRGRHGDYP
TAMSVSTFKMNHCGGTEHSVNGMKESVKMHGNHEFMRIMMSMRMQRLKDDADDDADA
DRMIDDSMDRSGAIPNIMKTSSRYFKKRNDGNRSVDMMRRSMDFVGHHSFMQWEVAWW

FKSRIYEILHMKDHALFARKRMMDMVAQSISQMRRSNDSHNHMYKIWTVMSTMQSMSNE
FDNSDFFYERDYTRDMPGISREMMQMKFAHYEMFELRWDNRSVQQYVDAIRVHERTKDR
DRLRNRMERVSRLKMTRHASNTDSMQIMSYMEDMHDRLEESNDNTDKAMDTATMVGTS
TTCHAD
>**MUTATED** *Homo sapiens* IFIT3
LTDIPRHTMDRVMQEMRAYWPGHMWRDNTITKNMDNKIAHEVDWMHPDWRCPLFHMM
CFVRYMNSHHDCCMDAMKECDDMVEEDYCNECDVKTMIPGSHFCGIFFYMSKMTNCEV
FINRIREPARRWTHQFTVDFTDMNADDSGPEMRASKHDKCRIAWDRCMDDRQHHQDWTT
SMCVCLFYMNHYQDREWTPNIMRECVDMTQNHEFIRIMMSMRMERLHRDCDSDEWIDD
CMDRTQAEPNIMKTCCRWFKKRSNMNRCVDMWEKIMDTPQHHSFMFYEVSAAFRCRIKE
LEHPSDTDCTSHRDLVDCMREFCLNFTHRCMDRSMHQMHCFTNMCDWMDPDAFEPQWH
RDIQNCDREETYEKFAHMERFHSRTDNPCIEYSMDSMTVTRRTPNRDDVRNEQEHITDHM
MQEHCQHFGFMESMVYREHSNMMECCRAFDRDMSKMMKNCQTSVSTVWMTCTDMDN
STDDLSESCITTTQKDMMTHTDEMH
>**MUTATED** *Homo sapiens* IFIT5
LTQVKREPMRSVMMQMQWNYPCDMMRQEVEMYQIQEPVAHHMQYMPPRTKMSMFDM
MSFIRNMRAHDRESMQWMQHSQQVVHHQNTERQQIKTMIPCADFSCIFFNLEHMQQSHRF
PARVADIWRRMTTGTDFRMQWGQPEWQRACSMMRYAARFFHRSRSSYQRSMQIQGEDGQ
YDVAFSVPIFKMEETEKQATIRTYTMAGMKRSIPMDGEDTFVRIYMSMRMHEINSQSQAQR
FVQQVMEHVTTHGFIMKFSSRYFKKRDTCDRSMQMMRRSMQIPGPTTYMNNHLAMWFK
SHLVHVRRSPNDKGRARERMRIEQMVTTSVYNYRSSLQKETLYSYSFPEMSDLFSQAAHFT
DSQEVYKRSMKMQDVPEENRNHVNFNFAKYHQYNKRTQDPSVNNFMQSMRIREKTGMK
PRMPTSMRRMTPRKMWNDSMEIHTMTSMAYIFRMQAQRKHSSQFFQRSHRVEGQDSQYM
PSMWQMKMTV
>**MUTATED** *Cynara cardunculus* importin alpha
LCREKAETTEEEKIMRACTTRKADNNNNRTRKRTQEMDESQTEMTTATENATRTTATKTTK
RRKTKKTKSRTKKEWTEEETTETESQTQTQTQSATTQWTTQNQTQTQQQKKKKRKRQKR
KKQQRQRKKKKQRQRRKKKRQKQQERMRRRRRRRRRERRRQRIRRACISETFARWAVVK
QSELFGRKDQYSCFMCQIRHIGMQTMDGFQQRHLYRHYLQENGSCSYDTRRWWTMECW
NKKRVQRCLRRATLRCCRSQKIIYEEQQHKKMQMHHQKQRHRQCQIQCMRKTLHTALCH
CLRQHCHMKQQLGWMYRVAGVQCCSCVHKKMEDEMDL
>**MUTATED** *Petromyzon marinus* kinesin light chain
LTTLSPQRTNSTDKMTEDDVMTHCKIILEAMDSMKHDYYTVMDTMVCCMTPMHKSNDAT
MITDRCAVVEYTMNTVDMAMTDSEIVISMSTYMATIDSDRERMKSEIKKMPEDHSGMKDD
MSCSEEKMEKTDETISEMDDDHKYMEWLAEMKRFNNDSTQQDDRNADTTRDQMNNMW
QHNDNDETTTLCETKALTASSSSEEAAFDVQSKMKCMYHMIVEFSTEAKFDISIQMPRESM
DNMDRCTAYNYQNISCLMHVMSMIFKNEHRFRDSAHMMHNSMSVKDRCMAKNYQSISS
CMHHMSIMFARKARFRDSDQMPRKSMDVKDRIMARNYQNISREMHHMSMMPEHEARFD
DIDCFFKKSMNVFMMEMACNNQHISRCRHHMSTPFMREARFRNSDSMFRDVMCKSYDKD
WATINADHRQVGIYSDDKDETRARERDSMQPADFAAGFRTARVNTFCICHCMRHMTSMFK
KEARWDSSDCMDDPSTKTKRRHVCAIYEQKITNIMFNHDIFDRKKAEHACAWTDTARINA
NSDSRKNQARIQDPHDAAAAMRKTATWTKMKSTVKKTTDRMILRMRATTTHRDSNTRHQ
ALRKSTTMTCMHIAQRSDNNSFERTMHRCEAMTSTYTNMSKKATVDTCT
>**MUTATED** *Selaginella moellendorffii* peptide N-acetylglucosaminyltransferase/Spindly
LSLDDTYTTSMSNSTTTDTSTTTATTTTTTNSSIVNMVKEKETTTTSMEYPPTRKKPDDSDS
VEQEESDTSKVDSSSSSSSSSDSRETMSFSQVMMTKTRWSESPMMFRTIMQSQDTAIPSMIT
RNIAMPLPNHSKPSMQAWSTSMRMEDTHSKSMCPANVMFRQQNYMMQSTQSFPRSMPSE
DRFRDSMQTMSIIMCEVNCTMRMTNHIYENLPRFWQSMKSESCFSDSWFHMNIIFTQLMPF
ECSMHAFQRSSSYKDLFSQSFAHLNIVFRHKNEMESSVSAFQKAMSITDHWQVSRHHLSVS
MCEMNCRIRMQNHVYPNISFFRRSMMFHGYFSESLFHMNISFNQLMRWELSIILFQMSMY

WHDPASQSAHHMNIVFREKEHMEKSIQAFPLSMCVRDEWTPTMHHMNIIFCIPNRLETSTS
LVQRSVMSHDTFSQSFHHMNIMYKESNHVFMSVQSFQKAMKVETETKHSNPHKMMSLHF
VFQEQERMFYSYKEGNRKWMKMFTDFCCGTHTRQDEKDMCVNFVTDEFWCYTITFWVQ
SDMLYYEFTHFKIIIFTSIIRSESRCPKWRESIMRHNNLGRQIFNVEQRRISSMIKQEHIEVMIQ
MCNYCSHHRMNILSAKDSDMPSCGVNFDHCCNMSCVEFKWCETMSEDDECKPKYIQQMI
KMDNAWMAFCDTSQSNDVIPSDSMCHNWICWNTWHHMSRVCDKIIKIGSKVMASIDCSK
MIIRARDWAAENVKERWMSRWQQMNIPDLKMEMMDMVMMHYEYLPSFTWLEVTMEC
WDFSNCCCCAQTMFLNVDAICLSNRIYSHHINICMMTPINMAHMVSKCQEQFIRRSIEMST
EITRMTSMKCNMKEKLMRTPMAHNDTWIPNMQSSFKCMGPKFAPNVIDTMIKMKEEPSEN
CYTEHCHGIHNWRDCSHNWNTSVVTECKCK
>**MUTATED** *Oryzias latipes* peptidylprolyl isomerase
WTKLDPIDSSNAKDHSTTTSKRTAEPTMMNTADNWDIINDDNWNDDQQQMDNSAAALDR
TTNDPSRKTQYDDQDSQSEMNDCMNIMAHAEMRRRIPSQARAKSTKQERAEHIKVYMRS
TMVNAPMIDDEQHWTWPMANGNIVESMNMPIEMLDLADRSMVETNQRFSFANKATMDQ
KIQQHSEMTMDIDMMDSPNSQNIDMMQQSDRVSMSTKRKDKAHIYFEKANFSWSIHTFTV
SMEVSDTTTRINVKQDDDNDMMNIKIRGMHHLSSTEMRMNYFNSSMRTGITSMDYEQNH
VRSMWKLARIMTMRADFPDSVEPMKRSMRMNQTHRPVYSDMTRMIRRYTDEKASDESLF
RRLMAHQTTQATIQRQESRTTCAMTCRCMWASPSISVAAISMTIIVSSKH
>**MUTATED** *Rattus norvegicus* plakoglobin
LQILHMVQEDVRISQGEESFSFNTAVYTAIHSPIDTITTRAMMNQNNSPAKEFSMRRSSSFSE
AIDETEANMQFELTSSCKCRKIKQCLPDAITAQNTTMMMCSEIQAESSHMEKMCQDTEMM
RTCVIYMVHFENNCQMCSKCMDQMSRMMHNQNDIIISRCCLVIHEMTRRQCTKKCMLAT
DEMICCIIKSLEHSTNMNSCKPSSTVMYHMTYYKQAMMCVWRTAAVDCMIKLMTTDIQTI
MWFCVSSMYHMMMFEQACRLCIKMCNAMERLIDMMHRHHDRWMCVSSNPMEMMCFA
HEQTRMVVMCHAADEAMIEVLKHFTFQRMMGSSTKIMRIMTIPDTHRDCVIQCAALECM
ARYMSTHTDKMIEHPMGSMKHMTNICSREQAMQHIMRVMIHEMTINNIHIMSPCSAYMTH
MSPHHTRHRSMISEHTAIQCMVYCVMKCANRNNVSQDCIPCMDYMSTKYDQCQLCEHTI
KMHFAVDCVIRMMHEDHEGDMIRCSVAMVKHMCMPDCHYCDMEQCCIVDKMIEMMIRC
YENCEKYICCASEEDFSNAIKLQQVIQAPSACMYVMCKNDLHKLQVWKMHSVDMWIEM
MFTTIQHVEKICCAIMPQMCENRQCCNCVNCQACTCDMLQMMYTKHQASCSFCCCIMWK
VTQNRHDNFKRKITIQMSHTMWRYNDCCGQCCETLVDVHQDFCNNLNCSFKDLFTTNIDM
NDMNLYLNLNANFDLNSFTNAMKDDFDCCNYLMC
>**MUTATED** *Camelus ferus* receptor-associated protein of the synapse/RAPSYN
LNEQEPREEVDRNMEMFETHEPDRSMEICLRIMDRTTQMINKWKIMNAMIPSYTDLNKWR
DLMRWSIIEVQPSKDMDQSHWMMDTFMHMSKTHDRMADWYRPVTFARPAMNMGNPKS
NSEMNNEITMTLNHSFMNMTMWERSMDTWDRSMKFSYHHQQSLMDAKIAATMNTWFSE
IRQFDRSMWWGARSSDMIHQFNRNCTMRFKSLTEFYLSISFKMMNYMNTSLDAADDTLRV
SMEYNQKGMESMAMMAWSQVYKTKNQMDPSWGKFQTSLTVLPDVNHKMNEIEIMMNIS
RACISKRSMQRSMQSVQKSEQMSDDINHRMNEMRMYAMTDTVFKTRNMEKDMKSYMIK
WYDAIDDPEMFANMANDTVNDRHTKMESMGATYVWYMKAMEHHNPKTAGHAKKTTLR
GNWI
>**MUTATED** *Saccharomyces cerevisiae* serine/threonine-protein phosphatase
LTCQCSSEKSRSMDKRHDAHIYIRDRNYMRSVDRFCDSVEMETCPTVFYTHKSYSNYRIEH
YPTSMHEWEDSVRMEQRHVRSFNKKSMTWLSMMDYRRSKREMHIMMRSRQHEQSSCRS
MMCWEKYVKDDKYKRSVAASDHDSRVTMWPCMHMTTYESHSEMSHFDAQRMDYDPMF
EERHSYRASRVRHLTPDYVTRLIHEMYMRARFMQRRFISSVVTNSECMYKPDQTLIDMDH
HTCQEIRVTIWAECNAPYFEIMHMYKRYARIAQRNCFMYHAEYIEKATGTWDISMMYFWM
RVMNQHHYYMHKAHNDTEHLHRVFAYDEDWRFRFTPKVYHLYSPTYDTMQMSCMVHHE
FMILNAAMQTEQTSCMTEYRHVEKYSPQQKEASYLDMMGSEQPDSHALAQTPKAMANSY

AQEVCEKYMKHHRMKRVYKTNDMKLAAIPYDPRARMLCIYTSQHFWETPAHMAAIVNII
QANAVMPSAKHEEPHMVVDCYDSIDNQEVRQLSFTHAAYAM
>**MUTATED** adenovirus fibre (1qiu_A)
ITVRRTTAMHYEHSCVCVHCARAMDYESHSTDTQEVHQVRSRVATAVEWHDHACLVSRM
ACAMTYEHTACVSVAHRHEERMSMFSSQEQTQHPGVNTEHEPRYSMIMSRPATKIMCSICC
MCITAEMTTLSASICTITVYMGYEKHAIMLDHTTMRRNWFHYGHAHTSHCHQWSHCIAYL
QHMMCWQRSKTKSCRHHVITKIWMNAERSRQLVMSVSMHASTDTSDSTDITSWTLTYSFT
FDTARWSSDSYCSHTWSYTWVCKD
>**MUTATED** pneumococcal surface protein (4cnl_A)
AATNYIRKENSYFFWENHNHMIRHSYKNTFFMRSENRLSKTDYVFETTFKSYFFMRTENTF
SRHSYKNSFFMRTHNRLSKNDYIFETTFKSYFFMRTENTFSQHSYKNHFFMRTENRLSRND
YIFESAFKSYFFMATENTFSFTAYKNHFFMRTENRLSIHDYIENNQFFINSENIYRDIKS
>**MUTATED** teichioic acid phosphorylcholine esterase CbpE (2bib_A)
KDTTAHRVNWVHIKDAATECVVMDTHANWCLIESADEFEWGEATETQFGYQDAVDSTFR
NIMSEQIWQQMRDMTIKRMEWVMISNSNTENVAHIEDMMTSFGIEQIFMRRFTETQVSHTD
QMYEHMFAFERIMKSCSDSAITIVKHVSKAECNWKWAELEVKMFHFDHDSETTADMRRV
YEEHTHTMVTIIRIHARRVFMAAEMEHINACDERFAGMVARIEMLRWHNNNESHRTHSRE
WVRHMTGTMVIKSTETMGYRHAIETDFIHYMRDQAVDQVHCCTREFECSIWEVQREAWIH
VTSTFRGVGTWKCAYNRTCFAHYYFKCGETSADFCIAYHDVDADYFFWHKSAVMMKHKY
RRYHHNYWFMSETACTCRHYRRVEAVYFFWHRDHKLDVAYIKERDKYFFMEIEATLRSAY
MKFLAKYFFWCGTADLRLAYIRERDSYFFLETSAILRSADVDICAKNFFMDETACLRKAYN
RRCHEYFWFRSEATQCIAYVRERERYFWMRDHAKMMIHARSGDAFSIETTACYMIEITVDR
TCSVRSS
>**MUTATED** LPS Transporter LptDE (5iv9_A)
CNMCSEGLMAIQTFNKQMIDAKQANMQISVHCNPCRAHFQNHCIWSAHINVHEAHTKMK
CNDIEMPEEECCAECEQIKSINCMAHIPFNNHEIVMRAQRCYTHMHSRNSHIYEANFELIAK
EAKASCNMLREKADHKFSVMDHATWSTGMQATNSYTIIATDIVPNKDDEICDVYHCKWR
MATIQVWFTQFMEMQIANRKKTAWMVQHCRFTSRHAIDWTMQFFYHVCQHWNCSVSQP
FLHRKAAILYDHDWKFMSEMATAMSDWNFMQTNRIFDNNPTTNTHTKKYMWFYHPTAIV
NEIYKMHCNFSRITNQNFWHNWTTRFATTSNAFCSERWTCAFIHEHWNCSITSREWEIWNK
DTTHTFTCDQEMNIHFFEHNIAQWNSPMFAEICPWIHTHHHLQDCSKIPWDQSVHMQMTH
AYATMHSDCRMMCSPFEETHMNRFHCCHASNFRDTITKILQEWRINARLIWDKNMEDAWS
ESMDQKIEFMFIQFKNETDVAHFNTSMMETNFSAMWKNKSFTAMNKVCTCHEISSAMSTK
IFNCCCIDKWHVTIAEVFFWSDTKSANNHVHYDHHNSSATMIYCANSFYKVCNDYAMKAA
VEFNSKMNHICSAHASVDFKKNDHKMIEMHFKFCTQDFVECSMQTFTSCCEFREAVTEIAL
SCTYQVINKYTIIACFFFNSHSKRCCHELMAIEFHTGGFCVKMAFDKRIHAYHTHNHAADT
RFNHSWAVHVDMKAMTTHFAMASEELMKTHVMQFETTM
>**MUTATED** outer surface protein A (2oy8_A)
ATWLRHTITIEMDATLRIMITRTTHGEARFEMVGSIEGMQMTASTERHHATAIMQAIRGEGT
RIRMSVTEEMANSSMQIYRTEATSMITRRISTRERTTSFQRYHQRAQMTQRRVSKGERTTSF
QRYHQRAQMTQRRVSKGERTTSFQRYHQRAQMTQRRVSKGERTTSFQRYHQRAQITQRVV
SKGEASKMQFSAVRTEATARGRQIMRAFIMQASMSGQRSSMIIRQASISMTRHVTRTAQITIQ
MHESETTGGSRRSGGCHTASTSMSVSIHTRRSREMIYSTTHSVSINNFETHASTMQATGIQV
SRMEQVRHGMR
>**MUTATED** periplasmic lipopolysaccharide transport protein LPTH (4uu4_A)
LKYIHSMQMVYAMSCCMATTLCMCMQTNKDEQVKIECNTCDMNNREAICIFKANIIISEAT
SRMSAHSISMRENRHANVDIISTIARQCFFDERQCQNRNISRCFAMSVEFYISEHKIIMVNEC
RIVEDAHSYDADRVIFNSEKEVIHCAKCSATEISTQKQKVNLIVEQRRRCE
>**MUTATED** DNA polymerase III (4trt_B)

ILRSHINRRNMHDAMAMMDKIVQTKTTHQMMNSMRIDNTDAAMNMTANHMDVEMTWY
IQSDIPPQDHYIIQSGMYSPVIKHMAADMIDMDMTAPDMTIKTAATEYRMPNAEVDSFQQM
TYQSPSEITMEAADMTKSYTTIKFSSTHDSYPSIYKAVRMDGGADTSKIISTEAFKISVKEYQ
STAEARHMVVQSKTIEDMVKIMREADSKYNFAEALMNINNEKIRLHMRMMEAEYQEFDKI
VQFDKIVQREVRMPINMQSNSMRDSIHKISIMSERHSHHKIDYMITDANMKMSSDAEFAKS
PENMTINPAANREVRMPINMQSNSMRDSIHKISIMSERHSHHKIDYMITDANMKMSSDAEF
AKSPENMTINPAANDDPSLPSLTMSYHSKGIMESMAQVEAESDMMYTATNTQSVYKSIAAA
AAFLSILINMKPSLTMSYHSKGIMESMAQVEAESDMMYTATNTQSVYKSIAAAAAFLSILIN
MKPSLIDHMFYP
>**MUTATED** DNA polymerase sliding clamp A (2nti_C)
LRIIFEEIQIMREVVKCMCQMIEDCIMRYRKETIDMICMEQCNVTMVTIHMGQDLYRDFEIH
EDYRYAYHSKFMLRVMRICRQRDCVDVCTDTGETIVVHVVATSHQDYHIQHMDITDKDVG
DVHMKYEVTCSVTTEAYRTCVTDITSISEHIIIDANDEQVMVRCDADTDIDIDYTRESAAMK
EMDYTRDTRHTFTCDFMEEIMTMSRMTEFIRVTYAHKRGMKMYYHLDAAARISFMMCGR
I
>**MUTATED** proliferating cell nuclear antigen (1vym_A)
LYQGKMIPNTVMRRIMQGMREMVHQGACEVTTTNIHMPTLETTWITMIPMSMKTQNYESF
KAEKHMGLNIHMSTLTRVMRAGNHQEVVSMKGQEHGESMGMIYQGDHPQRITEFQLRML
EMEIQPMNVDQPQFTAIIRLDTNQYGKVAKEMTWVNEGIIVTAGRENIRYTGTNQMNHNH
VRMTPSTHIERQQQGISVQLHQDIPMSYGMKFMHYYSRGSDMTTSISMTLTGEIDMIIQFRV
GELNWMRFFMGDRVQEQQNT
>**MUTATED** 4-hydroxy-2-oxoglutarate aldolase (1vlw_A)
LATERVNNNNNNLRLQQMYRRNRVICIMKCHTIQQCRQRCMCIYQAAINMVQVSYSIDEC
ESIVRQMTYMRQRACVVACASISTIQPWKRCIQTACQYVITDNMEQQVTPYWRQRAIYFLD
AILSDSQMIRCLRMANSVMRMYDAQIIADPYIRCLRADYDHIRYIDSAAIHMEHIWQGYRC
AIMCIAIATCMIRASDEQIKQRCRCYIQRVKAWSQ
>**MUTATED** deoxyribose-phosphate aldolase (3r12_A)
LNTERVYYYYYYLVQFKVQQGIGRFKQWFQWRDIKQTGNVQEIRTGVQYSHMRDWGSDE
EVRRMAMQGKQHKWYNIAIHDAFIRMGKQQMQNSEIRIISIINWDMNGHQSKSRGYQGV
WGIQTNGEQVELIVHINLMRGRQCQFIFQEVKTIIQTIRNRIIRIVVQSAFMESQQRVGGAIVT
RMGNGYWIRSTSNWNSNNGSGQEIYMLRCVINEQLNIRGTNNVKSWQEGIRLVLFNGEKV
NSTTNIRVIPNNQQKFNN
>**MUTATED** pyridoxal biosynthesis lyase (4wxz_A)
QHMADPYLSTLSMANAEKIRKNLSQLPRNNIVLEIIHSQPSRVSQSSNSISILSMQKIDSEVKS
SNNISKLSEDAIVQQILHSITVDILSRIKVNYFIQSKIMQSMNIEFVEQTQIMADSEQQWYVE
RKPWAIDWIGNGKEMNQSSKKVSQNSTLMKARNQDNANHVIQSIKYLKRIHSPVKRIIHLT
QEQMISQSRPMNSDIQIMKQVRKMNKMDIIHWSSNNIAADSESSMLLYMNSENIWINTNV
WRTQHDQRFSKSVIQSAAYFQEFQMVSYMTRNMNNSLKNVEVSAMMDQYKLPQKNC
>**MUTATED** ferric citrate transporter FecA (1pnz_A)
YYYYYYYYYYCEIHVCQATMNRCMHEFCCYTAWSMTINCTMSKARETHAMYANFNIDTA
MEEMMNATAMEIRQMAHHTGSMDQCQCQRDNCMSIIANGMANCKDHNIWDYCACKNIV
KKDNWCRSACSSLKDIMHKVQAITCQDHHASATYNMCLHWAVKAMHQKMCTKTSIMLN
AVQIQWCQFAEQEMTMCQITMAHLNCVNIIKAAACIKFAQETIAAIIHWISKCVQENWAVD
CAIDAEMTQSTTEHHQRDSYHMLIAASCNHAWASCMMFTASKATNGKDYTCSKVNNMLM
RTRFCQNDIYSWHTMMEFFNADCNLQAAMTKCNFNCNKGETSKQFNKWGAKKRMCTM
AFEWEQNTEYRWHVEAWFSESMKTAFMDEARKVSMTQKHFGIKAVDQKFTEVWLVAQTC
YDIAIAFKFMHDTSYDLKFFSCSTTAEMQTATTQFNKNSKTASDCYCGFMNNRVNVAHGSV
SQALKWDYVDTFEHHCVSASYDDITFHCQMQCMHIMFYMSNTGHMFCHSDATWASIEFT
EVARCIETAHIDQDRCKSGDMASKFNNACMSCDLAMWMVHWHHEFNTHESHNSISCKAR
SKYSAMDSECKFNMASMSQSMNHITVFCTFCFIHCDVKDRANSFAHMIQWTQRYRASMAI

NFRQAHGSWHMHTNWETTEWCNHCHSIRDTCNATSAKVQAWLMGACKICFNWAQELCN
MHMCWAIRHVWNENFWVKTFNNHHRAVFCAEQKSMFLEATMRW
>**MUTATED** intestinal fatty acid-binding protein (1sa8_A)
CYEASFRIAAMRMSVSNQADRYSIRQTTDYKDVEIIYQMAIEYCHTMCEASQMSASFSLQA
DRMIARYRKIEDARQMVCIKQVTADQMVNSHSHQAIQCRKVYRRQ
>**MUTATED** outer membrane permeability OccAB1 (5dl5_A)
CHIQMKNNNNNNNMDTDKTDCRAYIDECHATVMYQSAWMSQERRKACRESTTICKTCVI
TVDTAYSGAVIAYAIAIIAEATYRVADHRHCAHKLVGRNHEATCWENFCQAAATIRCQYTHS
SIQWASKIMEMGIMCTHSAQLIGDWYSASMMSTNDVRHMDIICARYSREKLTEKVHSECEC
TAQAMEQCVIFACRWRYHEHMHCTWWAMETRHCMDQNWCHIHYRKGMCHETTMSWEY
TAWNSRYECHCNSWTCSASICGHWCCEAVCADDRSHHVFCVTASWCSAGNTILMCWKKH
SAHIAWEWAKHCEAYKTVWMGHTWLTEYVAHNDRTCKVKWHIEYARMAIMGAMHFSSC
YIWAFEVRIQHISEECKDQDYYHKIRWSIKTAYCRECTMQVQHTWWQCTECWKACWVAES
HDFQVYMEVGIRMY
>**MUTATED** Kunitz-type kallikrein inhibitor BbKI (2go2_A)
TTIIIENHAPQITHASESFFMIQITYAYSAMSMSRVAHDSDQKSIIMEQYYKQAMQIKWDTQ
MKVHVVRDTFWMHVRWAQTTTETAIGEIVPPEQVAMSIRINENRTMMAQWRIDRDADAFR
VIFFQDKAPNAMEVAMIYKHERFFMSIREADQLIWRVKRSN
>**MUTATED** lysenin pore (5ec5_P)
ALTCRCCDAFDEVDINIICIYRDAFIFDHKATSTINERVSVSRALRHIHTDSKSISCSQTVATSV
TSANCWDVATIDITFTQTQERTEITLSESDIFTTRIVDQSVSVGGSTRWSKYEMHCNIAACAV
DFLFMVNDISGVAASETVGEIVSTKCRVVIAKEVVMARSDVKVRQCDKRDFLSIITKRTYGC
CSMAQTRMWRWIMFDNYAAWKVRSMHSLFTAFDFCFTTNEAAVFWNEASNHGREKYCV
HRTMGMKQANIISWLHRFWSKTAMPFNNAGCSHIFPMNRKDNRYVMDIIA
>**MUTATED** Winged bean chymotrypsin inhibitor (2beb_A)
CDYEEEMIEGDAHMIDSAASWWMMQNVFGNAAAVDSGRSAHDQLQMSIIKTQHDITRAD
QVKVTTPYMTMYVQKATMIGMAYGHQQTLGGTQFFSIIETQPAQGIRMTPPRMQDREVMI
YRYDRITNTHVNIWRMMWLPNEDDEIRLEPWVAVNKEKHAHKKMIISDDHQMDMIMMRG
RTDSGTTN
>**MUTATED** coat for the nuclear pore (3bg1_D)
CITIVHSIESTNQECVNEGPCEFFASKMGSLTTEKTIRVWEIKHAAPVMVGEMKANQADIYPI
GYGNDCFAHVMGTLTFEKRIVVYKQQHASYQRTNQNGANETTIHTILYGDNEFAMVMGLA
TTEAGVTMMSFSAQAPYQIRRVHHGNSVALHGITYGDGIIDATMVENDTAPRDHFVRKWG
TAALEHMVRMYRQQQEAPYRQQPRMQGNTEYIKEIGYGDTVAMDSTSVGTLTPEAKIWV
YSLEEGTTHSYTDRMMNRWHEIIYNITYTVSGHVMGITAAEHRISMYRQTIEAPYILVTEIH
RAPATITGTISQ
>**MUTATED** *Macaca mulatta* intraflagellar transport protein 122
LKCIMPFKNRCDQAVHNVCYRGNSPEMVMCCSHKMMIWNPTNSPMMEGMRSQRNPIWAI
CWCRNSRKYCTSTCNRTIVVFPTRMDSVMRWPQHNCVEAITWHGVPQEMCTATTTNYSM
FTGDERTITRQRTTTRVVAATFPHNSEWMCMSLYHSVVTVKHRHSDDRIRVDKGSSTMTGV
FTVAFHGTKDDKHNVMCICNFSERVTYWEMTSREVSRNKCMHYNGAAVTWYPRSDWVM
MSSTNREITMYPRNSIKMSPISDEHTFIFPAEIRGNTHWIIISAENSPVTYWEMVYTPIQSMW
RNKWCWKNTLPNIVIEQMVPDERIKVRARDMIRRVCVWKHKMCVEMGDRVMVWDMWT
DNTTNLQWKIRDRVVRRYDAHMMIIACHQVVMAEDRKMEAMTYTSIRDKDFELDTMVK
WVRIVSSGGSKDSMMISMRHSEVMRVYINHMYCVVMMRECPCIKAMNLTCTKRRMCIIN
DHNPAMIWNVNPRDMMYEDGHCHTICFHPEADNLMAYTSSSWMHVRCTPYGIQKERMES
YIISWHSTRVYAMQIYTVTCIDIGETCGLWEWMNKRMYRDCWEVCAMSIPNPNFKDMCLD
CMDSMNYDPCRRCYVKIENMKWMDMVTTVDDKRRKSDPHHNMYMCNIYTWESRYQDC
CRMWRKTSQDHMCMDLWPNMALYDWCRNYMSTSNGRDPRLMVPRECNFCKHVRDGRC
CIDLWVTCSDQIRCVDVASNQSFINLMVNVCKRMNRCDKDGMMMACPWMRRMNTGSW

CCDPWMRLSNMRTMIEMQIDPEKFNDCYCMSDRQGDYRNNVWIGWCEFMCDHNKYDD
CERCYQRCSKEKDCIEIMDEMPHHCICDTKYHNCCWWWFLMTLEAMNVCENKCERNLL
MSRYQQYEKMCDMWQSWQCVQKQPDNGYTIQKGDPMYHVTKYMMQTMGRNPGMSVT
RIRVMYPMCRETRCMSCWKMCKQCWNRMKSMWVGCKYERTVDMSPMPVKCRGYQNT
DDMIGMAWKATPHHGMMHHMSHIAVHAKEGYVYTCTTWNIMQMIDYWMDDSVPNDDC
VTMVNMDIGKGRENHKEEDVCHTTTEVMKMIDPRNTLSNDNGYPCRMTYDESSTDYIGIII
TKMIMKTLTKKNIMIRKFGGGMKFEWYKTMMGNCTVPLAGTAYELYQTDNWDMMIMEQ
SAAGWAKKARNNGSG
>**MUTATED** *Drosophila melanogaster* ring canal kelch protein
LVSMTSMMCRFCVAVLTHMTHAHTHHHHEEEEEEEEAEHDEEDSEHQAASASQWISDDDA
MASSIAISSLEEKHKMMEEEEEEYYYYEHDSSQATAMQKATPMMKFSTEHTMNQTTERYIE
KDHARQKACIAEFTHQEYCSKTWNSLHQLKREREMPNIVMISNNIQVYSYKLIMSTPTDFW
FSLWCTWQQTKESKVCMETINSKSMQMMVNFIFCSCIQIHQNHIEIMMCSSHMMEMCNIK
NSPPNWMECEMNSTHPMAVKQWSNVYSPIQMMHFSQCFVQEYWHQIVEWNQWMHMTY
QEIVTMVAHNKVTIDHQQKIFQPIVSGMKFNIDLKQEWCTMMLQYIKMDWMTRQFVCEKI
NRQVMMQAHVIPRHMVVQSMCFYMMDCQCRTSKCIDKRDIALDRVMMIVAAESDRSVKT
IQGFNMKQQRGFESSQLDHKKPKTAMTIMANRIFSIAAWHATMKIKCINIFNDSCNEGSHPT
HLQSKKTCMAISIMHAPVFSIAAWNACCAMTTSQLFNDRCNVGKWVSTLTCKKTTIAIAIIY
AMMFSIAAFNAWCKEPMTTIQKFHDNCNCGIHISQLTTKKTASAIAIMHHVMFSIAAYNAD
LIKKTIQSFNPQCHTGKTISNLTFPKKHSAIISYNAMMFIIAANNACTHMSTIQIFPDNTNTGK
VMDSMLCVAKTFSAIPLVNRDLPLQQEASMSKESSTMSVSMMNNQHTESQACLQASVAAS
VFAHMSDSAASSSSSSDSSDSESDEDHYDYFQHVFSDVAEDTHHHHHTATHTHESSSVSHS
HSDSHSQQVEEEEEDSDCQDHSHHHDEDDCSSSDSDTEEEEEEEESEDEEDEKVMDLHHFK
HNMFNKTSSAAIPTSFNIDKSIKTAMAFKKHWKVNLEHAHKPATAMKPCDMFCHTKTHPE
KEKTWNNCQTCNAFHMDFSASACLKFQHVFQEVKNQDMFKCTSSHKIDMFCKMNIMAYA
VAKVQKYMTTTPAHVNYFHMAAYFSIMAYTYWACIAYVKMHSHATAISSDAISACACPHID
HPEAFLCSSATCIDIQFSHIRIDIRHTSTTWWTPMYAQHTETLCHVFRCTACSSSLSSYHTDM
CDHITLQKSTKTSTSASSATSSSSIQQYTSSNTVDTTTHVHSHKCCASVDRIRCSHRDSRQTA
ATTCSSTDVMNRCCTCATARTICMSRRCTCSSSKTTTTANCHAHACMHKVTRTTMEGMMI
HRGMDMGVAEADNPRIVNWHWLWTKNPITPNCSTISTELTHDFACDKMTAMDENLIKWET
TPSASPSSSASSTCVKKNSHSTSKDMYTCMTKMKHAQRKHDHKISAHFEFQNDTFQHIYIE
GEHAWQWAKTKNFNDHTCFYEEKDMMEKSKTQTDCWTHEEKKMEKEASESEEETEEDR
DDATDNDFRHFRMHSNHHCWRDRDVSSNQMQASIAASISQVSMDQINVQIINDITMTNHQ
CQCCTTEHHMDTCCHTHHMHQYHN
>**MUTATED** 3-phosphoshikimate 1-carboxyvinyltransferase (2o15_A)
LCAFDSDASDADIKSAIAIDNTCTRAGKSMIMSSMSSSRNKNSTAVTNSMKTKEAQMLMES
MRAMNMKIENINTQMAITNKVQDNDNSKIEPNMSNAIMKWIDDMSSMNTIDIAWENERRS
KNKDVSDMMESMKQMNISIENANMDWKIKNGNTMSNNAISVESTSTTRWITNMMMTSST
WAENMAIRYANTTMDTSDYVSLASSLMKRSNIEVEETADGKFRIKDNDISSKKFEVQDEMA
GSISWMTSSIITNNAIKVANFDKITIRDSEYVMSVMKRMGSIIVYSETTMQIKNDANHENWEI
EMKSINQMADTISSMSSMSTDNTITKMTNVSYMKNYQAEKMSSMTAQVGKMNNAPKQA
DENMIVASADMKDNVFKSHSEYKLSLSNSVVNMKISNIQIEEVSSAACAMDQWDKMFSQL
INDNRNFNHDRDKTNRKSKKSANRNTNN
>**MUTATED** BamABCDE complex (5d0o_B)
LEMKRMMMDAMMTISMMTAGTMWHTQQNIIRLTDMDSIQHEWSDSSCYTSTIATAVAHW
FTHMPDCMCNHIIFCCNKCAMIRCMHCNNARQVYTITMCQRNAYWTRQDCMMTAAISITA
APIFVATQRCEIFCMHSTNASICYESRICAQCMTKDIITNAMIMVPSTHAEMECMHQCNACI
RYSIHMNLDTMTMKAQTCDSSCWACCIIAANHAKITCIMLQEAELVYEEKVTECSATSQVN
KMTNINSSDIIIHAIIWCMCFHAHMSCMNMKTAEVLYRKQMATIHNWVINAHKVFMINEHN

KILCMSVNAAISMYSETNMMPKMMSTDIMFHAHMIIANTQAFMPYVHIQNAKWICEERIN
TTAWESQDICCNARMMVECRNASIFTVSK
>**MUTATED** enolpyruvyl transferase NikO (4fqd_B)
LPEKCKQDSDDAQDMMQVYAAHKMTASIKNTAWRYTMINNISSSSNSTSDIKVQHGDEVI
QNSIMAQVWKSSASYSYFEASEQNWNIESTSCEKSQMDSEMIAKVYATMFMMDSMITKHA
ISKMTSTAAGDVAQADKAKDEQYMMEILAKWAINNKMNSEATIEMNSPKMNDGNVELME
FNKHRSMLTADGFTASIRNSMMLASINYANNNMPYDFMRDEINELINIMKEMASEVQWSA
DQNCIVYAKADQTMYKDIEINMVDEMVQIINCVGSAIMMSEQDMKVNADAVEKSIYSMSD
QWEMMEKLAIKIEIASEQINSYDMNRDMKDIQWNSLTKAIWTETPDWMSMMASFSQADN
FVKQSICQYKWAWSDQMQSMAVKNSIEENIMKIEADGDDYKDANEMKSNEMKSSSIMMM
SSMSIDAKNNMKHYYYMSKAFKEMIQEMIRMASEVKYNNSDEIKDRDDSASAIEDSYYYY
YY
>**MUTATED** complement component C3 (2a73_A)
TDLFTVVSDHVMKMQTQQSLIMQGPNGEANIDISISIPNYDARRMIMTTQRSIMSDGSHPLA
HISYSVDGHKQYRTQRAKHRYISIEGSYASEIIQRIIMITMETAFMYVESNRSVFSDATSIMFK
VVYSIHPRMMDIAKSILIHVQHDQAVDIRENTMTTEHEMAIMDMTCNVDQMIHLAECRVKG
FFQHTDEEIYTSQYQIRQFIMDTYQIVIQDSQRYFFVFHQRAMQISVSGKYMFARRIQASGYI
VYAVENAQEKVTMDQTMRKVDVQNATAQIIMTKRIMMNAIEHMKGQNMIARTMFITGSIV
MPTATNLIEGQKTAVDVISTDFEVPYSRSDRFYRDALDYNMLIYISHDNATDGFKIDIGIEAQ
NSIETMSEANAIGRMTVHSPDTERDMTVSIKSRREQMTQGQEGSKSLEGMDFTSIAHTHHF
MPMTIMKSQMKDAQSMHIHYMMKLNKGPQGRVKFFSFMVLHRAKMMRGAKEIKQDAE
NMIIMDMTVSSNYVDTYKMIGFFSMVAGTAEKQIIGNTICINIRNTWIATMIIRTAETQNKEDI
DAEELSMRVQANPAGKIIMIGINRAIYIMHRRHRMSETRVCNIIQRGNVAWSDATARNFGAI
YTNGAMSYSTTTAEESGEKGQMEWDED
>**MUTATED** squalene-hoplene cyclase (2sqc_A)
LSQHMIQSDSFSKCMEKSIQFMMTPHREQAFYYADMMTGICLQSQFIMMPNVMEKIEKEK
LQRVKKFMMNQHKQEACYSMFDAADDEMECCVQSFISMRFVALTKEQQDLHRSMKWVH
THAAVQTTKIWCKLYMSMIAQFDYQRIDLIDDQVLWMARKLDMGVFQWATYSKSCIISMT
VILTKHDIWDMDQKSKIDQMFQCEIDDKKKASRAAAAYVWESMEKSMNAFHRMTINDWK
KSSQVKSMEYMMQKHSAEATYAAVHDDYWFSMVSMRVMELCHNDSWVRAYQAMQMFA
IQMEFAAYLWHSTVTDIYECAMSIMSMKSSAMDSENEKMIRSAQYMMEKHVCIDAEYSIR
KDGMRDAAWSWHWEGIFFDEIPECSIIIYSMGCMKMDEQKKKKESLCRAWKYVIALHTTG
AAYASFEIEGCTEMDGNVDWTEWAQICEDDTQEICSNIMQPWATWAFEESYRIVKKSIQFM
RKQHRDEATYWAKYAIGFMFACASIITSMRSIAVECKQDFVHRSMEYIQHNHGDEAAYAQE
PKTFQEDSFSARASTCDTHCSYSMLSMVSAAKSQTQSSKKAIHFMIQCHKDEAAYEQDFFC
ACAWDAEWFMAFCLFKNIWDCMSMAKFRHSVQKK
>**MUTATED** thioester-containing protein I (2pn5_A)
MMIIADRYVKGHEQFSMIVTHYHTEMTRINMMMRMQAQSNHAMTIMHISRLINIKKHLHK
LVHYHLDQNMSGAHFRVSVNAEKAYTYPRQGQMIFMTRTVTAMVEINRDIYRDANSIHYK
IVIMNSQMRDDGKIRTIFISVKNDEKHIVKRCTSGRMFGAIYQTNMEVGDSDLMAICHVTIQ
IQAQQMITRSYQIRQFIMTSYNIEILDTIVDMQQPEGIHMSVQGHFPYARDIEAIGRIQMFMN
NNRMRMRRQMSIFARAEIQMKYNHYGLNGNEENIDIRITYIQEFSHKSIIRETEVSIFKFGFKI
QMVRQTDEYKDAMDYRWGMEYSPPNASDGRAVTARIQITNIKYQSSSSTNHNAMVRMQM
EDTQASQEMTVPYHGINAYYYFQNIHRIQSISNGFVRMQMRTDVRKHRMLKYLISWSQKL
SYYIFFILTRAHVVNGAYLKDHREDRFMMEMHGSQRLVDKGRVMVGSIGAKSIIFNYGNM
GYEQMKHHYNMTVNQEQVRDAKEVQMTLTAKDAGFIAMGGFNRGMMMYHRHPNMYC
QNVAEIYNAYPGVHQHQYNVYPTMAMYGKSMNNVMYNTGHQRSAKHGMETARDVARM
ITFKSHYEQTCMCRHITVAKTATKRMVQIIDNSSSTCFMSAYTVNDIFAMAVVRRDVEYSSIE
DYFVIQHMDFTVRKAQGIIMEYSMYHHMAGQFVGNISMFHIGHESQYIAKDHSNMTFSRTI
TIDDRIAIDVTYMVRGKRMAQLGIKIRGTVLMAPQSNGMQRIVKILDQTMIEDKLNSKYY

WYNNPRHESYDVHMNVHRRGNTATSRVQYKMHDHMMSSIVRHMNPMMAIDSAWAQEH
LIRYIDHVMIMNFMPGVATRQEPMVNRGSHMMKEAFEHELKFKESNATYAMCQSSHATIY
MSGYIASTLESGIRFVTNVNGGLIQRGMNCMGTREPYTAKYNRGAGQFPRQLEAAMKHAI
GMSTFIMLGMMQHNVGRGRPGQIVERALSFMTHEYATVHHGFNMTVGSFGLLMHAPSLR
QQGMHRMVNLTYVNGNRHQKYCHSSHDVQSSGFGMMTYILGQRFSNAVDILHCMIHEKFI
SATYDTSENSYIAMRGMSRLGQRVTDTKHNFSIEMRFRRTGRFYRVHTQEVNIQHYINVDQ
NSRRMQVHIAAVAYAMMQIIFEYHMHMIHYQHKYEMNMQREHSATNFQMKMRIWGTFV
DEMSNKKTHLGMVQISMDTAFIINKHDVTQESRIHDVERSQVKFAASTIIMFFNHLATQKH
WYSMSGFKKYRIGMRKDGFIIIFNFFHSHMHGVRIFQINREHMWQVWNQQNWDGQWAAP
PPPPP
>**MUTATED** eukaryotic ribosome anti-association factor IF6 (1g62_A)
LCSKSRWDHTHDVNIWTPMSHSFAMICINNTDHWFTCWDCDMNECVQVIYSSVCNSKVV
NKLSCNHKKNMMIQSRSSERDMRYMKHTMQETIPVRKIDDKMTCMNHIVAAHEFICMIYQ
EVEKDSDDMVTEIMNIDIWKRSVTNHVMINTFATMTHRNNMIYQRSTIRERDDMTTMMRI
QMICNSIHKNTTIINCNLIIHEFMCISNMESSCQDMTIVDTVWKM
>**MUTATED** prokaryotic ribosome anti-association factor IF6 (1g61_B)
LSLVVKRFYTNVDSVNIMGMSSQQVSMMDVYMEREEIHQITQIMQSRAMPSHVNNTTMIN
TMTIGHRFNMMMDRVIQEQQMEKVRHYMRQHHMEMHIQVVRTRHSGMNHMVMSHERN
GMVTDQMREYRREVQETMHIQIQVNSVGQMDSINTHGIISHRNAMSWDMIQEEQMQYMR
TMYRIQFVNRNSGHRNSSTINGAVVGHTRNGIINNESSNDQMMVVQEGMNMV
>**MUTATED** 90S pre-ribosome (5jpq_A)
LDPTHRPNKRGRDRHSGRRRMNSPANHGRGYGIGGQARLGKSLPKTTEIHDKRMNIQLIEK
SQDEEQQQYVIGIIAQQASARSSMVKTMIKKLSRTSMHEVPAQVSIITARNKKMSYMDWQG
EEMHGLVEVGRVGEMIMMMVEAHYAYDLDSLDYMHVGPNNALQKIMAIGSNMEMYRTP
TSMKGTRRKMRNKYCSDIFPAGRMYFMTAIVHAKFQEKDVMHMTKYVTILRYKQMRCK
HDNQFLMGEKYSEMSNQDMVDSPAMPVEKRIGVFAFMNASQMQTGQASKINVGAIAEYTI
GPVDRMQEQWQSQYFPPRMEEYDKDRLRDDGRGHADVSSGTSSKKKRKMEERERMVFG
QLTEIAAIMLEREGIFVEVARRHDDQTYIQAPDKADADRMLSAMPTIDPTVGDRYEAIAMP
MYTHASDMNDIGENDALEIDTADDTVDEEDARTRAKSTMKRQKVFARQIPDDEGEVEHM
QTEDDQFSHEEEIPETDQKLIDVEYHHSADPAGDRMGMDSETDYDDTDEDYTCDKSGGHR
MRRSDTRRKSCHVARMVFLEHVTQDDWVKKCKADEEETREDTEVDDEIEEEYYKRREASI
SRDAHRENGIEMDRYIQFYESYDRMGRRCRTIEGVRDKYMAGAVMAHEHRSRTETHDAA
DDMFAEYDEMDEAHQTDPGDEHTERDTDEDEDHDESHAEEEHTYSHYEGDDRREMSLDP
DKDLHGGRRDRMKGPYDVDDADHYRDEEDHHDFESCFDMPRGRVTRPMDVHHVDFPDL
SQDPKPKVDAYRGATFIKVIYDRIQLDYIRHYHQRYQVILAAMMQSDVRYAVIRGKMKKN
KCNRRVMRSHEQMIMTMACKKYPSMQVFSSSETKSKSKLMRFSQDNSFWHGGYFAQMW
TQHSQYWAIPVIGHTESAHAYKVGGSAVIDDVEIHVDVIRRMRMIAYQFRVYRHSGYVREL
YTTGLDIGKYDAGPVRSITAVKADVRKGMTRQDANFKGGYDERVMLTEVIVMKTCFQIKIR
RYFHQISTMMMRDRSDCRAMKMSAPVKGGLHMDSQTHQETGFNRVDKIDKNYHAMRIQ
RGIPRDMQYRTPVNPLRQPRRRSFLGRKGIIMAAEDRRGKTYVPRIMSVTRGRETRKRDPR
GTPKRDKMRRMGRLDDDRTPKERDRRRDFYGPHARKSSLAAEEDTKQKRLKK
>**MUTATED** a-L-arabinofuranosidase (3wmzA)
CATACMKACATHKPMNIMAATENNACMMEMFNPYAASHEEYSTSNSAKMSIFANRPMNID
AGCSCDASKIEVYTPTAACHEEYKIHTNASIIAIQTAMPMQCCACASCHASCIEMYSPHAAA
HERYSAMSASDDSNASPCMDTSFKYTTSAIMCEDRTAYICMRNWSSISGHAKGMIFATSTTA
TTFATLIWTDWSHYTNLCTCAEHCLHECCICDSMWFWCDRHVYIMCFEYATYDWVFKSTT
NDSNDHAYTCDEDMWSATVTATNSADVNESMVCNAEHLFMWWCANHARVFKCTLDVAH
WDAHWATTFSSVLTNSRCHMWQAIEIFRIEAEHEFMLVIQCLACHAKFWKTWSCTTMTAT
YSDECCTQAHDWCARCHTACSYSHNVTGANMIKNHDNESLSINDPHMEWMFEARTDHCA
ANFHTMDYKDAIMSMKK

>**MUTATED** *Heterocephalus glaber* regulator of chromosome condensation/RCC
LDDRKMCRKSTDDQNCCDRTRRLRIRDSCTKTADTSYTPEITYKTYYSQCAIIMSMAEANI
AEMAMAQHILQKRRDCMIDVDQQIIECQCAALYSIPMTRTAEVFTWAPHNQACMAKNSTI
QATQLIDARIQMEQRIIEITCANTYSCCMSQNAKIWMGATWKNHHAIVAMMQDLRRTLIDI
DIQMNIDIIRICTAHNYMILMSINANMFSMAPAQEAEMAKIDQMWCHKAAKRAMQKMMI
DRPILMRTKATKAFIKWENCWPACFWSWIVTKQAYVFAWAMTHFYEMASDASQTPWIDEH
MSTWRHTSRTGIAWTAAEYYSIPLNTQARCFTMAKCQFAKMAMAQACQQKTVDSMVTK
MDPITTICPACTIAFCISRNAKIWTGALASHFEMASAENNNCGTDIQLSAREMQHKIIMTITT
AAEYSIMMIRNRQET
>**MUTATED** b-L-arabinopyranosidase (3a21_B)
CISSKEVSIDTCDLAYCTYHTWCCRVNFTIVRREINCWICCAMDCCAFSFVHVNQAYYEAS
KNTCAHVSINSCQYDAALTCVSCFVGTRAMRCAVFSNCARNAPAFFFDSAKDCCDATATQA
GFNENLMEWTSYAWNWIRINYPAANCQAMNCCSSFRTVTNCIAKCCCSSAKDMSMTVPH
YAFEHDYHYCCAECDMYKSTSNVVFFAHEDTLSTMMTHWNESMGDSCEGSAFFHNDNL
MLIALNAWSCCEHKSGLHMYCVTACDMMCAHNMSSLSTQSCAVMRHDQIVCINENTKAM
EAIRICQNSSAMECFARIMTASAHKCIIMMHKSTCCGNVSIKYTNMAMSHCTCSIKNMYCK
EHIASTCSAFSCTIDCAATILMSISAASQCCAACFCCSTSAKFSAISCCTSAMHIINICFSHHST
TCKSCSMEIHAESCSSITWDDSACTCASITIQITMTRATCHSMCMTAADCSQAVSIKDMDAS
HACMISARETAKPCNVFHHSVSHASECQMYNPHAADHETYSFSTKRQMIMFAHRPMNCFH
MASSHASRIIVYNPHAECHERYHVHTNASVSHIHCAMPMNCFHCCSCHASTMIMYTPASA
NHERYSIS
>**MUTATED** endo-1,4-beta-xylanase A (1knm_A)
DGGCENNRVQNINTNKAMEIGECTSTENSRMRMYEAWTNSHRRYCCSECNDMKIFNEQA
MECCNSTHNTQIRVFTAYNNEHRQYKMHTENTIINIRTNMAMECINHNSCHNSMVRMFSAT
HNTHRKYSKS
>**MUTATED** GalNAc/Gal-specific lectin (5f8w_A)
LSSWMVRYRCTNRWMYAFNNTTDACDDSRMIMYTEVYQKLFWHWEIIEQKGNFVRYICT
NRVIYAFNNHCDAADQSDLIMYHEKYEKCMWCLEWWDEDVLYRNNRFVYARNNTADAA
DDSQSIVYNERYCCLQWVWITARDRERKIMIFCYYYYYY
>**MUTATED** pancreatic secretory protein ZG16p (3vzf_A)
ATCKTTTFTADFATAAARKYTNTAHPMEAQVSCMKIKIHSFFVIAMPIKFARILTEFIAAKHA
EMDDVYMNQADTIVPITARFRLFMRRMIYISERAKFMTYARETASTYHCIQMNQHSIMKYV
TAKTATMVECVAMNLEI
>**MUTATED** ripening-associated protein (2bn0_A)
LHACVRIACGAAHAATCYELADCFKVVTIRVYTAEIIEAIEISYSFFARSQSKNFAATAASDN
QVIMPQAQFMIALCAQICHFNACIIMARMAYTSHRRCFADYAHSAASDYTMDVCCARVTA
YYAKAARYMECVAIFMQD
>**MUTATED** vitelline membrane outer layer prot. 1 (1vmoA)
KSKDWSTIVSIQHAAGFARFAVKEYLGTAWCHAYCMRIDQTEYAKNNSCMHAVKMKLMN
ATIVDTMIARFASFSTYMILQSAWMITYTMKTDRTEAAANNSCCHHVEYKLTNDCIMIANA
MTFAKYAQFTRKLRVLAMESRIDTQEAMKNNSCMHHIKYYLLR
>**MUTATED** alpha-11 giardin (2iic_D)
AGMATFAESVGQIRSVMQSRDQQQMIPYPTKCTSQQKRQMKPHYHEPPAMQYVSYMRRW
VRDAGFQEILSMACEWDVTSKIDIVRRSLRDIDEYKSVNEIIMVSPGEQKMRMSHSFRQRPA
DEMMHEYIEHVGMPTSSTFMWNMSVKQDKPGKATISTESQIMRNDMVESEQGENQSIIKM
VVPTPSEQFRQVDNKYQIMPARTIHQSVQPKFSERQDSKAMWVSNFFDMSGSKSISFSYNTS
IQPHDEELSFQHSSKVPAMYNEMNRYSCINFSWCAILKEEVMTKYHTRQSDRIDYKESWML
YCRMSR
>**MUTATED** annexin XII (1dm5_B)

IIKANIRGYSTWHTQDESDNMQRSLRAVANEDRTVNYVMSNQTHSKQKKVRNEFNNMWA
RYMDEDMRTDMTAHFDSSSMSMMQRGEDWMSDKMYSSLRAMANERHSMVEVMPNKT
HSKVYSVRSSWRMMFRDEMDRDVVTDNTAHWKQMMITLMKAAQRDEDGIHSSYSSDES
SSVFKSADAKVANEDTQWHSIMSNQTFGKMYKVWYDFTRVTHRNVMKSVDHDWTAEVR
HAMMSVIRTIDHQWSFWSDQMYYSLRAMANTERNMVQVMITQTDVEMSHVRDNWKSLF
ARTMFDWVSEEPTAEFREMMMKVNAY
>**MUTATED** hypothetical protein EbhA (2dgj_A)
LSLAKMKYAVEEDHSNRKNKRFQESDKTRRNSFEKSISSSRSVMHRKNATHTERSSIEQSM
KKINTNRESMHAESRMSDSRSSSQKHMANMHYVNHSKQNSMDAKVHKSNNIEAIHNIRN
HSHNMEASLHTMKASVHERESNMQHKHFMESEDTRQHSFNKSINSSDAVMHRKNAAHNT
RSEIEHSMHSINQSRSSMHASDHMQHSRNTSNHNVHAMGHMNKMKREHMRYKIDKSKHII
AIHAIRERAHMDYYYYYY
>**MUTATED** accumulation associated protein (4fum_A)
AIEAEDVSTSQQVDYERRMQYEDHLGDASQRIIKRAQDASRSVSSDSSRHDLSAQRIAQAQ
DSQRVSRKDIEQVINFAAQKVDKANREQYEDHGDIETRSQIDARDAIRHDESAQIISDDIEEI
SRFADIEAETVSTSQQVDYERRMQYEDHLGDASQRIIKRAQDASRSVSSDSSRHDLSAQRIA
QARTSQRISRKDIEQVIQFADS
>**MUTATED** CD55 decay accelerating factor (1ok1_B)
LEPANMQQPIQHCEQCMDNKSTYQDPSIVSWRADDTYIRVQNDRPTIVAMRNTEFTPVDDY
AHKTADIQSKMHTCTMREQWVSEHWYQINSIIDWDAKQNWKKDQTMTQRMSAMEHMR
FTSCIDYARRRTAQHQNDVKHNEVPIQNNVMYNCSVTYTAHSNWRMYNTSTTYAMVTNT
TIEFTPQMQDAKDVWAQCQQEVPHNVVENDKPGWNWKETISWCAHRNYSLVNDGTVWA
SIHHPDNDFTNQQQDAKNA
>**MUTATED** complement control protein (1y8e_B)
AASVDTKDVHLRWRHTIQSECHCHFHVNESVQFMAMDNFKRPRLNDVFCRASNSNYSM
WHPAVRKKADTDKEVEHNPMEVNNIEWNTTVSFTAHTNFGMVNQTRTFAQMNTSNTLIY
HDQCDVAQTIRAPTDDTVTHNKGHNFQEWFSENTIISFTAHTNFTMVNHTNIMATNNQYTE
DDSAPVIRADGDSVTHNFMTTNWRKTFTFHEHIEWRARFNFRMTNTTTTSATDNHSYRDQ
MDRAIK
>**MUTATED** Aart, designed six finger zinc finger (2i13_B)
VTQWNTTTTIGEGGMQDNQRDFGADQANRTWTKTPYMGQYEKSYSNQRDFRADQANRT
WTPRRPMSKYEKSYSNQRDFRADQANRTWTEKGHMKGYEKSYSNQRDFGADQANRTWT
EMGYMKGYEKSYSNQRDFRADQANRTWTKQPHMYSYEKSYSNQRDFRADQANRTWTK
KPGMHIYEKSYSNRRST
>**MUTATED** histone-lysine N-methyltransferase PRDM9 (5eh2_F)
NTDRQFIAKDANKNWTHRTYMMKYEKSYSNDRQFIAKDANKNWKGRTYMMKYEKSYS
NDRQFIAKDANKNWKGRTHMMTYEKSYSNDRQFIAKDANKNWTHRTYMMKYEKSYSN
DRQFIAKDANKNWKHRTYMMKYEKSYSNDR
>**MUTATED** ser/thr kinase (3m9g_A)
FDDSTEIVARNIRDCDKVYHRHHMRMARVNQNFNERFTDHDVVRSSTHSADQIDQAENIEI
IVNRATDRIRLTHIVAMTRDDCMKRMRNMAMREISVDRIFHHKCTRAFVCHKNISCHSDVC
VGENHVRMFDNMAVRKIFIDEYDGRNYNRCRRCMDDRAYRIDNRDDFNEEVEDAEIVNK
NTRARNIEDANSVNYIINRA
>**MUTATED** C3 convertase (2win_E)
TDLFTVVSDHVMKMQTQQSLIMQCPNCEANIDISISIPNYDARRMIMTTQRSIMSDCSHPLA
HISYSVDCHKQYRTQRAKHRYISIECSYASEIIQRIIMITMETAFMYVESNRSVFSDATSIMFK
VYSIHPRMMDIAKSILIHVQHDQAVDIRENTMTTEHEMAIMDMTGNVDQMIHLAEGRVKC
FFQHTDEEIYTSQYQIRQFIMDTYQIVIQDSQRYFFVFHQRAMQISVSCKYMFARRIQASCYI
VYAVENAQEKVTMDQTMRKVDVQNATAQIIMTKRIMMNAIEHDKCQNMIARTMFITCSIV
MPTATNLIECQKTAVDVISTDFEVPYSRSDRFYRDALDYNMLIYISHDNATDCFKIDICIEAQ

NSIETMSEANAICRMTVHSPDTERDMTVSIKSRREQMTQCQECSKSLECMDFTSIAHTHHF
MPMTIMKSQMKDAQSMHIHYMMKLNKCPQCRVKFFSFMVLHRAKMMRCAKEIKQDAEN
MIIMDMTVSSNYVDTYKMICFFSMVACTAEKQIICNTIGINIRNTWIATMIIRTAETQNKEDID
AEELSMRVQANPACKIIMICINRAIYIMHRRHRMSETRVGNIIQRCNVAWSDATARNFCAIYT
NCAMSYSTTTAEESCEKCQMEWDEDCC
>**MUTATED** calcium-binding adhesin SiiE (2yn3_A)
STTHCTIISWAEVIHEMVVLKNSCDCRAKMVVSEADNHSWSMSITEHNRFALCVTWTADN
RYSVSAIECVNHQAEEITMEVLRDITIVAMATEAEANSINEHVSQERKTSYVVNHMDAEIIII
KIEVHNSIWHCDRHCENIFYYSTNSTMCENAWSVAIVCAECCNHKRHAMTVSISVEASMSI
TDVCMCCNDEHNCAEAEHISHPSKTRYSMKPVECEISNISIHISPHNISEVWKCSKNCENFSY
STTCCFHENHWSMAISIIEQCNHAKKACAMCISIEASISIS
>**MUTATED** receptor-type tyrosine-protein phosphatase delta (4rca_A)
DSQQKYSKSQINESAITAAICTYVWECSANQKQRVIGHRRARRITHEKYDIVDYNNATATIM
KVEQMKSQKNDCVFDWICTHHIADVTITSKMSIMKDNEVQKAYQSVNLAQEMRIIDKSKS
CSLMWCCTAHQNQDVSGYRNYMQINSTHHHAKVREMKTDTVAASQVKACMEVDETDDT
NEARFDWICSHTCASKFTCQCHMFIKDMKDIKKIQQKYTVQQSHPDVLQAATIHVSWICIA
TQLQFIRGLMACDNMSQDNNLQVAKHIMDMHNIKETCHFSWICLTSMAIVDCVCEVSIRC
MTKMIQK
>**MUTATED** Down syndrome cell adhesion molecule 1 (4x83_A)
AACNERADIYMRQDSHKVNYTHTSACQVQWRCTAHDLDQVVGVKTNASCIANIDAMKE
VTTNARMIYDDYKCQNFKEQIPCEIFCWMCKHEYATVVTKNIPIKCIIHEYFQCQVLSQFIV
KAHCCIMRWTVDTYICNYIKIQTGVNNQAHIMTYTNHFNARFMIMDTAQMPVKQIADQNA
FRTFEWKSRPKMSAQSKMTCSRAKMIVSQDIATRCDSYCSCTRVTTMMATTTTNVIMMWE
CECYDIDFSKGFRYVQASSKRECIIMHNKIREITASMVVRNCIIQNTARFMWIIHHTIAAQTIQ
SIMSISCDMTCRVNDDSESINYAKDCIYSWEFSAHDVRSITGLRNARCVAPTQDIMKVQTIR
RQNRALFEWYIKHNEQTCQCTCQMRMAA
>**MUTATED** EP-cadherin (1q55_D)
LATSKMKHGTICMWAMMWMMPIIDTVHGEITAWRDAYTTGQFVYTIHKKQMQKAKRMA
RIHYTEWSSKRNAMFEIAETKYKIMDEASIMIRKNIRMNRESRYSVTSCEGKAVRNTSHVGI
GTRKNKTAQQGNTKTTRMDIMSYDQSNSAMRKRRKECIVDDVRITQHQKADYDRKMIPV
RTHREKYHRIFFTVSAPAGEHDDPAIYKVQCQSACLMISKDMEKQQFERFIMTTNGITQHAT
DIQQDLQVSVHIVEPHEHKDRYSPEIYKATIKQAIPDASPILGITGSEQEEHVETMHAIMTFT
VMRPEDQQDVDHMYSVHKQSAIVTMVASAMEKQRYDQFSMSIPGSEMQAGAMTIQARG
VVPVSEGHEHGDVYEDRSFSGMIDQHQVAYQIPKMTISEMELDASDGCPGIFRVKIHQAAY
YHVSSEDQTHPAVMSSGRAMEYQMKRPFIMPVSIQHGQDYTIDMDSTSGSISISIQEIHQGD
YYIDGITKIEITQEMTKAQRVVTMIGPEDERPPVPRMTFYVAHEDGKCMSIHREHAVISAHA
HMEKQTQFIRHHSFSIVLMISEEAITIASASASMVMNIMEIHEHADIDTDKIYSLWEPHDQDP
IMSVTEGEVDDHSFDFRITMTNATEMSCRGQMETRASTLMMTDSPPMRRAEFTVFIMMTE
GPHHDPMSIIHGSIWTWQARGVRWPQRMIAAYEMDVVMIVMATIMGMMVMYMMMMM
YMRKRRIIRQDMMMDQEESKEHVYFFAQQAAAQQEPEFEMTPMNKAMETKDEVLKHEII
DSMLDGDNFKDKDTHDEQVAHYVEQHMEGGEHEDSGDDFETMMIYEFQATATQGGTMTT
MHTTHTHEQNEFHFMTECATKYKRMGELFAAEEEQQ
>**MUTATED** killer cell immunoglobulin-like receptor 3DL1 (3vh8_G)
YYYYYYNTNTEEEERNTYLNNPERQWMTCGQTCIIQKNNYISMKAYFKYKWHHWLMFR
DEKVYVQVWYNKVWPDTWHLTQISSCYCNHFSAKNTYQYTQSNGTCQTHQIIVLISNHYK
RQTMMCYQNQMIRTNDKIVMPAGTEVLWDYWWMYRDNVTREQTKMINPVYENITRCH
WTVNQLLMCMCNSFKAFNTISYSQFPMTCQTEQMEVIISNQFDRQTMTCPQNQRIPCNDTI
SMTATTKTTFELFYMTKDNNCYDKKMQCIKRIHKSWPCEWQMNQCSYNNSFKAWNTWK
YTQFDGTEQTEQMMITISNHQT

SI Table 3: Analysis of the clustering methods for hierarchical clustering of the RepeatsDB set. The numbers indicate the total number of clusters in each category. The scoring at the 90, 60, & 40 % sequence identity levels as well as the grouping according to the RepeatsDB sub-class are indicated. The optimal methods (Complete & McQuitty) maximized all these values at a non-zero height while minimizing the number of groups generated.

Linkage	90% ID	60% ID	40% ID	Structure	# of groups
**Single**	1305	1318	1281	1463	1554
**Complete**	1422	1437	1391	1603	1714
**Average**	1129	1137	1101	1319	1439
**Centroid**	634	633	632	646	658
**Median**	395	396	392	421	441
**McQuitty**	1448	1462	1419	1629	1725
**Ward**	1059	1070	1023	1283	1478
**Ward 2**	892	901	857	1109	1326

Notes on SI Dendrogram:

A dendrogram of the clustering of the proteins in RepeatsDB follows. Clustering was performed using the dendextend package in R (T. Galili, *Bioinf.* **31,** 3718 (2015)). Clusters were separated using the McQuitty method and colored for ease of identification. The colors themselves have no explicit meaning other than visual identification. Zooming in to 400% is recommended for detailed examination. Each protein chain is given a set of three cluster numbers (based on RepeatsDB classifications at 40, 60 or 90% sequence identity respectively), followed by an asterisk indicating which structural sub-category(s) the protein is included in. Starting closest to the sequence cluster numbers and asterisk in the first position indicates the protein is part of RepeatsDB sub-class II.1 (collagen triple helix). An asterisk in the next position indicates sub-class II.2, then III.1, III.2…. until V.5 which is indicated by an asterisk at the final position nearest to the PDB ID (http://repeatsdb.bio.unipd.it/browse). We found no obvious connection between structure types that was not already explained by sequence identity although this data set is limited to repeat proteins present in the PDB.

Notes on UniRef90_DOTTER_MCL_repeat_protein_list:

We include the cluster lists obtained for analysis of UniRef90 by DOTTER analysis and clustered by HipMCL. Each line represents a separate cluster and contains the UniRef90 names for all the proteins in that cluster. Clusters were ordered by HipMCL.
