# Supplementary material for: Self-analysis of repeat proteins reveals evolutionarily conserved patterns
Source: BMC Bioinformatics. 2020 May 7;21:179. doi: 10.1186/s12859-020-3493-y (PMC7204011; doi:10.1186/s12859-020-3493-y)
Supplement: Supplementary file 2 — Additional file 2. [file 12859_2020_3493_MOESM2_ESM.pdf]

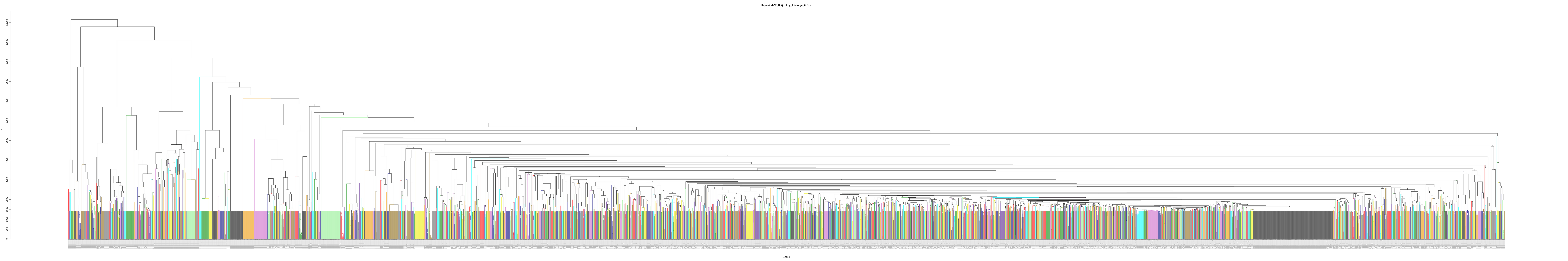
RepeatsDB2_McQuitty_Linkage_Color
0
11000
10000
9000
8000
7000
6000
5000
4000
3000
2000
1500
1000
500
0
Index
